# Divergent synthesis of chiral cyclic azides via asymmetric cycloaddition reactions of vinyl azides

**DOI:** 10.1038/s41467-019-11134-8

**Published:** 2019-07-18

**Authors:** Nuligonda Thirupathi, Fang Wei, Chen-Ho Tung, Zhenghu Xu

**Affiliations:** 1 Key Lab of Colloid and Interface Chemistry of Ministry of Education, School of Chemistry and Chemical Engineering, Shandong University, No. 27 South Shanda Road, 250100 Jinan, Shandong China; 2 State Key Laboratory of Organometallic Chemistry, Shanghai Institute of Organic Chemistry, Chinese Academy of Sciences, 200032 Shanghai, PR China

**Keywords:** Asymmetric catalysis, Synthetic chemistry methodology, Homogeneous catalysis, Stereochemistry

## Abstract

Vinyl azides, bearing conjugated azide and alkene functional groups, have been recognized as versatile building blocks in organic synthesis. In general vinyl azides act as 3-atom (CCN) synthons through the fast release of molecular nitrogen and have been extensively utilized in the construction of structurally diverse *N*-heterocycles. Keeping the azide moiety intact in organic transformations to synthesis chiral azides is an important but challenging task. Herein, we report an enantioselective copper(II)/BOX-catalyzed cycloaddition of vinyl azides, generating diverse chiral cyclic azides. α-Aryl substituted vinyl azides react with unsaturated keto esters through an inverse-electron-demand hetero-Diels-Alder reaction to afford chiral azido dihydropyrans with excellent enatioselectivities. In contrast, cyclohexenyl azides undergo a diastereo- and enantio-selective Diels-Alder reaction giving important azido octahydronaphthalenes with three continuous stereogenic centers. Notable features of these reactions include a very broad scope, mild reaction conditions and 100% atom economy.

## Abstract (web summary)

Vinyl azides generally act as 3-atom synthon through the fast release of molecular nitrogen, whereas keeping the azide group intact is more challenging. Here, the authors show a copper-catalyzed enantioselective cycloaddition of two types of vinyl azides generating a diverse pool of valuable chiral cyclic azides.

## Introduction

Organic azides are energy-rich, flexible intermediates and have attracted significant interest in recent decades^1,2^. Among them, a special subclass, chiral azides, are extensively distributed in many bioactive molecules^3,4^, and are valuable chiral synthons in organic synthesis. They can participate in diverse transformations, such as cycloaddition, reduction, and aza-Wittig reactions to give chiral nitrogen-containing products. Chiral azides have been utilized as key intermediates in the total synthesis of many natural products, such as callipeltosides A and B^5^, and pharmaceuticals such as the antibiotic chloramphenicol^6^. Moreover, application of chiral azides in copper(I)-catalyzed Huisgen cycloaddition with alkynes has become a powerful click chemistry tool^7–10^, widely used in chemical biology^7^, drug discovery^8^, and also synthesis of chiral materials^9^. Current methods to synthesize chiral azides are mainly stereospecific transformations from chiral starting materials^11–13^; development of catalytic asymmetric approaches is important and has attracted great attention in recent years^14^. Several methods involving asymmetric nucleophilic or electrophilic azidation of prochiral compounds, with a key enantioselective C–N_3_ bond formation, have been developed^15–23^, but it is always difficult to control enantiofacial selectivity when handling the very small azido group. Catalytic asymmetric transformation of organic azides into chiral azides is an attractive alternative, which does not involve C–N_3_ bond construction^24–28^. Such transformations are comparatively less common, because keeping the energy-rich reactive azido group intact in asymmetric transformations is a challenge.

**Fig. 1:**
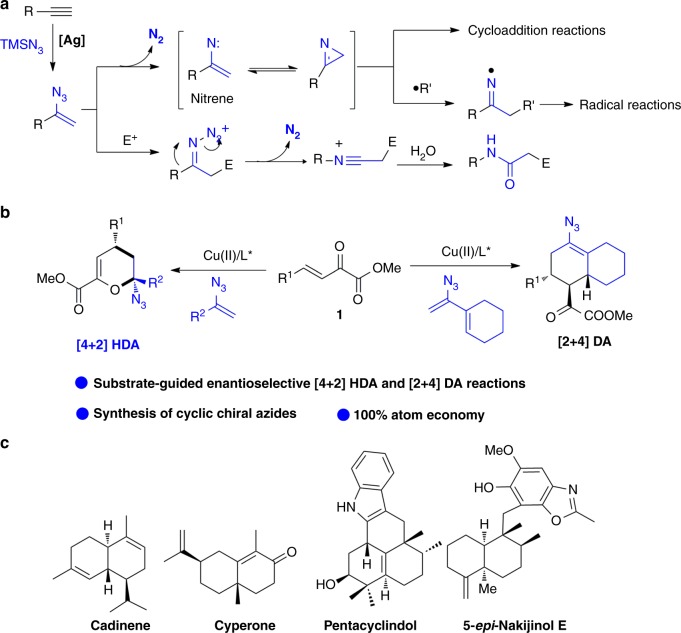
Reactivities of vinyl azides. **a** General reactivity of vinyl azides as CCN synthon driven by the release of N_2_. **b** Cycloaddition reactivity of vinyl azides with azide retention (this work). **c** Selected important natural sesquiterpenoids with decalin motif

Vinyl azides bearing azide and alkene functional groups conjugated together, could be easily obtained by the reaction of silver-catalzyed hydroazidation of terminal alkynes^29^, and have been recognized as versatile building blocks in organic synthesis^2,30–32^. Generally, vinyl azides undergo a fast release of two nitrogens to generate vinyl nitrene or strained 2*H*-aziridine intermediates, and subsequent cycloaddition or radical addition reactions lead to structurally diverse *N*-heterocycles^33–40^ (Fig. 1a). Vinyl azides can also react with electrophiles to form iminium ions, or undergo Schmidt-type rearrangements driven by loss of a dinitrogen unit to afford functionalized amides^41^. In this general denitrogenation reactivity mode, vinyl azides act as an important three-atom synthon in the construction of complex *N*-heterocyclic skeletons and have been extensively studied in recent years. Although keeping the conjugated azide moiety intact in organic transformations is a challenge^42^, such reactions can afford valuable organic azides which are highly desirable. For instance, in 2017, López et al. developed a copper(I)-catalyzed [3+2]-cycloaddition of vinyl azides, with unsaturated carbene precursors, to produce azidocyclopentenes^43^. However, this reaction requires 50 mol% of catalyst loading and only racemic reactions were reported. Consequently, the development of an efficient catalytic asymmetric cycloaddition reaction of vinyl azides to produce chiral azides is important. We report here the copper(II)-catalyzed asymmetric [4+2]-cycloaddition of vinyl azides with unsaturated ketone esters (**1**) to build chiral cyclic azides (Fig. 1b). More importantly, structurally diverse cyclic azides were obtained through a substrate reactivity-guided inverse-electron-demand hetero-Diels–Alder or Diels–Alder reactions. The Diels–Alder reaction produces a very important decalin motif, which is an omnipresent structural unit in a wide range of natural products, with various significant biological activities (Fig. 1c)^44–50^. Many important natural sesquiterpenoids and diterpenoids are isoprenoid decalins, for example, cadinene and cyperone are components of important essential oils from plants, and the natural indole sesquiterpenes pentacyclindole^47^ and 5-epi-nakijinol E^48^ have important antiproliferative and cytotoxic activities. Generally, such decalin scaffolds are built by a long linear stepwise construction of a chiral triene structure, followed by an intramolecular DA reaction^45,50^. The intermolecular catalytic asymmetric Diels–Alder reaction not reported to date is a more effective synthetic route to access this scaffold.

## Results

### Optimization of HDA reaction

To avoid formation of nitrene-type intermediates, we took advantage of the nucleophilic character of the C = C bond in vinyl azides, which enables it to react at low temperatures with highly electron-deficient unsaturated ketone esters (**1**) through an inverse-electron-demand hetero-Diels–Alder (HDA) reaction. We reasoned that when a chiral Lewis acid catalyst is applied to activate the unsaturated ketone ester (**1**) through chelation with the two carbonyl groups, chiral azides might be generated by a catalytic asymmetric reaction.

A vinyl azide (**2a**) and the ketone ester (**1a**) were selected as model substrates to test this reactivity in the presence of various Lewis acids (Table 1). The reaction proceeds smoothly in the presence of several metal Lewis acids, such as Sc(OTf)_3_, InCl_3_, and Cu(OTf)_2_, giving however the target azide-containing product (**3a**) in moderate-to-good yield with moderate diastereoselectivity (entries 1–3). The endo-adduct (**3a**) is the major product under these conditions. In view of the wide application of Cu(II)/bisoxazoline (BOX) catalysts in asymmetric catalysis, we further tried to realize the catalytic asymmetric reaction to produce chiral cyclic azides. Details are provided in Supplementary Table 1. A screening of various bisoxazoline ligands (entries 4–9) revealed that Ph-BOX (**L1**) or *t*-Bu-BOX (**L3**) gave very high enantioselectivity of 94% and 98% ee, respectively. Other ligands such as Bn-BOX (**L2**), *i*Pr-BOX (**L5**), and *i*PrPyBOX (**L6**) all gave inferior results. Further screening of various copper salts using ligand **L3** revealed that Cu(SbF_6_)_2_ gave an 86% yield and 96% ee, albeit with a low 60/40 diastereomeric ratio (dr) (entry 10). Recently, the Tang group have developed a successful sidearm modification strategy to elaborate the bisoxazoline ligands by introducing a pendant group at the bridging carbon^51–55^. We synthesized the sidearm-modified BOX ligands **L8** and **L9** and the Ph-BOX ligand bearing one pendant 4-*t*-BuC_6_H_4_ group (**L9**) gave the best results, affording the desired azide product in 89% yield with >99% ee and 80/20 dr (entry 14).

**Table 1:** Optimization of reaction conditions^a^


Entry	Metal	Ligand	endo/exo^b^	Yield (%)^c^	ee (%)^d^
1	Sc(OTf)_3_	–	–	30	–
2	InCl_3_	–	80/20	83	–
3	Cu(OTf)_2_	–	60/40	62	–
4	Cu(OTf)_2_	**L1**	50/50	50	94
5	Cu(OTf)_2_	**L2**	10/90	17	41
6	Cu(OTf)_2_	**L3**	60/40	25	98
7	Cu(OTf)_2_	**L4**	80/20	40	96
8	Cu(OTf)_2_	**L5**	–	0	–
9	Cu(OTf)_2_	**L6**	–	0	–
10	Cu(SbF_6_)_2_	**L3**	60/40	86	96
11	Cu(ClO_4_)_2_·6H_2_O	**L3**	–	0	–
12	Cu(SbF_6_)_2_	**L7**	60/40	83	37
13	Cu(SbF_6_)_2_	**L8**	60/40	99	96
14	Cu(SbF_6_)_2_	**L9**	80/20	89	>99

^a^Reaction conditions: a mixture of **1a** (0.2 mmol), **2a** (0.24 mmol), metal catalyst (10 mol%), and ligand (12 mol%), solvent (2 mL), 30 °C

^b^Determined by crude ^1^H NMR analysis

^c^Combined isolated yield

^d^Determined by HPLC using a chiral stationary phase

### Substrate scope of HDA reaction

After establishing the optimal conditions for the asymmetric cycloaddition reaction, the scope of substrates was further investigated. First, the reactivities of various vinyl azides were tested. As shown in Table 2, aromatic vinyl azides bearing various electron-withdrawing or electron-donating functional groups at the phenyl ring are well tolerated and produced the corresponding cyclic azides in good-to-excellent yields with high enantioselectivities (72–99% ee, entries 1–10). Strong electron-donating 4-methoxyl-substituted azides (**2e**) appear to be too reactive in this inverse-electron-demand HDA and give a lower ee of 72% (entry 5). The vinyl azides (**2k**) bearing a 2-naphthyl group and **2l** containing a thienyl group also reacted smoothly under the standard conditions, giving the corresponding azides **3k** and **3l** with 97% and 90% ee, respectively (entries 11, 12). However, aliphatic vinyl azides were found to be averse to this transformation. Then the scope of unsaturated ketoesters was also explored (Table 2). Various unsaturated ketoesters with both electron-withdrawing and electron-donating aromatic substituents at the *γ*-position all react smoothly with vinyl azide (**2a**), giving cyclic azides (**3m–3w)** in good yields with excellent enantioselectivities (>97% ee, entries 13–23). However, aliphatic benzyl group-substitued unsaturated ketoesters were not suitable substrates in this reaction, and no cycloaddition products were produced under standard conditions.

**Table 2:** Scope of Cu(II)-catalyzed hetero-Diels–Alder reactions^a^


Entry	1 (R^1^, R^2^)	2 (R^3^)	3	endo/exo	Yield (%)^b^	ee (%)^c^
1	**1a** (Ph, Me)	**2a** (Ph)	**3a**	80/20	89	**>**99
2	**1a**	**2b** (4-ClC_6_H_4_)	**3b**	80/20	82	97
3	**1a**	**2c** (4-FC_6_H_4_)	**3c**	78/22	84	96
4	**1a**	**2d** (4-BrC_6_H_4_)	**3d**	75/25	85	99
5	**1a**	**2e** (4-MeOC_6_H_4_)	**3e**	60/40	94	72
6	**1a**	**2f** (4-MeC_6_H_4_)	**3f**	90/10	74	95
7	**1a**	**2g** (4-^*t*^BuC_6_H_4_)	**3g**	93/7	66	98
8	**1a**	**2h** (4-PhC_6_H_4_)	**3h**	74/26	80	97
9	**1a**	**2i** (3-ClC_6_H_4_)	**3i**	84/16	74	98
10	**1a**	**2j** (3-MeC_6_H_4_)	**3j**	91/9	54	97.5
11	**1a**	**2k** (2-naphthyl)	**3k**	78/22	88	97
12	**1a**	**2l** (3-thienyl)	**3l**	72/28	81	90
13	**1b** (Ph, Et)	**2a** (Ph)	**3m**	86/14	82	99.5
14	**1c** (Ph, ^i^Pr)	**2a** (Ph)	**3n**	88/12	80	98
15	**1d** (4-MeC_6_H_4_, Bn)	**2a** (Ph)	**3o**	91/9	58	98
16	**1e** (4-ClC_6_H_4_, Me)	**2a** (Ph)	**3p**	90/10	78	97
17	**1f** (4-BrC_6_H_4_, Me)	**2a** (Ph)	**3q**	90/10	75	97
18	**1g** (4-FC_6_H_4_, Me)	**2a** (Ph)	**3r**	84/16	86	97
19	**1h** (4-MeC_6_H_4_, Me)	**2a** (Ph)	**3s**	89/11	94	99
20	**1i** (4-MeOC_6_H_4_, Me)	**2a** (Ph)	**3t**	93/7	58	98
21	**1j** (3-BrC_6_H_4_, Me)	**2a** (Ph)	**3u**	90/10	67	96.7
22	**1k** (2-naphthyl, Me)	**2a** (Ph)	**3v**	90/10	81	**>**99
23	**1l** (3-thienyl, Me)	**2a** (Ph)	**3w**	84/16	86	97

^a^Reaction conditions: **1** (0.2 mmol), **2** (0.24 mmol), Cu(SbF_6_)_2_ (10 mol%), and **L9** (12 mol%), 30 °C

^b^Isolated yield

^c^Determined by HPLC using a chiral stationary phase

### Asymmetric DA reaction to chiral decalin motif

To study the effect of conjugation in vinyl azides for the HDA reaction, we synthesized the *α,β*-unsaturated azide (**2m**) and exposed it to the standard conditions. Very interestingly, a distinct product (**4a**), was formed as the sole product, albeit with low (21%) ee through the [2+4] Diels–Alder (DA) cycloaddition (Table 3, entry 1). An inverse-electron-demand HDA between **1a** and **2m**, followed by a [3,3]-rearrangement could also produce the target product **4a**. If this is the case, the first HDA reaction generally will give high enantioselectivities and since the second step is a stereospecific percyclic reaction, the product should be produced with good enantioselectivity. The low enantioselectivity obtained under previous standard conditions indicates that this might not be a hetero-Diels–Alder/[3,3]-rearrangement but a direct Diels–Alder reaction process. The reactivity of the vinylic π bond might encourage it to act as a 4-π partner in a Diels–Alder reaction rather than the previous 2-π partner in the HDA reaction pathway.

**Table 3:** Optimization of the Cu(II)-catalyzed DA reactions^a^


Entry	Ligand	*T* (°C)	Yield (%)^b^	ee (%)^c^
1	**L9**	30	84	21
2	**L8**	30	64	73
3	**L3**	30	75	80
4	**L3**	−20	78	87
5	**L10**	−20	84	87
6	**L11**	−20	81	84
7	**L12**	−20	80	93

^a^Reaction conditions: a mixture of **1a** (0.2 mmol), **2m** (0.24 mmol), Cu(SbF_6_)_2_ (10 mol%), and ligand (12 mol%)

^b^Isolated yield

^c^Determined by HPLC using a chiral stationary phase

We next aimed to get higher enantioselectivities in the synthesis of decalins and the obtained results are summarized in Table 3. *t-*Bu-BOX ligand with two sidearm *t-*BuC_6_H_4_CH_2_ groups (**L8**) enhanced the ee to 73% (Table 3, entry 2). *t-*Bu-BOX with *gem* dimethyl groups at the bridging carbon (**L3**) gave the desired product with 75% yield and 80% ee (entry 3). The ee was further improved to 87%, while maintaining good yield (78%), by lowering the temperature to −20 °C (entry 4). Finally, we employed *t-*Bu-BOX ligands with a variable bite angle (**L10**, **L11**, and **L12**) and found the optimum conditions with a cyclopentyl-substituted *t-*Bu-BOX (**L12**), which gave the azidodecalin (**4a**) in 80% yield with 93% ee (entry 7).

Using the optimum conditions, the substrate scope of the Diels–Alder reaction with respect to unsaturated ketoesters was evaluated (Table 4). Variously substituted unsaturated ketoesters produced the corresponding octahydronaphthalenes (**4b**–**4g**) in good yields (61–81%) and with good-to-excellent enantioselectivity (82–93%). Notably, all these reactions gave very good diastereoselectivity and no HDA product was observed. A five-membered conjugated azido diene **2n** was also synthesized and subjected to the Diels–Alder reaction, with the unsaturated ketoester **1a** at −30 °C; the desired hexahydro-indene azide **4h** was obtained with 87% yields, with 88% ee as a single diastereomer.

**Table 4:** Substrate scope of Cu(II)-catalyzed DA reactions^a^

^a^Reaction conditions: **1** (0.2 mmol), **2m or 2n** (0.24 mmol), Cu(SbF_6_)_2_ (10 mol%), and **L12** (12 mol%) at −20 °C

^b^Isolated yield

^c^Determined by HPLC using a chiral stationary phase

^d^At −10 °C

^e^At −30 °C

### Synthetic applications and absolute configuration determination

**Fig. 2:**
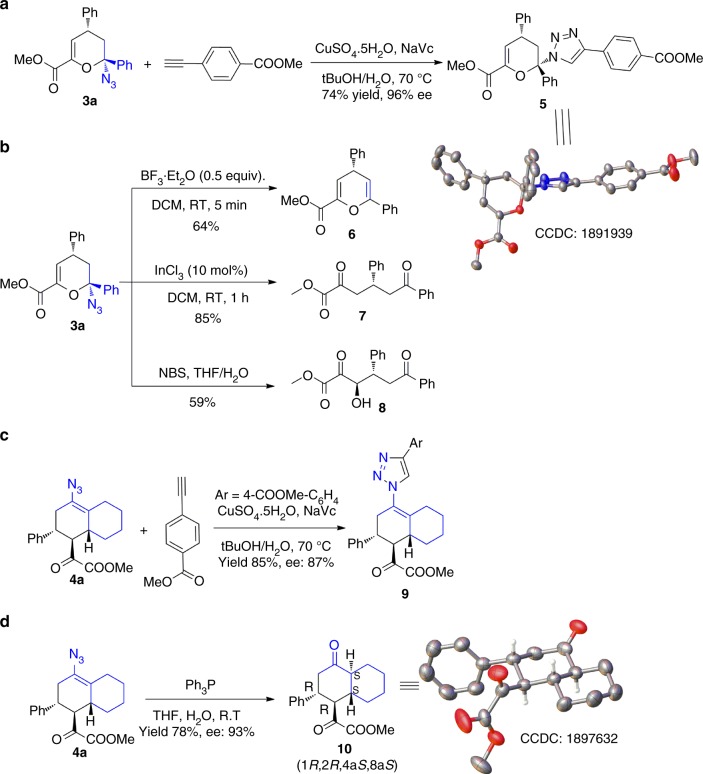
Further synthetic applications of chiral cyclic azides and absolute configuration determination. **a** Click reaction of chiral cyclic azide **3a** and the crystal structure of triazole **5**. **b** Transformations of **3a** into **6**, **7**, and **8**. **c** Click reaction of chiral bicyclic azide **4a**. **d** Reduction of **4a** into *trans*-decalone (**10**)

To demonstrate the synthetic utility of the developed method, **3a** was synthesized on a gram scale and several transformations were carried out. As shown in Fig. 2, a click reaction on **3a**, produced the triazole (**5**) with 74% yield in good enatioselectivity (96% ee). X-ray diffraction of a single crystal of this compound confirmed its structure, and the absolute configuration of compound **3a** was established as (2*S*, 4*R*). In the presence of a Lewis acid (BF_3_·Et_2_O), **3a** afforded chromene **6** in 64% yield, by the expulsion of hydrazoic acid. Treatment of **3a** with InCl_3_ afforded a ring-opening product, a 1,5-diketone (**7**) in 85% yield. Likewise, a linear hydroxyl-functionalized tricarbonyl compound (**8**) was synthesized in 59% yield, using N-bromosuccinimide (NBS) and H_2_O.

Further, synthetic transformations of the azido octahydro naphthalene (**4a**) were also performed. The triazole **9** was easily obtained in good yields and with retained enantioselectivities under traditional click conditions. Finally, under Staudinger reaction conditions, the decalin (**4a**) produced the *trans*-decalone (**10**), a ubiquitous structural unit in various natural products, in 78% yield with 93% ee. The absolute configuration of *trans*-decalone (**10**) was established as (*1R, 2R, 4aS, 8aS*) by X-ray crystallography (Fig. 2).

## Discussion

In summary, we have examined the reactivity of vinyl azides in asymmetric cycloaddition reactions for the synthesis of diverse chiral cyclic azides. α-Aryl-substituted vinyl azides react with unsaturated ketoesters through inverse-electron-demand hetero-Diels–Alder reaction, but cyclohexenyl azide reacts through diastereoselective and enantioselective Diels–Alder reactions. The prominent features of these reactions include 100% atom economy, ambient reaction conditions, a very broad substrate scope, excellent enantioselectivities, and useful product applications. Further application of this strategy is in progress in our laboratory.

## Methods

### Materials

All the solvents were treated according to standard methods. Unless otherwise noted, materials were purchased from commercial suppliers and used directly without further purification. Flash-column chromatography was performed using 100–200-mesh silica gel. All air-sensitive and moisture-sensitive reactions were performed under an atmosphere of N_2_ with standard Schlenk techniques. For ^1^H, ^13^C NMR and high-resolution mass spectrometry of compounds, the synthetic procedures, and details of the mechanism study, see Supplementary Methods.

## Supplementary information

Supplementary Information

## Data Availability

The X-ray crystallographic structures for compounds **5**, **10**, reported in this article, have been deposited at the Cambridge Crystallographic Data Centre (CCDC), with the accession codes CCDC 1891939 and 1897632 (http://www.ccdc.cam.ac.uk/data_request/cif). The authors declare that all other relevant data supporting the findings of this study are available within the article and its Supplementary Information files.
