# Supplementary material for: Divergent synthesis of chiral cyclic azides via asymmetric cycloaddition reactions of vinyl azides
Source: Nat Commun. 2019 Jul 18;10:3158. doi: 10.1038/s41467-019-11134-8 (PMC6639305; doi:10.1038/s41467-019-11134-8)
Supplement: Supplementary file 1 — Supplementary Information [file 41467_2019_11134_MOESM1_ESM.pdf]

SUPPLEMENTARY INFORMATION

Divergent Synthesis of Chiral Cyclic Azides via Asymmetric Cycloaddition Reactions of Vinyl Azides

Nuligonda et al.

Supplementary Methods

Reagents: All reagents and solvents were purchased from commercial sources and used without purification.

β, ¥-unsaturated α ketoesters were synthesized by reported route,[1] vinyl azides were synthesized by reported methods.[2,3]

Instruments: NMR spectra were recorded with a 500 MHz spectrometer for ^1H NMR, 125 MHz for ^{13}C NMR spectroscopy. Chemical shifts are reported relative to the residual signals of tetramethylsilane in $CDCl_3$ ^1H and ^{13}C NMR spectroscopy. Multiplicities are reported as follows: singlet (s), doublet (d), doublet of doublets (dd), doublet of triplets (dt), triplet (t), quartet (q), multiplet (m). HRMS were measured on the Q-TOF6510 instruments. Column chromatography was performed with silica gel (100–200 mesh) as the stationary phase. All reactions were monitored by using TLC. The purity and characterization of compounds were further established by using HRMS.

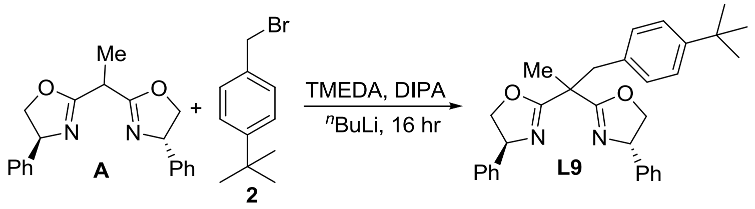

Supplementary Figure 1. Synthesis of Ligand **L9**

Following a reported procedure,[4] to the solution of (4S,4'S)-2,2'-(ethane-1,1-diyl)bis(4-phenyl-4,5-dihydrooxazole) **A** (179 mg, 0.56 mmol, 1.0 eq) in THF (10 mL) in a 50 mL schlenk tube, was added TMEDA (170 µL, 1.12 mmol, 2.0 eq) and DIPA (80 µL, 0.56 mmol, 1.0 eq). The solution was cooled to -78 ℃ and *n*-BuLi (0.45 mL, 2.5 M in hexane, 1.12 mmol, 2.0 equiv) was added. The reaction mixture was warmed to -20 ℃ and stirred at that temperature for 30 minutes. The solution was cooled back to -78 ℃ and bromo compound **2** (254 mg, 1.12 mmol, 2.0 equiv) was added to the reaction mixture in 10 minutes. After the addition, the cold bath was removed and the reaction mixture was allowed to stir at room temperature for an additional 16 h. The reaction mixture was quenched with the sat. aq. NH_4Cl (5.0 mL) and diluted with water (4 mL) to dissolve the resulting salts. The mixture was extracted with diethylether (3 X 20 mL). The combined organic layers were washed with brine (20 mL), dried over $MgSO_4$, and concentrated. The resulting oily residue was purified by column chromatography using 1:30 to 2:30

acetone:petroleum ether as mobile phase to afford (**L9**) as a light brown color solid (166.8 mg, 64% yield); ^{1}H NMR (500 MHz, $CDCl_3$) δ 7.34-7.24 (m, 10H), 7.15 (d, J = 8.2 Hz, 2H), 7.12-7.10 (m, 2H), 5.29-5.18 (m, 2H), 4.73-4.69 (m, 2H), 4.19 (t, J = 8.0 Hz, 1H), 4.13 (t, J = 8.5 Hz, 1H), 3.41 (q, J = 13.5 Hz, 1H), 1.60 (s, 3H), 1.32 (s, 9H); ^{13}C NMR (125 MHz, $CDCl_3$) δ 169.3, 169.2, 149.6, 142.3, 142.0, 133.3, 130.3, 130.2, 128.6, 128.5, 127.9, 127.4, 126.8, 126.7, 125.0, 75.3, 75.2, 69.7, 69.5, 43.8, 41.5, 34.4, 31.4, 21.5; HRMS exact mass calcd for $C_{31}H_{35}N_2O_2$ $[M + H]^+$ 467.2699, found 467.2709. $[\alpha]^{29}_D$ = -92.6 °(c 0.65, $CHCl_3$).

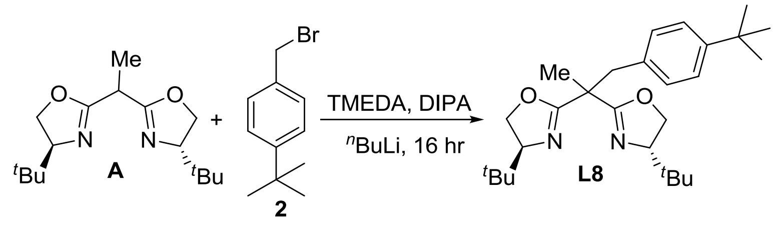

Supplementary Figure 2. Synthesis of Ligand **L8**

Following a reported procedure,[4] to the solution of (4S,4'S)-2,2'-(ethane-1,1-diyl)bis(4-*tert*-butyl-4,5-dihydrooxazole) **A** (156 mg, 0.56 mmol, 1.0 eq) in THF (10 mL) in a 50 mL schlenk tube, was added TMEDA (170 µL, 1.12 mmol, 2.0 eq) and DIPA (80 µL, 0.56 mmol, 1.0 eq). The solution was cooled to -78 ℃ and *n*-BuLi (0.45 mL, 2.5 M in hexane, 1.12 mmol, 2.0 equiv) was added. The reaction mixture was warmed to -20 ℃ and stirred at that temperature for 30 minutes. The solution was cooled back to -78 ℃ and bromo compound **2** (254 mg, 1.12 mmol, 2.0 equiv) was added in 10 minutes. After the addition, the cold bath was removed and the reaction mixture was allowed to stir at room temperature for an additional 16 h. The reaction mixture was quenched with the sat. aq. NH_4Cl (5.0 mL) and diluted with water (4 mL) to dissolve the resulting salts. The mixture was extracted with diethylether (3 X 20 mL). The combined organic layers were washed with brine (20 mL), dried over $MgSO_4$, and concentrated. The resulting oily residue was purified by column chromatography using 1:30 to 2:30 acetone:petroleum ether as mobile phase to afford (**L8**) as a white solid (147.1 mg, 62% yield); ^{1}H NMR (500 MHz, $CDCl_3$) δ 7.23 (d, J = 8.2 Hz, 2H), 7.09 (d, J = 8.2 Hz, 2H), 4.21-4.11 (m, 3H), 4.02 (t, J = 7.8 Hz, 1H), 3.87-3.80 (m, 2H), 3.32 (d, J = 13.6 Hz, 1H), 3.16 (t, J = 13.6 Hz, 1H), 1.44 (s, 3H), 1.27 (s, 9H), 0.87 (s, 9H), 0.80 (s, 9H); ^{13}C NMR (125 MHz, $CDCl_3$) δ 167.6, 167.4, 149.2, 133.6, 130.2, 124.8, 75.6, 75.3, 68.7, 68.7, 43.4, 41.4, 34.3, 34.0, 33.7, 31.3, 25.8, 25.7, 21.1; HRMS exact mass calcd for $C_{27}H_{43}N_2O_2$ $[M + H]^+$ 427.3325, found 427.3324. $[\alpha]^{29}_D$ = -92.6 °(c 0.43, $CHCl_3$).

Supplementary table 1: Optimization for the HDA reaction[a]

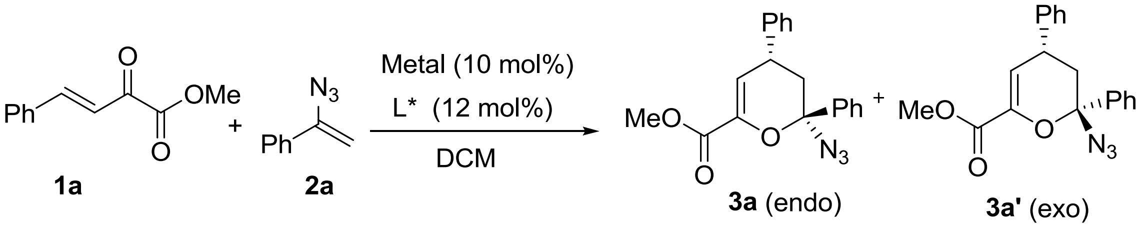

1a **2a** **3a** (endo) **3a'** (exo)

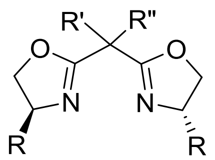

L1, R = Ph, R' = R" = Me
L2, R = Bn, R' = R" = Me
L3, R = ^{t}Bu, R' = R" = Me
L4, R = Ph, R' = R" = 4-^{t}Bu$C_6H_4CH_2$
L5, R = ^{i}Pr, R' = R" = Bn
L8, R = ^{t}Bu, R' = Me, R" = 4-^{t}BuC6H4CH2
L9, R = Ph, R' = Me, R" = 4-^{t}BuC6H4CH2
L13, R = ^{i}Pr, R' = Me, R" = Bn
L14, R = ^{t}Bu, R' = R" = Bn
L15, R = ^{i}Pr, R' = R" = 2, 4-di-^{t}Bu$C_6H_4CH_2$

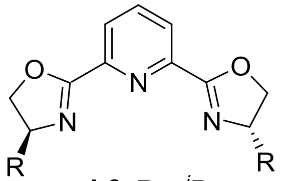

L6, R = ^{i}Pr
L7, R = Ph

Entry	Metal	Ligand	endo/exo[b]	Yield (%)[c]	ee (%)[d]
1	$Cu(OTf)_2$	**L1**	50/50	50	94
2	$Cu(OTf)_2$	**L2**	10/90	17	41
3	$Cu(OTf)_2$	**L3**	60/40	25	98
4	$Cu(OTf)_2$	**L4**	80/20	40	96
5	$Cu(OTf)_2$	**L5**	/	0	--
6	$Cu(OTf)_2$	**L6**	/	0	--
7	$Cu(OTf)_2$	**L7**	/	0	--
8	$Cu(OTf)_2$	**L13**	40/60	50	7
9	$Cu(OTf)_2$	**L14**	/	0	--
10	$Cu(OTf)_2$	**L15**	/	0	--
11	$Cu(SbF_6)_2$	**L3**	60/40	86	96
12	$Cu(ClO_4)_2.6H_2O$	**L3**	/	0	/
13	$Cu(SbF_6)_2$	**L4**	60/40	92	96

14	$Cu(SbF_6)_2$	**L7**	60/40	83	37
15	$Cu(SbF_6)_2$	**L8**	60/40	99	89
16	$Cu(SbF_6)_2$	**L9**	80/20	89	>99

[a]**Reaction conditions**: Lewis acid (10 mol%), Ligand (12 mol%), 4A^{o} MS (100 mg) in 1 mL DCM were stirred at 30 ^{o}C for 3h, then mixture of **1a** (0.2 mmol) and **2a** (0.24 mmol) in 1 mL DCM were added to the reaction mixture. [b]Determined by crude ^{1}H NMR analysis. [c]Combined isolated yield. [d]Determined by HPLC using a chiral stationary phase.

Preparation and Characterization of Compound 3

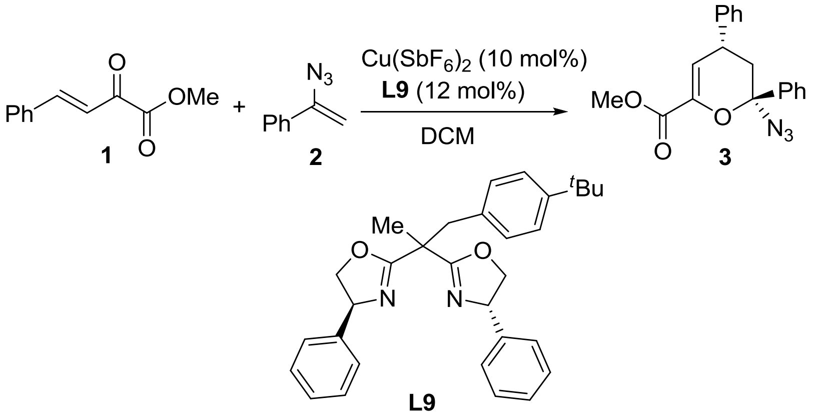

Supplementary Figure 3. Procedures for the preparation of compounds **3a-3w**

General Procedure for the *Cu(II)-BOX* catalyzed enantioselecetive [4+2]-cycloaddition of keto esters 1 with azides 2 taking synthesis of 3a as an example:

A mixture of $CuBr_2$ (4.4 mg, 0.02 mmol, 0.1 eq), $AgSbF_6$ (13.7 mg, 0.04 mmol, 0.2 eq), ligand (**L9**, 11.2 mg, 0.024 mmol, 0.12 eq) and 100 mg 4A^{o} MS in DCM (1 mL) was stirred at 30 ^{o}C for 3 h under nitrogen. Then, a mixture of **1a** (38.0 mg, 0.2 mmol, 1 eq) and **2a** (34.8 mg, 0.24 mmol, 1.2 eq) in DCM (1 mL) were added to the catalyst solution via a syringe. The resulting suspension was allowed to stir at 30 ^{o}C and monitored by TLC, until the complete consumption of **1** (usually 48 to 60 hours). Then, the mixture was filtered through a pad of celite and washed with DCM. The filterate was concentrated under reduced pressure and the residue was purified by column chromatography over silicagel using (ethyl acetate/petroleum ether, 1/50) to afford **3a** (59.6 mg, 89% yield) as a Colorless oil.

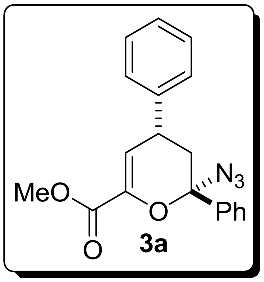

Colorless oil; (59.6 mg, 89% yield); ^{1}H NMR (500 MHz, $CDCl_3$) δ 7.55-7.50 (m, 2H), 7.50-7.40 (m, 3H), 7.36-7.30 (m, 2H), 7.27-7.24 (m, 1H), 7.21-7.15 (m, 2H), 6.20 (d, J = 3.0 Hz, 1H), 3.89 (s, 3H), 3.31-3.24 (m, 1H), 2.71-2.64 (m, 1H), 2.21 (dd, J = 14.0, 10.0 Hz, 1H); ^{13}C NMR (125 MHz, $CDCl_3$) δ 162.5, 142.6, 141.7, 138.1, 129.3, 129.0, 128.7, 127.9, 127.7, 125.2, 115.0, 94.4, 52.4, 39.7, 36.6; IR (neat): 2949, 2107, 1731, 1647, 1448, 1254, 1113, 1042, 759, 698 cm^{-1};HRMS exact mass calcd for $C_{19}H_{17}N_3NaO_3$ [M + Na]$^+$ 358.1168, found 358.1171. $[\alpha]^{18}_{D}$ = 11.5 ° (c 0.51, $CHCl_3$); >99% ee; Chiral HPLC analysis of the product: Daicel Chiralcel OD-H 250X4.6 mm 5u column; hexane/2-propanol = 99/1, detected at 254 nm, Flow rate = 1 mL/min, Retention times: 14.1 min (minor), 18.3 min (major).

Supplementary Figure 4. HPLC spectra of 3a

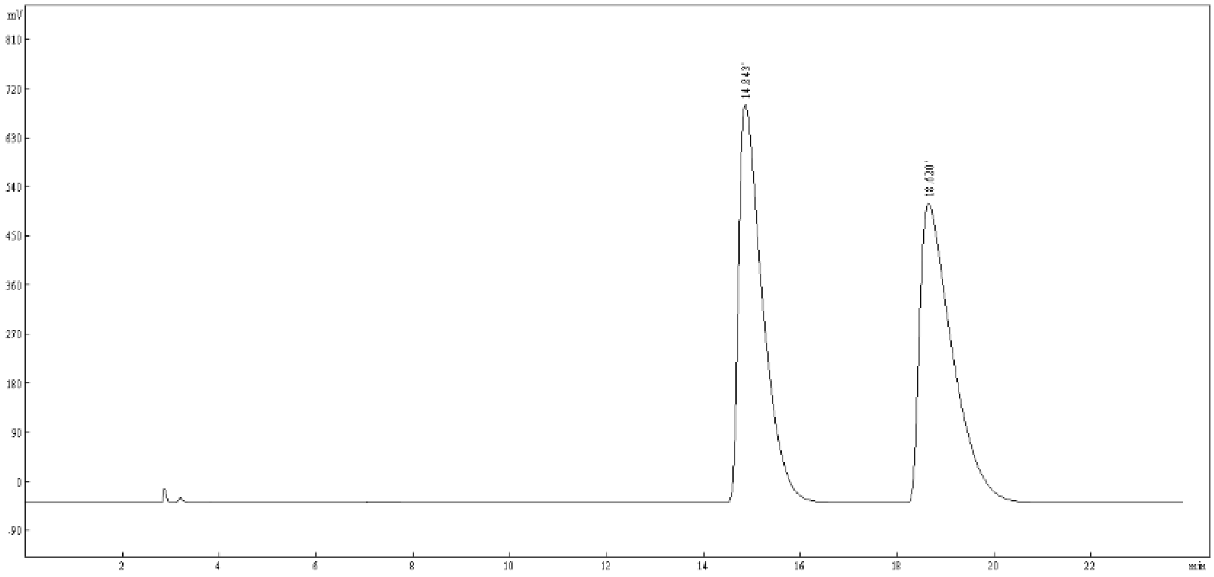

Peak#	Ret.Time	Area	Area %
1	14.843	25026654	49.62
2	18.620	25412685	50.38
Total		50439339	100

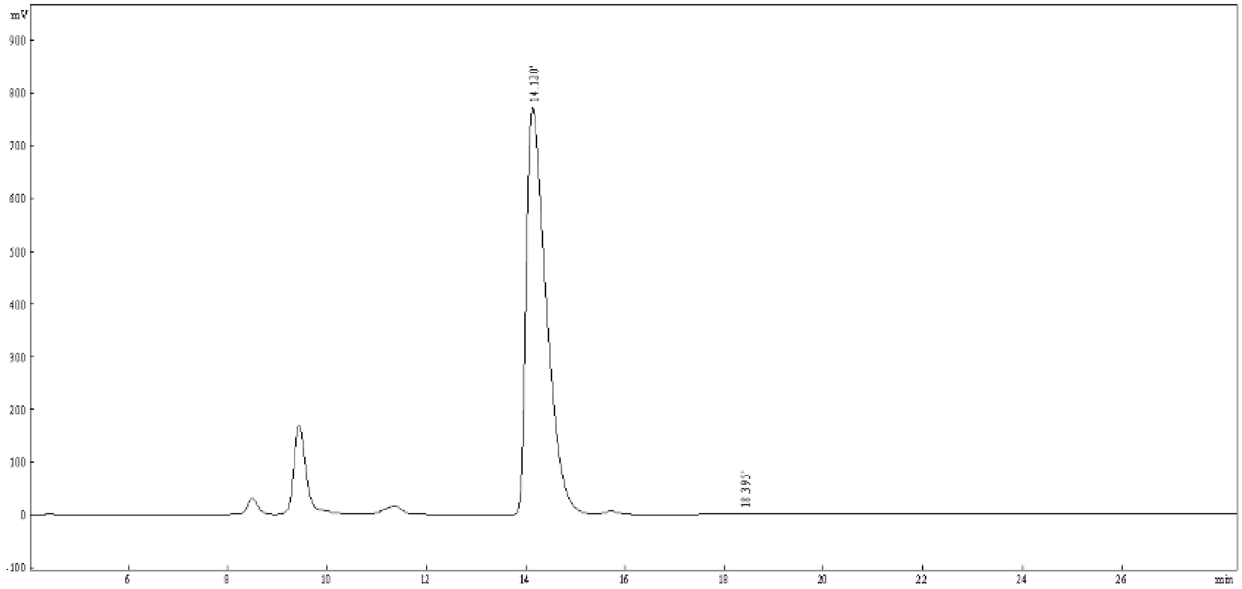

Peak#	Ret.Time	Area	Area %
1	14.120	21461562	100
2	18.395	132	0.00
Total		21461694	100

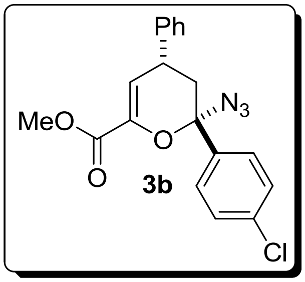

3b

Light yellow oil; (60.5 mg, 82% yield); ^{1}H NMR (500 MHz, $CDCl_3$) δ 7.50-7.39 (m, 4H), 7.36-7.31 (m, 2H), 7.29-7.27 (m, 1H), 7.22-7.14 (m, 2H), 6.22 (dd, J = 3.6 Hz, 1H), 3.88 (s, 3H), 3.30-3.27 (m, 1H), 2.67-2.57 (m, 1H), 2.21 (dd, J = 13.9, 9.5 Hz, 1H); ^{13}C NMR (125 MHz, $CDCl_3$) δ 162.3, 142.5, 141.5, 136.8, 135.3, 129.3, 128.8, 127.5, 127.2, 126.8, 115.1, 93.8, 52.5, 39.7, 36.5; IR (neat): 2946, 2110, 1736, 1261, 1093, 993, 760, 696 cm^{-1}; HRMS exact mass calcd for $C_{19}H_{16}ClN_3NaO_3$ $[M + Na]^+$ 392.0778, found 392.0774. $[\alpha]^{29}_{D}$ = 42.0 ° (c 0.95, $CHCl_3$); 97% ee; Chiral HPLC analysis of the product: Phenomenex 00G-4457-E0 250X4.6 mm 5u column;

hexane/2-propanol = 99.5/0.5, detected at 254 nm, Flow rate = 1 mL/min, Retention times: 15.5 min (major), 19.5 min (minor).

Supplementary Figure 5. HPLC spectra of 3b

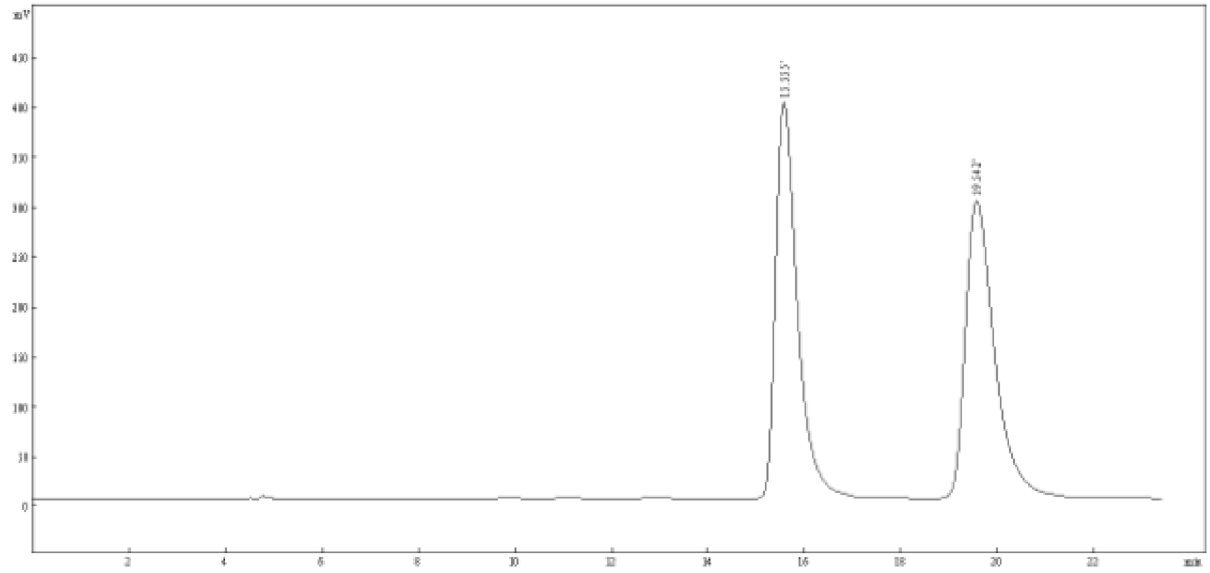

Peak#	Ret.Time	Area	Area %
1	15.555	12466282	49.79
2	19.542	12571870	50.21
Total		25038152	100

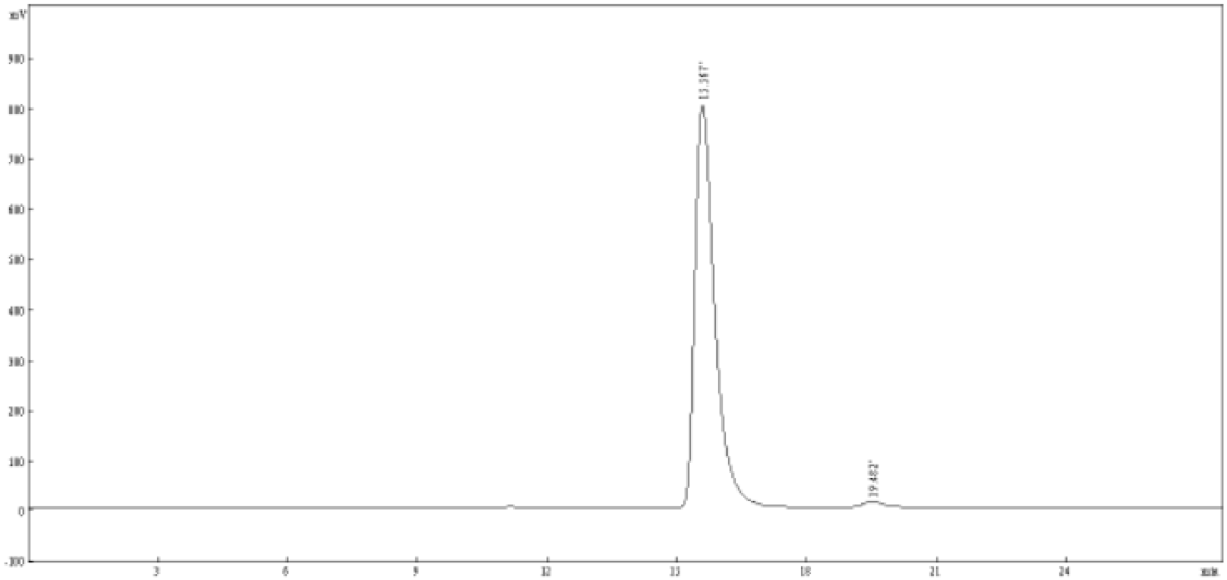

Peak#	Ret.Time	Area	Area %
1	15.567	25616662	98.47
2	19.482	396523	1.52
Total		26013185	100

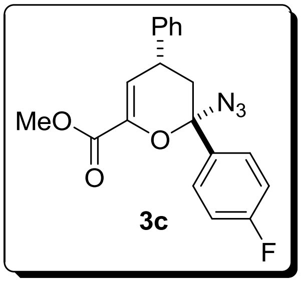

Light yellow oil; (59.3 mg, 84% yield); ^{1}H NMR (500 MHz, $CDCl_3$) δ 7.51-7.49 (m, 2H), 7.34-7.31 (m, 2H), 7.28-7.25 (m, 1H), 7.20-7.12 (m, 4H), 6.22 (dd, J = 3.0, 0.8 Hz, 1H), 3.89 (s, 3H), 3.30-3.26 (m, 1H), 2.63-2.59 (m, 1H), 2.22 (dd, J = 13.9, 9.6 Hz, 1H); ^{13}C NMR (125 MHz, $CDCl_3$) δ 163.0 (d, J = 250 Hz), 162.4, 142.0 (d, J = 112 Hz), 134.1 (d, J = 2 Hz), 128.8, 127.5, 127.3, 127.3 (d, J = 25 Hz), 116.0 (d, J = 25 Hz), 115.0, 93.8, 52.5, 39.8, 36.5; IR (neat): 2949, 2110, 1727, 1506, 1258, 1235, 1110, 1040, 757, 699 cm^{-1}; HRMS exact mass calcd for $C_{19}H_{16}FN_3NaO_3$ $[M + Na]^+$ 376.3368, found 376.1077. $[\alpha]^{29}_{D}$ = -5.4 ° (c 0.83, $CHCl_3$); 96% ee; Chiral HPLC analysis of the product: Phenomenex 00G-4457-E0 250X4.6 mm 5u column; hexane/2-propanol = 99.5/0.5, detected at 254 nm, Flow rate = 1 mL/min, Retention times: 15.2 min (major), 19.0 min (minor).

Supplementary Figure 6. HPLC spectra of 3c

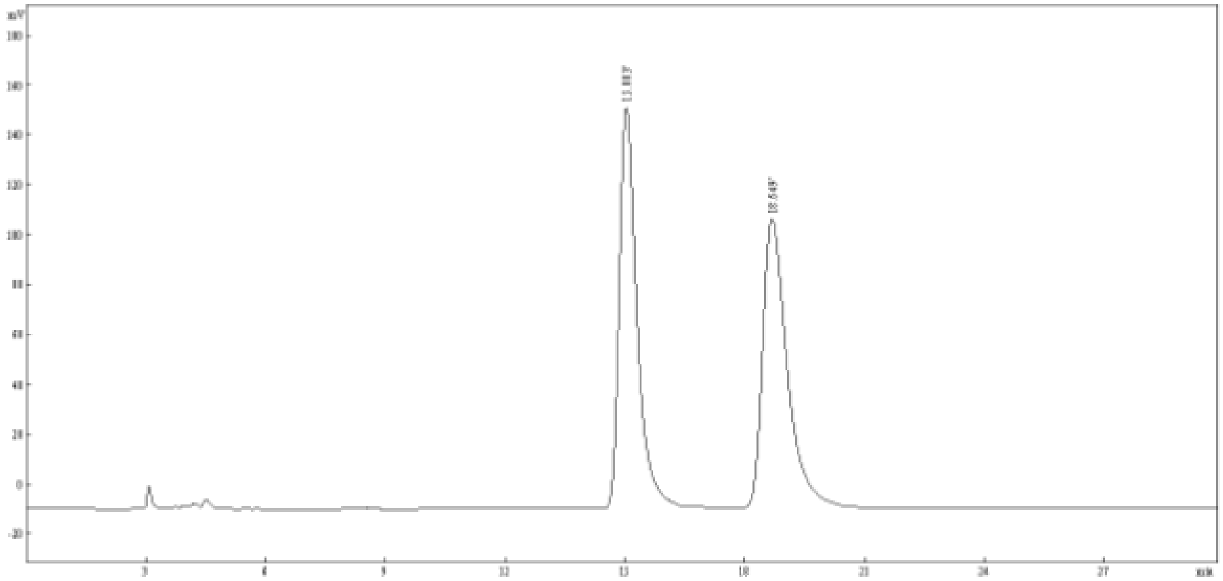

Peak#	Ret.Time	Area	Area %
1	15.003	4959682	49.99
2	18.649	4961335	50.01
Total		40724820	100

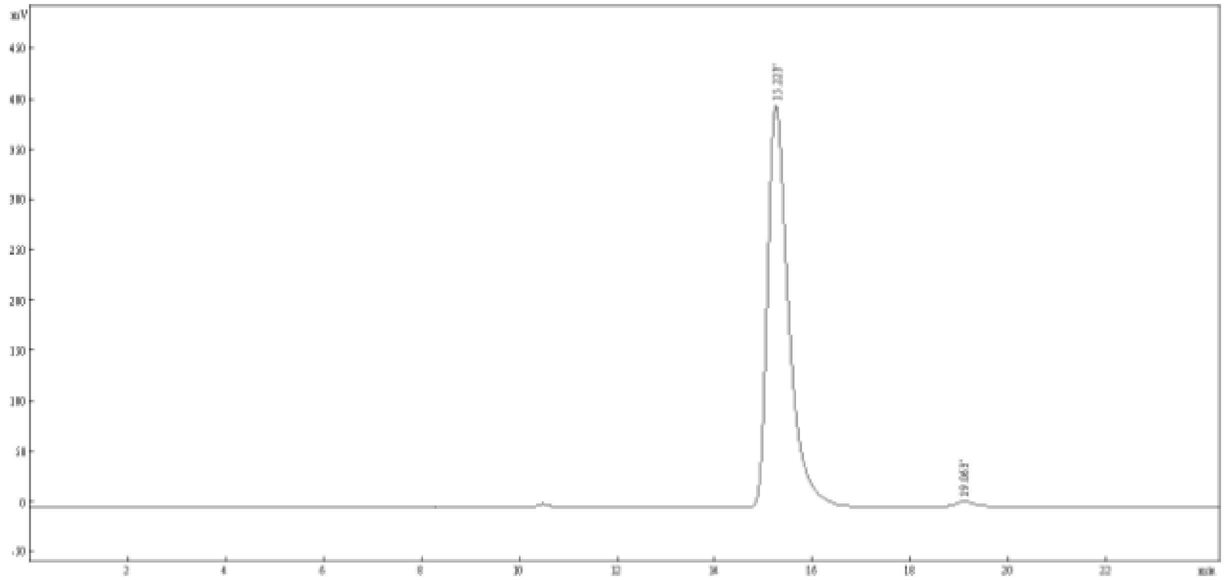

Peak#	Ret.Time	Area	Area %

1	15.223	11995022	98.17
2	19.063	223789	1.831
Total		12218811	100

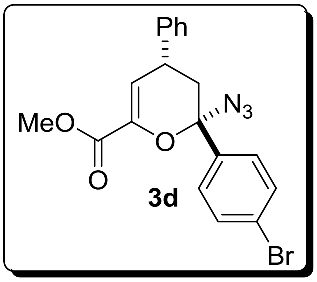

Light yellow oil; (70.2 mg, 85% yield); ^{1}H NMR (500 MHz, $CDCl_3$) δ 7.60-7.57 (m, 2H), 7.40-7.37 (m, 2H), 7.34-7.31 (m, 2H), 7.28-7.25 (m, 1H), 7.19-7.18 (m, 2H), 6.23-6.22 (m, 1H), 3.87 (s, 3H), 3.30-3.26 (m, 1H), 2.21 (dd, J = 13.9, 9.6 Hz, 1H); ^{13}C NMR (125 MHz, $CDCl_3$) δ 162.3, 142.5, 141.5, 137.4, 132.2, 128.8, 127.5, 127.2, 127.1, 123.5, 115.1, 93.8, 52.5, 39.6, 36.5; IR (neat): 2934, 2110. 1735, 1653, 1439, 1260, 1111, 999, 762, 697 cm^{-1}; HRMS exact mass calcd for $C_{21}H_{14}BrO$ [M - H]$^+$ 436.0273, found 436.0270. $[\alpha]^{29}_D$ = -65.1 ° (c 1.01, $CHCl_3$); 97% ee; Chiral HPLC analysis of the product: Phenomenex 00G-4457-E0 250X4.6 mm 5u column; hexane/2-propanol = 99.2/0.8, detected at 254 nm, Flow rate = 1 mL/min, Retention times: 12.1 min (major), 14.5 min (minor).

Supplementary Figure 7. HPLC spectra of 3d

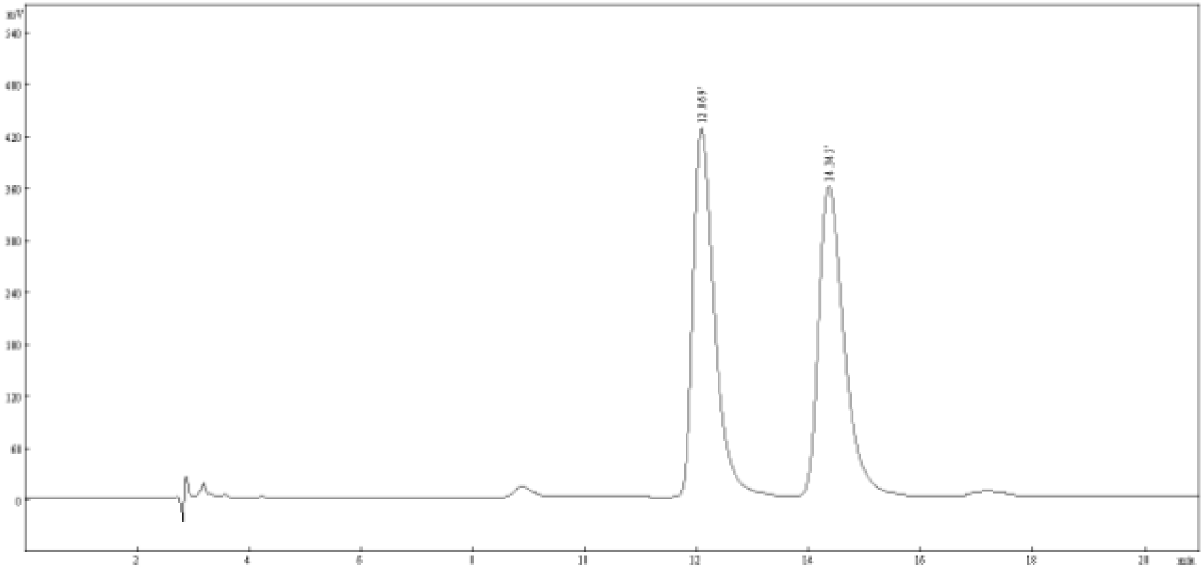

Peak#	Ret.Time	Area	Area %
1	12.069	11247628	49.97
2	14.345	11259798	50.03
Total		22507426	100

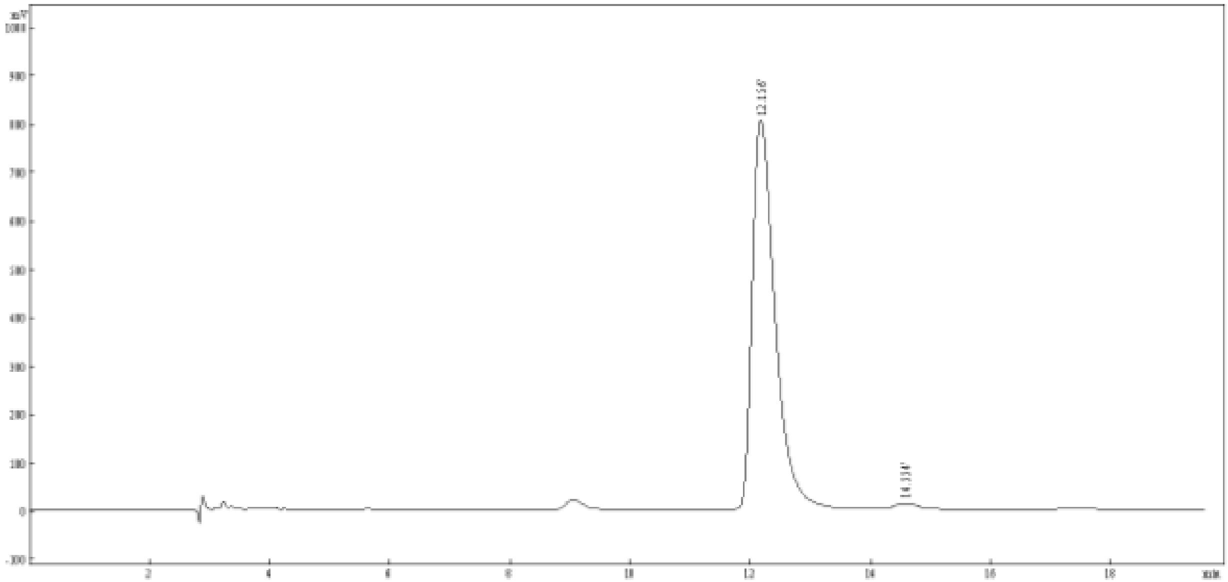

Peak#	Ret.Time	Area	Area %
1	12.156	21150438	98.7
2	14.544	278323	1.299
Total		21428761	100

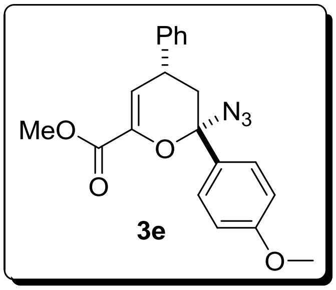

Light yellow soild; (68.6 mg, 94% yield); ^{1}H NMR (500 MHz, $CDCl_3$) δ 7.45-7.42 (m, 2H), 7.33-7.18 (m, 5H), 6.98-6.95 (m, 2H), 6.18 (dd, J = 2.7, 1.1 Hz, 1H), 3.89 (s, 3H), 3.88 (s, 3H),

3.84 (s, 3H), 3.30-3.26 (m, 1H), 2.66-2.62 (m, 1H), 2.18 (dd, J = 13.8, 10.1 Hz, 1H); ^{13}C NMR (125 MHz, $CDCl_3$) δ 162.7, 160.1, 142.6, 141.8, 130.0, 128.7, 127.1, 126.7, 115.0, 114.3, 94.5, 55.3, 52.4, 39.7, 36.8; IR (neat): 2925, 2849, 2107, 1735, 1525, 1260, 1034, 769, 703 cm^{-1}; HRMS exact mass calcd for $C_{20}H_{19}N_3NaO_4$ [M + Na]$^+$ 388.1273, found 388.1282. $[\alpha]^{28}_D$ = 21.1 ° (c 0.40, $CHCl_3$); 74% ee; Chiral HPLC analysis of the product: Phenomenex 00G-4457-E0 250X4.6 mm 5u column; hexane/2-propanol = 99.4/0.6, detected at 254 nm, Flow rate = 1 mL/min, Retention times: 27.8 min (major), 32.0 min (minor).

Supplementary Figure 8. HPLC spectra of 3e

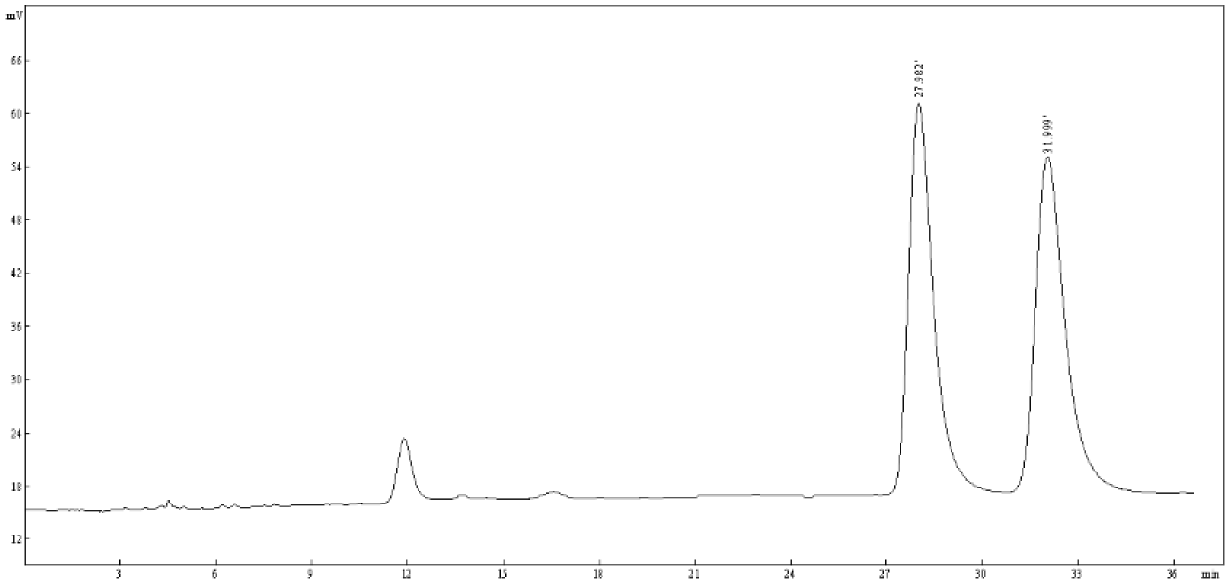

Peak#	Ret.Time	Area	Area %
1	27.982	2421384	49.42
2	31.999	2478446	50.58
Total		4899830	100

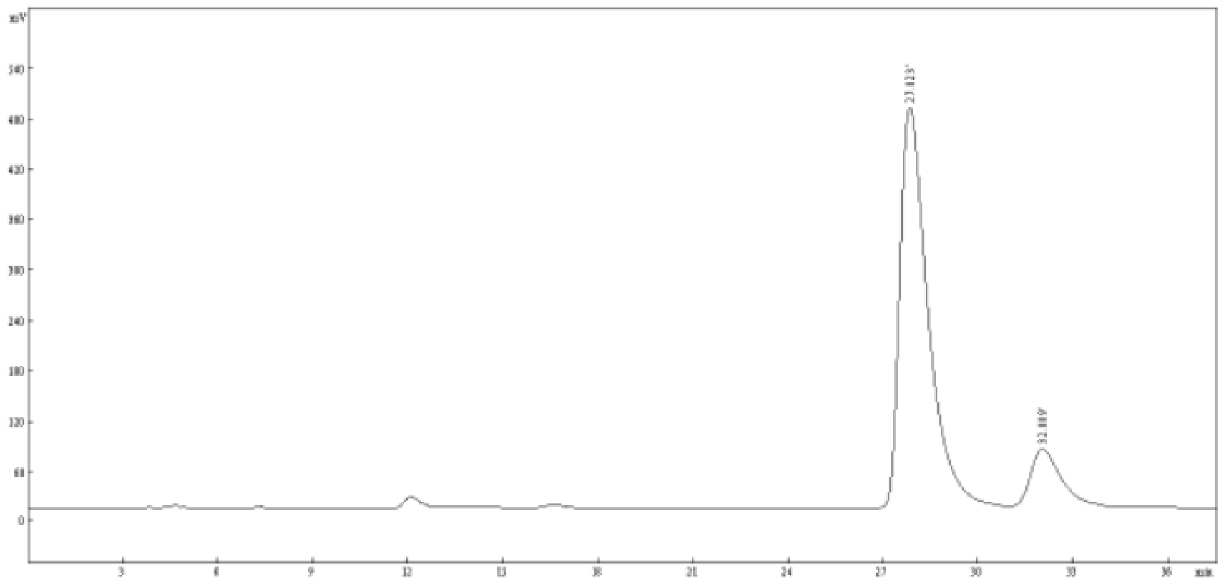

Peak#	Ret.Time	Area	Area %
1	27.823	29529648	86.89
2	32.009	4454318	13.11
Total		33983966	100

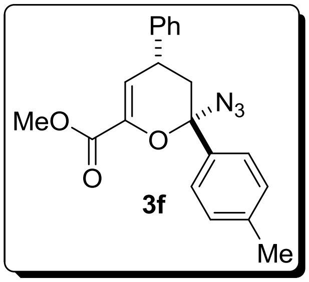

Colorless oil; (51.6 mg, 74% yield); ^{1}H NMR (500 MHz, $CDCl_3$) δ 7.39 (d, J = 8.5, Hz, 2H), 7.33-7.30 (m, 2H), 7.26-7.24 (m, 3H), 7.19-7.17 (m, 2H), 6.17 (dd, J = 2.8, 1.1 Hz, 1H), 3.88 (s, 3H), 3.30-3.26 (m, 1H), 2.68-2.63 (m, 1H), 2.38 (s, 3H), 2.18 (dd, J = 13.8, 10.2 Hz, 1H); ^{13}C NMR (125 MHz, $CDCl_3$) δ 162.5, 142.6, 141.8, 139.2, 135.0, 129.7, 128.7, 127.5, 125.2, 115.0, 94.5, 52.4, 39.7, 36.7, 31.2; IR (neat): 2943, 2107, 1729, 1433, 1251, 1113, 1043, 999, 817, 764, 700 cm^{-1}; HRMS exact mass calcd for $C_{20}H_{19}N_3NaO_3$ [M + Na]$^+$ 372.1324, found 372.1329. $[\alpha]^{29}_D$ = 43.3 °(c 0.68, $CHCl_3$); 95% ee; Chiral HPLC analysis of the product: Phenomenex 00G-4457-E0 250X4.6 mm 5u column; hexane/2-propanol = 99.2/0.8, detected at 254 nm, Flow rate = 1 mL/min, Retention times: 11.8 min (major), 13.6 min (minor).

Supplementary Figure 9. HPLC spectra of 3f

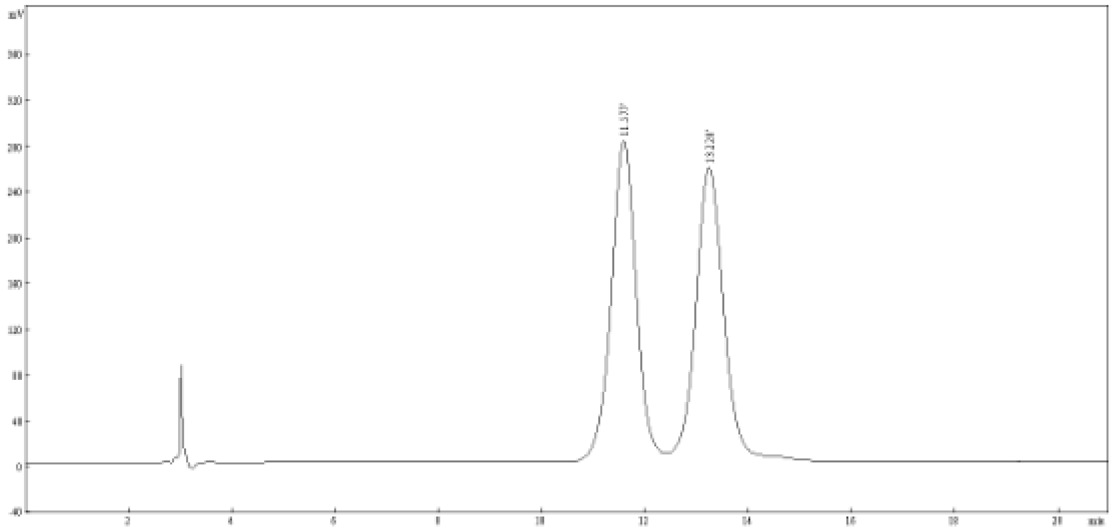

Peak#	Ret.Time	Area	Area %
1	11.577	9081269	50.01
2	13.228	9078730	49.99
Total		18159999	100

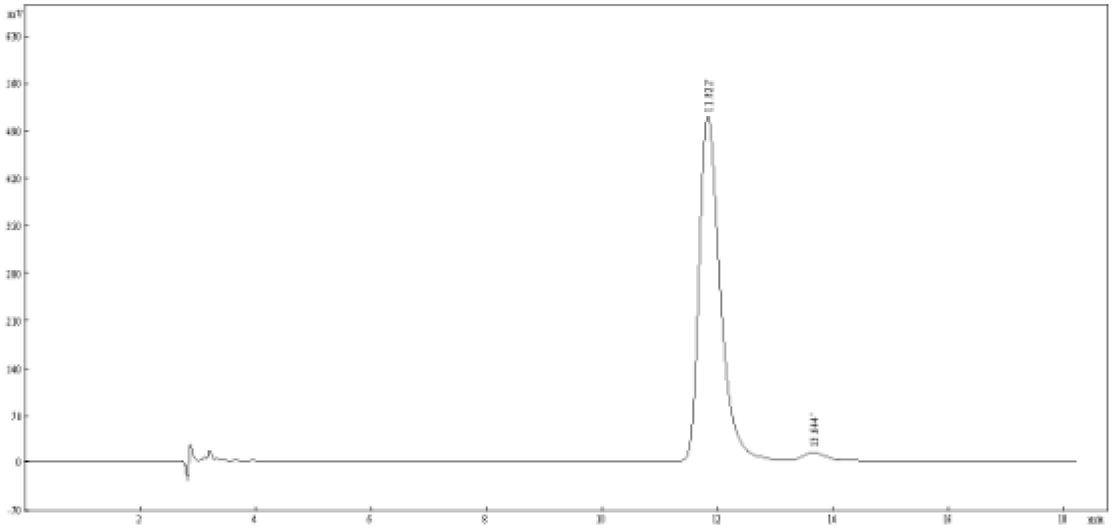

Peak#	Ret.Time	Area	Area %
1	11.822	12756181	97.81

2	13.644	286609	2.198
Total		13042790	100

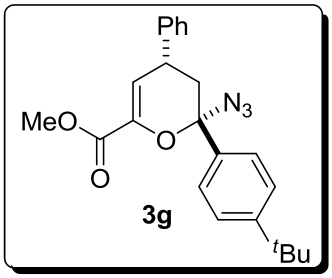

Colorless oil; (51.6 mg, 66% yield); ^{1}H NMR (500 MHz, $CDCl_3$) δ 7.46-7.41 (m, 4H), 7.33-7.30 (m, 2H), 7.27-7.24 (m, 1H), 7.21-7.19 (m, 2H), 6.19 (dd, J = 2.8, 1.0 Hz, 1H), 3.88 (s, 3H), 3.33-3.29 (m, 1H), 2.69-2.65 (m, 1H), 2.19 (dd, J = 13.8, 10.0 Hz, 1H), 1.3 (s, 9H); ^{13}C NMR (125 MHz, $CDCl_3$) δ 162.6, 152.2, 142.7, 141.9, 135.1, 128.7, 127.9, 127.1, 125.9, 124.9, 115.0, 94.4, 52.4, 39.6, 36.7, 34.6, 31.2; IR (neat): 2963, 2928, 2107, 1732, 1260, 1105, 1046, 756, 697 cm^{-1}; HRMS exact mass calcd for $C_{23}H_{25}N_3NaO_3$ [M + Na]$^+$ 414.1794, found 414.1795. $[\alpha]^{29}_D$ = 47.4 ° (c 0.56, $CHCl_3$); 98% ee; Chiral HPLC analysis of the product: Phenomenex 00G-4457-E0 250X4.6 mm 5u column; hexane/2-propanol = 99.4/0.6, detected at 254 nm, Flow rate = 1 mL/min, Retention times: 9.5 min (major), 11.2 min (minor).

Supplementary Figure 10. HPLC spectra of 3g

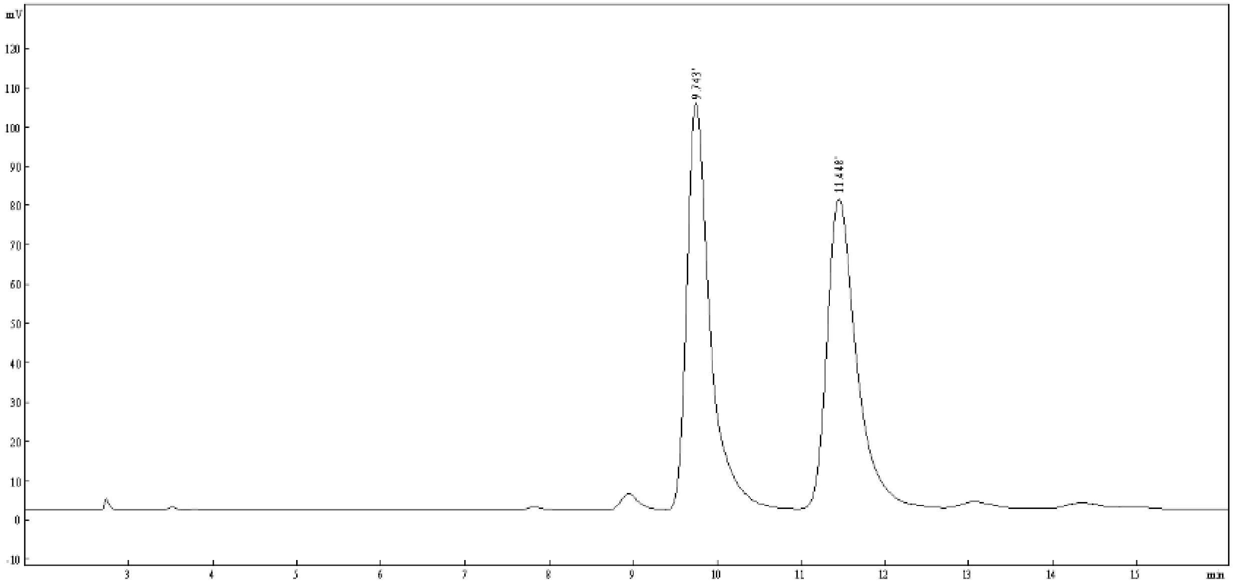

Peak#	Ret.Time	Area	Area %

1	9.743	1976466	50.79
2	11.448	1915258	49.21
Total		3891724	100

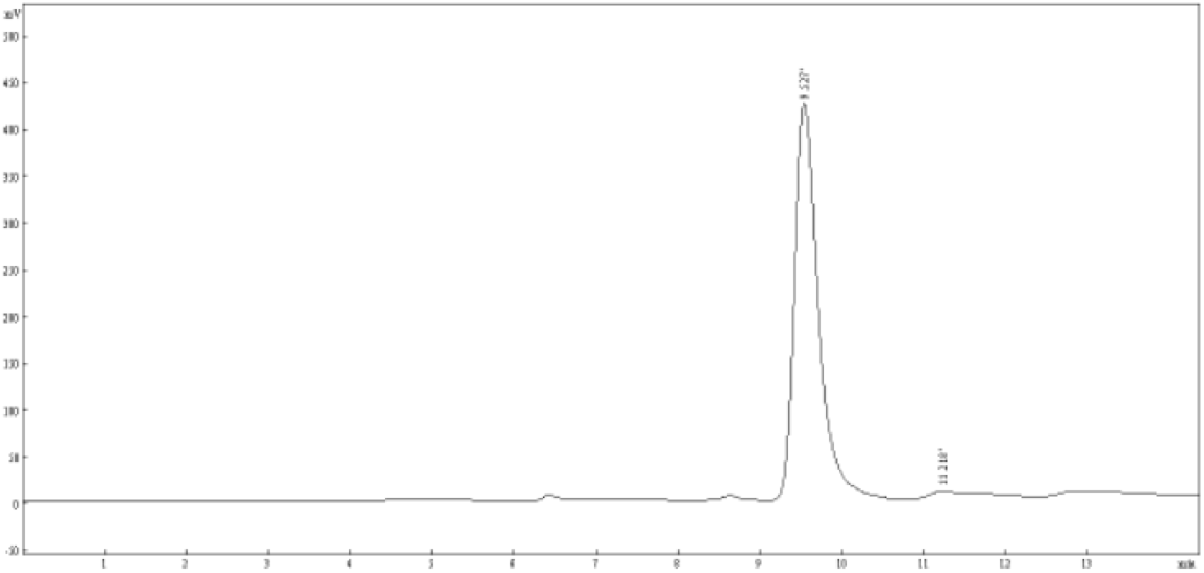

Peak#	Ret.Time	Area	Area %
1	9.527	8575077	99.02
2	11.218	85037	0.9819
Total		3891724	100

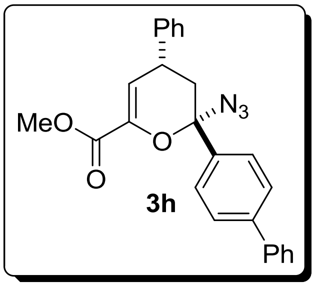

Colorless oil; (65.7 mg, 80% yield); ^{1}H NMR (500 MHz, $CDCl_3$) δ 7.68-7.66 (m, 1H), 7.62-7.57 (m, 4H), 7.45 (t, J = 7.3 Hz, 2H), 7.38-7.31 (m, 3H), 7.27-7.20 (m, 3H), 6.23 (dd, J = 2.8, 1.0 Hz, 1H), 3.89 (s, 3H), 3.36-3.32 (m, 1H), 2.71-2.67 (m, 1H), 2.25 (dd, J = 13.9, 9.9 Hz, 1H); ^{13}C NMR (125 MHz, $CDCl_3$) δ 162.5, 142.7, 142.1, 141.7, 140.1, 137.0, 128.9, 128.8, 127.7, 127.6,

127.2, 127.1, 125.7, 115.1, 94.3, 52.5, 39.7, 36.7; IR (neat): 2949, 2107, 1731, 1647, 1448, 1254, 1113, 1042, 759, 698 cm^{-1}; HRMS exact mass calcd for $C_{25}H_{21}N_3NaO_3$ $[M + Na]^+$ 434.1481, found 434.1473. $[\alpha]^{29}_D$ = 98.3 ° (c 1.08, $CHCl_3$); 97% ee; Chiral HPLC analysis of the product: Phenomenex 00G-4457-E0 250X4.6 mm 5u column; hexane/2-propanol = 99.5/0.5, detected at 254 nm, Flow rate = 1 mL/min, Retention times: 25.7 min (major), 32.9 min (minor).

Supplementary Figure 11. HPLC spectra of 3h

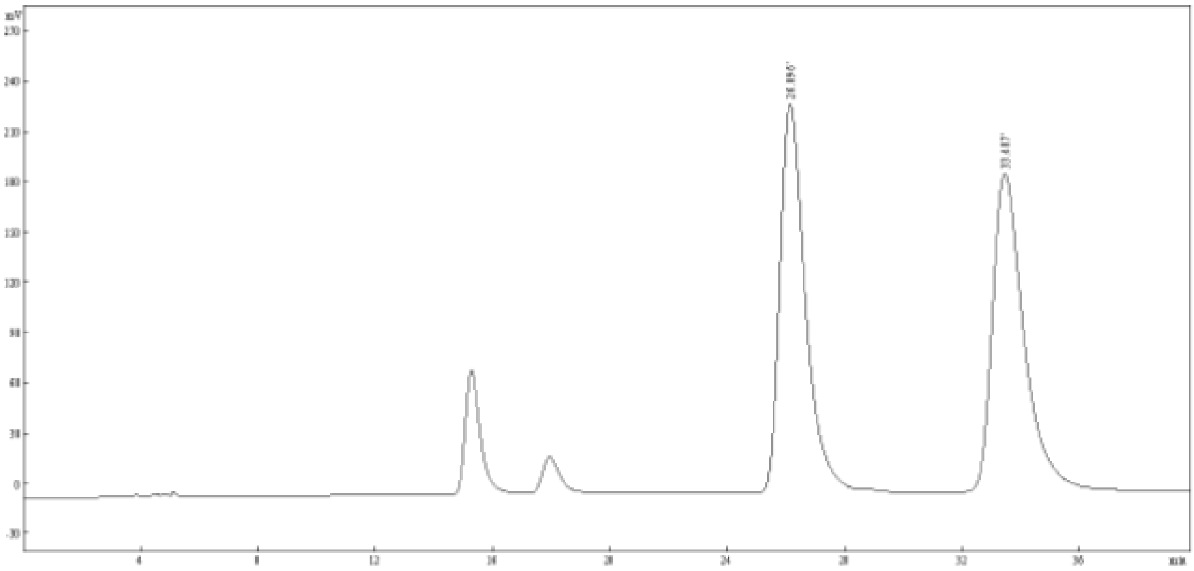

Peak#	Ret.Time	Area	Area %
1	26.096	13861294	49.28
2	33.407	14264242	50.72
Total		28125536	100

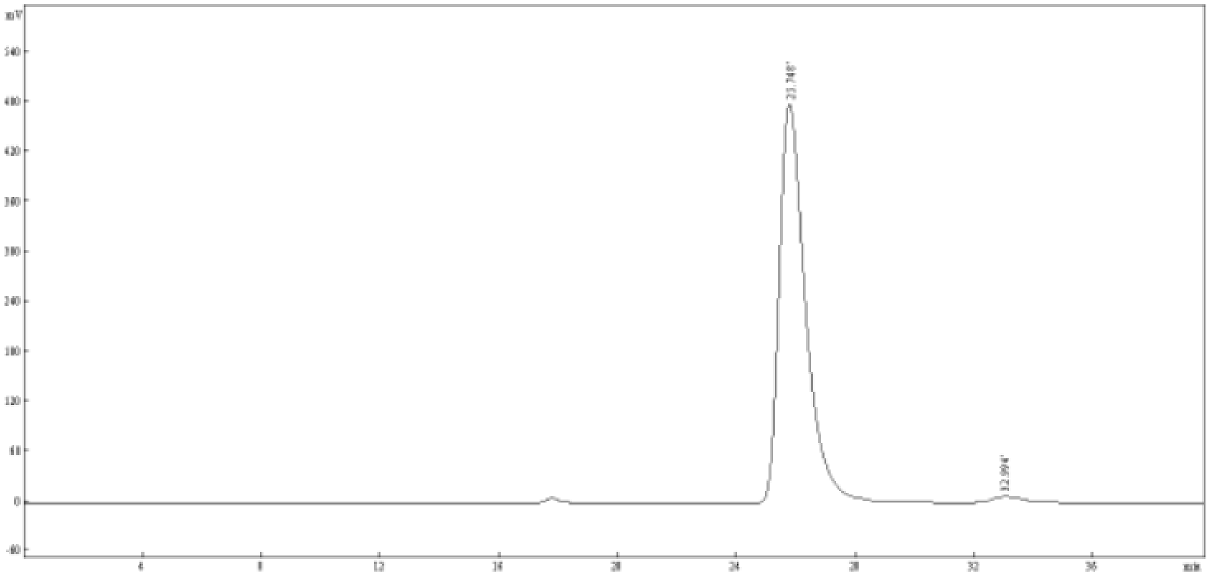

Peak#	Ret.Time	Area	Area %
1	25.748	29641178	98.32
2	32.994	504329	1.673
Total		30145507	100

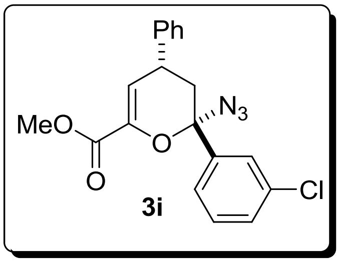

Light yellow oil; (54.6 mg, 74% yield); ^{1}H NMR (500 MHz, $CDCl_3$) δ 7.49-7.49 (m, 1H), 7.40-7.39 (m, 3H), 7.34-7.31 (m, 2H), 7.28-7.25 (m, 1H), 7.21-7.19 (m, 2H), 6.25 (dd, *J* = 3.0, 0.9 Hz, 1H), 3.89 (s, 3H), 3.33-3.29 (m, 1H), 2.60-2.56 (m, 1H), 2.22 (dd, *J* = 13.9, 9.3 Hz, 1H); ^{13}C NMR (125 MHz, $CDCl_3$) δ 162.3, 142.5, 141.5, 140.5, 135.0, 130.3, 129.5, 128.8, 127.9, 127.2, 125.6, 123.5, 115.1, 93.4, 52.5, 39.7, 36.3; IR (neat): 2931, 2110, 1735, 1653, 1433, 1248, 1105, 1043, 746, 694 cm^{-1}; HRMS exact mass calcd for $C_{19}H_{16}ClN_3NaO_3$ [M + Na]$^+$ 392.0778, found 392.0777. $[\alpha]^{28}_D$ = -2.9 ° (c 0.76, $CHCl_3$); 98% ee; Chiral HPLC analysis of the product: Phenomenex 00G-4457-E0 250X4.6 mm 5u column; hexane/2-propanol = 99.3/0.7, detected at 254 nm, Flow rate = 1 mL/min, Retention times: 12.1 min (major), 13.9 min (minor).

Supplementary Figure 12. HPLC spectra of 3i

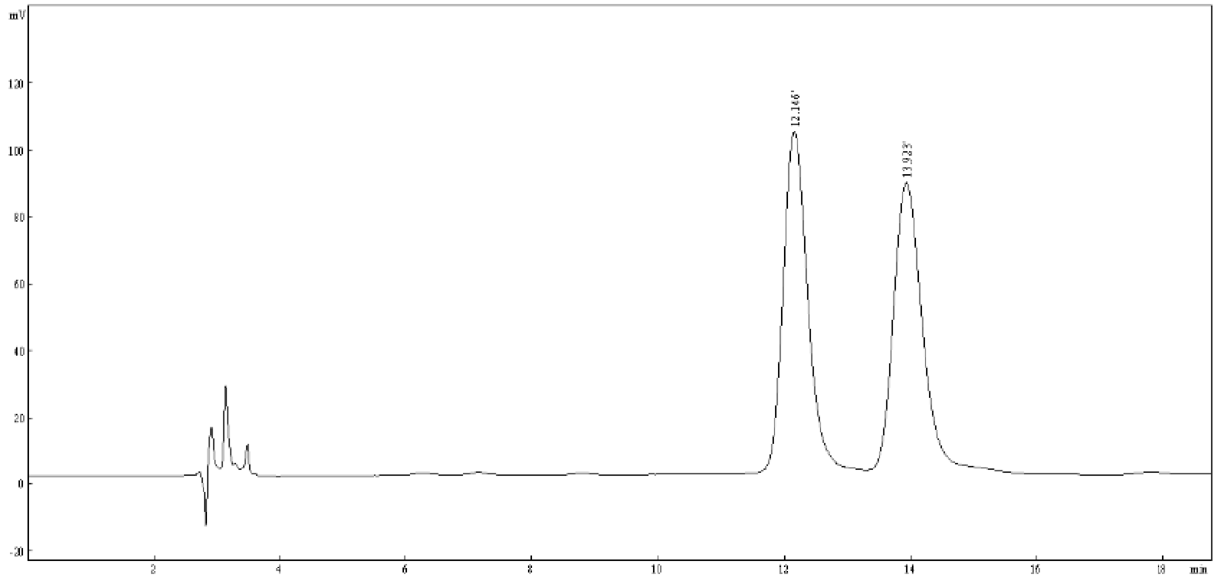

Peak#	Ret.Time	Area	Area %
1	12.146	2749311	49.9
2	13.923	2760029	50.1
Total		5509340	100

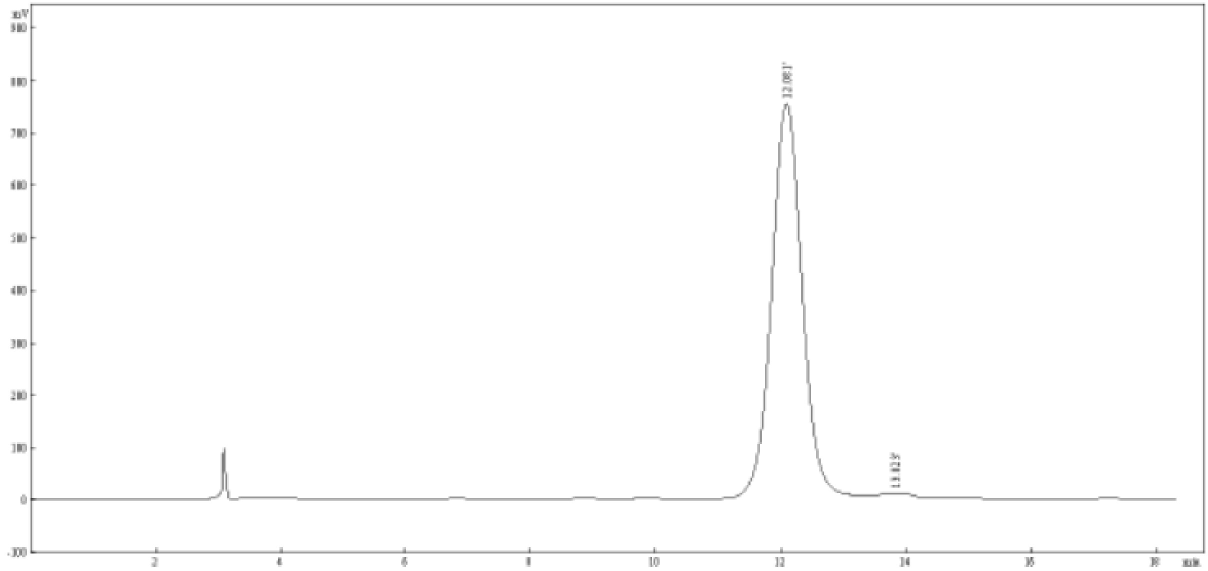

Peak#	Ret.Time	Area	Area %
1	12.081	25245518	99.24
2	13.823	194873	0.766
Total		25440391	100

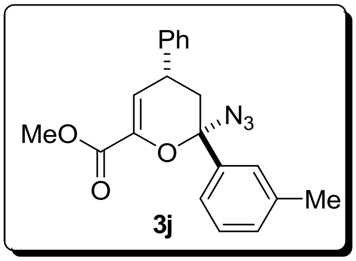

Light yellow oil; (37.6 mg, 54% yield); ^{1}H NMR (500 MHz, $CDCl_3$) δ 7.35-7.19 (m, 9H), 7.50-7.40 (m, 3H), 6.21 (dd, J = 2.8, 1.0 Hz, 1H), 3.89 (s, 3H), 3.32-3.28 (m, 1H), 2.66-2.40 (m, 1H), 2.20 (dd, J = 13.8, 9.8 Hz, 1H); ^{13}C NMR (125 MHz, $CDCl_3$) δ 162.5, 142.6, 141.8, 138.8, 138.1, 30.0, 128.9, 128.7, 127.9, 125.7, 122.3, 115.0, 94.4, 52.4, 39.7, 36.6, 21.6; IR (neat): 2925, 2855, 2110, 1729, 1439, 1257, 1105, 1040, 1014, 753, 699 cm^{-1}; HRMS exact mass calcd for $C_{20}H_{19}N_3NaO_3$ $[M + Na]^+$ 372.1324, found 372.1322. $[\alpha]^{29}_{D}$ = 5.7 ° (c 0.52, $CHCl_3$); 97% ee; Chiral HPLC analysis of the product: Phenomenex 00G-4457-E0 250X4.6 mm 5u column; hexane/2-propanol = 99.5/0.5, detected at 254 nm, Flow rate = 1 mL/min, Retention times: 14.4 min (major), 17.7 min (minor).

Supplementary Figure 13. HPLC spectra of 3j

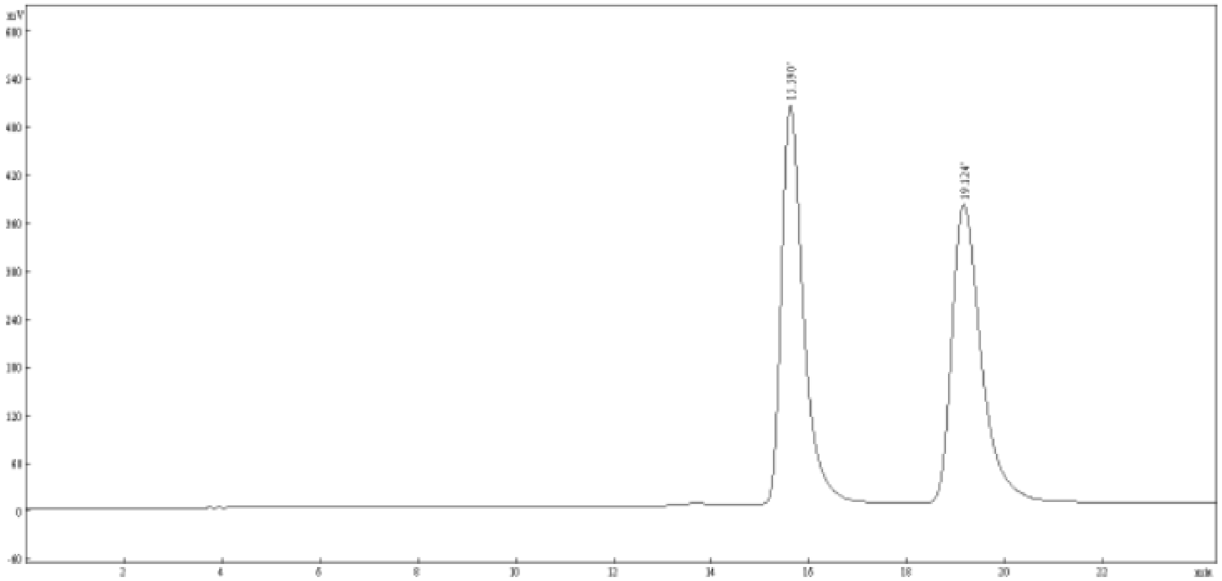

Peak#	Ret.Time	Area	Area %
1	15.590	15545419	49.85
2	19.124	15636435	50.15
Total		31181854	100

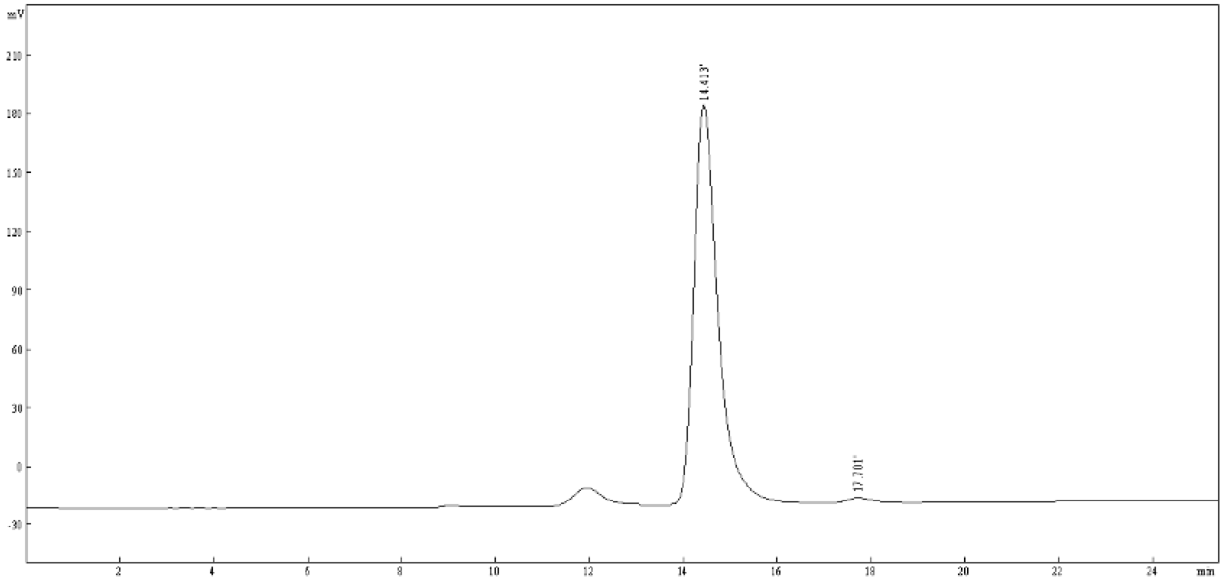

Peak#	Ret.Time	Area	Area %

1	14.413	7285082	98.76
2	17.701	91593	1.242
Total		7376675	100

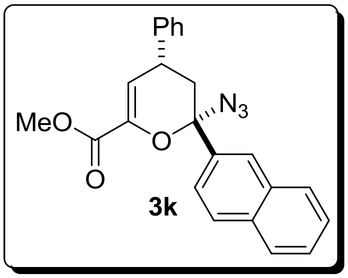

3k

Colorless oil; (67.7 mg, 88% yield); ^{1}H NMR (500 MHz, $CDCl_3$) δ 7.98-7.88 (m, 4H), 7.59-7.52 (m, 3H), 7.33-7.30 (m, 2H), 7.27-7.22 (m, 1H), 7.20-7.18 (m, 2H), 6.22 (d, J = 3.9 Hz, 1H), 3.92 (s, 3H), 3.30-3.27 (m, 1H), 2.77-2.73 (m, 1H), 2.32-2.27 (m, 1H); ^{13}C NMR (125 MHz, $CDCl_3$) δ 162.5, 142.7, 141.7, 135.3, 133.4, 132.9, 129.3, 128.8, 128.7, 127.7, 127.5, 127.3, 127.0, 126.7, 125.0, 122.4, 115.2, 94.6, 52.5, 39.8, 36.6; IR (neat): 2928, 2110, 1735, 1439, 1257, 1108, 1099, 1037, 762, 700 cm^{-1}; HRMS exact mass calcd for $C_{23}H_{19}N_3NaO_3$ [M + Na]$^+$ 408.1324, found 408.1329. $[\alpha]^{29}_D$ = 47.9 ° (c 0.76, $CHCl_3$); 97% ee; Chiral HPLC analysis of the product: Phenomenex 00G-4457-E0 250X4.6 mm 5u column; hexane/2-propanol = 99.5/0.5, detected at 254 nm, Flow rate = 1 mL/min, Retention times: 22.9 min (major), 29.3 min (minor).

Supplementary Figure 14. HPLC spectra of 3k

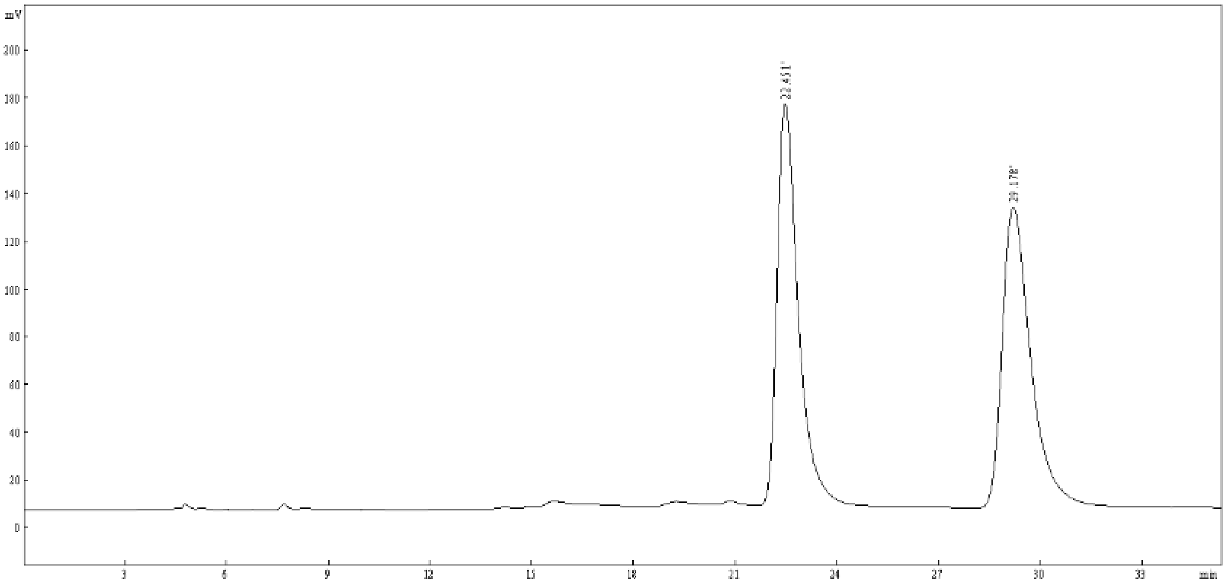

Peak#	Ret.Time	Area	Area %
1	22.451	73774046	49.85
2	29.178	7417721	50.15
Total		14791767	100

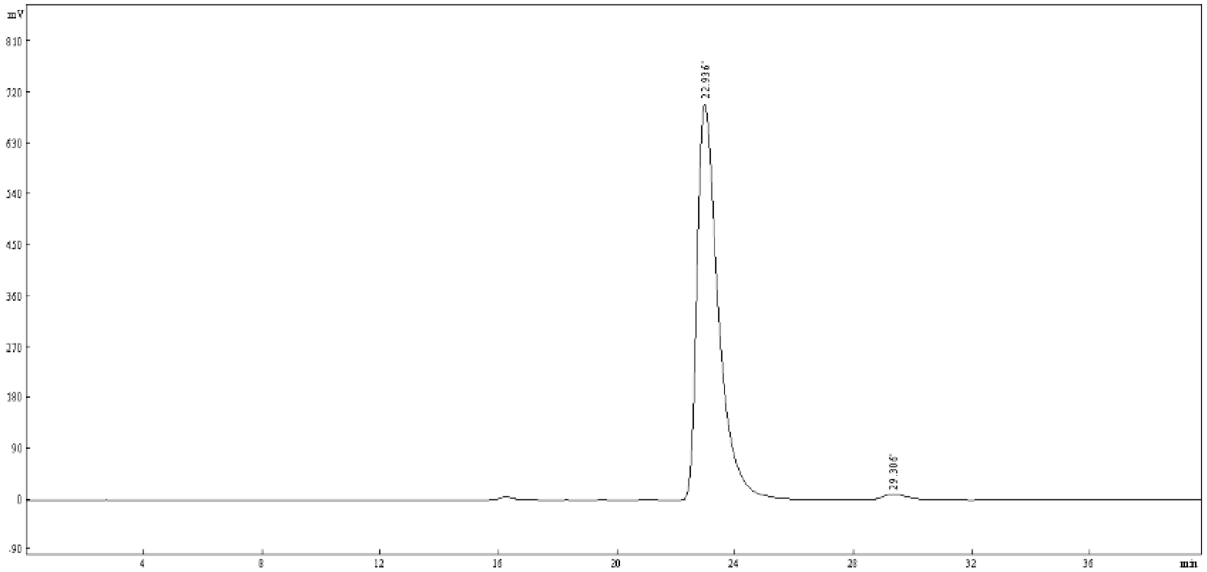

Peak#	Ret.Time	Area	Area %
1	22.936	34935232	98.55
2	29.306	514534	1.451
Total		35449766	100

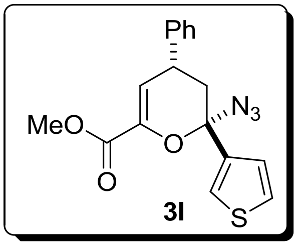

Light yellow oil; (55.2 mg, 81% yield); ^{1}H NMR (500 MHz, $CDCl_3$) δ 7.46 (dd, J = 3.0, 1.0 Hz, 1H), 7.42-7.40 (m, 1H), 7.34-7.31 (m, 2H), 7.27-7.24 (m, 1H), 7.20-7.19 (m, 2H), 7.13 (dd, J = 5.0, 1.3 Hz, 1H), 6.18 (dd, J = 2.8, 1.1 Hz, 1H), 3.87 (s, 3H), 3.38-3.34 (m, 1H), 2.62-2.58 (m, 1H), 2.19 (dd, J = 13.8, 10.0 Hz, 1H); ^{13}C NMR (125 MHz, $CDCl_3$) δ 162.5, 142.6, 141.7, 139.6,

128.8, 127.5, 127.2, 124.5, 123.6, 114.7, 92.8, 52.4, 40.0, 36.8; IR (neat): 2925, 2101, 1732, 1433, 1260, 1040, 1011, 756, 697 cm^{-1}; HRMS exact mass calcd for $C_{17}H_{15}N_3NaO_3S$ $[M + Na]^+$ 364.0732, found 364.0732. $[\alpha]^{29}_D = 36.7$ °(c 0.60, $CHCl_3$); 90% ee; Chiral HPLC analysis of the product: Phenomenex 00G-4457-E0 250X4.6 mm 5u column; hexane/2-propanol = 99.5/0.5, detected at 254 nm, Flow rate = 1 mL/min, Retention times: 22.5 min (major), 29.5 min (minor).

Supplementary Figure 15. HPLC spectra of 3l

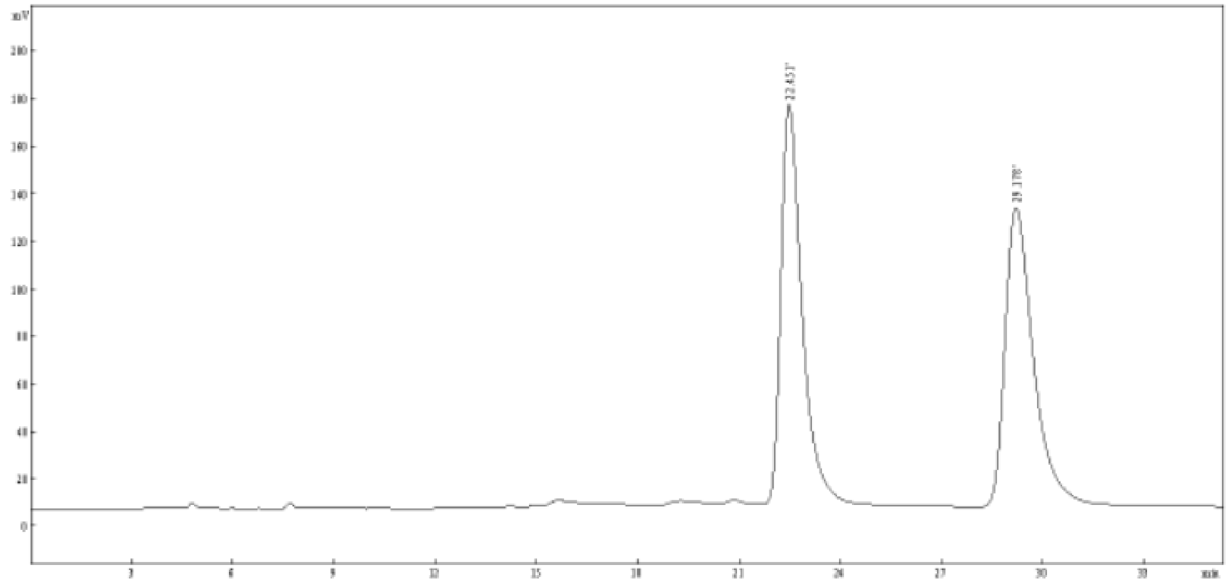

Peak#	Ret.Time	Area	Area %
1	22.451	7374046	49.85
2	29.178	7417721	50.15
Total		14791767	100

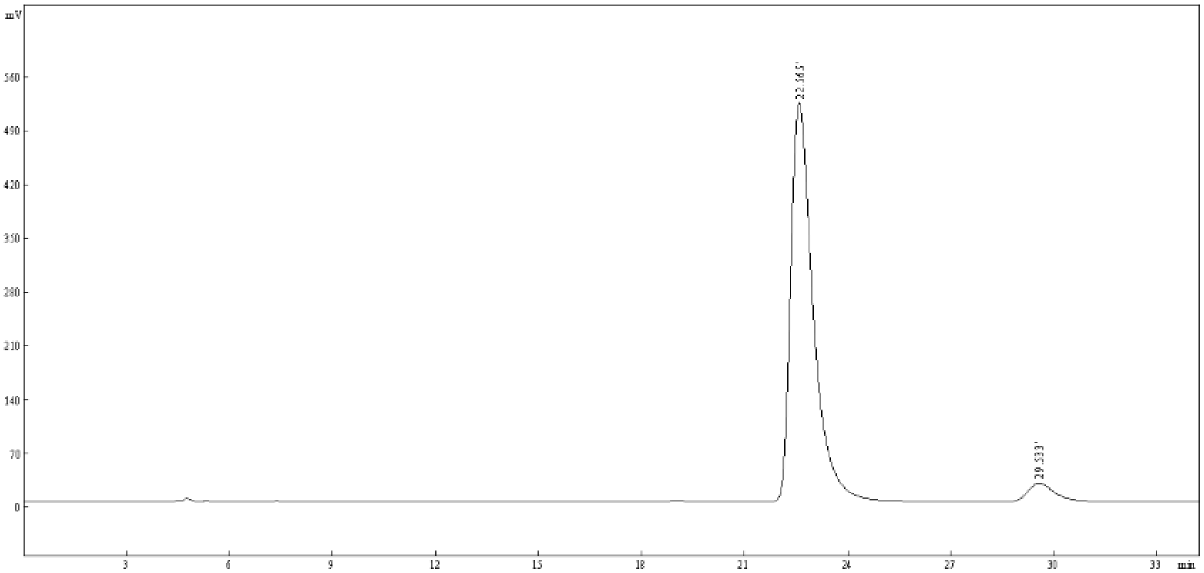

Peak#	Ret.Time	Area	Area %
1	22.565	23643829	94.73
2	29.533	1314885	5.268
Total		24958714	100

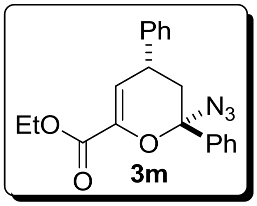

Colorless oil; (57.2 mg, 82% yield); ^{1}H NMR (500 MHz, $CDCl_3$) δ 7.53-7.51 (m, 2H), 7.47-7.39 (m, 3H), 7.33-7.30 (m, 2H), 7.27-7.23 (m, 1H), 7.20-7.18 (m, 2H), 6.18 (dd, J = 2.8, 1.1 Hz, 1H), 4.34 (q, J = 7.1 Hz, 2H), 3.30-3.26 (m, 1H), 2.68-2.64 (m, 1H), 2.22 (dd, J = 13.8, 10.0 Hz, 1H), 1.36 (t, J = 7.1 Hz, 3H); ^{13}C NMR (125 MHz, $CDCl_3$) δ 162.0, 142.8, 141.8, 138.2, 129.2, 129.0, 128.7, 127.6, 127.1, 125.2, 114.7, 94.3, 61.5, 39.7, 36.7, 14.2; IR (neat): 2957, 2107, 1732, 1242, 1031, 1014, 759, 700 cm^{-1}; HRMS exact mass calcd for $C_{20}H_{19}N_3NaO_3$ [M + Na]$^+$ 372.1324, found 372.1332. $[\alpha]^{19}_{D}$ = 15.3 ° (c 1.58, $CHCl_3$); 99% ee; Chiral HPLC analysis of the product: Phenomenex 00G-4457-E0 250X4.6 mm 5u column; hexane/2-propanol = 99.6/0.4, detected at 254 nm, Flow rate = 1 mL/min, Retention times: 22.0 min (major), 24.1 min (minor).

Supplementary Figure 16. HPLC spectra of 3m

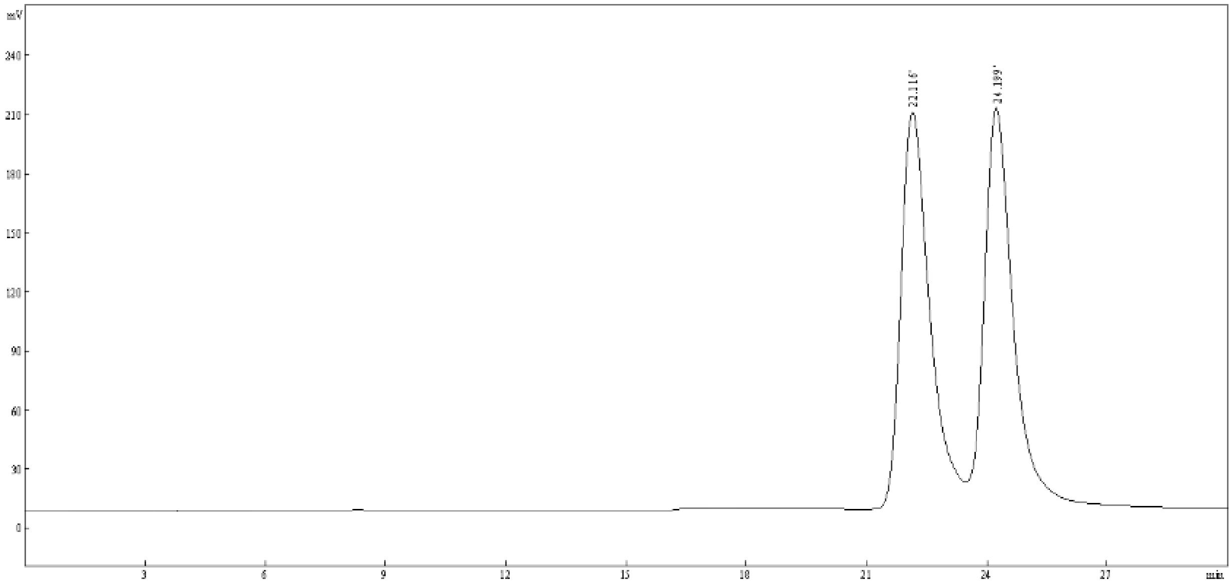

Peak#	Ret.Time	Area	Area %
1	22.116	9166102	50.83
2	24.199	8866250	49.17
Total		18032352	100

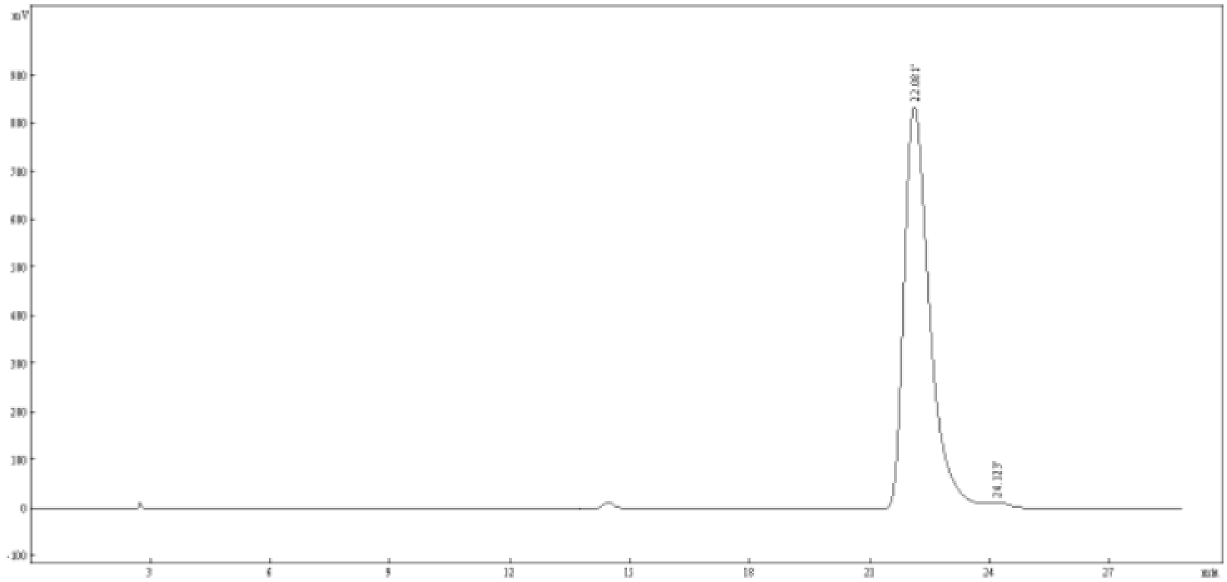

Peak#	Ret.Time	Area	Area %

1	22.081	36253597	99.59
2	24.123	149554	0.4108
Total		36403151	100

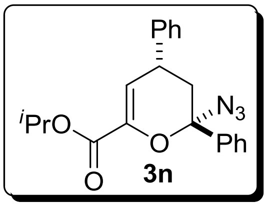

3n

Colorless oil; (58.0 mg, 80% yield); Mp: ^{1}H NMR (500 MHz, $CDCl_3$) δ 7.53-7.51 (m, 2H), 7.47-7.37 (m, 3H), 7.32 (t, J = 7.1 Hz, 2H), 7.27-7.24 (m, 1H), 7.19 (d, J = 7.1 Hz, 2H), 6.15 (dd, J = 2.8, 1.1 Hz, 1H), 5.23-5.16 (m, 1H), 3.29-3.26 (m, 1H), 2.68-2.63 (m, 1H), 2.22 (dd, J = 13.8, 10.0 Hz, 1H); ^{13}C NMR (125 MHz, $CDCl_3$) δ 161.5, 143.0, 141.9, 138.3, 129.2, 129.0, 128.7, 127.6, 127.1, 125.2, 114.4, 94.2, 69.2, 39.6, 36.7, 21.8, 21.8; IR (neat): 2990, 2104, 1714, 1278, 1260, 764, 747, 706 cm^{-1}; HRMS exact mass calcd for $C_{21}H_{21}N_3NaO_3$ [M + Na]$^+$ 386.1481, found 386.1487. $[\alpha]^{20}_{D}$ = 14.3 ° (c 1.06, $CHCl_3$); 98% ee; Chiral HPLC analysis of the product: Daicel Chiralcel OD-H 250X4.6 mm 5u column; hexane/2-propanol = 99/1, detected at 254 nm, Flow rate = 1 mL/min, Retention times: 8.5 min (major), 9.9 min (minor).

Supplementary Figure 17. HPLC spectra of 3n

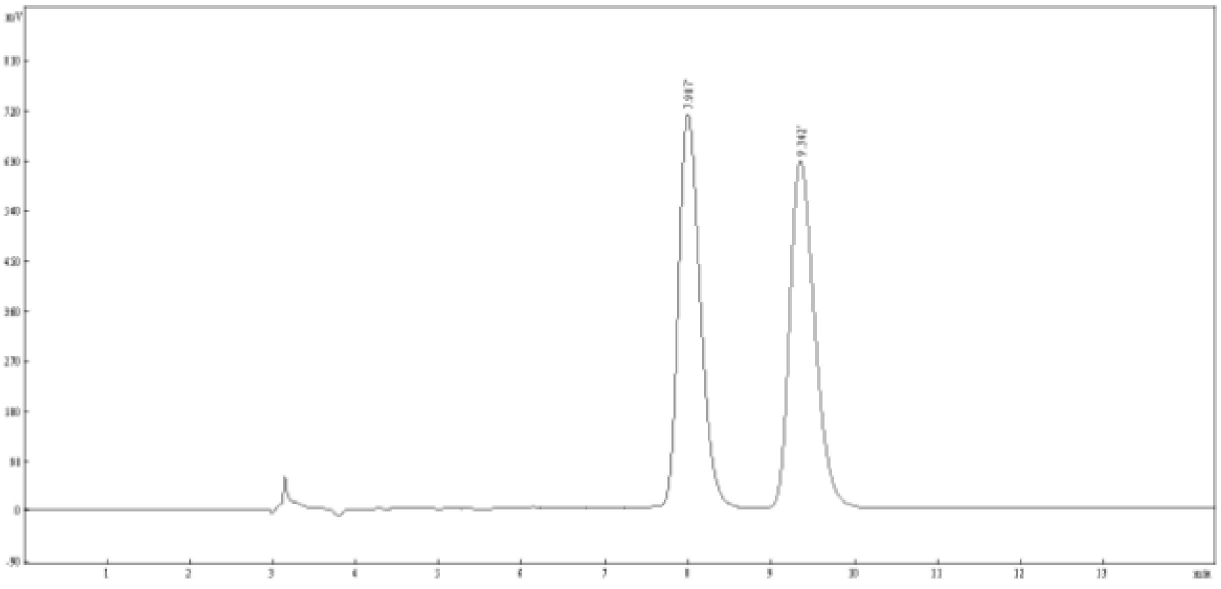

Peak#	Ret.Time	Area	Area %
1	7.987	12890567	49.92
2	9.342	12930933	50.08
Total		25821500	100

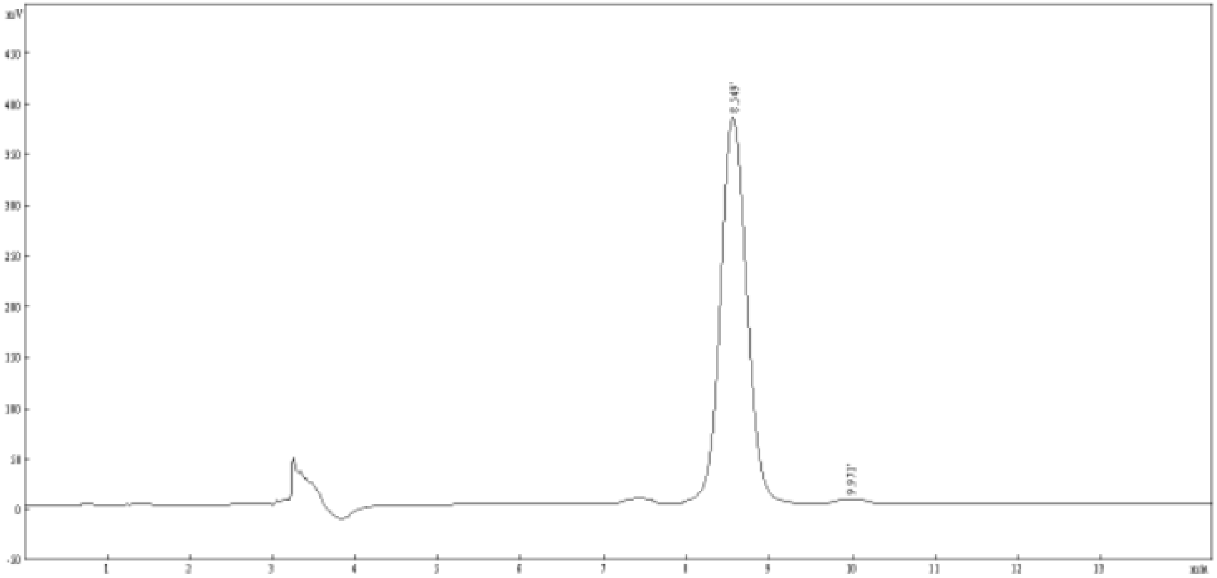

Peak#	Ret.Time	Area	Area %
1	8.549	8369922	98.94
2	9.971	89217	1.055
Total		8459139	100

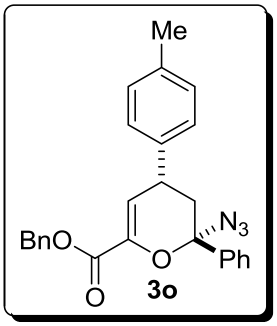

Light yellow oil; (49.3 mg, 58% yield); ^{1}H NMR (500 MHz, $CDCl_3$) δ 7.52-7.50 (m, 2H), 7.45-7.33 (m, 8H), 7.09 (dd, J = 25.0, 7.9 Hz, 4H), 6.20 (dd, J = 2.9, 1.0 Hz, 1H), 5.32 (s, 3H), 3.27-

323 (m, 1H), 2.65-2.61 (m, 1H), 2.32 (s, 3H), 2.20 (dd, J = 13.9, 9.9 Hz, 1H); ^{13}C NMR (125 MHz, $CDCl_3$) δ 161.8, 142.5, 138.7, 138.2, 136.8, 135.6, 129.4, 129.2, 129.0, 128.6, 128.3, 128.1, 127.4, 125.2, 115.6, 94.3, 66.9, 39.7, 36.3, 21.0; IR (neat): 2949, 2926, 2107, 1726, 1260, 1108, 1028, 759, 697 cm^{-1}; HRMS exact mass calcd for $C_{26}H_{23}N_3NaO_3$ [M + Na]$^+$ 448.1637, found 448.1641. $[\alpha]^{29}_D$ = -3.2 ° (c 0.56, $CHCl_3$); 98% ee; Chiral HPLC analysis of the product: Phenomenex 00G-4457-E0 250X4.6 mm 5u column; hexane/2-propanol = 99/1, detected at 254 nm, Flow rate = 1 mL/min, Retention times: 14.9 min (major), 16.7 min (minor).

Supplementary Figure 18. HPLC spectra of 3o

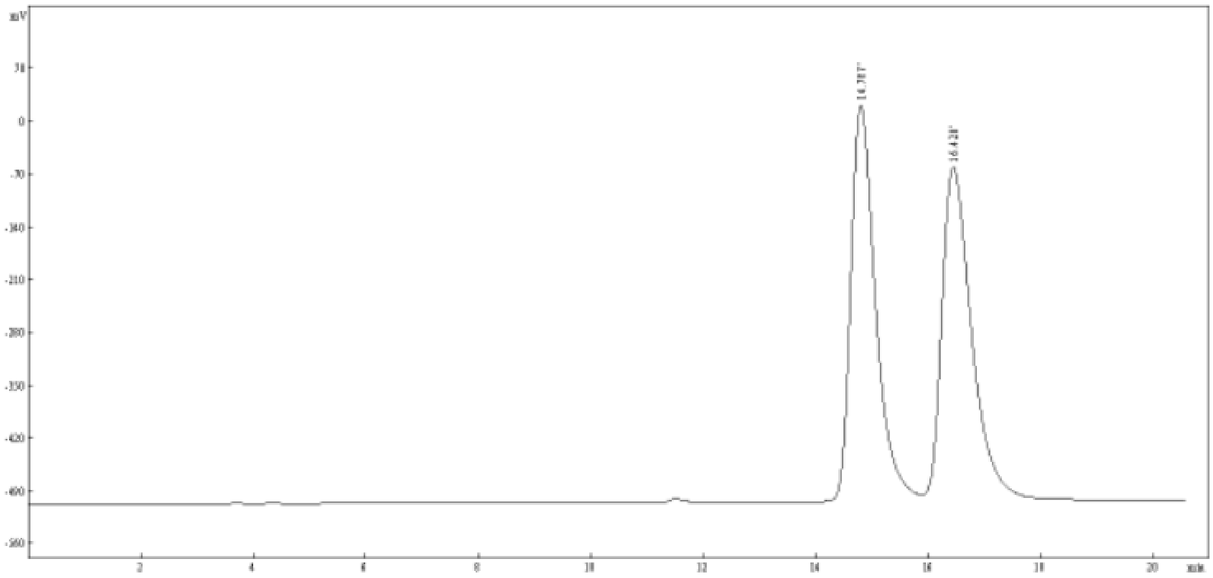

Peak#	Ret.Time	Area	Area %
1	14.787	15632192	49.93
2	16.428	15674872	50.07
Total		31307064	100

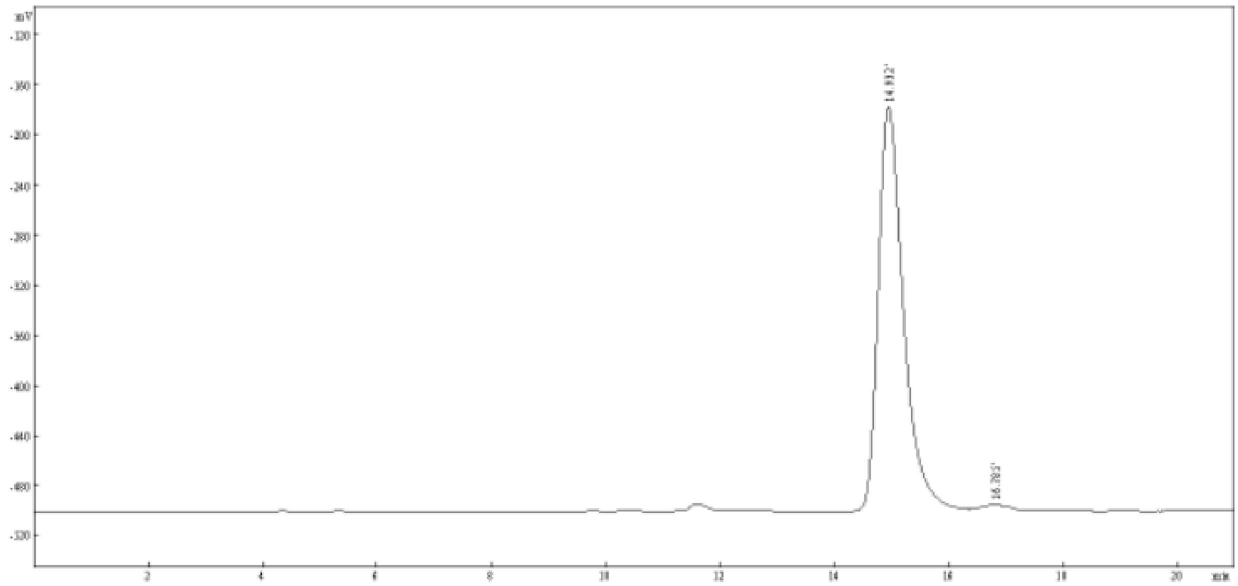

Peak#	Ret.Time	Area	Area %
1	14.932	9826989	98.95
2	16.785	104303	1.05
Total		9931292	100

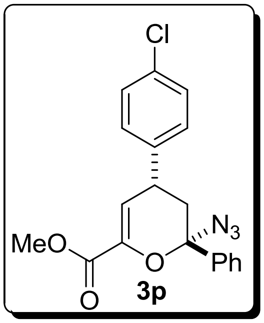

Light yellow oil; (57.5 mg, 78% yield); ^{1}H NMR (500 MHz, $CDCl_3$) δ 7.51-7.49 (m, 2H), 7.47-7.40 (m, 3H), 7.30-7.27 (m, 2H), 7.14-7.12 (m, 2H), 6.17 (dd, J = 3.0, 0.9 Hz, 1H), 3.89 (s, 3H), 3.30-3.26 (m, 1H), 2.62-2.58 (m, 1H), 2.17 (dd, J = 13.9, 10.0 Hz, 1H); ^{13}C NMR (125 MHz, $CDCl_3$) δ 162.4 142.9, 140.3, 138.0, 132.9, 129.3, 129.0, 128.9, 128.8, 125.2, 114.2, 94.2, 52.5, 39.7, 36.0; IR (neat): 2925, 2107, 1732, 1492, 1254, 1013, 762, 697 cm^{-1}; HRMS exact mass calcd for $C_{19}H_{16}ClN_3NaO_3$ [M + Na]$^+$ 392.0778, found 392.0777. $[\alpha]^{19}_D$ = 23.2 °(c 1.33, $CHCl_3$); 97% ee; Chiral HPLC analysis of the product: Phenomenex 00G-4457-E0 250X4.6 mm 5u

column; hexane/2-propanol = 99.3/0.7, detected at 254 nm, Flow rate = 1 mL/min, Retention times: 15.6 min (major), 19.4 min (minor).

Supplementary Figure 19. HPLC spectra of 3p

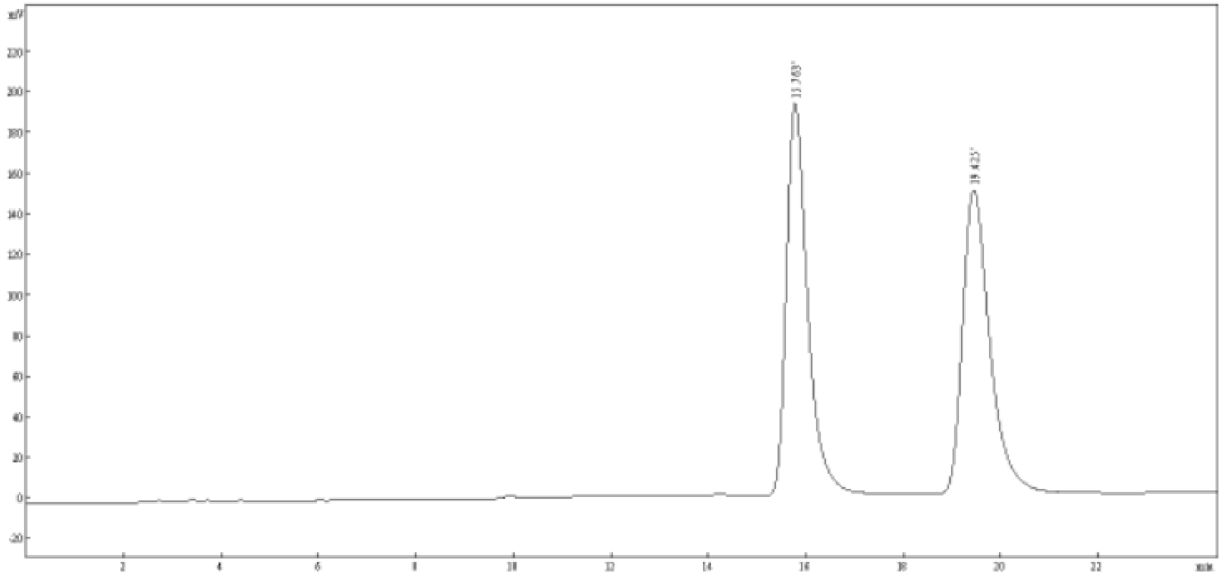

Peak#	Ret.Time	Area	Area %
1	15.763	5877008	49.98
2	19.425	5881954	50.02
Total		11758962	100

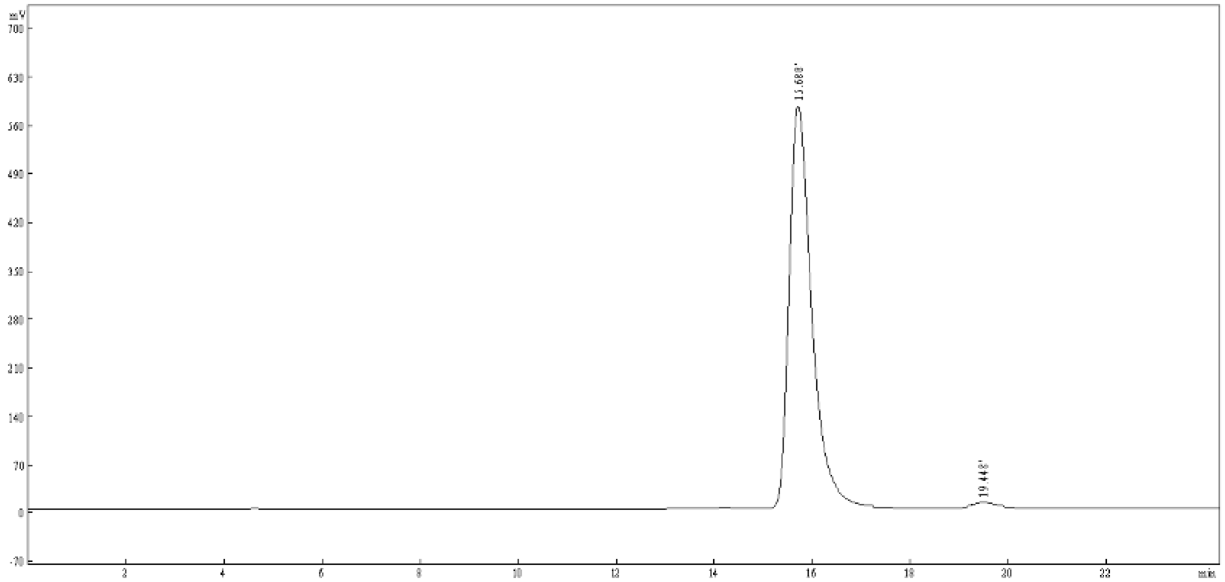

Peak#	Ret.Time	Area	Area %
1	15.688	18676533	98.44
2	19.448	295550	1.558
Total		18972083	100

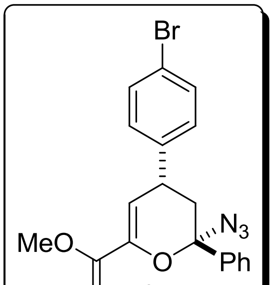

3q

Yellow oil; (61.9 mg, 75% yield); ^{1}H NMR (500 MHz, $CDCl_3$) δ 7.51-7.49 (m, 2H), 7.47-7.40 (m, 5H), 7.09-7.06 (m, 2H), 6.16 (dd, J = 3.0, 0.9 Hz, 1H), 3.89 (s, 3H), 3.29-3.25 (m, 1H), 2.62-2.58 (m, 1H), 2.17 (dd, J = 13.9, 9.3 Hz, 1H); ^{13}C NMR (125 MHz, $CDCl_3$) δ 162.4, 142.9, 140.8, 138.0, 131.8, 129.3, 129.0, 125.2, 120.9, 114.1, 94.2, 52.5, 39.7, 36.0; IR (neat): 2952, 2110, 1732, 1489, 1242, 1113, 1008, 756, 703 cm^{-1}; HRMS exact mass calcd for $C_{19}H_{16}BrN_3NaO_3$ [M + Na]$^+$ 436.0271, found 436.0271. $[\alpha]^{19}_{D}$ = 28.0 °(c 1.37, $CHCl_3$); 97% ee; Chiral HPLC analysis of the product: Phenomenex 00G-4457-E0 250X4.6 mm 5u column; hexane/2-propanol = 99.3/0.7, detected at 254 nm, Flow rate = 1 mL/min, Retention times: 17.3 min (major), 21.6 min (minor).

Supplementary Figure 20. HPLC spectra of 3q

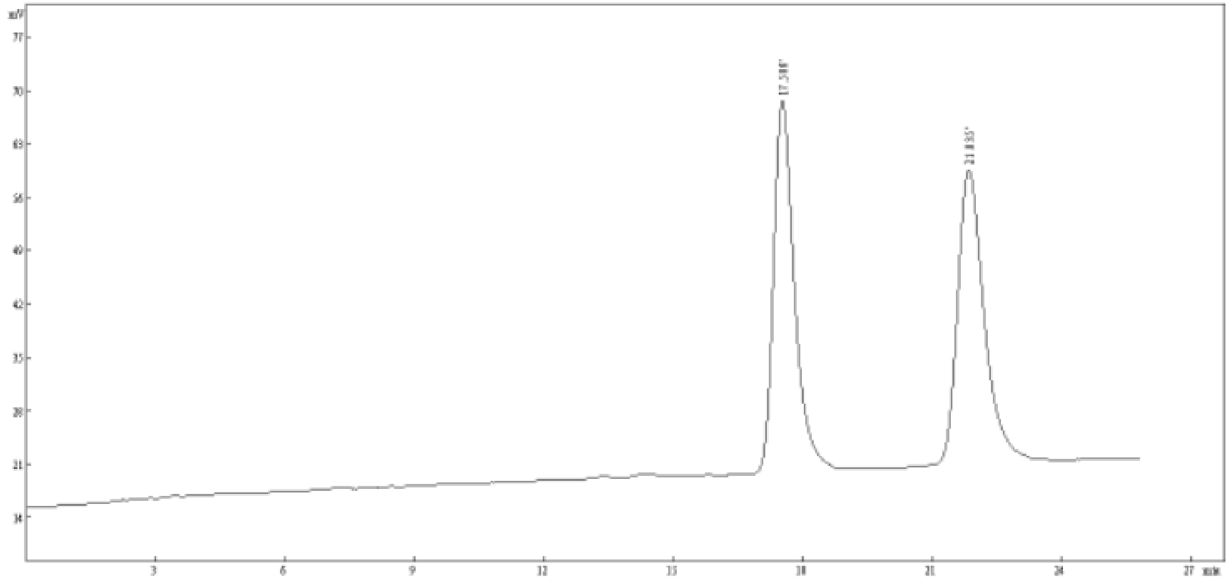

Peak#	Ret.Time	Area	Area %
1	17.506	1643558	49.68
2	21.835	1664764	50.32
Total		3308322	100

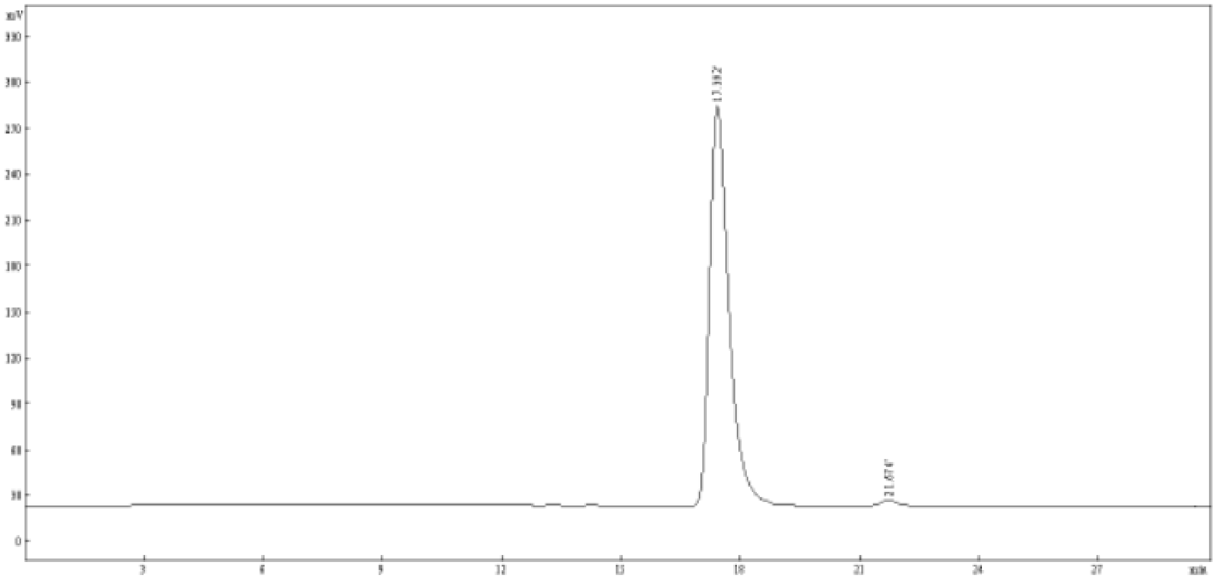

Peak#	Ret.Time	Area	Area %

1	17.392	8980751	98.43
2	21.674	143458	1.572
Total		9124209	100

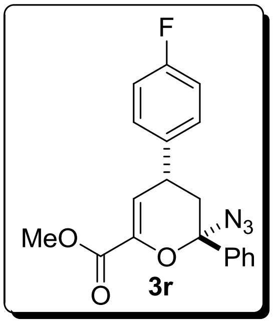

Light yellow oil; (60.7 mg, 86% yield); ^{1}H NMR (500 MHz, $CDCl_3$) δ 7.52-7.50 (m, 2H), 7.47-7.41 (m, 3H), 7.17-7.14 (m, 2H), 7.00 (t, J = 8.6 Hz, 1H), 6.17 (dd, J = 2.9, 1.0 Hz, 1H), 3.89 (s, 3H), 3.30-3.26 (m, 1H), 2.64-2.60 (m, 1H), 2.17 (dd, J = 13.9, 9.6 Hz, 1H); ^{13}C NMR (125 MHz, $CDCl_3$) δ 162.4, 161.5 (d, J = 240 Hz), 142.7, 137.5 (d, J = 3 Hz), 129.3, 129.1, 129.0, 125.2, 115.5 (d, J = 21 Hz), 114.6, 94.3, 52.5, 39.9, 35.9; IR (neat): 2949, 2107, 1732, 1506, 1260, 1200, 1108, 835, 760, 703 cm^{-1}; HRMS exact mass calcd for $C_{19}H_{16}FrN_3NaO_3$ [M + Na]$^+$ 376.3368, found 376.1072. $[\alpha]^{20}_D$ = 2.1 ° (c 1.51, $CHCl_3$); 97% ee; Chiral HPLC analysis of the product: Phenomenex 00G-4457-E0 250X4.6 mm 5u column; hexane/2-propanol = 99.3/0.7, detected at 254 nm, Flow rate = 1 mL/min, Retention times: 14.7 min (major), 17.9 min (minor).

Supplementary Figure 21. HPLC spectra of 3r

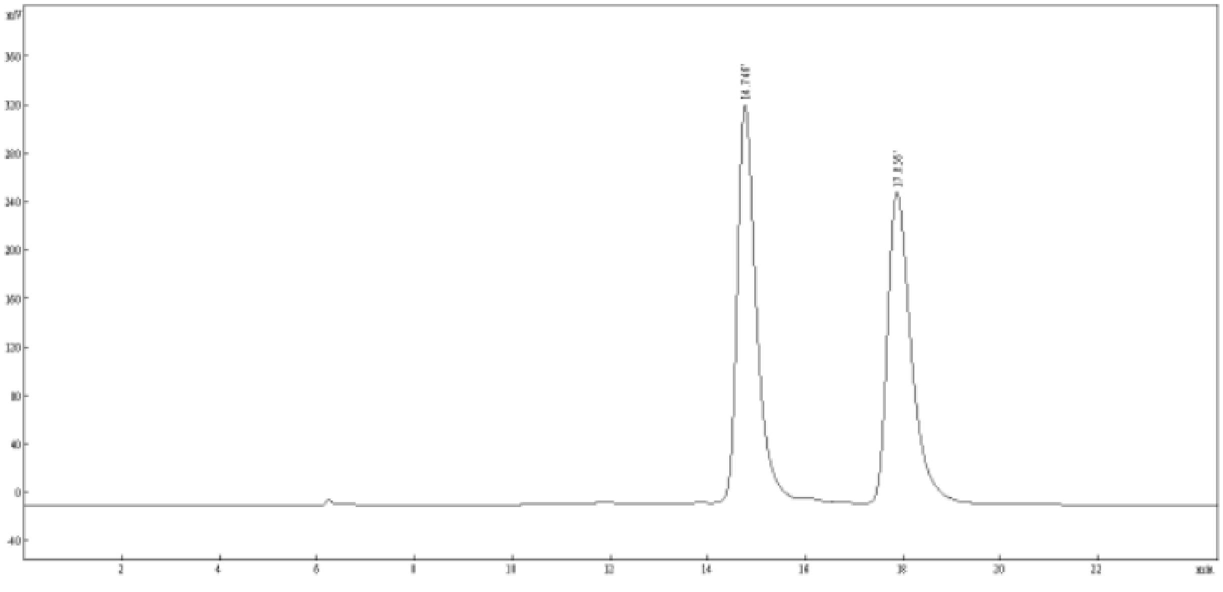

Peak#	Ret.Time	Area	Area %
1	14.746	9032638	50.96
2	17.856	8693052	49.04
Total		17725690	100

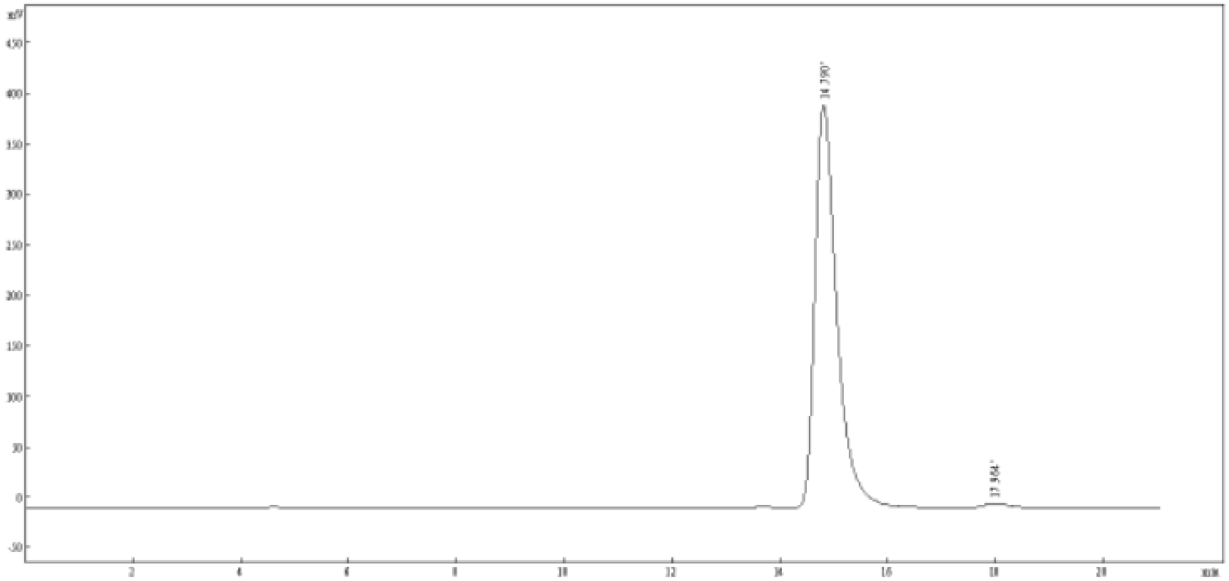

Peak#	Ret.Time	Area	Area %
1	14.790	11244635	98.64
2	17.964	154427	1.355
Total		11399062	100

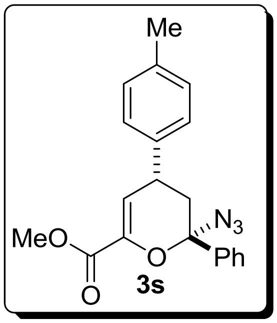

White solid; (65.6 mg, 94% yield); ^{1}H NMR (500 MHz, $CDCl_3$) δ 7.53-7.51 (m, 1H), 7.48-7.42 (m, 3H), 7.11 (dd, J = 25.3, 7.9 Hz, 2H), 6.20 (dd, J = 2.8, 0.9 Hz, 1H), 3.89 (s, 3H), 3.28-3.24

(m, 1H), 2.67-2.63 (m, 1H), 2.34 (s, 3H), 2.21 (dd, J = 13.9, 9.9 Hz, 1H); ^{13}C NMR (125 MHz, $CDCl_3$) δ 162.5, 142.5, 138.7, 138.2, 136.8, 129.4, 129.2, 129.0, 127.4, 125.2, 115.4, 94.4, 52.4, 39.8, 36.2, 21.0; IR (neat): 2949, 2110, 1740, 1637, 1438, 1230, 1110, 1055, 758, 699 cm^{-1}; HRMS exact mass calcd for $C_{20}H_{19}N_3NaO_3$ $[M + Na]^+$ 372.1324, found 372.1327. $[\alpha]^{19}_D$ = 26.9 ° (c 1.37, $CHCl_3$); 99% ee; Chiral HPLC analysis of the product: Phenomenex 00G-4457-E0 250X4.6 mm 5u column; hexane/2-propanol = 99.3/0.7, detected at 254 nm, Flow rate = 1 mL/min, Retention times: 13.7 min (major), 15.0 min (minor).

Supplementary Figure 22. HPLC spectra of 3s

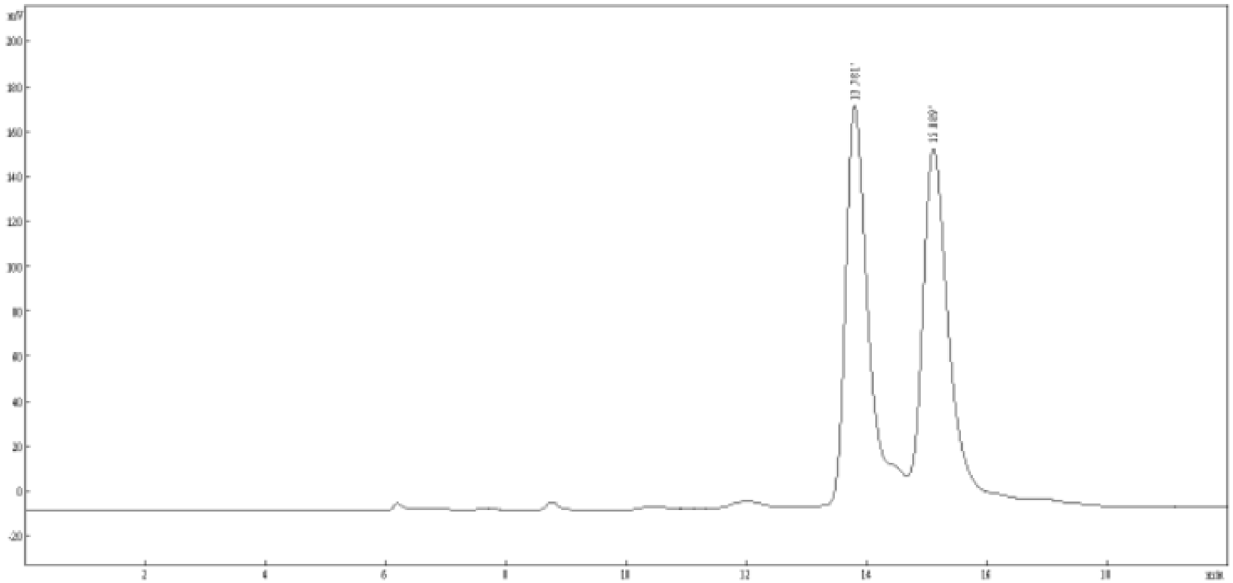

Peak#	Ret.Time	Area	Area %
1	13.781	4189492	50.91
2	15.089	4040281	49.09
Total		8229773	100

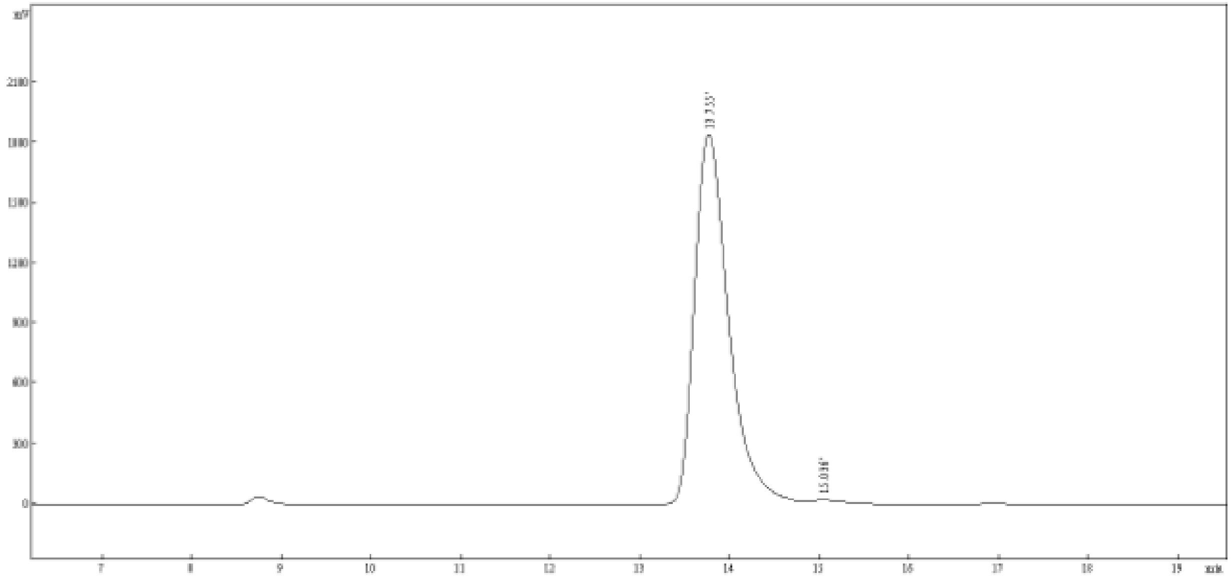

Peak#	Ret.Time	Area	Area %
1	13.755	48163862	99.54
2	15.036	225903	0.4669
Total		48389765	100

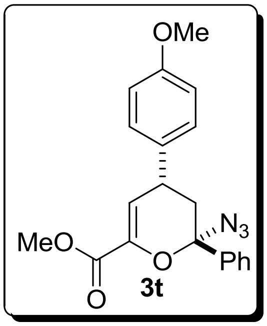

Light yellow oil; (42.3 mg, 58% yield); ^{1}H NMR (500 MHz, $CDCl_3$) δ 7.52-7.49 (m, 2H), 7.47-7.39 (m, 3H), 7.10 (d, J = 8.6 Hz, 2H), 6.85 (d, J = 8.6 Hz, 2H), 6.17 (dd, J = 2.8, 1.0 Hz, 1H), 3.88 (s, 3H), 3.79 (s, 3H), 3.25-3.21 (m, 1H), 2.65-2.60 (m, 1H), 2.18 (dd, J = 13.9, 9.9 Hz, 1H); ^{13}C NMR (125 MHz, $CDCl_3$) δ 162.5, 158.6, 142.4, 138.2, 133.7, 129.2, 129.0, 128.5, 125.2, 115.5, 114.1, 94.3, 55.3, 52.4, 39.9, 35.8; IR (neat): 2954, 2107, 1726, 1512, 1242, 1031, 1025, 756, 700 cm^{-1}; HRMS exact mass calcd for $C_{20}H_{19}N_3NaO_4$ [M + Na]$^+$ 388.1273, found 388.1272. $[\alpha]^{19}_{D}$ = 35.9 °(c 0.92, $CHCl_3$); 98% ee; Chiral HPLC analysis of the product: Phenomenex 00G-

4457-E0 250X4.6 mm 5u column; hexane/2-propanol = 99.2/0.8, detected at 254 nm, Flow rate = 1 mL/min, Retention times: 25.0 min (major), 29.3 min (minor).

Supplementary Figure 23. HPLC spectra of 3t

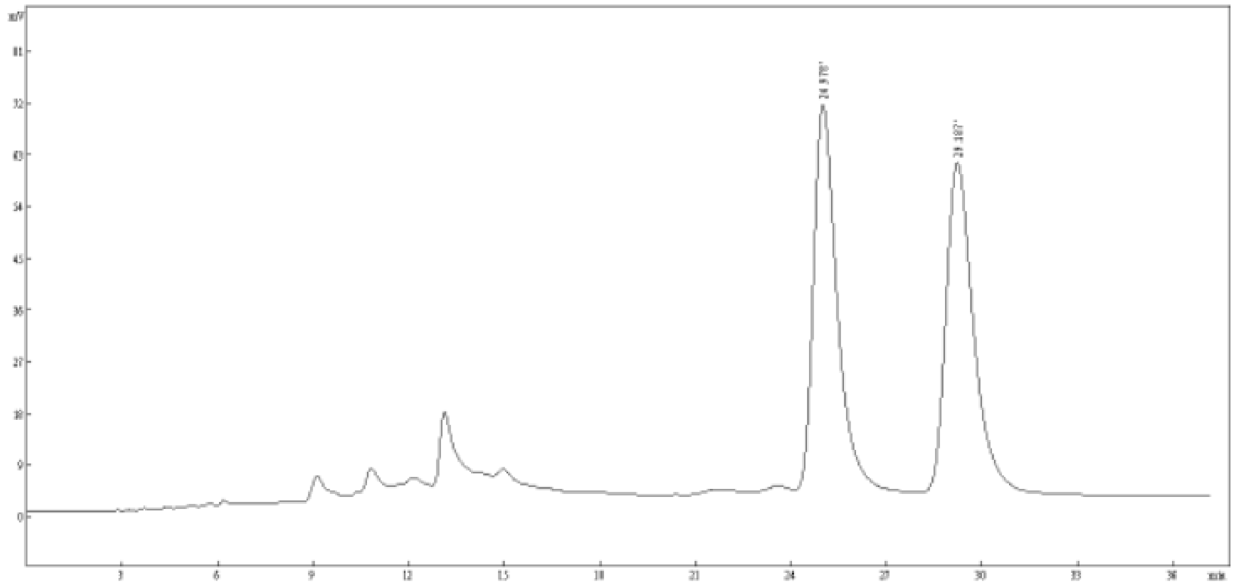

Peak#	Ret.Time	Area	Area %
1	24.978	3452964	49.88
2	29.187	3470113	50.12
Total		6923077	100

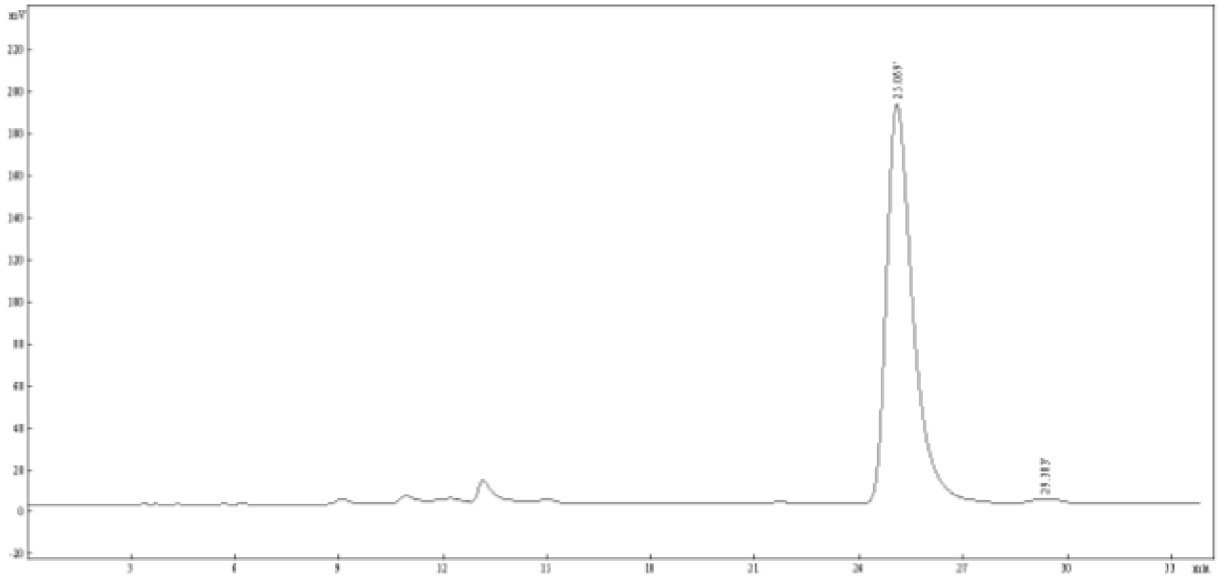

Peak#	Ret.Time	Area	Area %
1	25.069	9961314	99.03
2	29.303	97785	0.9721
Total		10059099	100

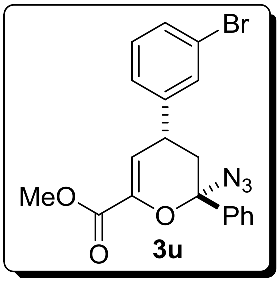

3u

White solid; (55.3 mg, 67% yield); ^{1}H NMR (500 MHz, $CDCl_3$) δ 7.51-7.49 (m, 2H), 7.47-7.37 (m, 4H), 7.34 (t, J = 1.7 Hz, 1H), 7.19 (t, J = 7.8 Hz, 1H), 7.12 (d, J = 7.8 Hz, 1H), 6.16 (dd, J = 2.9, 0.9 Hz, 1H), 3.89 (s, 3H), 3.28-3.24 (m, 1H), 2.64-2.60 (m, 1H), 2.18 (dd, J = 13.9, 9.5 Hz, 1H); ^{13}C NMR (125 MHz, $CDCl_3$) δ 162.3, 144.1, 143.0, 137.9, 130.7, 130.3, 130.3, 129.3, 129.1, 126.2, 125.2, 122.7, 113.8, 94.2, 52.5, 39.6, 36.2; IR (neat): 2954, 2107, 1729, 1437, 1240, 1113, 1108, 1028, 759, 697 cm^{-1}; HRMS exact mass calcd for $C_{19}H_{16}BrN_3NaO_3$ [M + Na]$^+$ 436.0273, found 436.0264. $[\alpha]^{20}_D$ = 11.5 °(c 1.45, $CHCl_3$); 97% ee; Chiral HPLC analysis of the product: Phenomenex 00G-4457-E0 250X4.6 mm 5u column; hexane/2-propanol = 99.3/0.7, detected at 254 nm, Flow rate = 1 mL/min, Retention times: 13.5 min (major), 15.4 min (minor).

Supplementary Figure 24. HPLC spectra of 3u

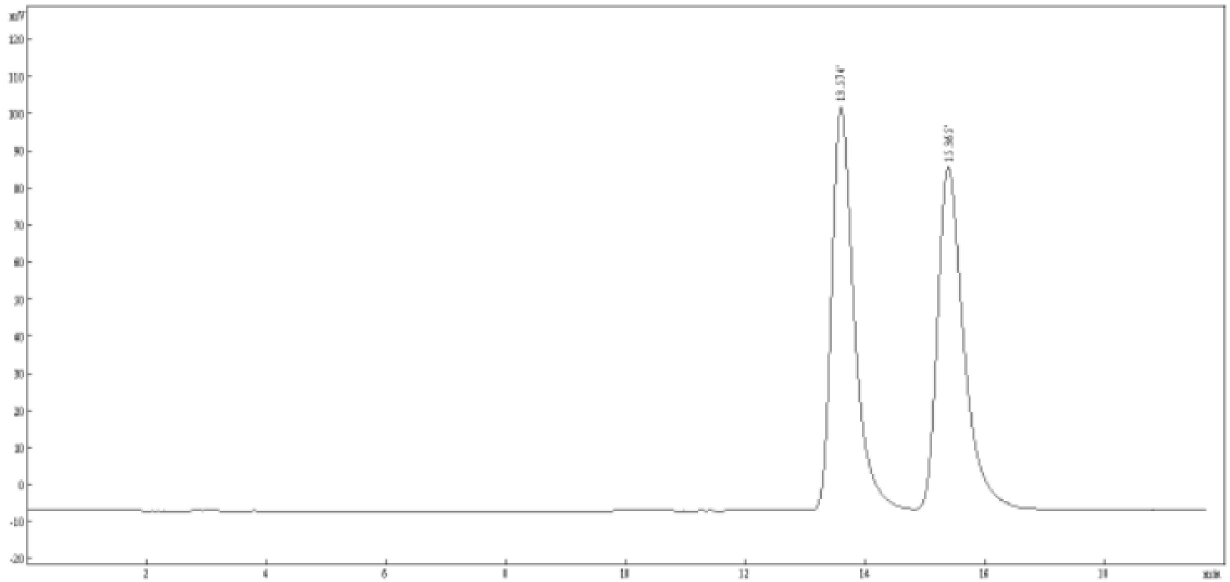

Peak#	Ret.Time	Area	Area %
1	13.574	2835467	50.19
2	15.365	2813976	49.81
Total		5649443	100

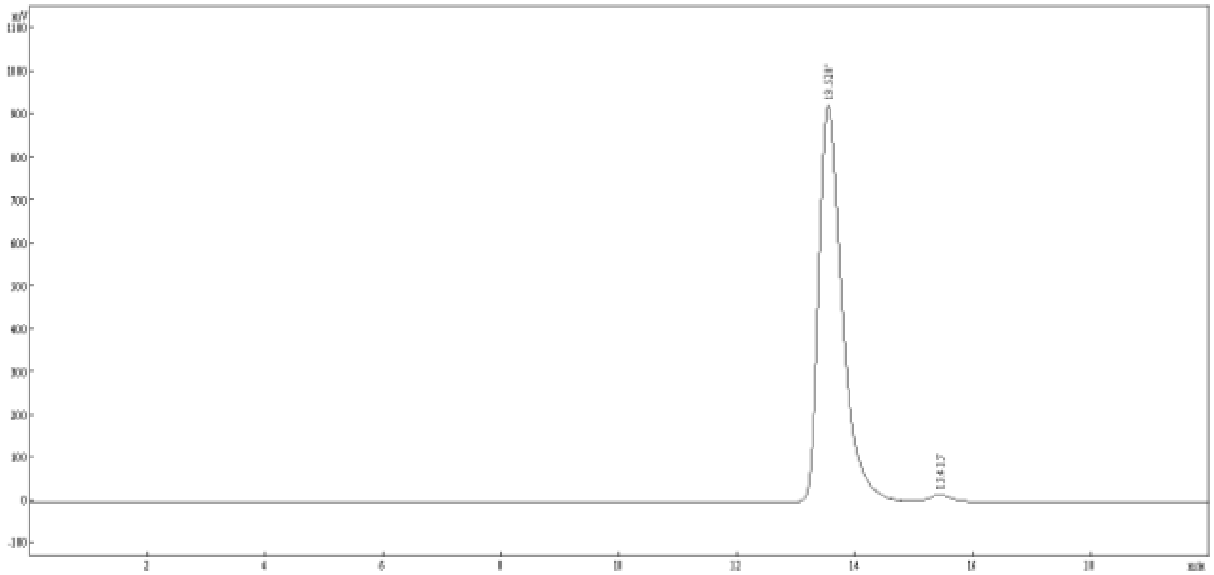

Peak#	Ret.Time	Area	Area %

1	13.528	25643982	98.37
2	15.415	423491	1.625
Total		26067473	100

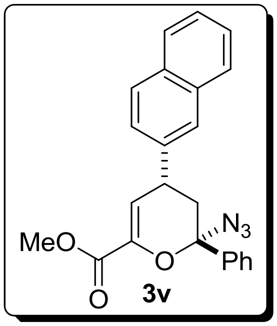

3v

Colorless oil; (62.3 mg, 81% yield); ^{1}H NMR (500 MHz, $CDCl_3$) δ 7.81-7.77 (m, 3H), 7.62 (s, 1H), 7.55-7.52 (m, 2H), 7.48-7.41 (m, 2H), 7.39-7.29 (m, 1H), 6.18 (dd, *J* = 2.8, 1.1 Hz, 1H), 3.90 (s, 3H), 3.46-3.43 (m, 1H), 2.74-2.70 (m, 1H), 2.31 (dd, *J* = 13.8, 9.9 Hz, 1H); ^{13}C NMR (125 MHz, $CDCl_3$) δ 162.5, 142.8, 139.1, 138.2, 133.5, 132.5, 129.3, 129.1, 128.5, 127.7, 127.6, 126.3, 126.1, 125.9, 125.7, 125.3, 114.7, 94.4, 52.5, 39.7, 36.8; IR (neat): 2972, 2101, 1735, 1257, 1025, 753, 703, 477 cm^{-1}; HRMS exact mass calcd for $C_{23}H_{19}N_3NaO_3$ $[M + Na]^+$ 408.1324, found 408.1328. $[\alpha]^{19}_D$ = 69.3 ° (c 1.25, $CHCl_3$); 99% ee; Chiral HPLC analysis of the product: Phenomenex 00G-4457-E0 250X4.6 mm 5u column; hexane/2-propanol = 99.3/0.7, detected at 254 nm, Flow rate = 1 mL/min, Retention times: 18.7 min (major), 21.1 min (minor).

Supplementary Figure 25. HPLC spectra of 3v

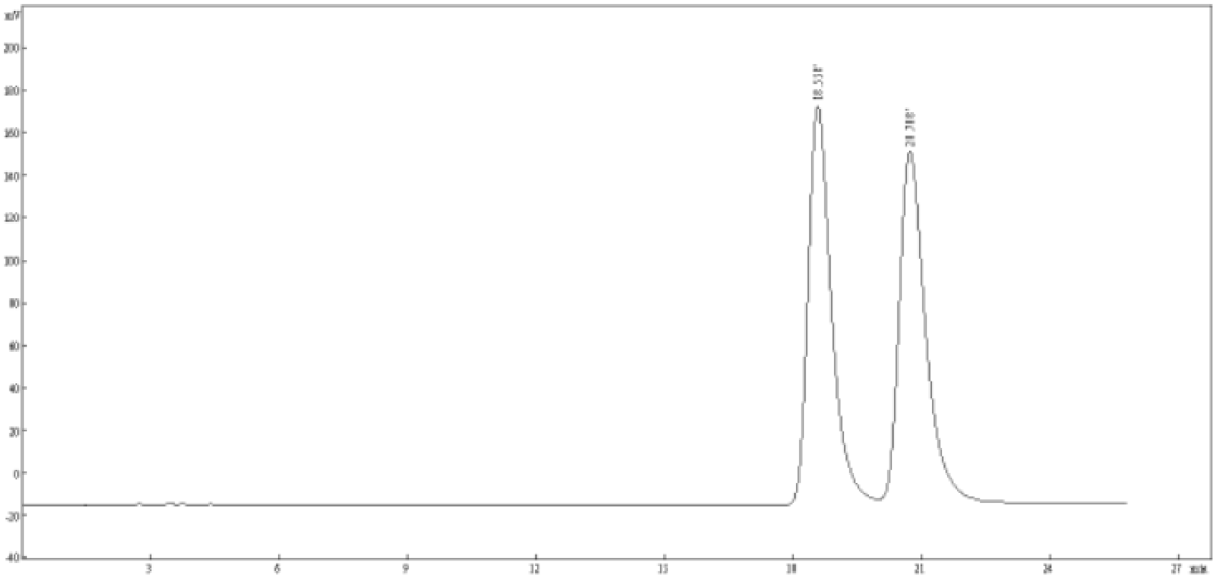

Peak#	Ret.Time	Area	Area %
1	18.558	6951586	49.98
2	20.708	6958049	50.02
Total		13909635	100

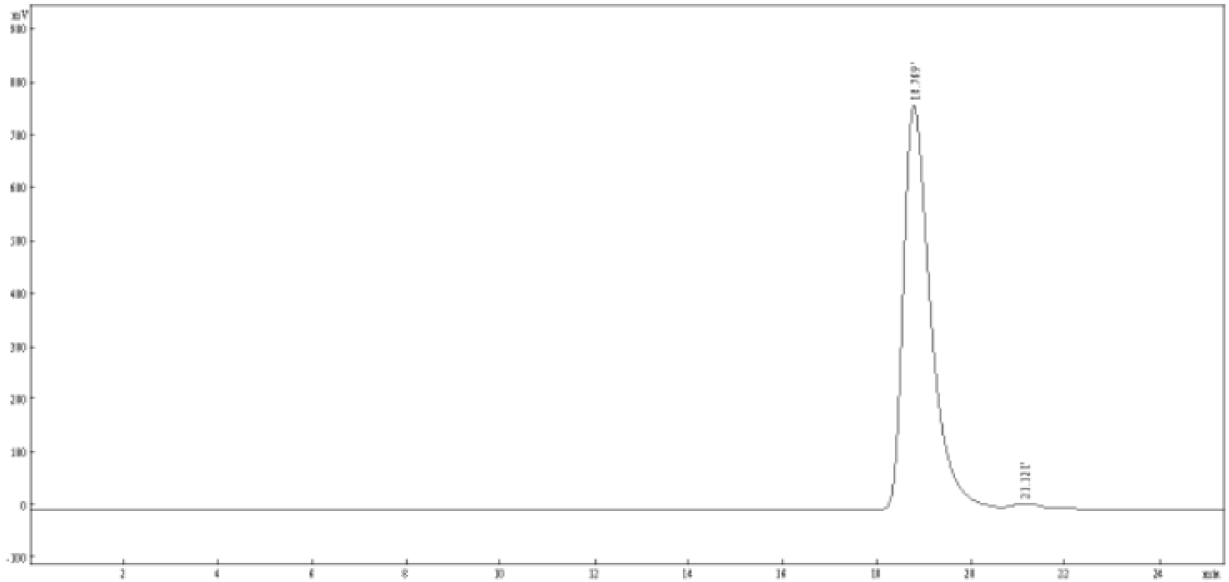

Peak#	Ret.Time	Area	Area %

1	18.769	30378630	99.23
2	21.121	236166	0.7714
Total		30614796	100

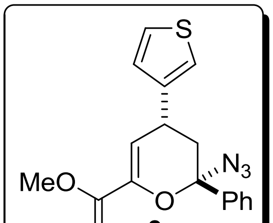

3w

Yellow oil; (58.6 mg, 86% yield); ^{1}H NMR (500 MHz, $CDCl_3$) δ 7.51-7.49 (m, 1H), 7.46-7.39 (m, 3H), 7.29 (dd, J = 5.0, 2.9 Hz, 1H), 7.04-7.04 (m, 1H), 6.96 (dd, J = 5.0, 1.2 Hz, 1H), 6.21 (dd, J = 2.9, 0.9 Hz, 1H), 3.87 (s, 3H), 3.42-3.89 (m, 1H), 2.67-2.63 (m, 1H), 2.25 (dd, J = 13.8, 9.6 Hz, 1H); ^{13}C NMR (125 MHz, $CDCl_3$) δ 162.5, 142.0, 141.9, 138.1, 129.3, 129.0, 126.8, 126.2, 125.2, 121.0, 114.8, 94.2, 52.4, 38.7, 32.0; IR (neat): 2951, 2107, 1732, 1433, 1256, 1108, 1028, 756, 697 cm^{-1}; HRMS exact mass calcd for $C_{17}H_{15}N_3NaO_3S$ [M + Na]$^+$ 364.0732, found 364.0741. $[\alpha]^{19}_{D}$ = 31.6 ° (c 1.24, $CHCl_3$); 97% ee; Chiral HPLC analysis of the product: Daicel Chiralcel OD-H 250X4.6 mm 5u column; hexane/2-propanol = 99/1, detected at 254 nm, Flow rate = 1 mL/min, Retention times: 16.0 min (major), 22.5 min (minor).

Supplementary Figure 26. HPLC spectra of 3w

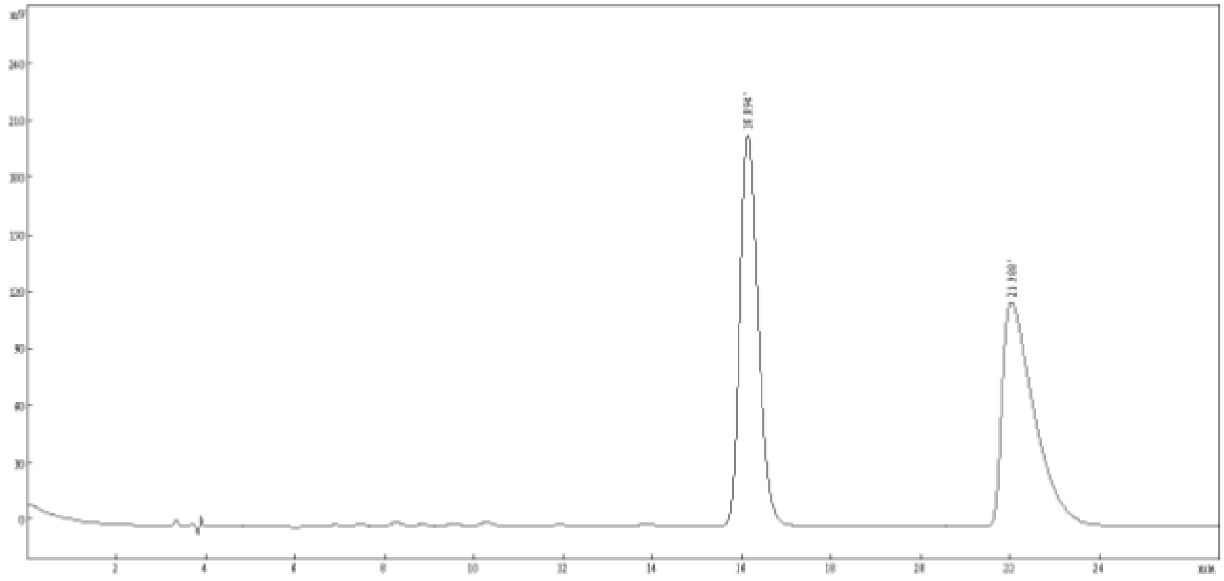

Peak#	Ret.Time	Area	Area %
1	16.094	5827797	49.95
2	21.988	5838574	50.05
Total		11666371	100

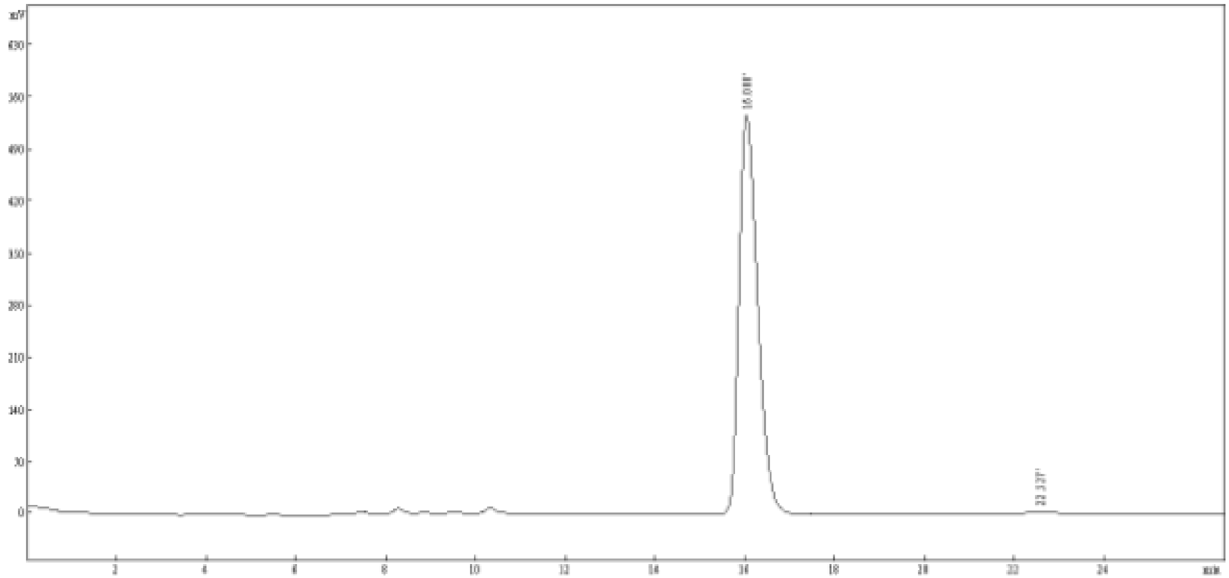

Peak#	Ret.Time	Area	Area %
1	16.000	15529902	98.68
2	22.527	206578	1.313
Total		15736480	100

Supplementary table 2: Optimization for the Diels-Alder reaction[a]

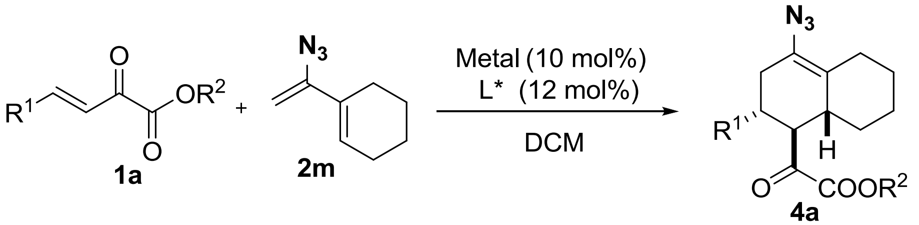

1a 2m 4a

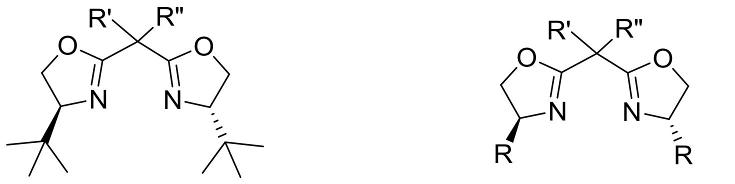

L3, R' = R" = Me
L10, R' = R" = $-CH_2CH_2-$
L11, R' = R" = $-(CH_2)_4-$
L12, R' = R" = $-(CH_2)_5-$

L8, R = ^{t}Bu, R' = Me, R" = 4-$^tBuC_6H_4CH_2$
L9, R = Ph, R' = Me, R" = 4-$^tBuC_6H_4CH_2$

**Entry**	**Metal**	**Ligand**	**T (°C)**	**Yield (%)[c]**	**ee (%)[d]**
1	$Cu(SbF_6)_2$	**L9**	30	84	21
2	$Cu(SbF_6)_2$	**L8**	30	64	73
3	$Cu(SbF_6)_2$	**L3**	30	75	80
4	$Cu(OTf)_2$	**L3**	30	85	77
5	$Cu(ClO_4)_2.6H_2O$	**L3**	30	81	78
6	$Cu(SbF_6)_2$	**L3**	0	80	84
7	$Cu(SbF_6)_2$	**L3**	-20	78	87
8	$Cu(SbF_6)_2$	**L3**	-40	72	87
9	$Cu(SbF_6)_2$	**L10**	-20	84	87
10	$Cu(SbF_6)_2$	**L11**	-20	79	93
11	$Cu(SbF_6)_2$	**L12**	-20	81	84

[a]**Reaction conditions**: Lewis acid (10 mol%), Ligand (12 mol%) and 4A° MS (100 mg) in 1 mL DCM were stirred at 30 °C for 3h, then the mixture of **1a** (0.2 mmol) in 1ml DCM at 30 °C, and **2m** (0.24 mmol) in 1 mL DCM at -20 °C were added to the reaction mixture. [c]Isolated yield. [d]Determined by HPLC using a chiral stationary phase.

Preparation and Characterization of Compound 4

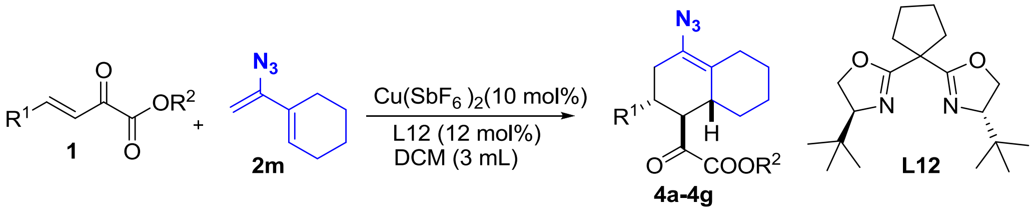

Supplementary Figure 27. Procedures for the preparation of compounds **4a-4g**

General Procedure for the *Cu(II)-BOX* catalyzed enantioselecetive [2+4]-cycloaddition of ketoesters 1 with azide 2m taking synthesis of 4a as an example:

A mixture of $CuBr_2$ (4.4 mg, 0.02 mmol, 0.1 eq), $AgSbF_6$ (13.7 mg, 0.04 mmol, 0.2 eq), ligand (**L12**, 7.6 mg, 0.024 mmol, 0.12 eq) and 100 mg 4A^o MS in DCM (1 mL) was stirred at 30 ^oC for 3 h under nitrogen. Then, to the catalyst solution a mixture of **1a** (38.0 mg, 0.2 mmol, 1 eq) in DCM (1 mL) at the room temperature followed by **2m** (35.7 mg, 0.24 mmol, 1.2 eq) in DCM (1 mL) at -20 ^oC were added. The resulting suspension was allowed to stir at -20 ^oC and monitored by TLC, until the complete consumption of **1** (usually 48 to 60 hours). Then, the mixture was filtered through a pad of celite and washed with DCM. The filterate was concentrated under reduced pressure and the residue was purified by column chromatography over silicagel using (ethyl acetate/petroleum ether, 1/50) to afford **4a** (53.5 mg, 79% yield) as a light yellow oil.

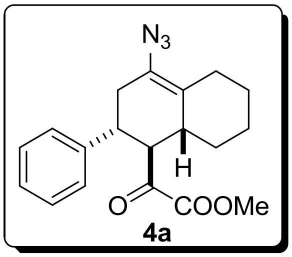

Light yellow oil; (53.5 mg, 79% yield); ^{1}H NMR (500 MHz, $CDCl_3$) δ 7.28 (d, J = 7.1 Hz, 2H), 7.22-7.18 (m, 3H), 3.60 (dd, J = 11.9, 9.7 Hz, 1H), 3.52 (s, 3H), 3.09 (td, J = 11.5, 5.1 Hz, 1H), 2.95-2.92 (m, 1H), 2.66-2.59 (m, 2H), 2.52-2.48 (m, 1H), 1.78-1.60 (m, 4H), 1.32-1.25 (m, 2H), 1.20-1.10 (m, 1H); ^{13}C NMR (125 MHz, $CDCl_3$) δ 198.3, 161.7, 140.5, 128.8, 127.9, 127.9, 125.4, 123.9, 55.0, 52.7, 43.8, 41.4, 33.6, 33.5, 27.2, 26.1, 25.5; IR (neat): 2949, 2107, 1731, 1647, 1448, 1254, 1113, 1042, 759, 698 cm^{-1}; HRMS exact mass calcd for $C_{19}H_{21}N_3NaO_3$ [M + Na]$^+$ 362.1481, found 362.1478. $[\alpha]^{16}_D$ = 4.0 ° (c 4.35, $CHCl_3$); 93% ee; Chiral HPLC analysis of the product: Phenomenex 00G-4457-E0 250X4.6 mm 5u column; hexane/2-propanol = 98/2, detected at 254 nm, Flow rate = 1 mL/min, Retention times: 11.0 min (major), 13.0 min (minor).

Supplementary Figure 28. HPLC spectra of 4a

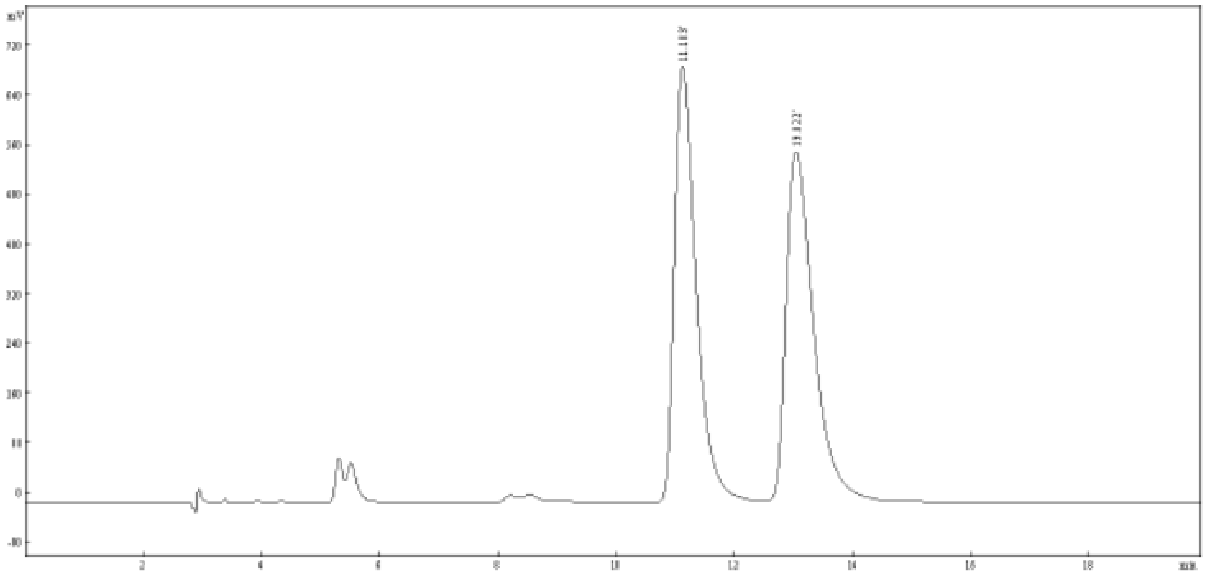

Peak#	Ret.Time	Area	Area %
1	11.103	17910171	49.68
2	13.022	18141214	50.32
Total		36051385	100

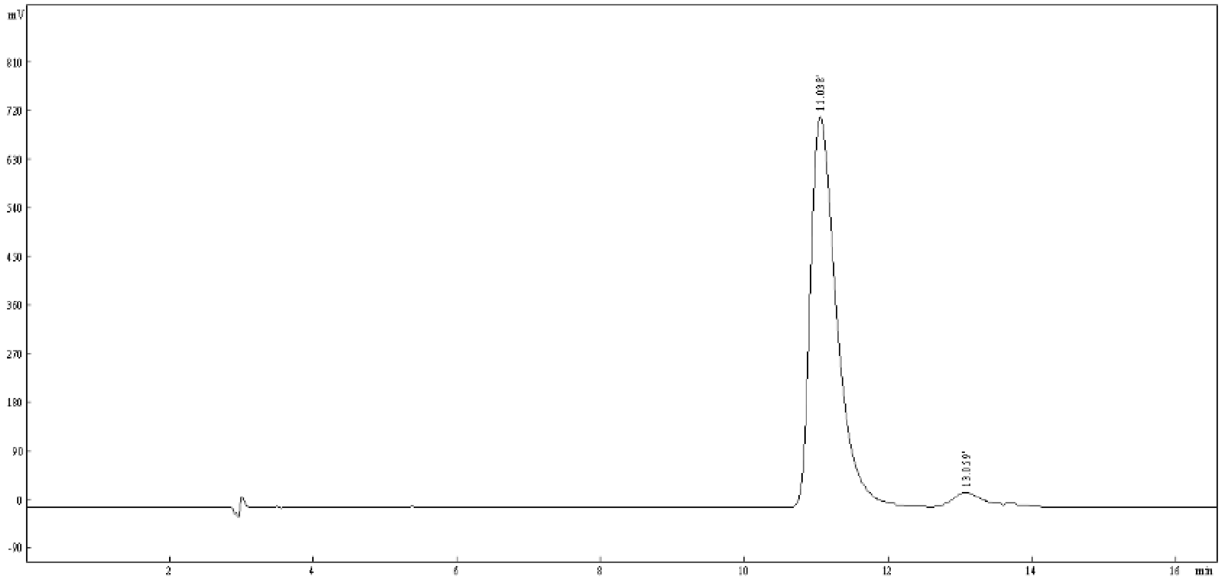

Peak#	Ret.Time	Area	Area %

1	11.038	18218062	96.34
2	13.059	690795	3.653
Total		18908857	100

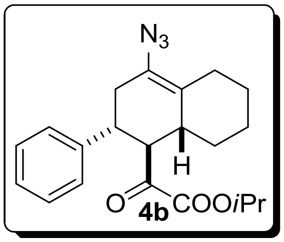

Light yellow oil; (64.5 mg, 88% yield); ^{1}H NMR (500 MHz, $CDCl_3$) δ 7.23-7.18 (m, 2H), 7.14-7.11 (m, 3H), 4.75-4.70 (m, 1H), 3.54 (dd, J = 11.8, 9.8 Hz, 1H), 3.02 (td, J = 11.5, 5.1 Hz, 1H), 2.87-2.84 (m, 1H), 2.57-2.50 (m, 2H), 2.44-2.40 (m, 1H), 1.70-1.51 (m, 4H), 1.30-1.18 (m, 3H), 1.09 (d, J = 6.2 Hz, 3H), 0.95 (d, J = 6.2 Hz, 3H); ^{13}C NMR (125 MHz, $CDCl_3$) δ 198.0, 159.7, 139.7, 127.8, 126.9, 126.3, 124.5, 122.8, 69.4, 53.6, 42.7, 40.6, 32.6, 32.6, 26.2, 25.1, 24.5, 20.3; IR (neat): 2922, 2096, 1714, 1260, 1102, 1055, 753, 700 cm^{-1}; HRMS exact mass calcd for $C_{21}H_{25}N_3NaO_3$ [M + Na]$^+$ 390.1794, found 390.1782. $[\alpha]^{16}_{D}$ = 0.4 ° (c 2.55, $CHCl_3$); 89% ee; Chiral HPLC analysis of the product: Daicel Chiralcel OD-H 250X4.6 mm 5u column; hexane/2-propanol = 99/1, detected at 254 nm, Flow rate = 1 mL/min, Retention times: 10.2 min (minor), 57.2 min (major).

Supplementary Figure 29. HPLC spectra of 4b

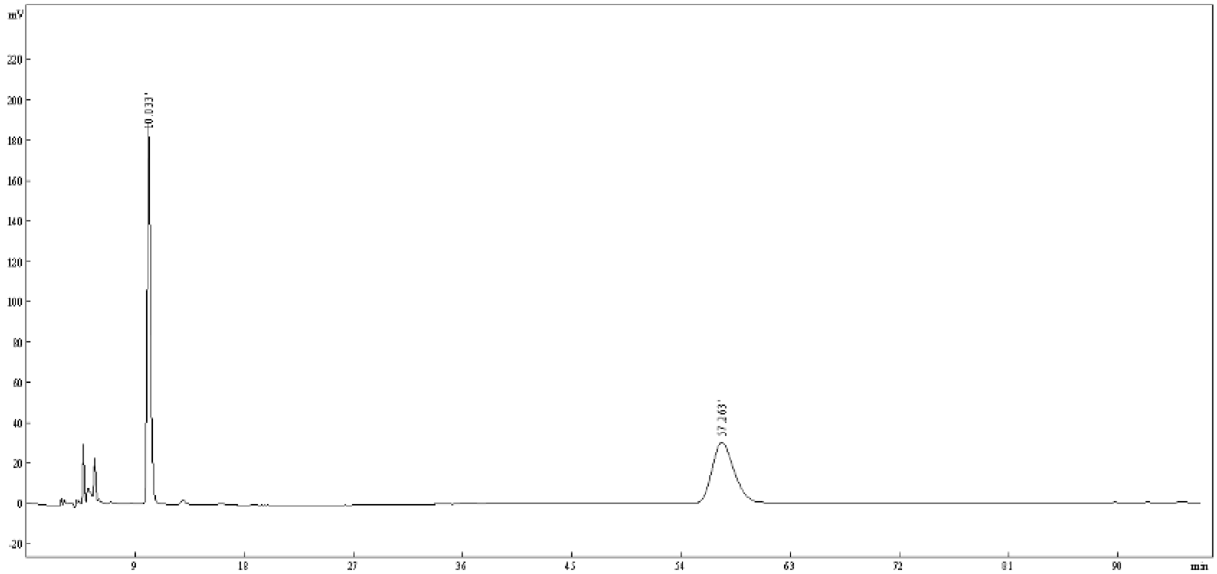

Peak#	Ret.Time	Area	Area %
1	10.033	3910718	50.3
2	57.263	3864564	49.7
Total		7775282	100

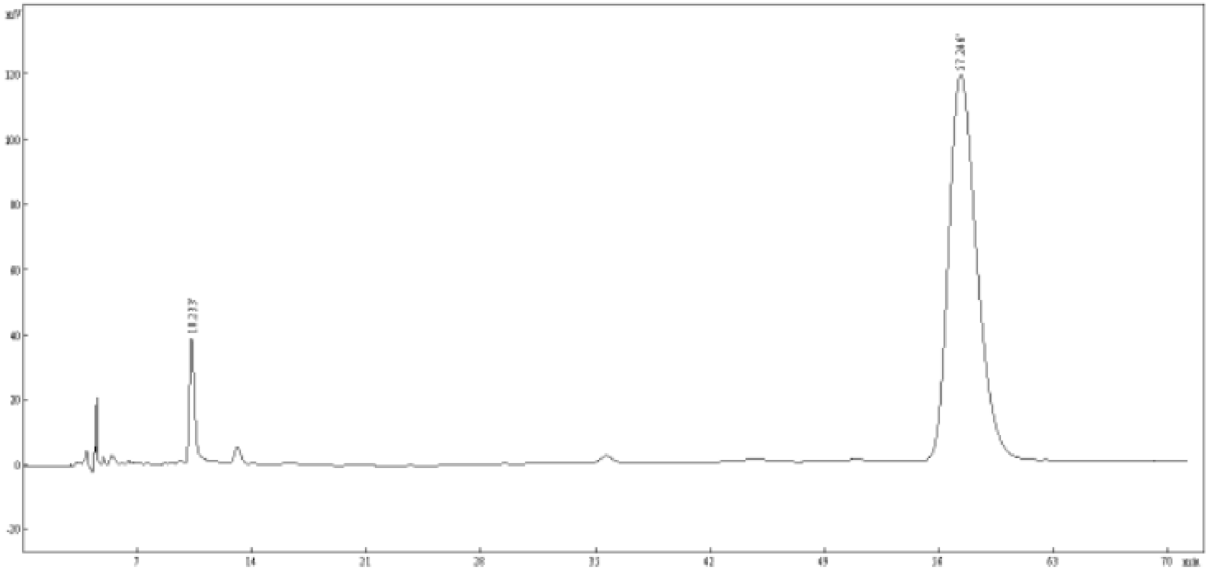

Peak#	Ret.Time	Area	Area %
1	10.233	825673	5.538
2	57.246	14082858	94.46
Total		14908531	100

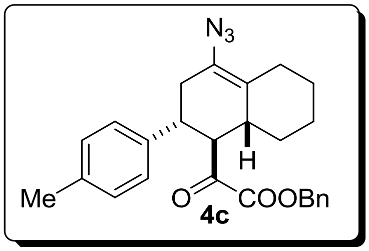

Light yellow oil; (70.3 mg, 82% yield); ^{1}H NMR (500 MHz, $CDCl_3$) δ 7.35-7.33 (m, 3H), 7.23-7.18 (m, 2H), 7.06-6.98 (m, 4H), 4.96 (dd, J = 33.0, 15.2 Hz, 2H), 3.57 (dd, J = 14.7, 12.4 Hz, 1H), 3.12-3.05 (m, 1H), 2.94-2.91 (m, 1H), 2.65-2.40 (m, 3H), 2.30-2.23 (m, 1H), 2.26 (s, 3H),

1.78-1.58 (m, 4H), 1.24-1.06 (m, 3H); ^{13}C NMR (125 MHz, $CDCl_3$) δ 197.3, 160.0, 136.5, 136.0, 133.2, 128.4, 127.5, 127.5, 127.3, 126.6, 124.3, 122.9, 66.6, 54.1, 42.2, 40.5, 32.7, 32.6, 26.2, 25.0, 24.4, 20.0; IR (neat): 2931, 2098, 1726, 1260, 1046, 747, 696 cm^{-1}; HRMS exact mass calcd for $C_{26}H_{27}N_3NaO_3$ $[M + Na]^+$ 452.1950, found 452.1983. $[\alpha]^{16}_D$ = 1.1 ° (c 2.48, $CHCl_3$); 85% ee; Chiral HPLC analysis of the product: Phenomenex 00G-4457-E0 250X4.6 mm 5u column; hexane/2-propanol = 99.4/0.6, detected at 254 nm, Flow rate = 1 mL/min, Retention times: 13.4 min (major), 16.5 min (minor).

Supplementary Figure 30. HPLC spectra of 4c

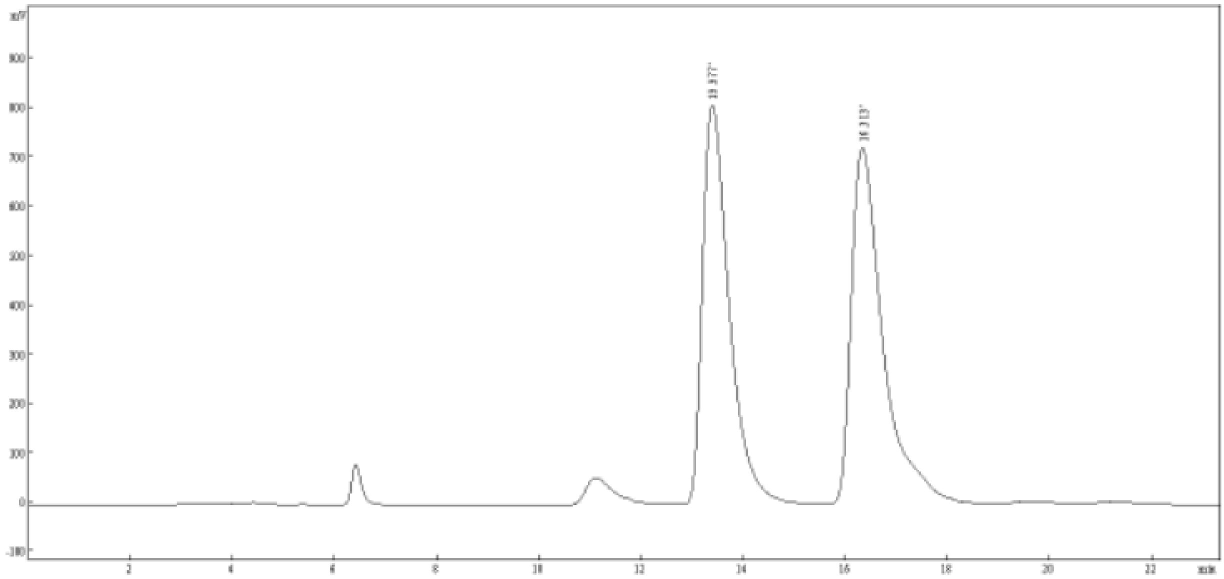

Peak#	Ret.Time	Area	Area %
1	13.377	29388960	48.73
2	16.313	30916230	51.27
Total		60305190	100

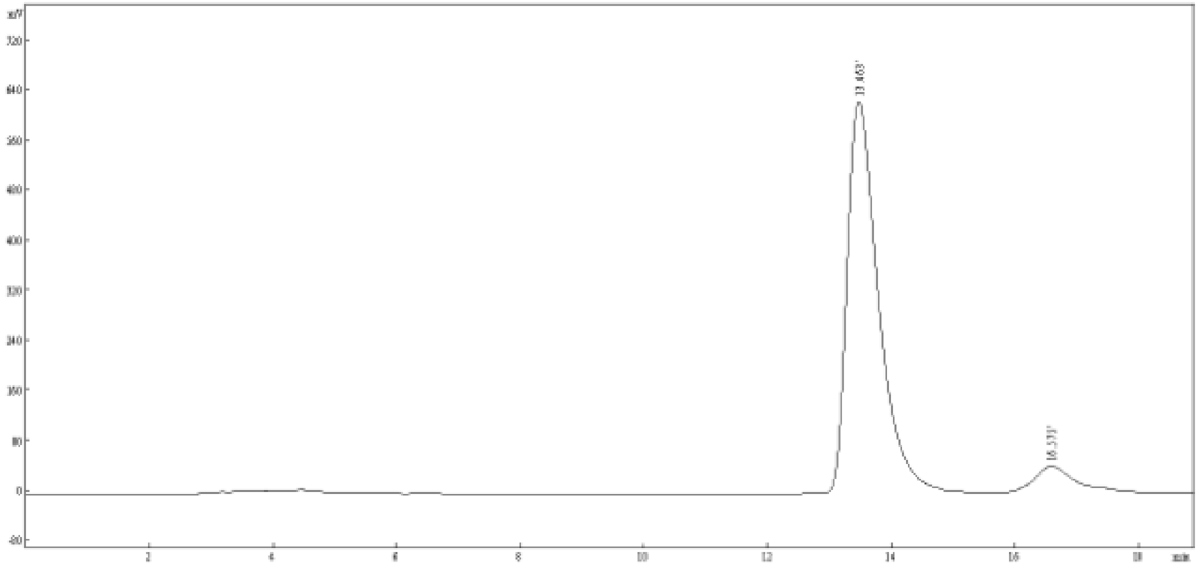

Peak#	Ret.Time	Area	Area %
1	13.463	22193594	92.39
2	16.575	1828682	7.612
Total		24022276	100

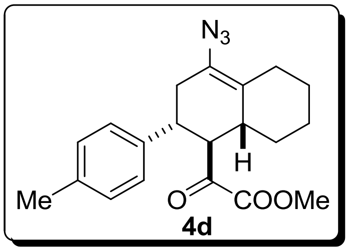

Colorless liquid; (59.3 mg. 84% yield); ^{1}H NMR (500 MHz, $CDCl_3$) δ 7.09-7.06 (m, 4H), 3.59-3.54 (m, 1H), 3.54 (s, 3H), 3.07 (td, J = 11.6, 5.1 Hz, 1H), 2.94-2.91 (m, 1H), 2.64-2.56 (m, 2H), 2.50-2.45 (m, 1H), 1.77-1.59 (m, 4H), 1.32-1.09 (m, 3H); ^{13}C NMR (125 MHz, $CDCl_3$) δ 198.3, 161.6, 137.4, 137.1, 129.4, 127.8, 125.3, 123.9, 55.1, 52.6, 43.3, 41.4, 33.7, 33.6, 27.2, 26.1, 25.5, 20.9; IR (neat): 2925, 2849, 2098, 1723, 1433, 1266, 1058, 821, 750 cm^{-1}; HRMS exact mass calcd for $C_{20}H_{23}N_3NaO_3$ [M + Na]$^+$ 376.1637, found 376.1640. $[\alpha]^{16}_{D}$ = 6.5 ° (c 1.96, $CHCl_3$); 91% ee; Chiral HPLC analysis of the product: Phenomenex 00G-4457-E0 250X4.6 mm 5u column; hexane/2-propanol = 99.4/0.6, detected at 254 nm, Flow rate = 1 mL/min, Retention times: 12.1 min (major), 16.7 min (minor).

Supplementary Figure 31. HPLC spectra of 4d

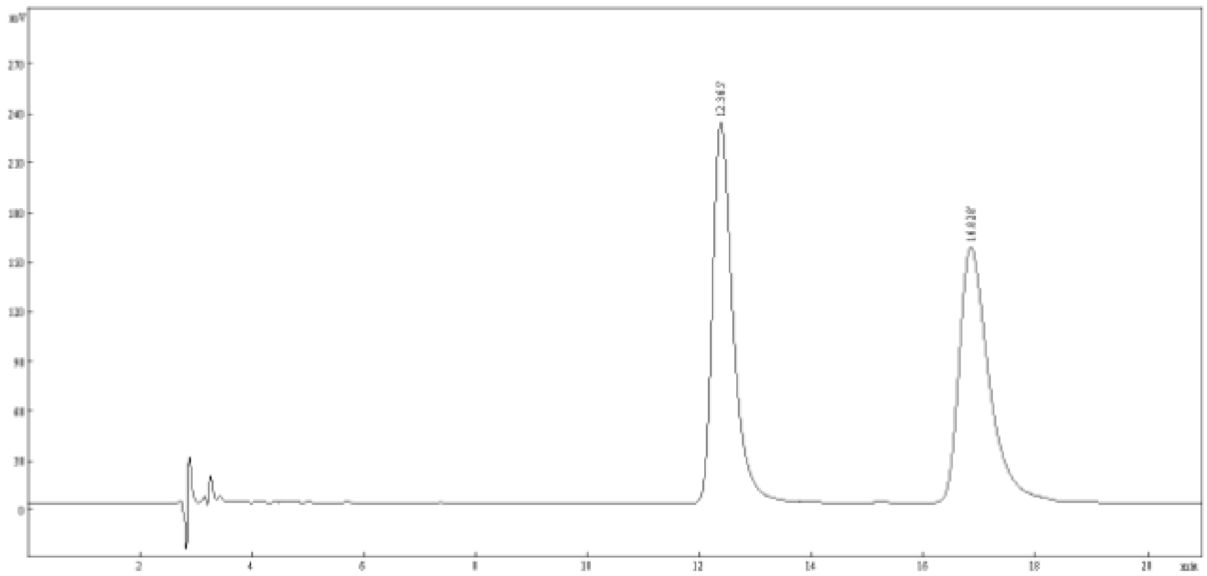

Peak#	Ret.Time	Area	Area %
1	12.365	5874450	50.45
2	16.828	5770320	49.55
Total		11644770	100

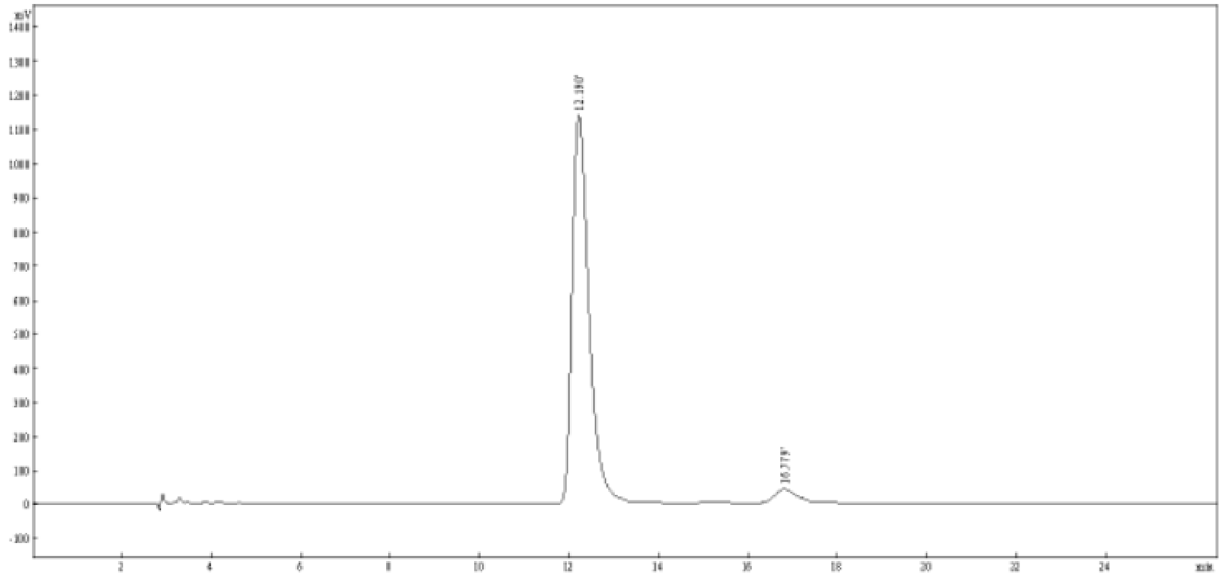

Peak#	Ret.Time	Area	Area %
1	12.190	29687110	95.63
2	16.779	1358641	4.376
Total		31045751	100

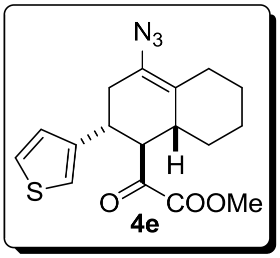

Yellow oil; (54.5 mg, 79% yield); ^{1}H NMR (500 MHz, $CDCl_3$) δ 7.26 (t, J = 4.2 Hz, 2H), 7.01 (dd, J = 3.6, 1.5 Hz, 1H), 6.95 (dd, J = 7.2, 1.5 Hz, 1H), 3.66-3.52 (m, 1H), 3.61 (s, 3H), 3.26 (td, J = 14.2, 6.4 Hz, 1H), 2.94-2.90 (m, 1H), 2.66-2.58 (m, 2H), 2.54-2.48 (m, 1H), 1.78-1.61 (m, 4H), 1.25-1.09 (m, 3H); ^{13}C NMR (125 MHz, $CDCl_3$) δ 198.2, 141.3, 126.6, 126.4, 125.5, 123.6, 122.2, 55.1, 52.8, 41.1, 39.0, 33.6, 33.2, 27.2, 26.1, 25.5; IR (neat): 2931, 2852, 2093, 1720, 1263, 1061, 1058, 782, 738, 654 cm^{-1}; HRMS exact mass calcd for $C_{17}H_{19}N_3NaO_3S$ [M + Na]$^+$ 368.1045, found 368.1017. $[\alpha]^{16}_D$ = 12.2 °(c 1.09, $CHCl_3$); 85% ee; Chiral HPLC analysis of the product: Daicel Chiralcel OD-H 250X4.6 mm 5u column; hexane/2-propanol = 98/2, detected at 254 nm, Flow rate = 1 mL/min, Retention times: 11.4 min (minor), 25.7 min (major).

Supplementary Figure 32. HPLC spectra of 4e

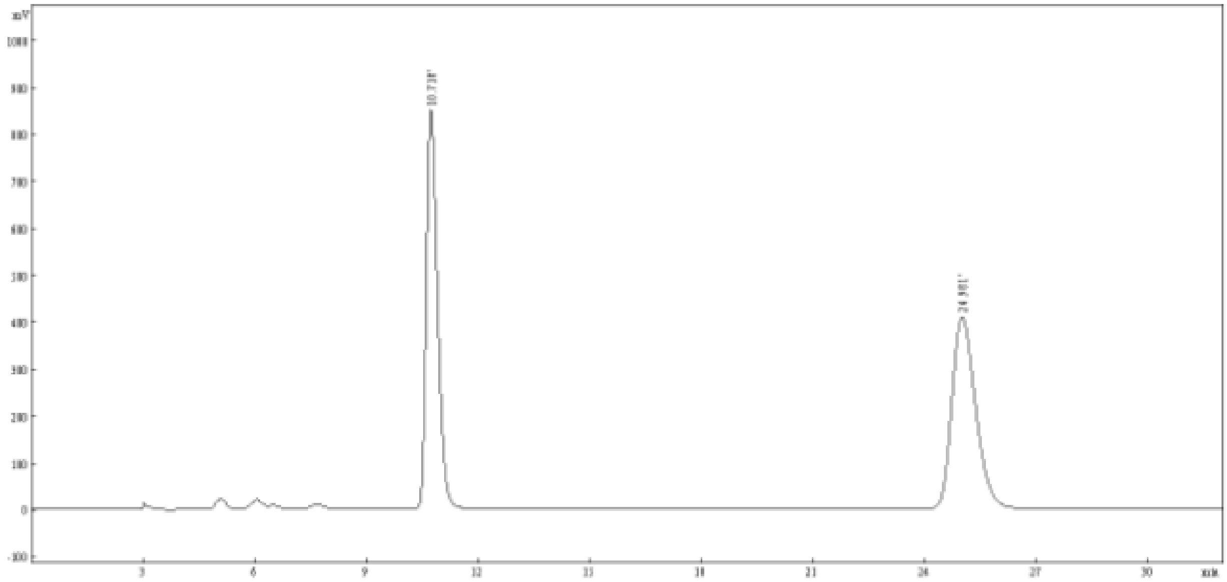

Peak#	Ret.Time	Area	Area %
1	10.718	17602248	49.63
2	24.981	17862517	50.37
Total		35464765	100

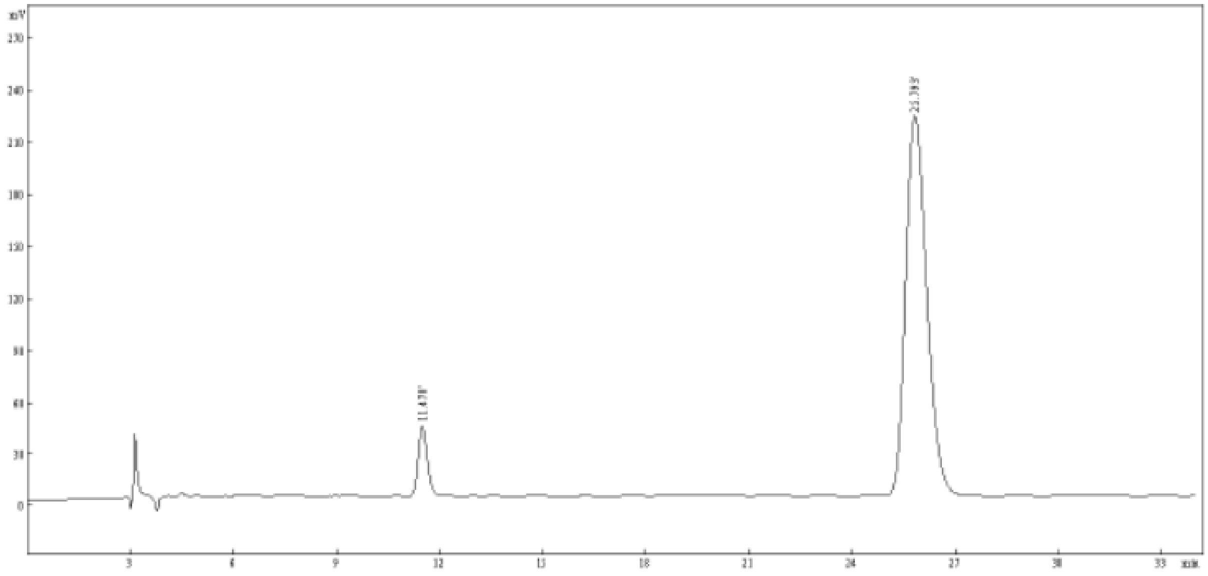

Peak#	Ret.Time	Area	Area %

1	11.470	757660	7.429
2	25.793	9441051	92.57
Total		10198711	100

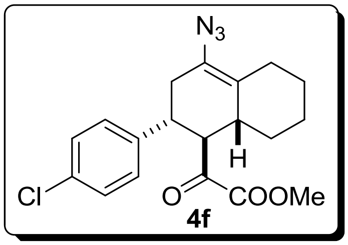

Light yellow oil; (48.4 mg, 65% yield); ^{1}H NMR (500 MHz, $CDCl_3$) δ 7.26-7.24 (m, 2H), 7.14-7.12 (m, 2H), 3.62-3.54 (m, 1H), 3.60 (s, 3H), 3.11 (td, J = 14.3, 6.7 Hz, 1H), 2.94-2.91 (m, 1H), 2.62-2.53 (m, 2H), 2.50-2.45 (m, 1H), 1.78-1.57 (m, 4H), 1.32-1.10 (m, 3H); ^{13}C NMR (125 MHz, $CDCl_3$) δ 198.0, 161.6, 139.1, 133.2, 129.2, 128.9, 125.4, 123.7, 54.8, 52.8, 43.0, 41.6, 33.6, 33.4, 27.2, 26.0, 25.5; IR (neat): 2919, 2101, 1729, 1275, 1049, 747, 690 cm^{-1}; HRMS exact mass calcd for $C_{19}H_{20}ClN_3NaO_3$ $[M + Na]^+$ 396.1091, found 396.1094. $[\alpha]^{17}_{D}$ = 3.8 °(c 0.99, $CHCl_3$); 82% ee; Chiral HPLC analysis of the product: Phenomenex 00G-4457-E0 250X4.6 mm 5u column; hexane/2-propanol = 99.3/0.7, detected at 254 nm, Flow rate = 1 mL/min, Retention times: 10.5 min (major), 14.1 min (minor).

Supplementary Figure 33. HPLC spectra of 4f

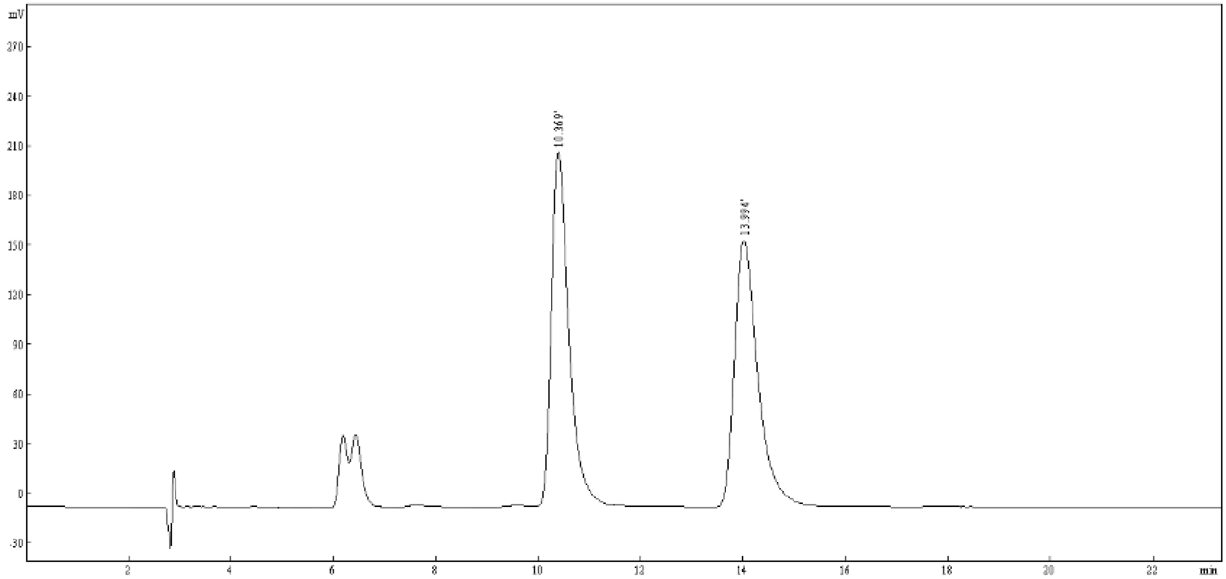

Peak#	Ret.Time	Area	Area %
1	10.369	5130831	49.56
2	13.994	5221999	50.44
Total		10352830	100

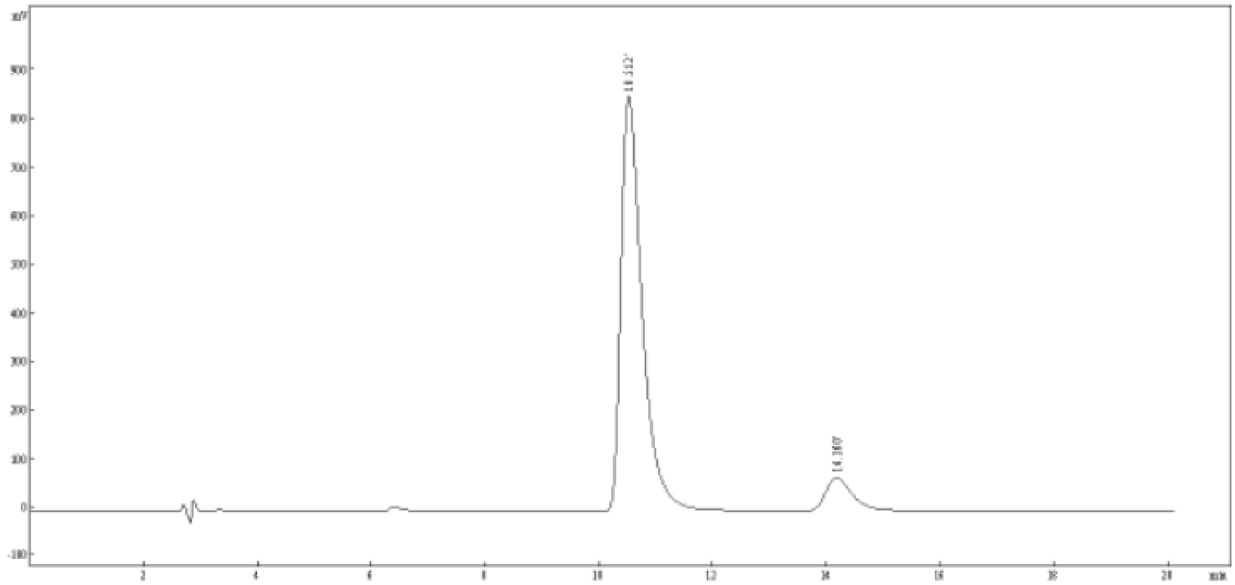

Peak#	Ret.Time	Area	Area %
1	10.512	22303814	91.12
2	14.160	2151742	8.799
Total		24455556	100

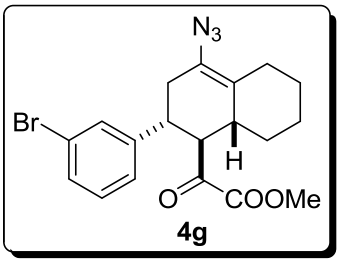

4g

Light yellow oil; (50.8 mg, 61% yield); ^{1}H NMR (500 MHz, $CDCl_3$) δ 7.36-7.34 (m, 2H), 7.17-7.10 (m, 2H), 3.64 (s, 3H), 3.63-3.55 (m, 1H), 3.13-3.06 (m, 1H), 2.94-2.91 (m, 1H), 2.62-2.53 (m, 2H), 2.54-2.46 (m, 1H), 1.78-1.58 (m, 4H), 1.32-1.10 (m, 3H); ^{13}C NMR (125 MHz, $CDCl_3$)

δ 198.0, 161.6, 143.0, 130.9, 130.6, 130.4, 126.6, 125.4, 123.6, 122.8, 54.7, 52.9, 43.3, 41.6, 33.6, 33.5, 27.2, 26.0, 25.5; IR (neat): 2928, 2101, 1726, 1430, 1260, 1061, 753, 695 cm^{-1}; HRMS exact mass calcd for $C_{19}H_{20}BrN_3NaO_3$ $[M + Na]^+$ 440.0586, found 440.0594. $[\alpha]^{17}_D$ = -0.9 ° (c 1.06, $CHCl_3$); 82% ee; Chiral HPLC analysis of the product: Phenomenex 00G-4457-E0 250X4.6 mm 5u column; hexane/2-propanol = 99.3/0.7, detected at 254 nm, Flow rate = 1 mL/min, Retention times: 11.7 min (major), 14.2 min (minor).

Supplementary Figure 34. HPLC spectra of 4g

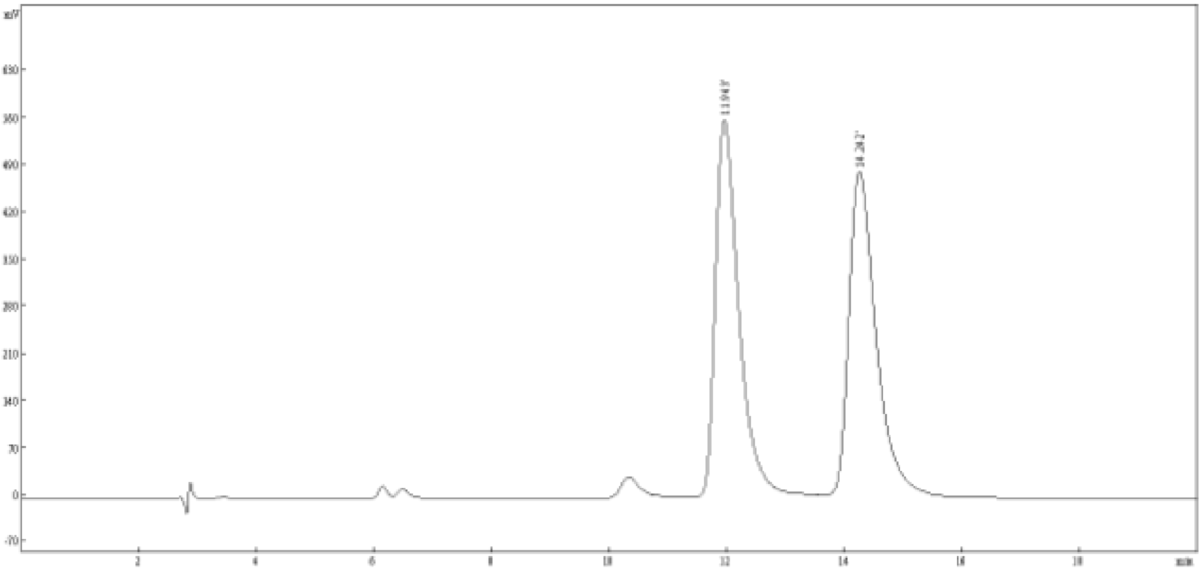

Peak#	Ret.Time	Area	Area %
1	11.943	15401205	50.03
2	14.242	15382797	49.97
Total		30784002	100

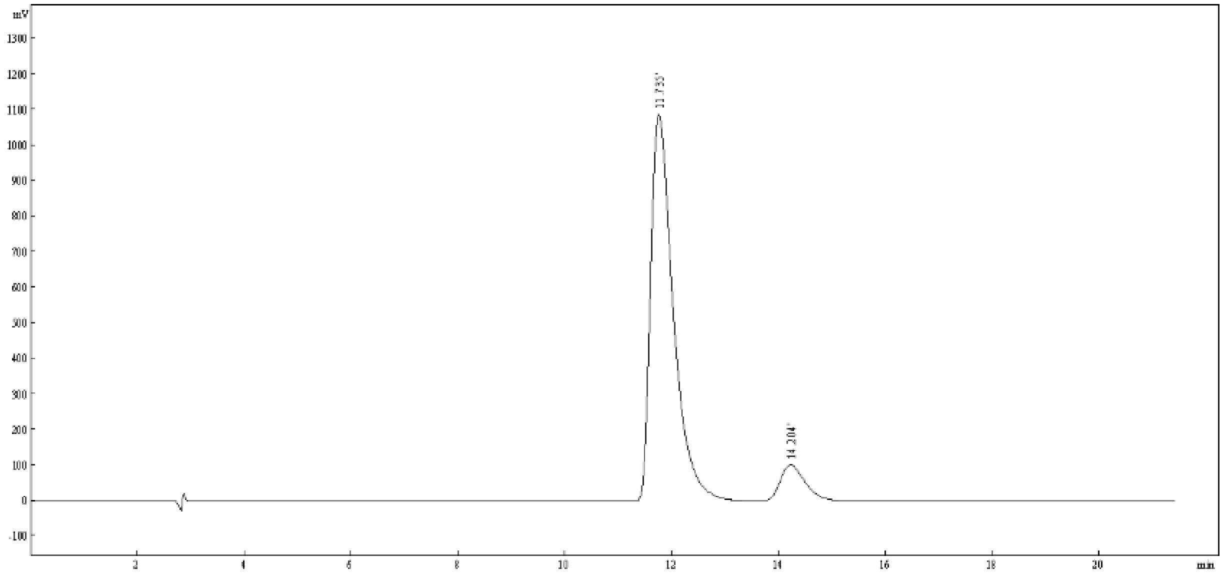

Peak#	Ret.Time	Area	Area %
1	11.735	32336611	90.76
2	14.204	3290030	9.234
Total		35626641	100

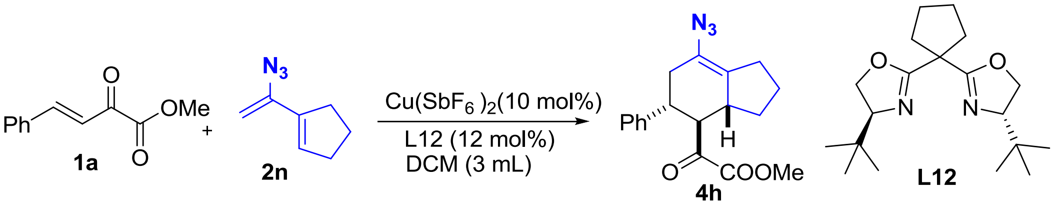

Supplementary Figure 35. Procedures for the preparation compounds **4h**

Procedure for the *Cu(II)-BOX* catalyzed enantioselecetive [2+4]-cycloaddition of ketoester 1 with azide 2n:

A mixture of $CuBr_2$ (4.4 mg, 0.02 mmol, 0.1 eq), $AgSbF_6$ (13.7 mg, 0.04 mmol, 0.2 eq), ligand (**L12**, 7.6 mg, 0.024 mmol, 0.12 eq) and 100 mg 4A^o MS in DCM (1 mL) was stirred at 30 ^{o}C for 3 h under nitrogen. Then, to the catalyst solution a mixture of **1a** (38.0 mg, 0.2 mmol, 1 eq) in DCM (1 mL) at the room temperature followed by **2n** (32.4 mg, 0.24 mmol, 1.2 eq) in DCM (1 mL) at -30 ^{o}C were added. The resulting suspension was allowed to stir at -30 ^{o}C and monitored by TLC, until the complete consumption of **1** (60 hours). Then, the mixture was filtered through a pad of celite and washed with DCM. The filterate was concentrated under reduced pressure and

the residue was purified by column chromatography over silicagel using (ethyl acetate/petroleum ether, 1/50) to afford **4a** (56.5 mg, 87% yield) as a yellow color gum.

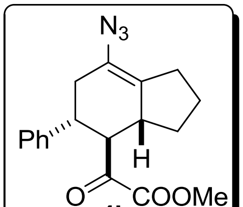

4h

Yellow color gum; (56.5 mg, 87% yield); ^{1}H NMR (500 MHz, $CDCl_3$) δ 7.22-7.18 (m, 2H), 7.15-7.12 (m, 3H), 3.52 (s, 3H), 3.47 (dd, J = 11.6, 10.3 Hz, 1H), 3.09 (td, J = 11.3, 6.0 Hz, 1H), 2.68-2.55 (m, 2H), 2.48-2.40 (m, 1H), 2.27-2.24 (m, 2H), 1.81-1.74 (m, 2H), 1.53-1.47 (m, 1H), 1.14-1.11 (m, 1H); ^{13}C NMR (125 MHz, $CDCl_3$) δ 197.2, 161.6, 141.0, 129.3, 128.8, 127.8, 127.4, 123.6, 53.1, 52.7, 45.8, 44.9, 34.6, 31.9, 26.6, 23.9; IR (neat): 2943, 2098, 1729, 1454, 1263, 1058, 764, 700 cm^{-1}; HRMS exact mass calcd for $C_{18}H_{19}N_3NaO_3$ [M + Na]$^+$ 348.1324, found 348.1325. $[\alpha]^{26}_{D}$ = 87.5 ° (c 1.79, $CHCl_3$); 88% ee; Chiral HPLC analysis of the product: Phenomenex 00G-4457-E0 250X4.6 mm 5u column; hexane/2-propanol = 98/2, detected at 254 nm, Flow rate = 1 mL/min, Retention times: 11.1 min (minor), 15.0 min (major).

Supplementary Figure 36. HPLC spectra of 4h

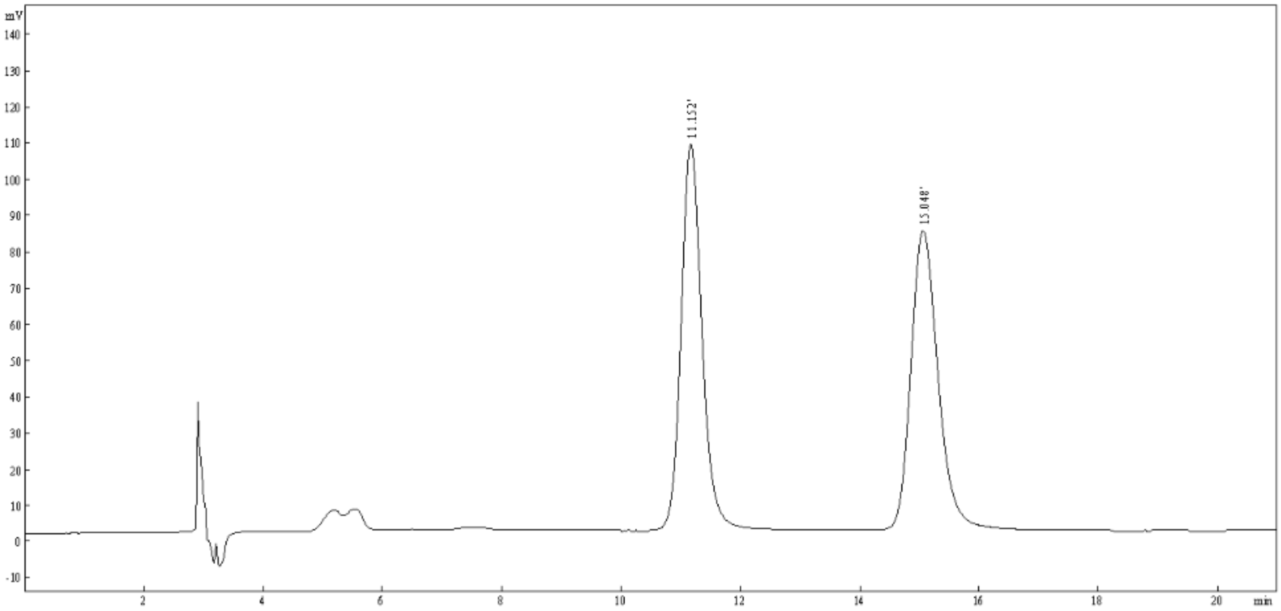

Peak#	Ret.Time	Area	Area %
1	11.152	2605946	49.76

2	15.048	2630822	50.24
Total		5236768	100

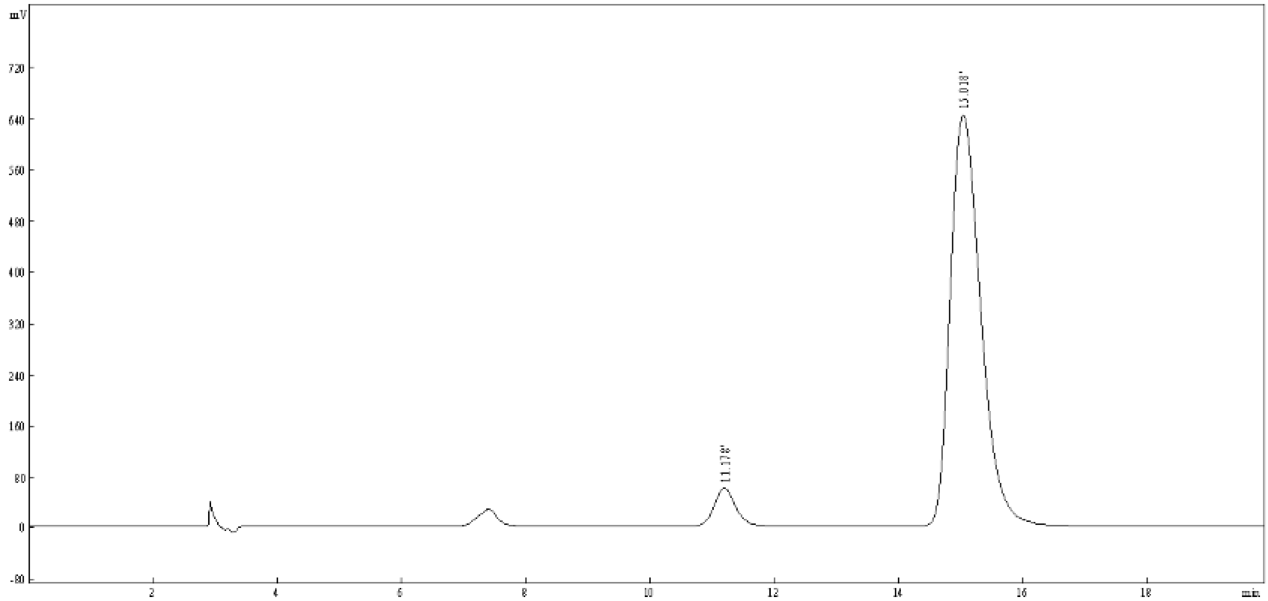

Peak#	Ret.Time	Area	Area %
1	11.178	1453858	6.235
2	15.018	21863942	93.76
Total		23317800	100

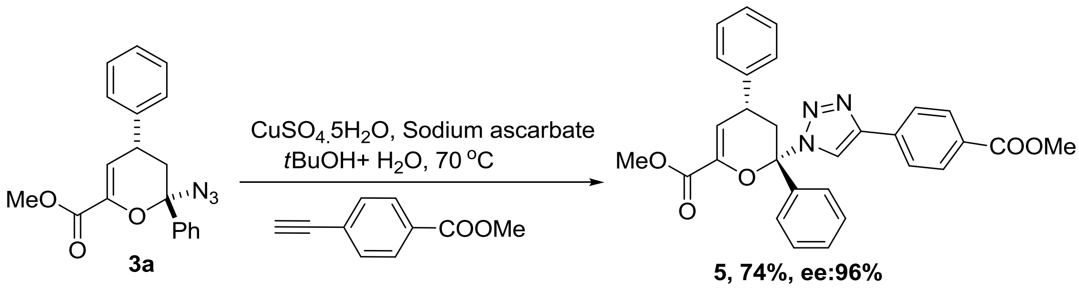

Supplementary Figure 37. Procedure for the preparation compound **5**

Preparation and Characterization of Compound 5:

To the mixture of **3a** (67.0 mg, 0.2 mmol, 1 eq), $CuSO_4$ $5H_2O$ (9.9 mg, 0.04 mmol, 0.2 eq), and Sodium ascarbate (15.8 mg, 0.08 mmol, 0.4 eq) in 2 mL (*t*-BuOH+H_2O, 1:1) was added alkyne (38.4 mg, 0.24 mmol, 1.2 eq) at room temperature. Then, the reaction mixture was stirred at 70 ^{o}C for 12 hours. After cool down to the room temperatute, it was concentrated, diluted with 5 mL of water and extracted with ethyl acetate (3X10 mL). The combined organic layers were dried on Na_2SO_4, concentrated under reduced pressure, purified by column chromatography (silica gel, Petroleum ether/ EtOAc: 6.6/1) to get the desired product **5** (73.2 mg**,** 74% yield) as a white soild**.**

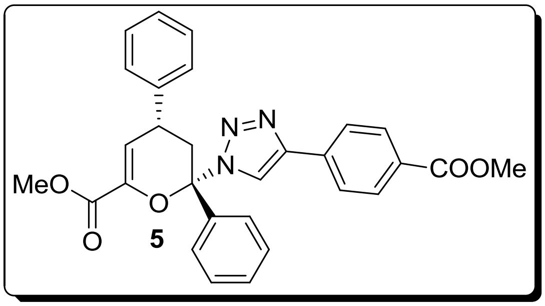

White solid; (73.2 mg, 74% yield); ^{1}H NMR (500 MHz, $CDCl_3$) δ 8.05-8.04 (m, 2H), 7.87 (s, 1H), 7.85-7.84 (m, 2H), 7.61-7.59 (m, 2H), 7.49-7.46 (m, 3H), 7.34-7.31 (m, 2H), 7.29-7.27 (m, 1H), 7.21-7.19 (m, 2H), 6.28 (dd, *J* = 2.2, 1.4 Hz, 1H) 3.91 (s, 3H), 3.90 (s, 3H), 3.50-3.46 (m, 1H), 3.36-3.33 (m, 1H), 3.14 (dd, *J* = 13.7, 11.4 Hz, 1H); ^{13}C NMR (125 MHz, $CDCl_3$) δ 166.7, 162.1, 146.5, 141.9, 141.0, 137.8, 134.6, 130.1, 129.9, 129.6, 129.4, 128.9, 127.4, 125.5, 125.3, 119.6, 116.3, 92.6, 52.6, 52.1, 38.7, 36.5; IR (neat): 2946, 2107, 1732, 1653, 1489, 1436, 1295, 1234, 1113, 1008, 759, 703 cm^{-1}; HRMS exact mass calcd for $C_{29}H_{25}N_3NaO_5$ $[M + Na]^+$ 518.1692, found 518.1686. $[\alpha]^{19}_{D}$ = -31.7 °(c 0.41, $CHCl_3$); 96% ee; Chiral HPLC analysis of the product: Daicel Chiralcel OD-H 250X4.6 mm 5u column; hexane/2-propanol = 70/30, detected at 254 nm, Flow rate = 1 mL/min, Retention times: 11.1 min (major), 27.7 min (minor).

Supplementary Figure 38. HPLC spectra of 5

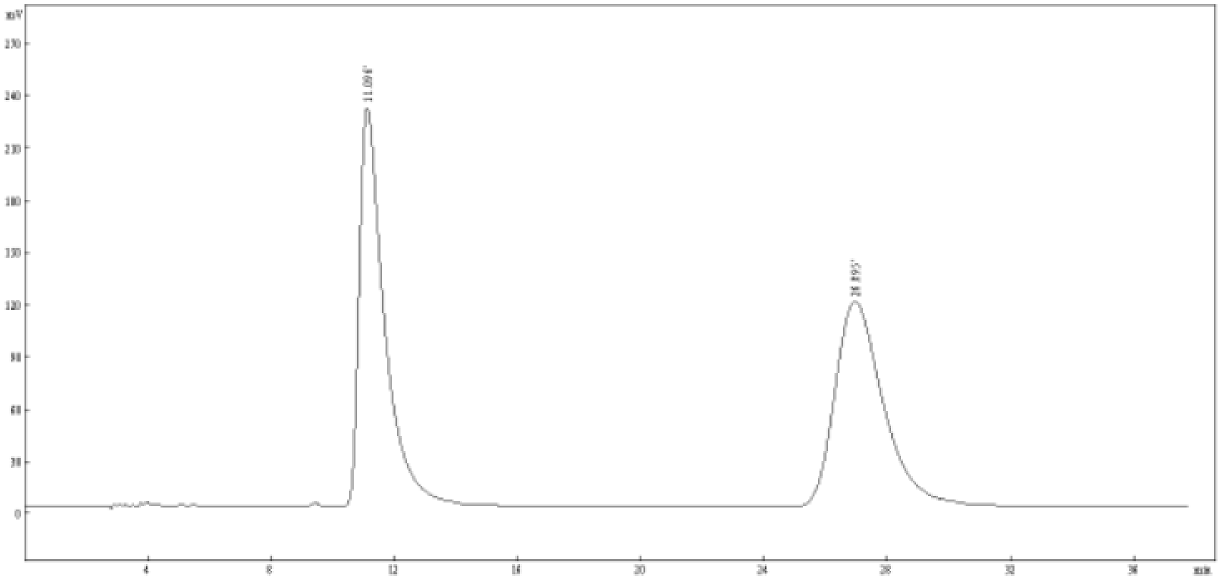

Peak#	Ret.Time	Area	Area %
1	11.096	13047258	49.78
2	26.895	13162734	50.22
Total		26209992	100

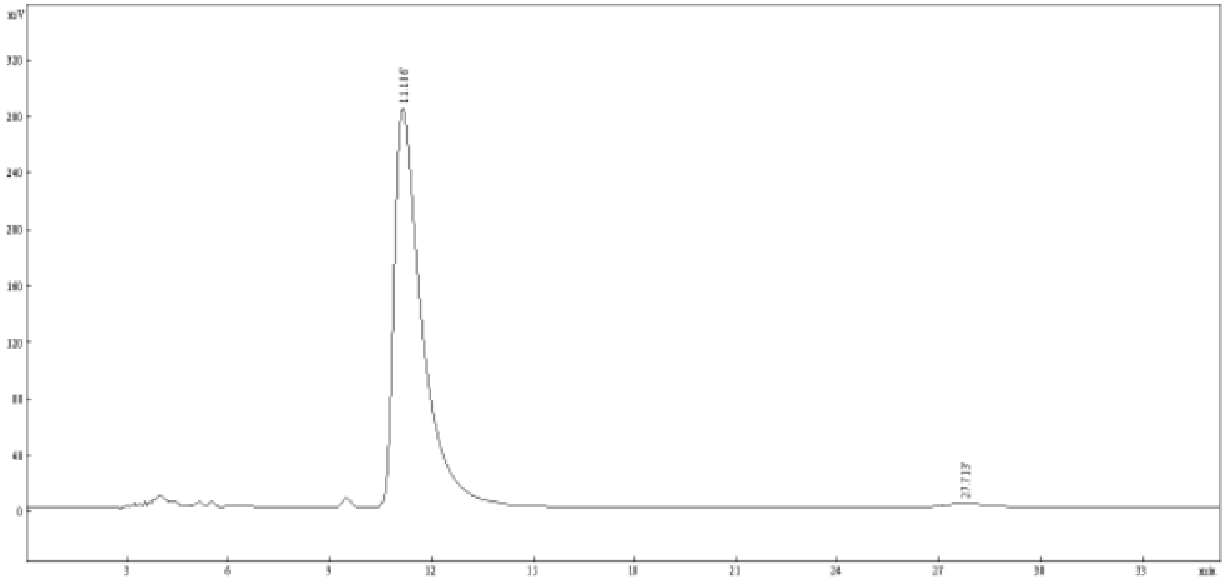

Peak#	Ret.Time	Area	Area %

1	11.106	16226754	97.79
2	27.713	367838	2.217
Total		16594592	100

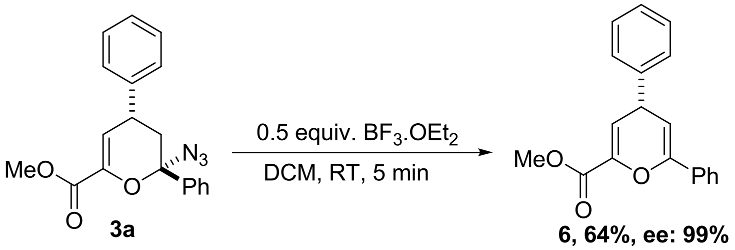

Supplementary Figure 39. Procedure for the preparation compound **6**

To a solution of **3a** (67.0 mg, 0.2 mmol, 1 eq) and 2 mL of DCM, $BF_3.OEt_2$ (46.5 %) was (30 µL, 0.1 mmol, 0.5 eq) added at 0 °C. After 5 min of stirring, the reaction mixture was diluted with 5 mL of water and extracted with DCM (2X10 mL). The combined organic layers were washed with brine, dried over Na_2SO_4, concentrated under reduced pressure. Crude was purified by column chromatography (silica gel, EtOAc/Petroleum ether: 1/50) to obtain the desired product **6** **(**37.3 mg, 64% yield) as a colorless oil**.**

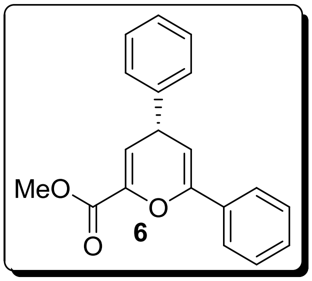

Colorless oil; (37.3 mg, 64% yield); ^{1}H NMR (500 MHz, $CDCl_3$) δ 7.67-7.66 (m, 2H), 7.38-7.25 (m, 8H), 6.17 (dd, J = 4.0, 2.2 Hz, 1H), 5.43 (dd, J = 3.6, 2.2 Hz, 1H), 4.36 (t, J = 4.0 hz, 1H), 3.85 (s, 3H); ^{13}C NMR (125 MHz, $CDCl_3$) δ 162.3, 148.3, 144.7, 140.6, 133.4, 128.8, 128.7, 128.3, 128.0, 127.1, 124.6, 113.4, 99.3, 52.3, 38.4; IR (neat): 2925, 2846, 1465, 1377, 1275, 1260, 764, 749 cm^{-1}; HRMS exact mass calcd for $C_{19}H_{16}NaO_3$ [M + Na]$^+$ 315.0997, found 315.1001. $[\alpha]^{19}_D$ = 70.5 ° (c 0.38, $CHCl_3$); 99% ee; Chiral HPLC analysis of the product: Phenomenex 00G-4457-E0 250X4.6 mm 5u column; hexane/2-propanol = 99.6/0.4, detected at 254 nm, Flow rate = 1 mL/min, Retention times: 14.1 min (major), 20.4 min (minor).

Supplementary Figure 40. HPLC spectra of 6

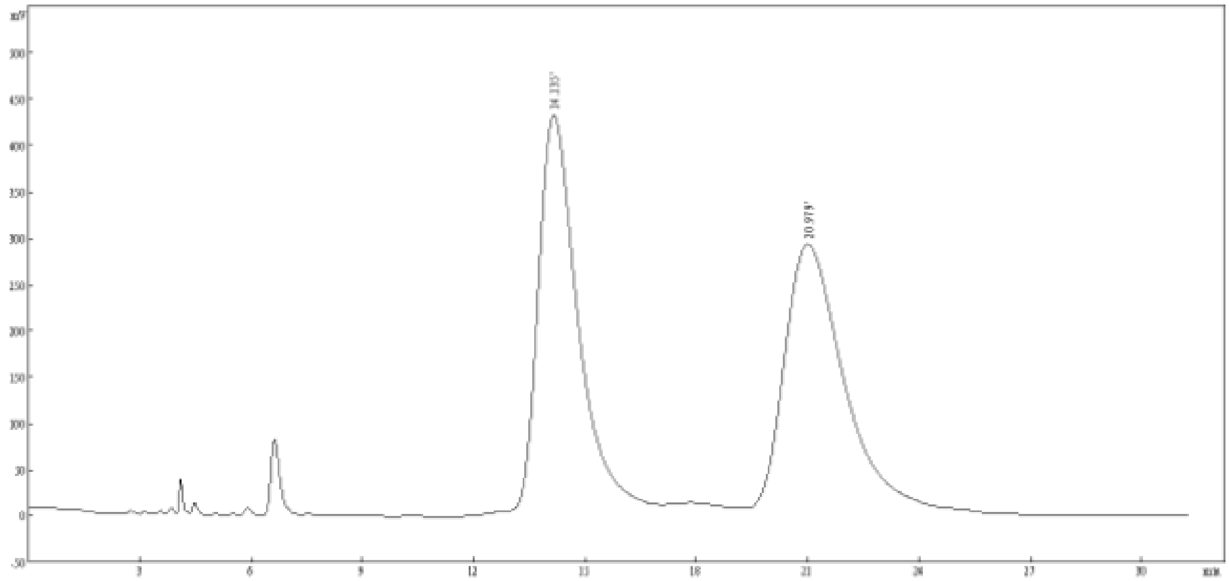

Peak#	Ret.Time	Area	Area %
1	14.135	30969597	49.02
2	20.979	32207229	50.98
Total		63176826	100

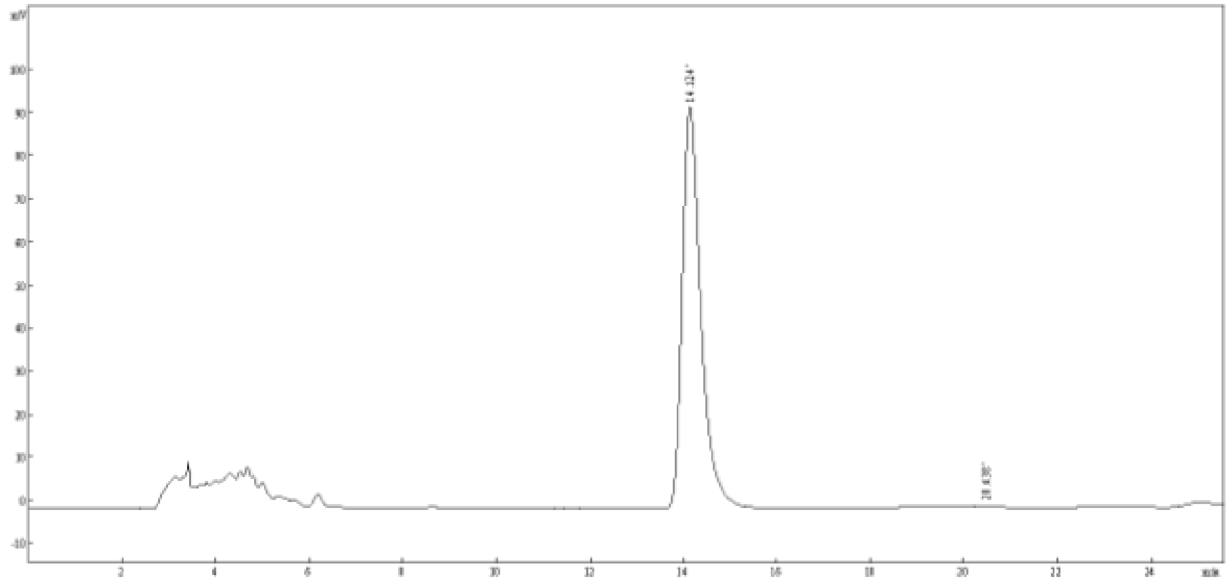

Peak#	Ret.Time	Area	Area %
1	14.124	2602996	99.4

2	20.438	15669	0.5984
Total		2618665	100

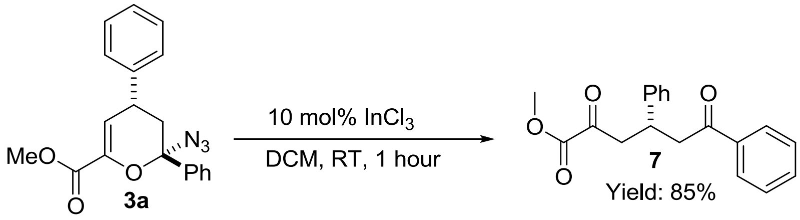

Supplementary Figure 41. Procedure for the preparation compound **7**

To a solution of **3a** (67.0 mg, 0.2 mmol, 1 eq) in 2 mL of DCM, $InCl_3$ (4.4 mg, 0.02 mmol, 0.1 eq) was added at room temperature. After 1 hour of stirring (complete consumption of starting material, indicated by TLC analysis) the reaction mixture was filtered through the celite pad, evaporated under reduced pressure, purified by column chromatography (silica gel, EtOAc/Petroleum ether: 5/50 to 10/50) to obtain the desired product **7** (52.7 mg, 85% yield) as a light yellow soild **.**

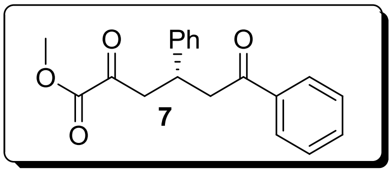

Light yellow soild; (52.7 mg, 85% yield); ^{1}H NMR (500 MHz, $CDCl_3$) δ 7.91-7.89 (m, 2H), 7.56-7.52 (m, 1H), 7.43 (t, J = 8.0 Hz, 2H), 7.29-7.25 (m, 4H), 7.21-7.18 (m, 1H), 3.96 (t, J = 6.9 Hz, 1H), 3.81 (s, 3H), 3.37-3.35 (m, 2H), 3.30 (t, J = 7.3 Hz, 2H); ^{13}C NMR (125 MHz, $CDCl_3$) δ 198.2, 192.2, 161.1, 143.1, 136.7, 133.2, 128.7, 128.6, 128.0, 127.4, 126.9, 52.9, 45.4, 44.5, 36.1; IR (neat): 2928, 1729, 1680, 1448, 1263, 1067, 747, 697 cm^{-1}; HRMS exact mass calcd for $C_{19}H_{18}NaO_4$ $[M + Na]^+$ 333.1103, found 333.1102.

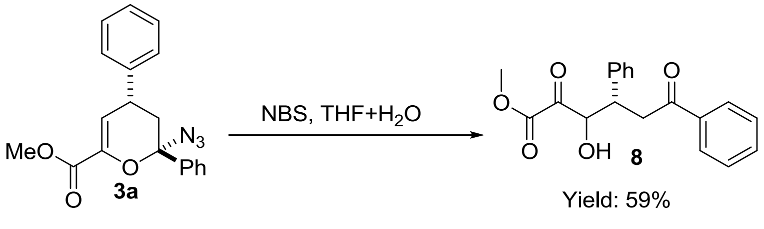

Supplementary Figure 42. Procedure for the preparation compound **8**

To a mixture of **3a** (67.0 mg, 0.2 mmol, 1 eq) and 2 mL of THF:H_2O (2:1), NBS (70.8 mg, 0.4 mmol, 2 eq) was added at room temperature. After 12 hour of stirring (complete consumption of starting material, indicated by TLC analysis) the reaction mixture was diluted with 5 mL of water and extracted with EtOAc (2X10 mL). The combined organic layers were washed with brine, dried over Na_2SO_4, concentrated under reduced pressure. Crude was purified by column chromatography (silica gel, EtOAc/Petroleum ether: 10/50) to obtain the desired product **8** (38.4 mg, 59% yield) as a white solid.

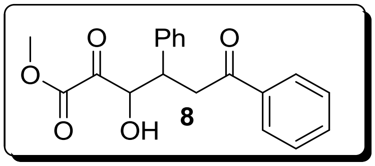

White solid; (38.4 mg, 59% yield); ^{1}H NMR (500 MHz, $CDCl_3$) δ 7.90 (dd, J = 8.1, 1.0 Hz, 2H), 7.56-7.53 (m, 1H), 7.47-7.46 (m, 2H), 7.42 (t, J = 8.1 Hz, 2H), 7.37-7.34 (m, 2H), 7.29-7.26 (m, 1H), 5.00 (s, 1H), 4.19-4.15 (m, 1H), 3.98 (s, 3H), 3.79-3.73 (m, 1H), 3.33 (bs, 1H), 3.18 (dd, J = 18.6, 3.7 Hz, 1H); ^{13}C NMR (125 MHz, $CDCl_3$) δ 199.4, 193.1, 160.4, 142.4, 136.3, 133.5, 128.8, 128.6, 128.1, 128.0, 127.2, 77.8, 53.2, 42.8, 38.0; IR (neat): 2925, 2855, 1665, 1465, 1278, 1263, 749, 700 cm^{-1}; HRMS exact mass calcd for $C_{19}H_{18}NaO_5$ $[M + H]^+$ 349.1052, found 349.1048.

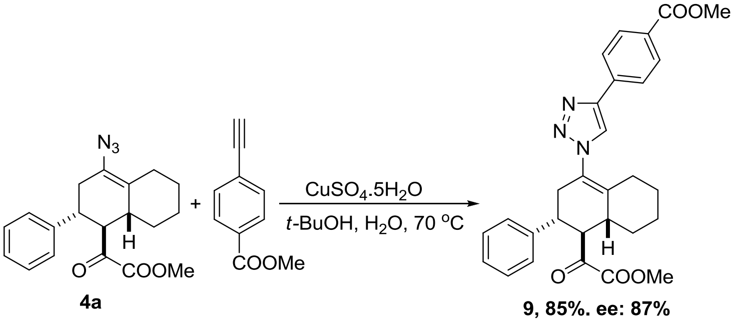

Supplementary Figure 43. Procedure for the preparation compound **9**

To the mixture of **4a** (67.8 mg, 0.2 mmol, 1 eq), $CuSO_4$ $5H_2O$ (9.9 mg, 0.04 mmol, 0.2 eq), and Sodium ascarbate (15.8 mg, 0.08 mmol, 0.4 eq) in 2 mL (t-BuOH+H_2O, 1:1) alkyne (38.4 mg, 0.24 mmol, 1.2 eq) was added at room temperature. Then, the reaction mixture was stirred at 70 ^{o}C for 12 hours. After cool down to the room temperatute, it was concentrated, diluted with 5 mL of water and extracted with ethyl acetate (3X10 mL). The combined organic layers were dried on Na_2SO_4, concentrated under reduced pressure, purified by column chromatography (silica gel, Petroleum ether/ EtOAc: 6.6/1) to get the desired product **9 (**84.8 mg, 85% yield) as a light yellow soild.

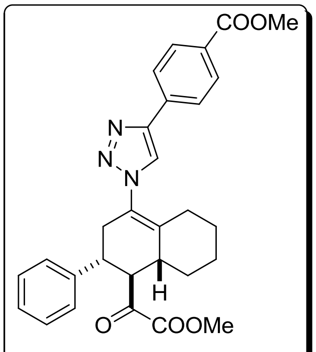

9, 85%. ee: 87%

Light yellow solid; (84.8 mg, 85% yield); ^{1}H NMR (500 MHz, $CDCl_3$) δ 8.03 (d, J = 8.2 Hz, 2H), 7.86 (d, J = 8.2 Hz, 2H), 7.79 (s, 1H), 7.21-7.11 (m, 5H), 3.86 (s, 3H), 3.72 (t, J = 10.1 Hz, 1H), 3.46 (s, 3H), 3.21-3.15 (m, 1H), 2.89-2.81 (m, 2H), 2.67-2.64 (m, 1H), 2.54-2.46 (m, 1H), 2.28-2.25 (m, 1H), 1.74-1.72 (m, 4H), 1.35-1.24 (m, 3H); ^{13}C NMR (125 MHz, $CDCl_3$) δ 196.9, 165.7, 160.6, 145.2, 138.7, 136.9, 133.6, 129.2, 128.6, 127.8, 126.9, 126.6, 125.5, 124.4, 120.2, 53.7, 51.7, 51.1, 42.9, 40.4, 37.2, 32.4, 27.4, 25.4, 24.2; IR (neat): 2931, 2096, 1720, 1436, 1275, 1111, 1055, 767, 703 cm^{-1}; HRMS exact mass calcd for $C_{29}H_{30}N_3O_5$ $[M + H]^+$ 500.2185, found 500.2178. $[\alpha]^{16}_{D}$ = -82.9 ° (c 0.29, $CHCl_3$); 87% ee; Chiral HPLC analysis of the product: Daicel Chiralpak AD-H 250X4.6 mm 5u column; hexane/2-propanol = 60/40, detected at 254 nm, Flow rate = 1 mL/min, Retention times: 25.4 min (minor), 38.5 min (major).

Supplementary Figure 44. HPLC spectra of 9

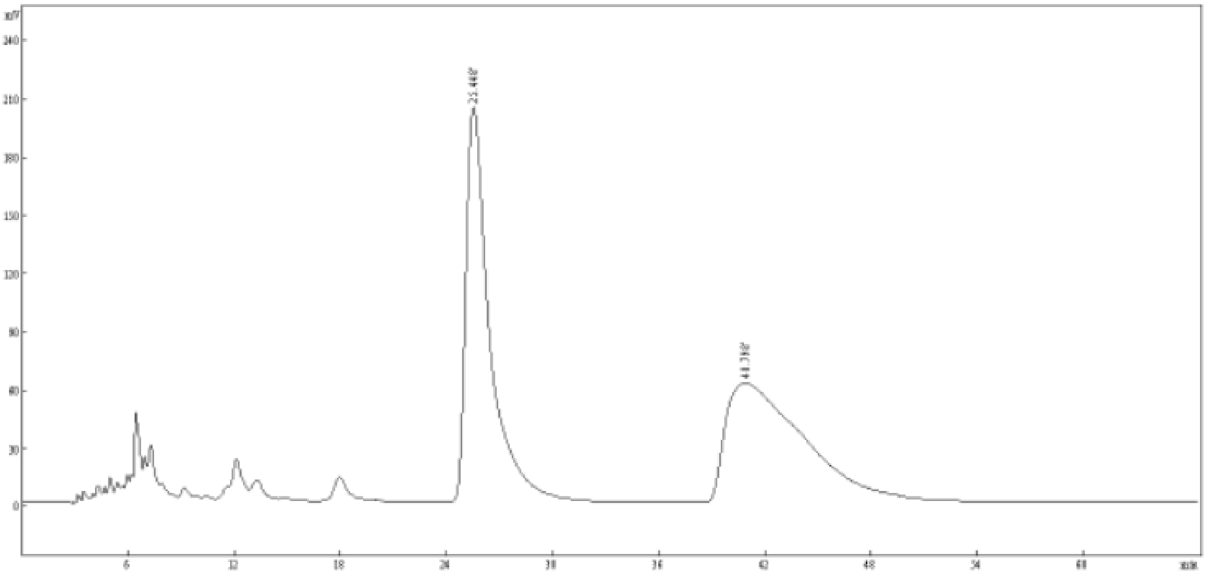

Peak#	Ret.Time	Area	Area %

1	25.448	19075534	50.4
2	29.525	18772944	49.6
Total		37848478	100

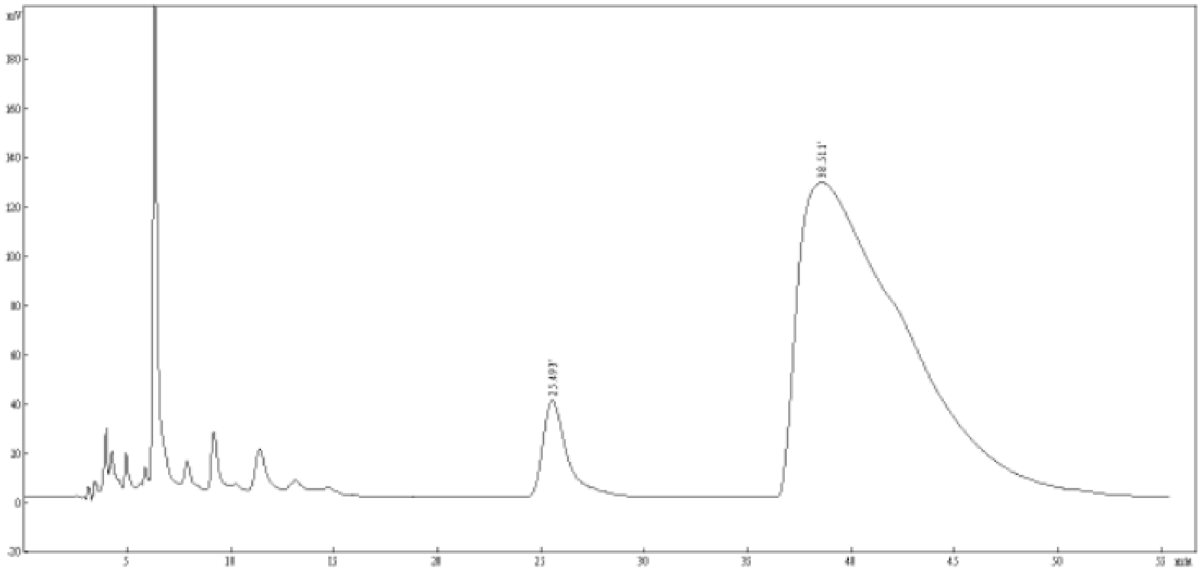

Peak#	Ret.Time	Area	Area %
1	25.493	3261669	6.639
2	29.525	45871098	93.36
Total		49132767	100

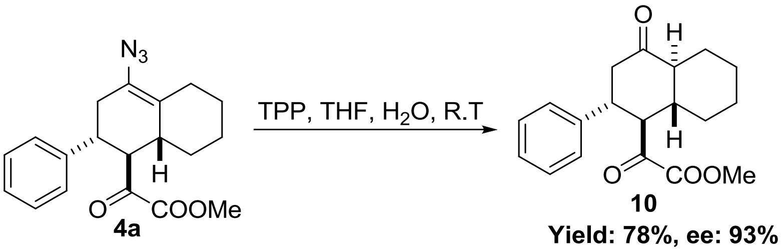

Supplementary Figure 45. Procedure for the preparation compound **10**

To the mixture of **4a** (67.8 mg, 0.2 mmol, 1 eq) and H_2O (18.0 mg, 1 mmol, 5 eq) in 2 mL of THF, PPh_3 (131 mg, 0.5 mmol, 2.5 eq) was added at room temperature. After 12 hour of stirring

(complete consumption of starting material, indicated by TLC analysis) the reaction mixture was diluted with 5 mL of water and extracted with EtOAc (2X10 mL). The combined organic layers were washed with brine, dried over Na_2SO_4, concentrated under reduced pressure. Crude was purified by column chromatography (silica gel, EtOAc/Petroleum ether: 5/50) to obtain the desired product **10 (**48.9 mg, 78% yield) as a white solid.

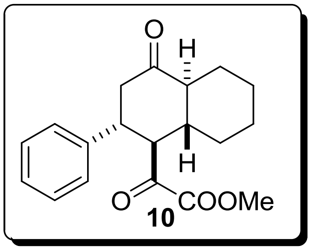

White solid; (48.9 mg, 78% yield); ^{1}H NMR (500 MHz, $CDCl_3$) δ 7.29-7.26 (m, 2H), 7.22-7.17 (m, 3H), 3.97 (t, J = 11.2 Hz, 1H), 3.58 (s, 3H), 3.29-3.23 (m, 1H), 2.74-2.69 (m, 1H), 2.58 (dd, J = 13.7, 4.2 Hz, 1H), 2.25 (td, J = 11.3, 2.4 Hz, 1H), 2.06-1.93 (m, 2H), 1.85-1.82 (m, 1H), 1.71-1.68 (m, 1H), 1.62-1.57 (m, 1H), 1.36-1.16 (m, 4H); ^{13}C NMR (125 MHz, $CDCl_3$) δ 208.6, 197.4, 161.3, 139.9, 128.9, 127.5, 127.4, 56.0, 53.0, 52.8, 48.4, 48.0, 46.0, 31.8, 25.3, 25.0, 24.9; IR (neat): 2990, 1720, 1465, 1275, 1263, 1034, 764, 753 cm^{-1}; HRMS exact mass calcd for $C_{19}H_{22}NaO_4$ [M + H]$^+$ 337.1416, found 337.1422. $[\alpha]^{16}_D$ = -10.7 ° (c 0.38, $CHCl_3$); 94% ee; Chiral HPLC analysis of the product: Phenomenex 00G-4457-E0 250X4.6 mm 5u column; hexane/2-propanol = 92/8, detected at 254 nm, Flow rate = 1 mL/min, Retention times: 15.7 min (major), 23.8 min (minor).

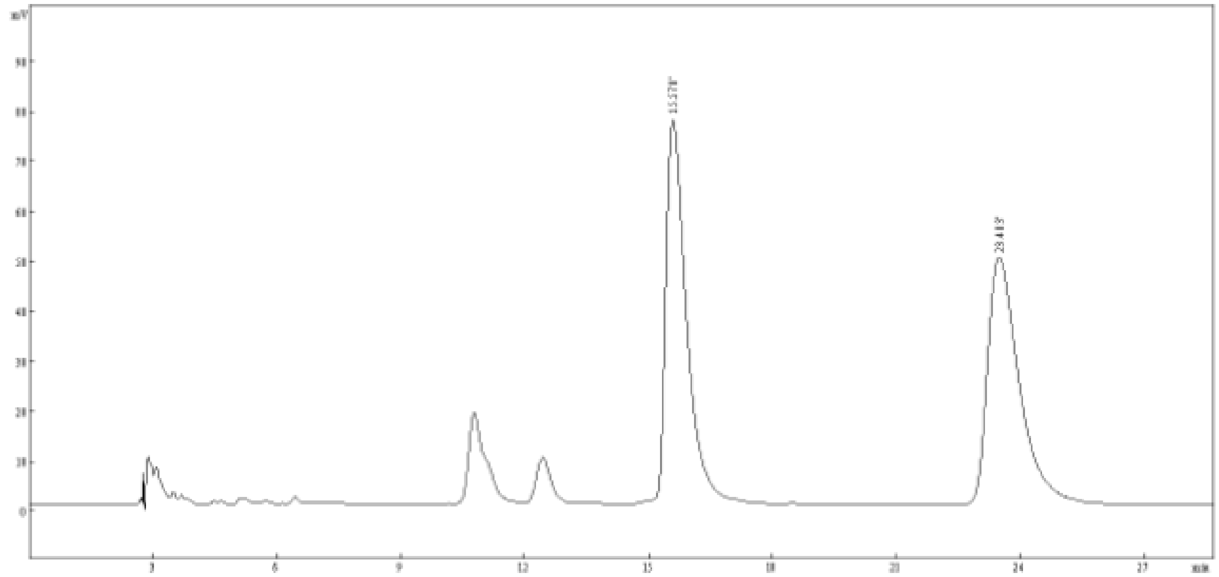

Peak#	Ret.Time	Area	Area %

1	15.578	2698692	50.49
2	23.483	2646837	49.51
Total		5345529	100

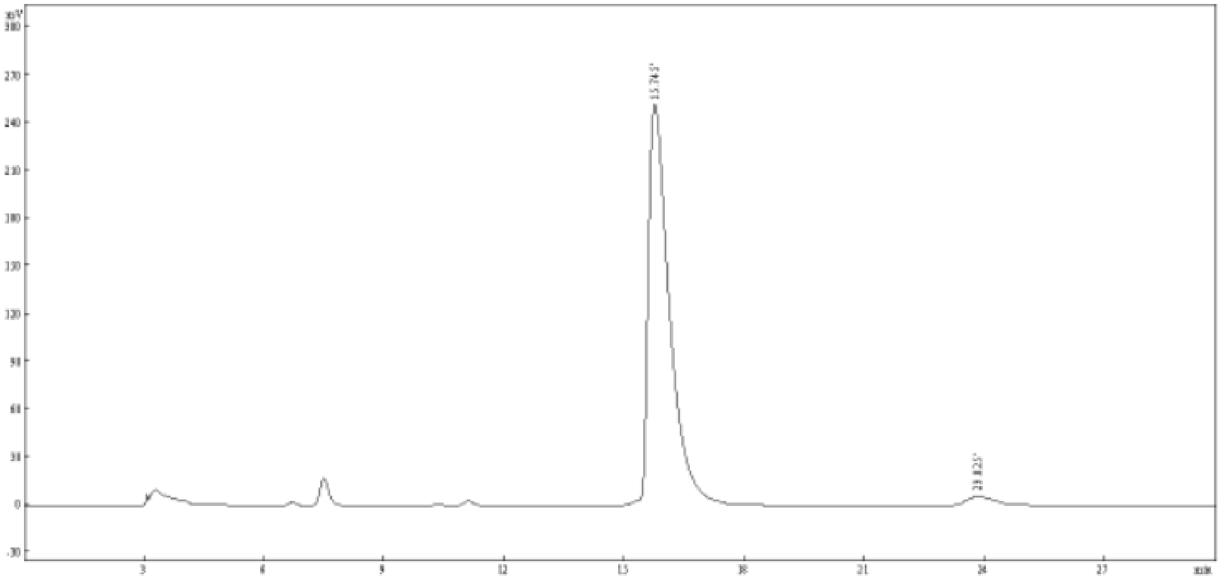

Peak#	Ret.Time	Area	Area %
1	15.745	9372527	96.92
2	23.825	297711	3.079
Total		9670238	100

Scale up Experiment

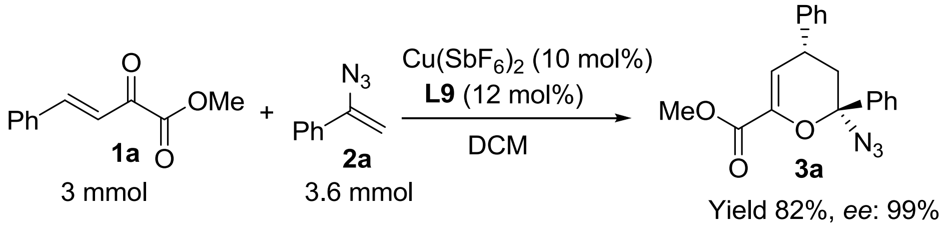

A mixture of $CuBr_2$ (66.9 mg, 0.3 mmol, 10 mol%), $AgSbF_6$ (205.8 mg, 0.6 mmol, 20 mol%), ligand **L9** (167.8 mg, 0.36 mmol, 12 mol%) in DCM (9 mL) were stirred at 30 ^oC for 3 h under nitrogen. Then, this catalyst solution was added via syringe to the mixture of **1a** (570 mg, 3 mmol, 1 eq), **2a** (522 mg, 3.6 mmol, 1.2 eq), and 1.5 gm of 4A^o MS in DCM (9 mL). The resulting suspension was stirred at 30 ^oC and monitored by TLC, until the complete consumption

of **1** (48 hours). Then, the mixture was filtered through a pad of celite and washed with DCM. The filterate was concentrated under reduced pressure and the residue was purified by column chromatography over silicagel using (ethyl acetate/petroleum ether, 1/50) to afford **3a (**824.0 mg, 82 % yield).

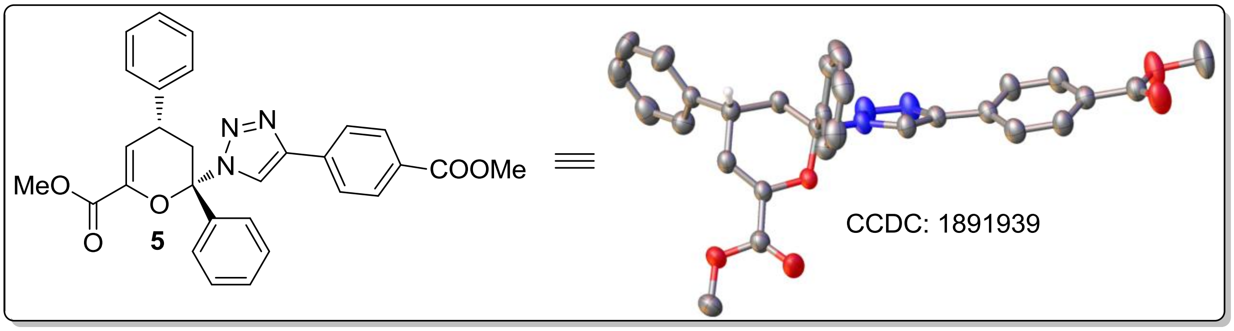

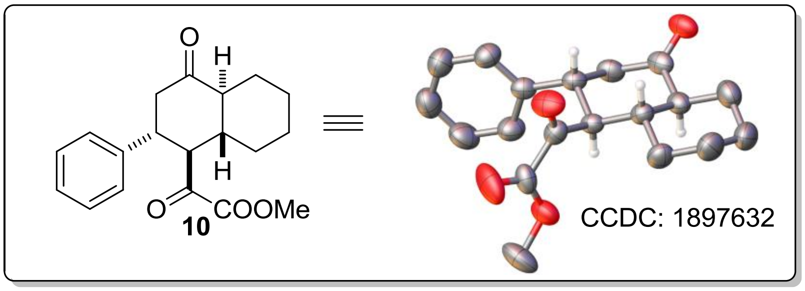

Supplementary Figure 46. X-ray Crystallography Data for compounds 5 and 1

Supplementary Figure 47. ^{1}H and ^{13}C NMR spectra for 3a

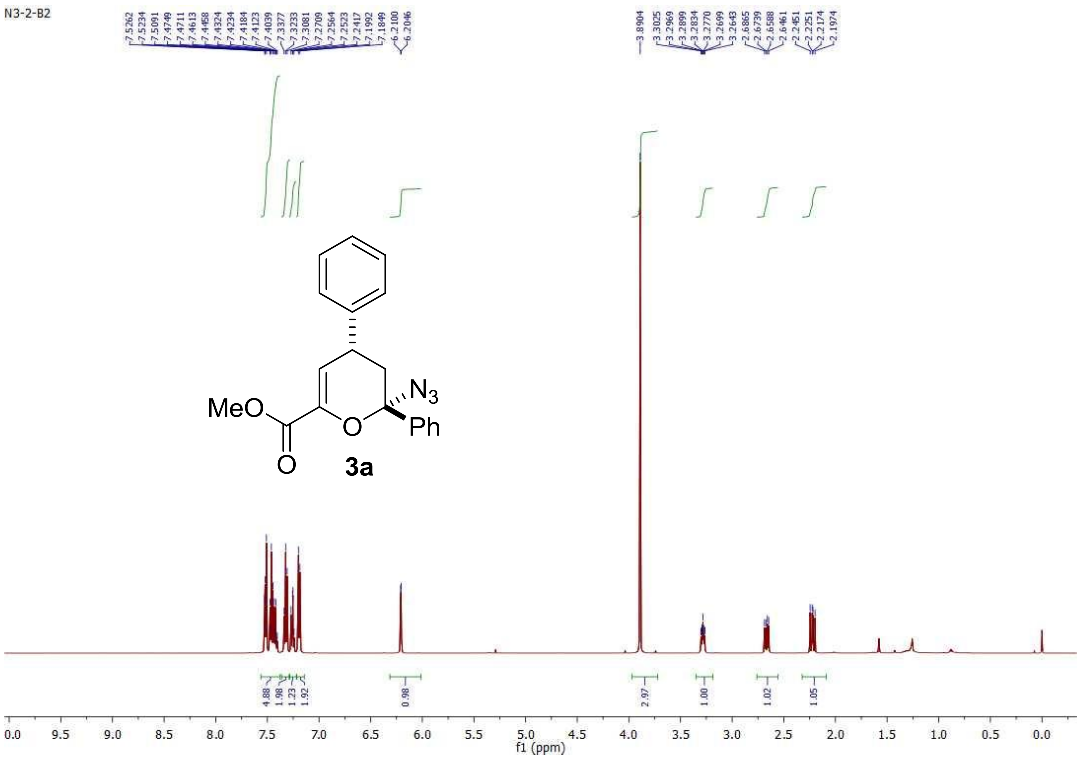

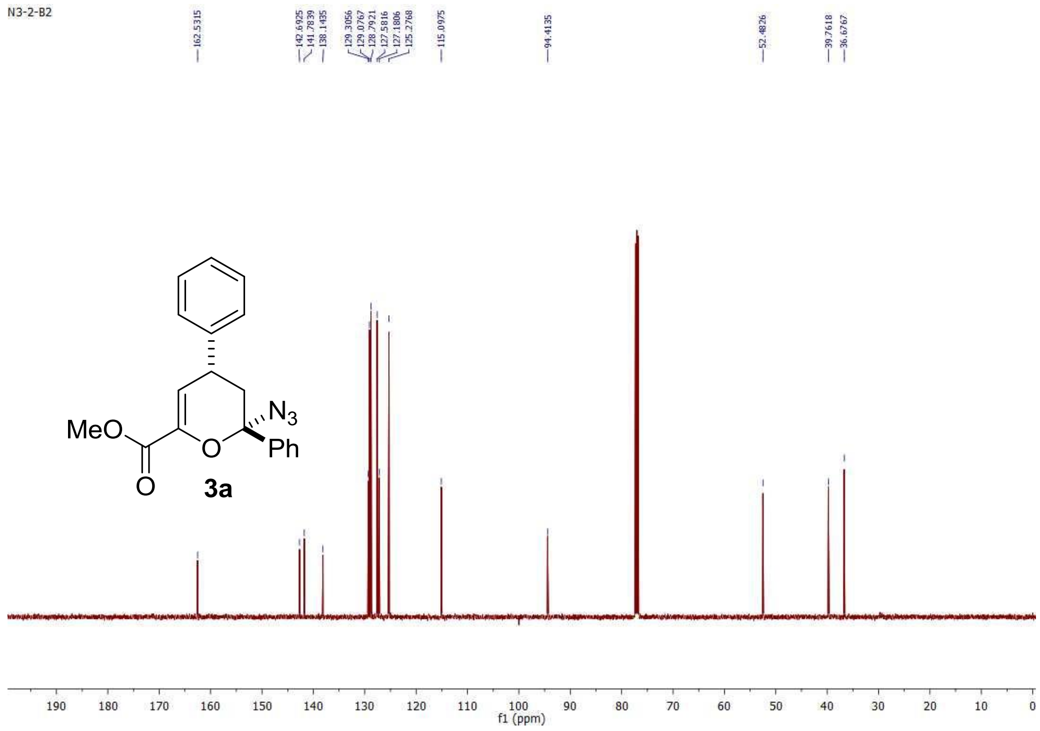

Supplementary Figure 48. HRMS spectra for 3a

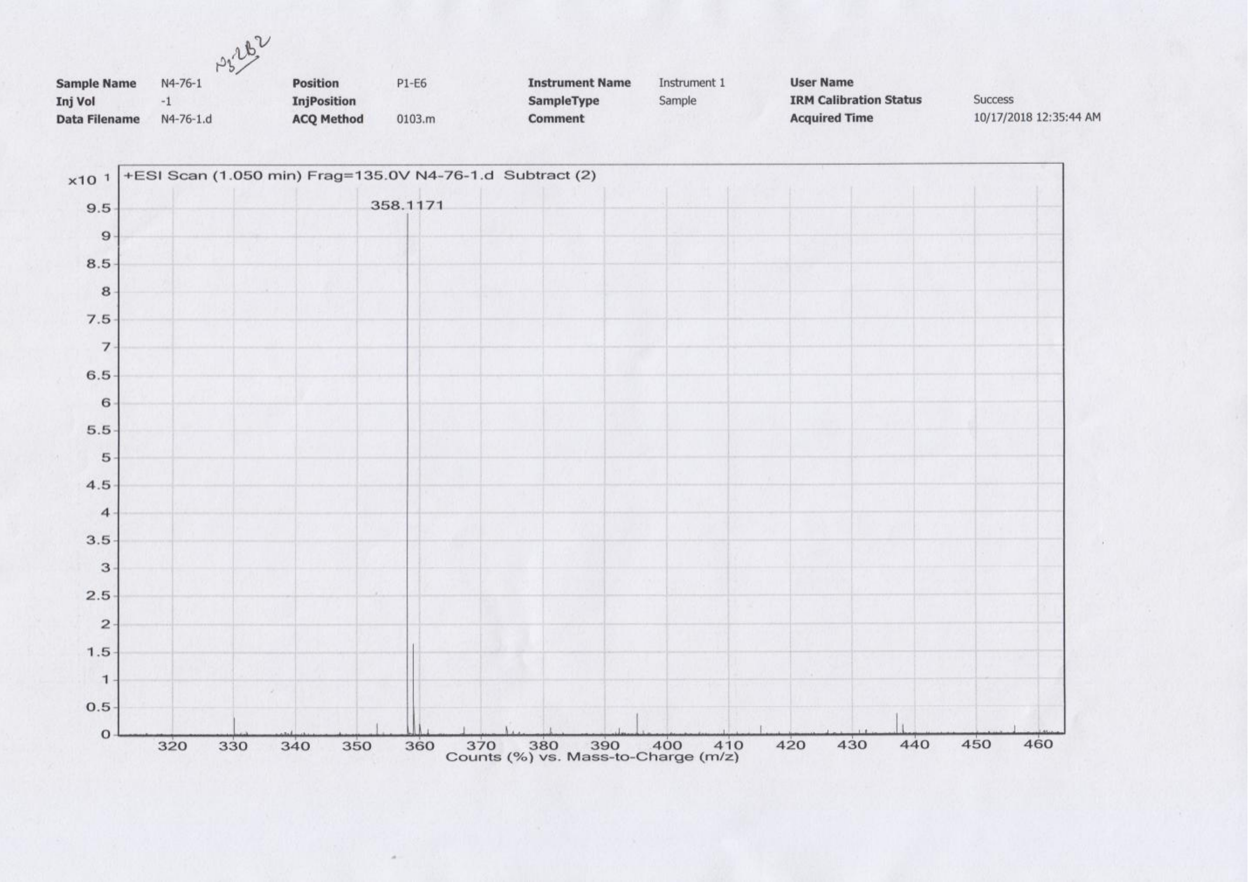

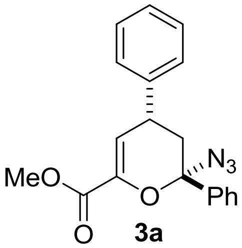

3a

Chemical Formula: $C_{19}H_{17}N_3O_3$
Exact Mass: 335.1270
Molecular Weight: 335.3566
m/z: 335.1270 (100.0%), 336.1303 (20.5%), 337.1337 (2.0%), 336.1240 (1.1%)

HRMS exact mass calcd for $C_{19}H_{17}N_3NaO_3$ $[M + Na]^+$ **358.1168**, found **358.1171**.

Supplementary Figure 49. ^{1}H and ^{13}C NMR spectra for 3b

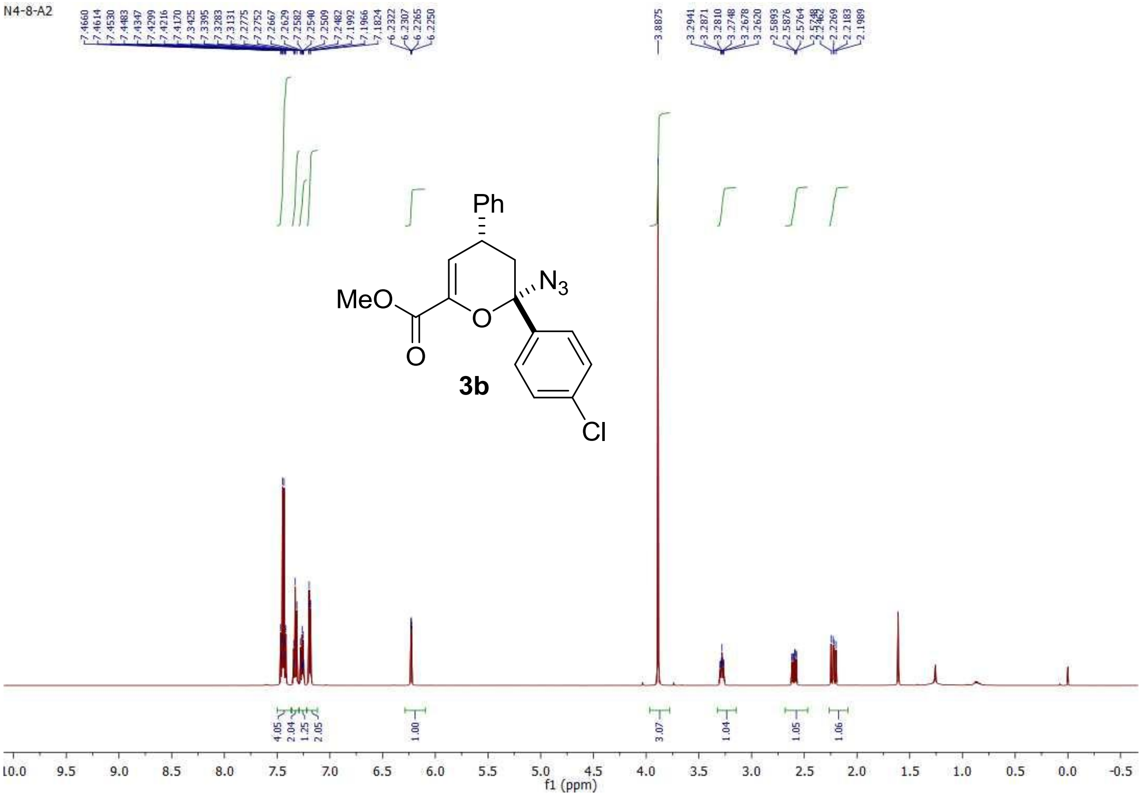

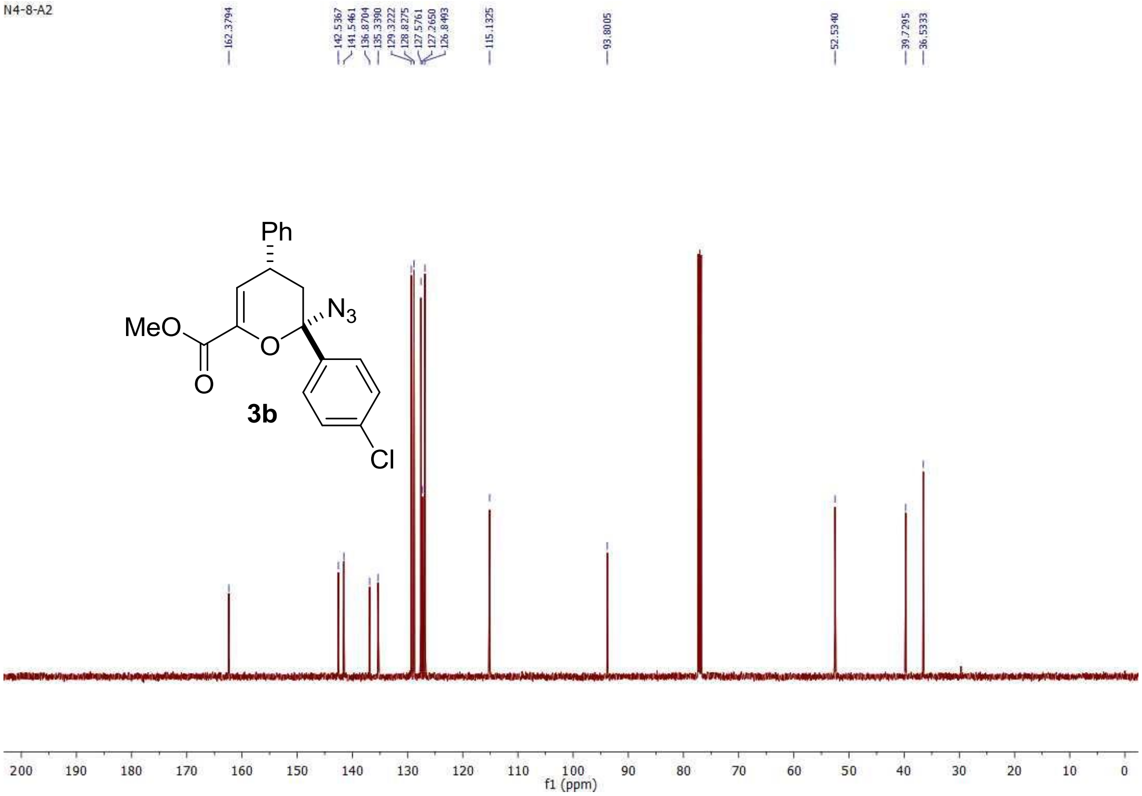

Supplementary Figure 50. HRMS spectra for 3b

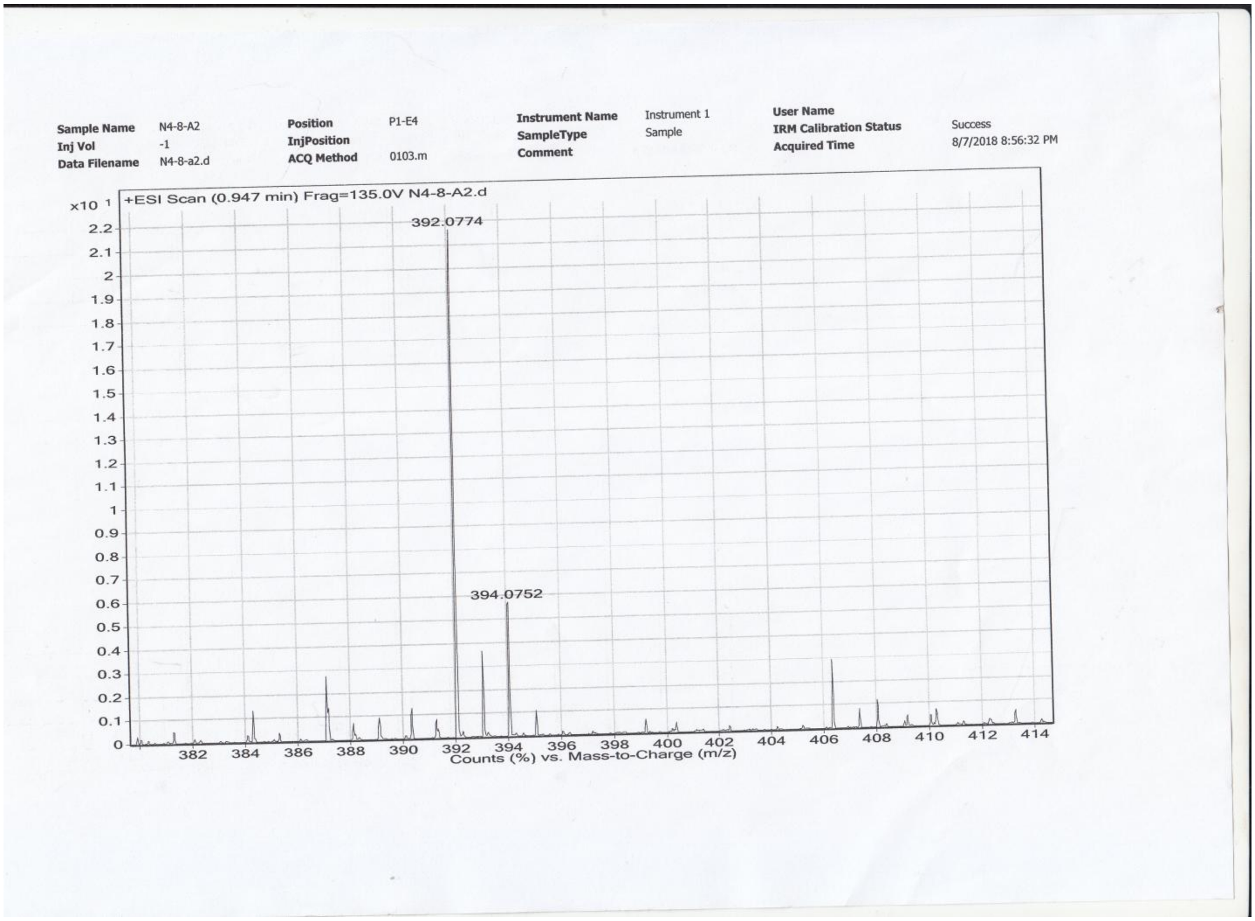

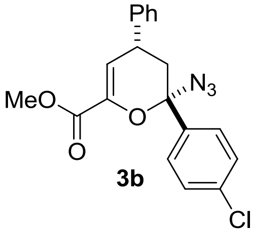

3b

Chemical Formula: $C_{19}H_{16}ClN_3O_3$
Exact Mass: 369.0880
Molecular Weight: 369.8016
m/z: 369.0880 (100.0%), 371.0851 (32.0%), 370.0914 (20.5%), 372.0884 (6.6%), 371.0947 (2.0%), 370.0851 (1.1%)

HRMS exact mass calcd for $C_{19}H_{16}ClN_3NaO_3$ $[M + Na]^+$ **392.0778**, found **392.0774**.

Supplementary Figure 51. ^{1}H and ^{13}C NMR spectra for 3c

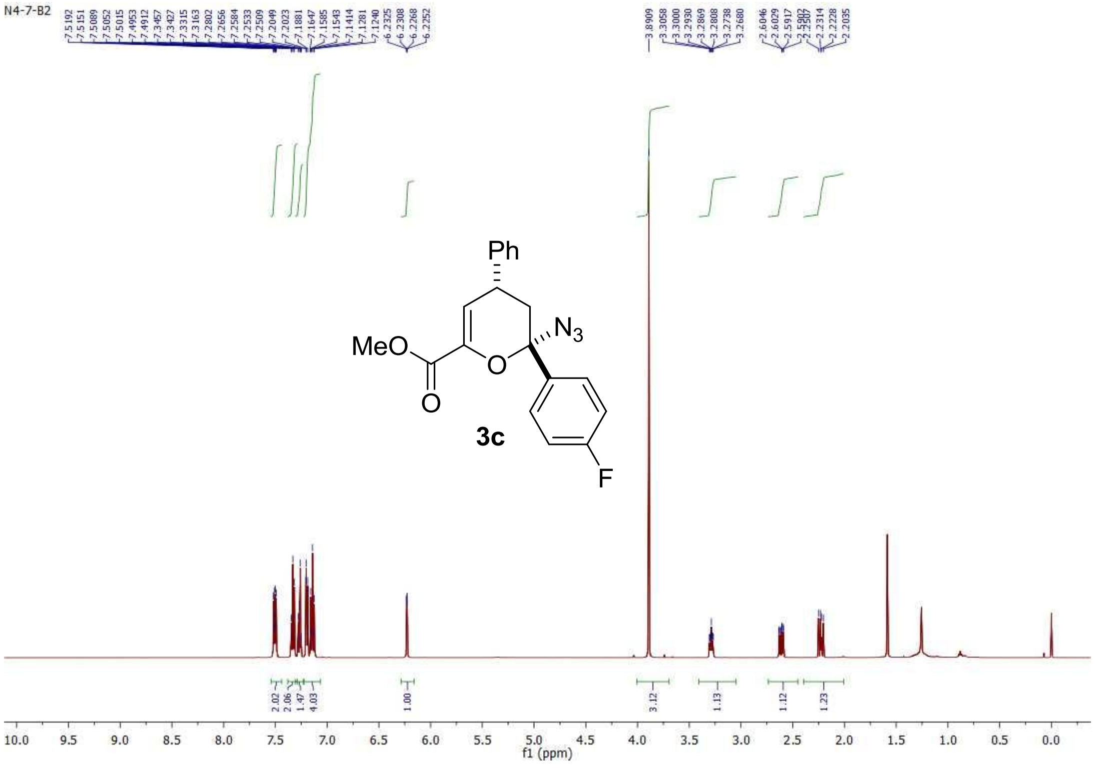

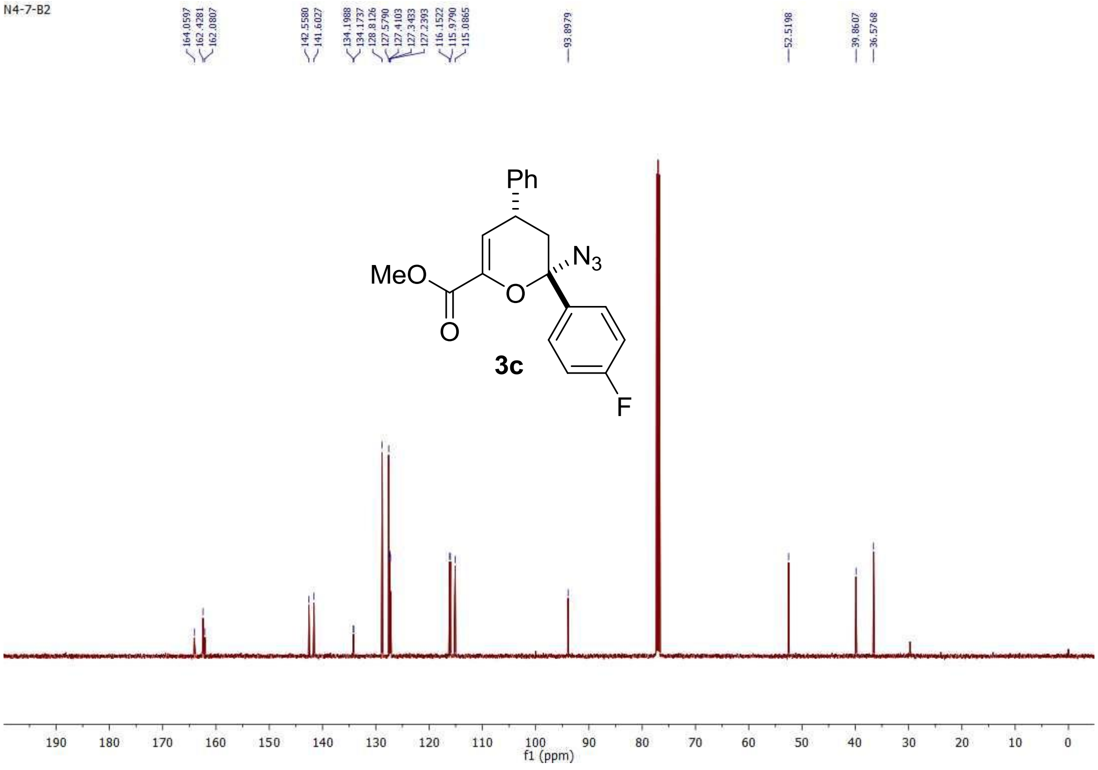

Supplementary Figure 52. HRMS spectra for 3c

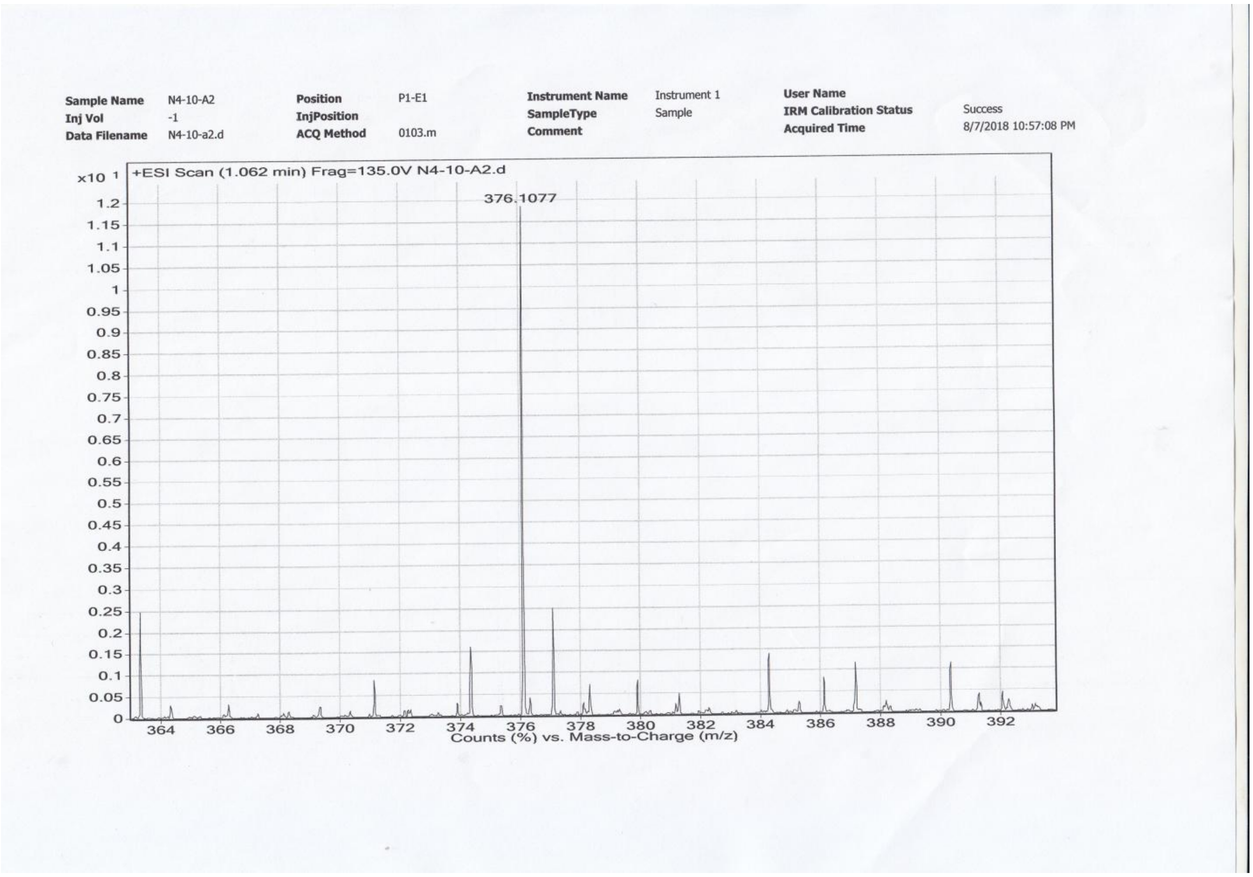

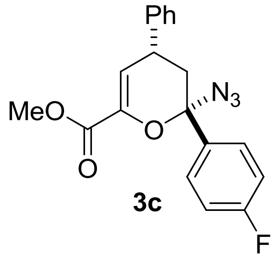

3c

Chemical Formula: $C_{19}H_{16}FN_3O_3$
Exact Mass: 353.1176
Molecular Weight: 353.3470
m/z: 353.1176 (100.0%), 354.1209 (20.5%), 355.1243 (2.0%), 354.1146 (1.1%)

HRMS exact mass calcd for $C_{19}H_{16}FN_3NaO_3$ $[M + Na]^+$ **376.3368**, found **376.1077**.

Supplementary Figure 53. ^{1}H and ^{13}C NMR spectra for 3d

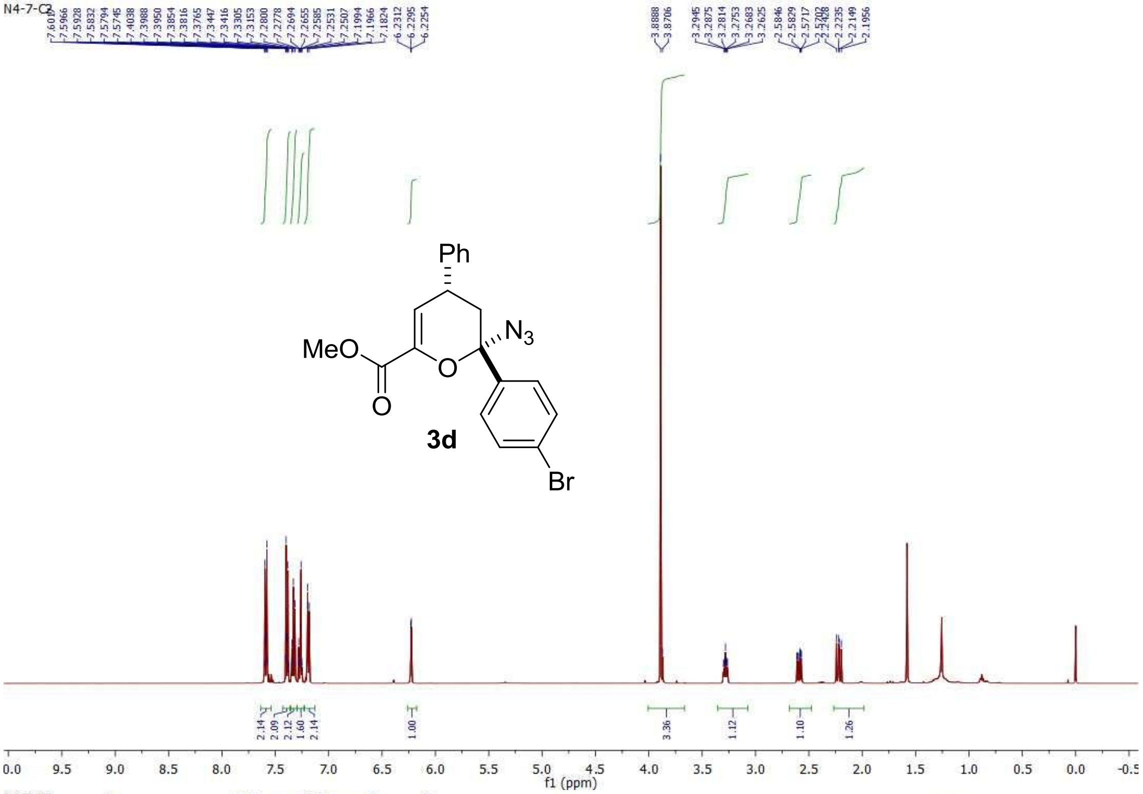

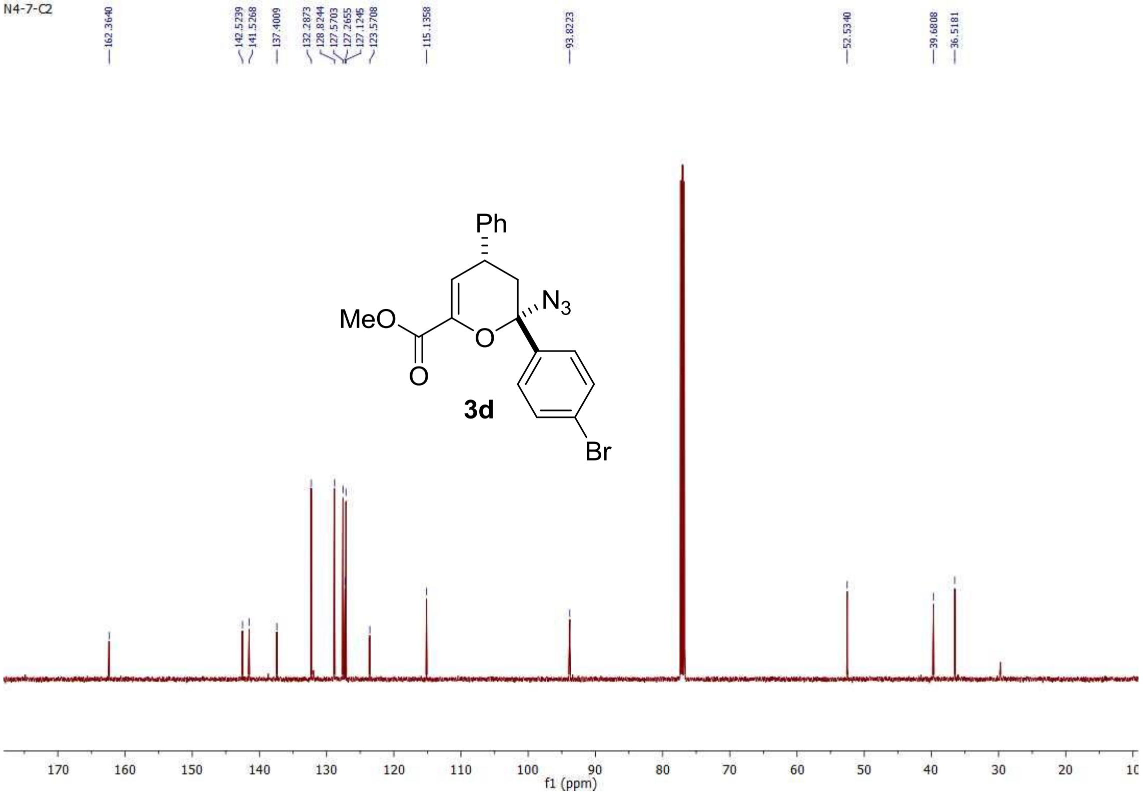

Supplementary Figure 54. HRMS spectra for 3d

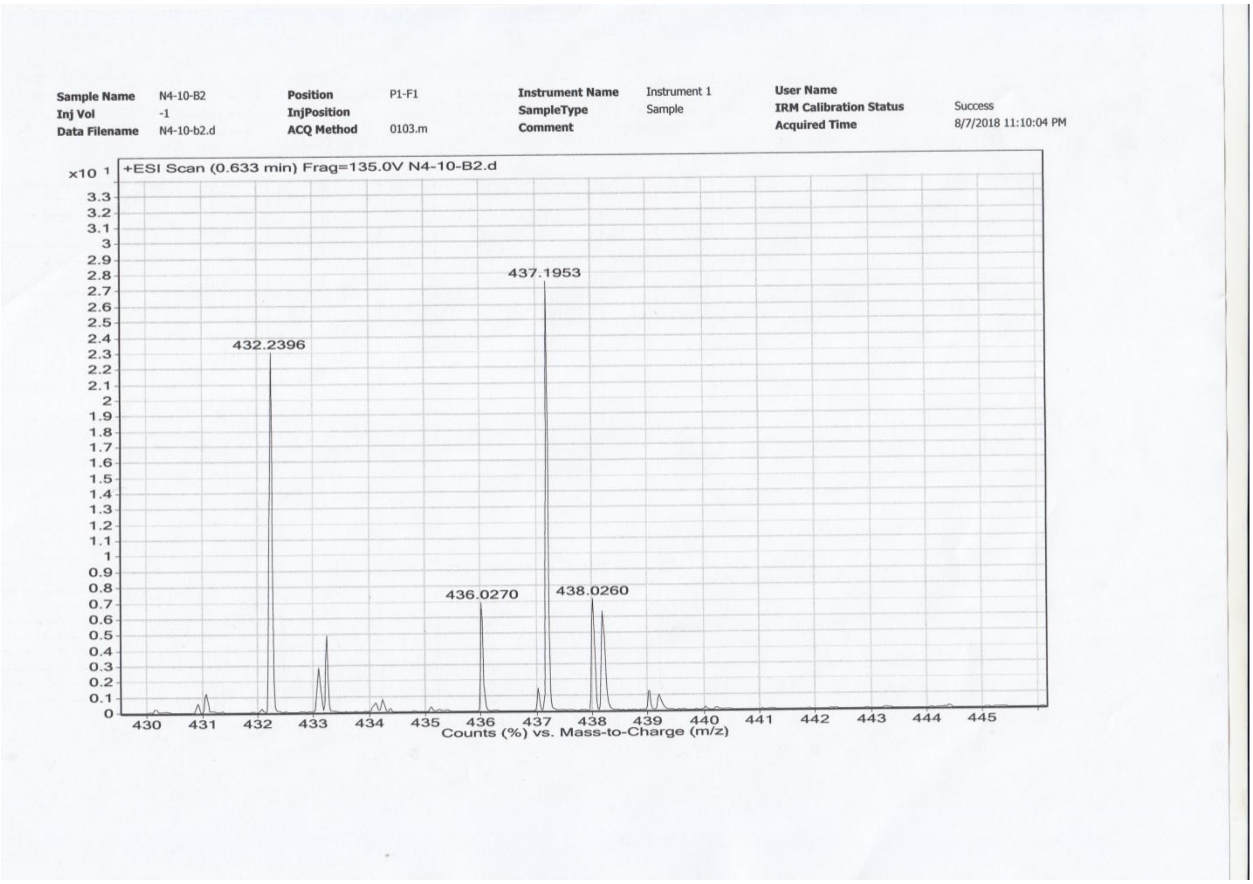

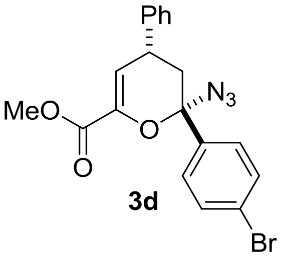

3d

Chemical Formula: $C_{19}H_{16}BrN_3O_3$
Exact Mass: 413.0375
Molecular Weight: 414.2526
m/z: 413.0375 (100.0%), 415.0355 (97.3%), 414.0409 (20.5%),
416.0388 (20.0%), 415.0442 (2.0%), 417.0422 (1.9%), 414.0345 (1.1%), 416.0325 (1.1%)

HRMS exact mass calcd for $C_{21}H_{14}BrO$ $[M - H]^+$ **436.0273**, found **436.0270**.

Supplementary Figure 55. ^{1}H and ^{13}C NMR spectra for 3e

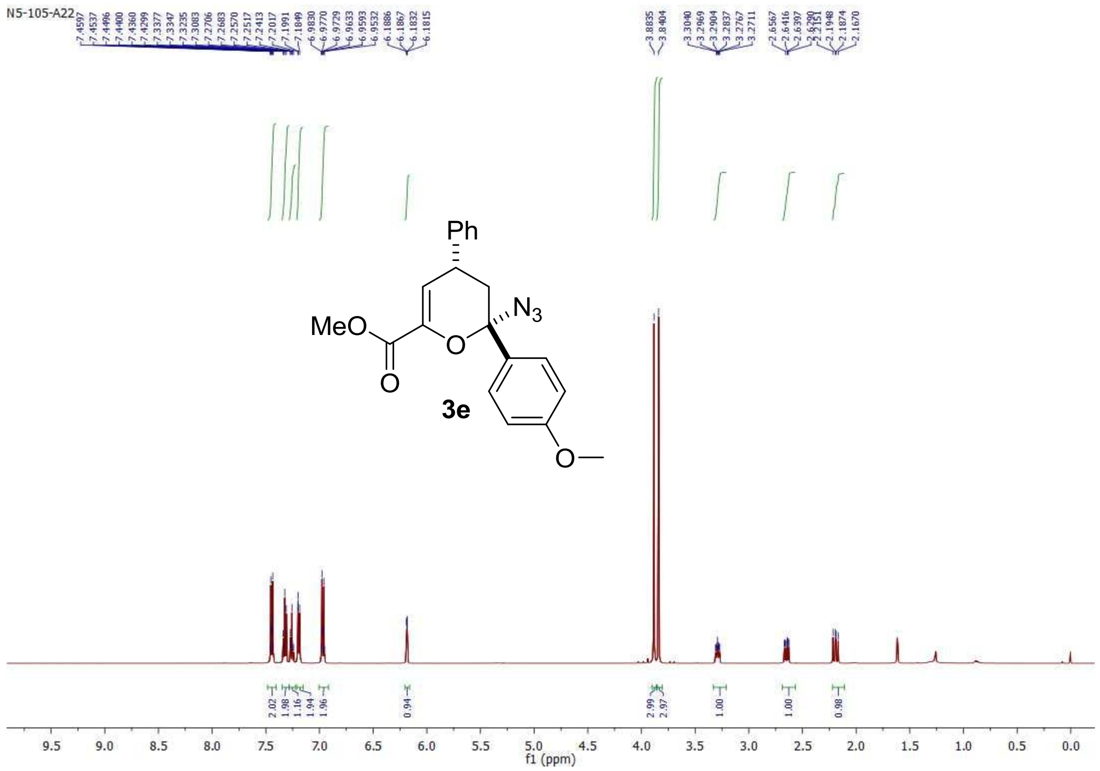

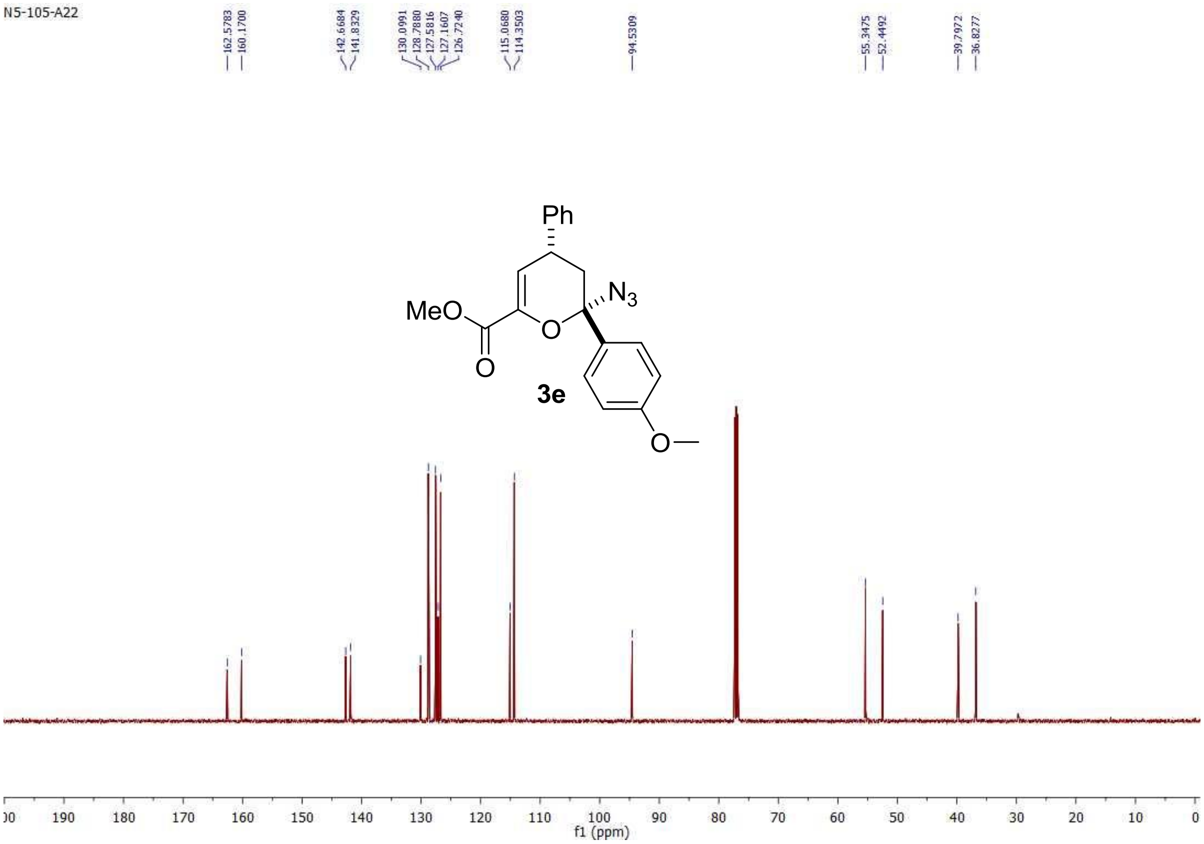

Supplementary Figure 56. HRMS spectra for 3e

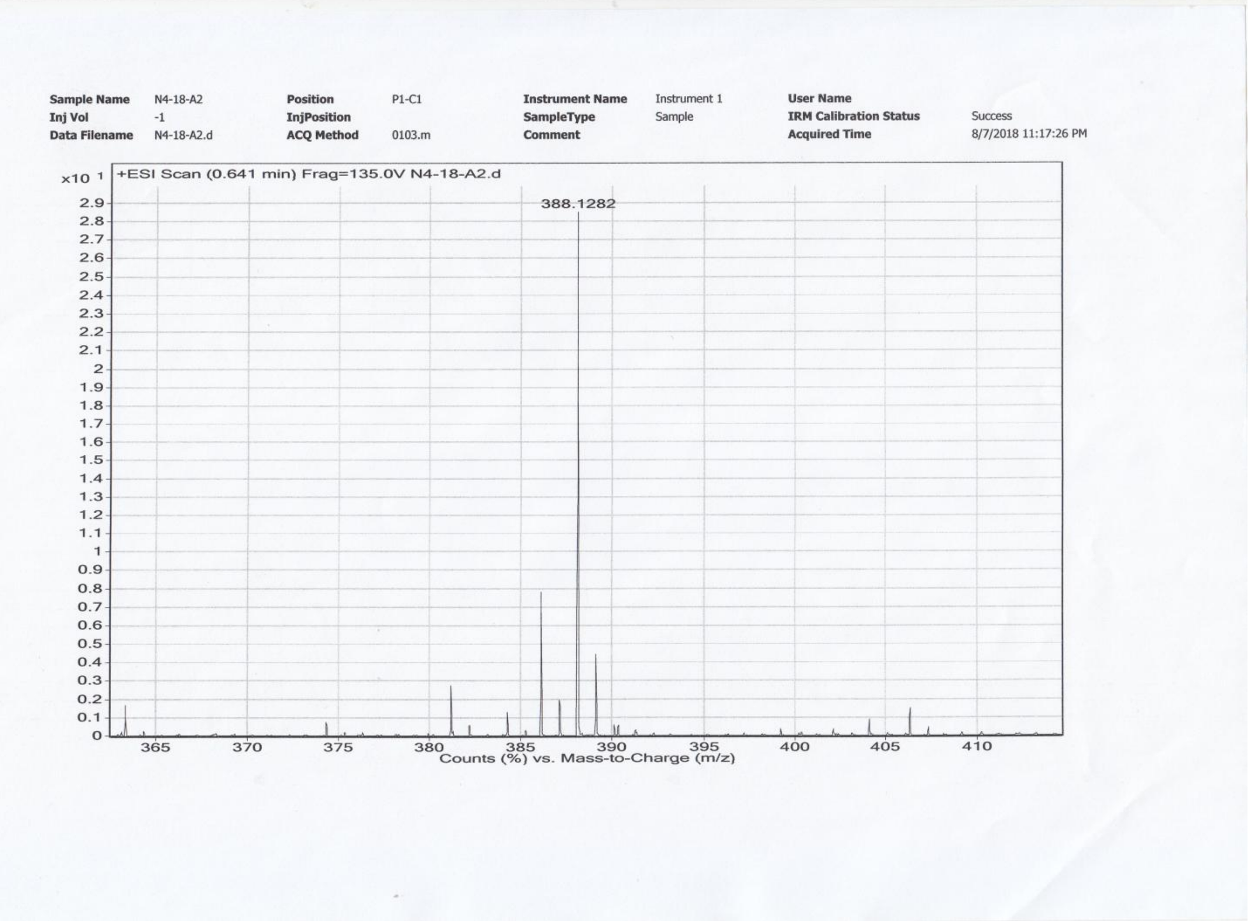

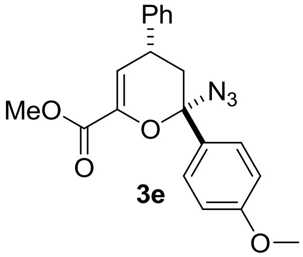

3e

Chemical Formula: $C_{20}H_{19}N_3O_4$

Exact Mass: 365.1376

Molecular Weight: 365.3826

m/z: 365.1376 (100.0%), 366.1409 (21.6%), 367.1443 (2.2%), 366.1346 (1.1%)

HRMS exact mass calcd for $C_{20}H_{19}N_3NaO_4$ $[M + Na]^+$ **388.1273**, found **388.1282**.

Supplementary Figure 57. ^{1}H and ^{13}C NMR spectra for 3f

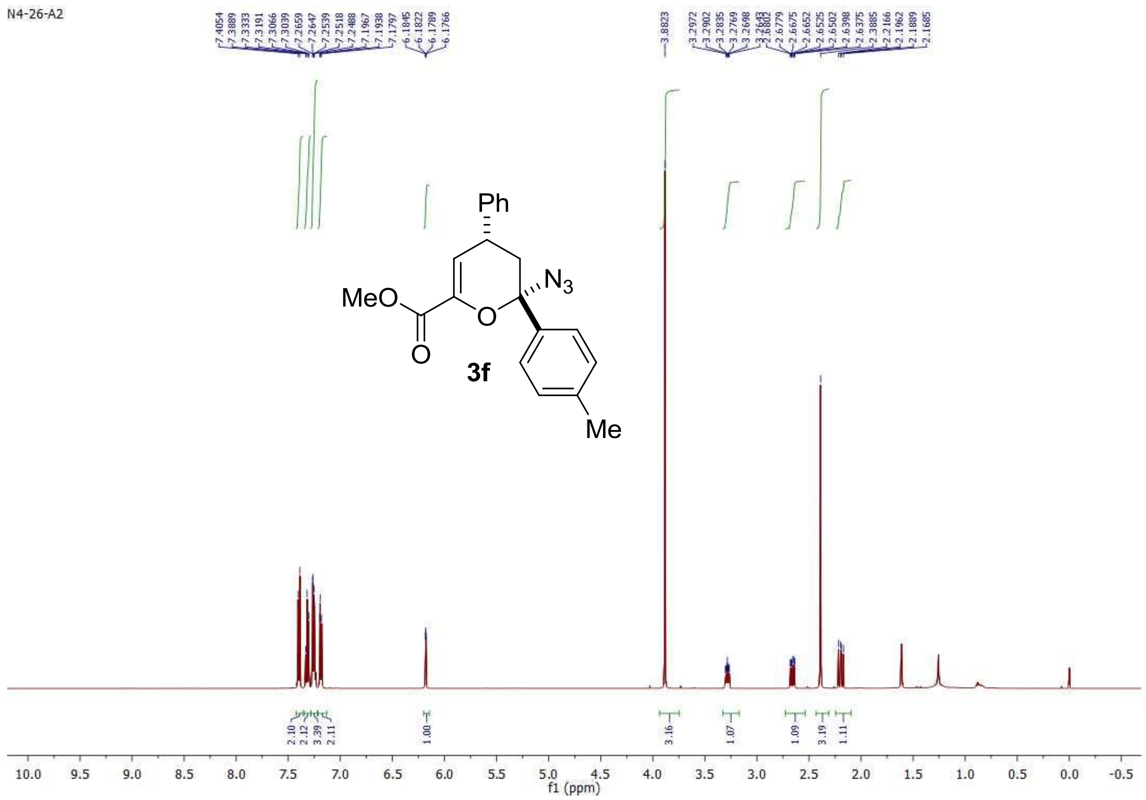

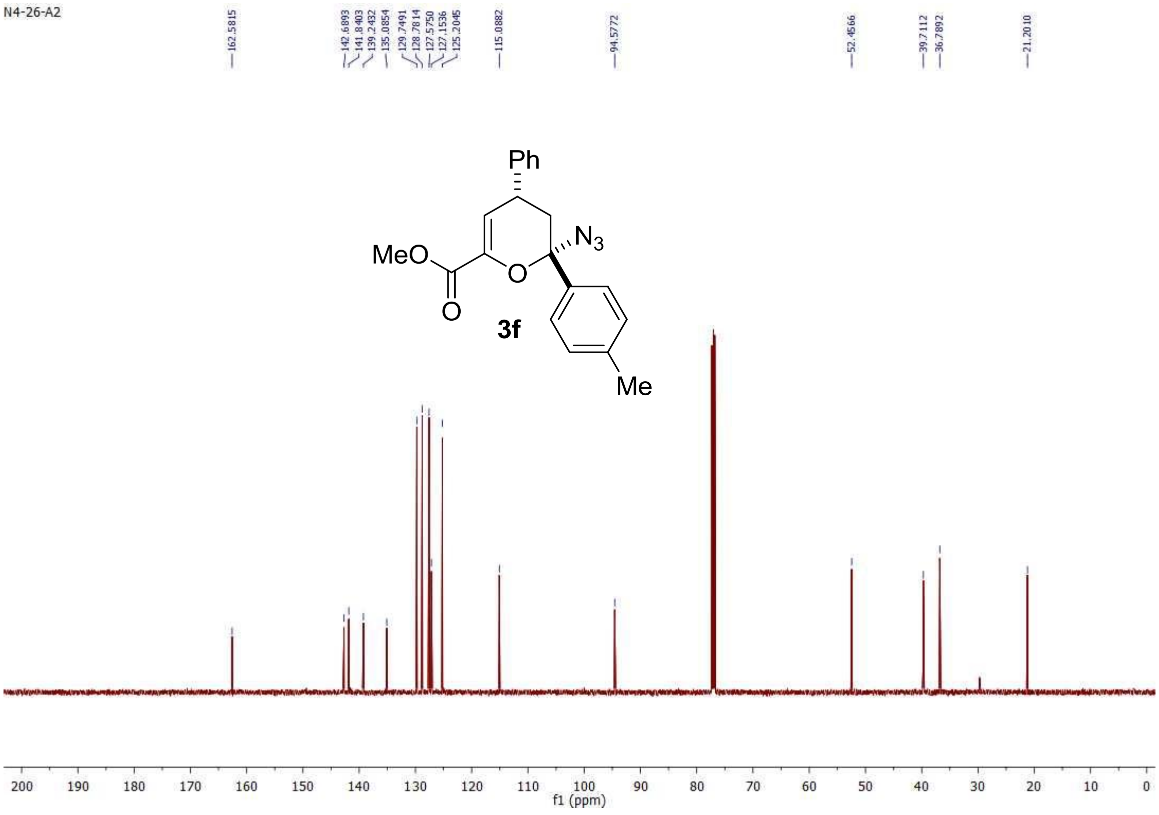

Supplementary Figure 58. HRMS spectra for 3f

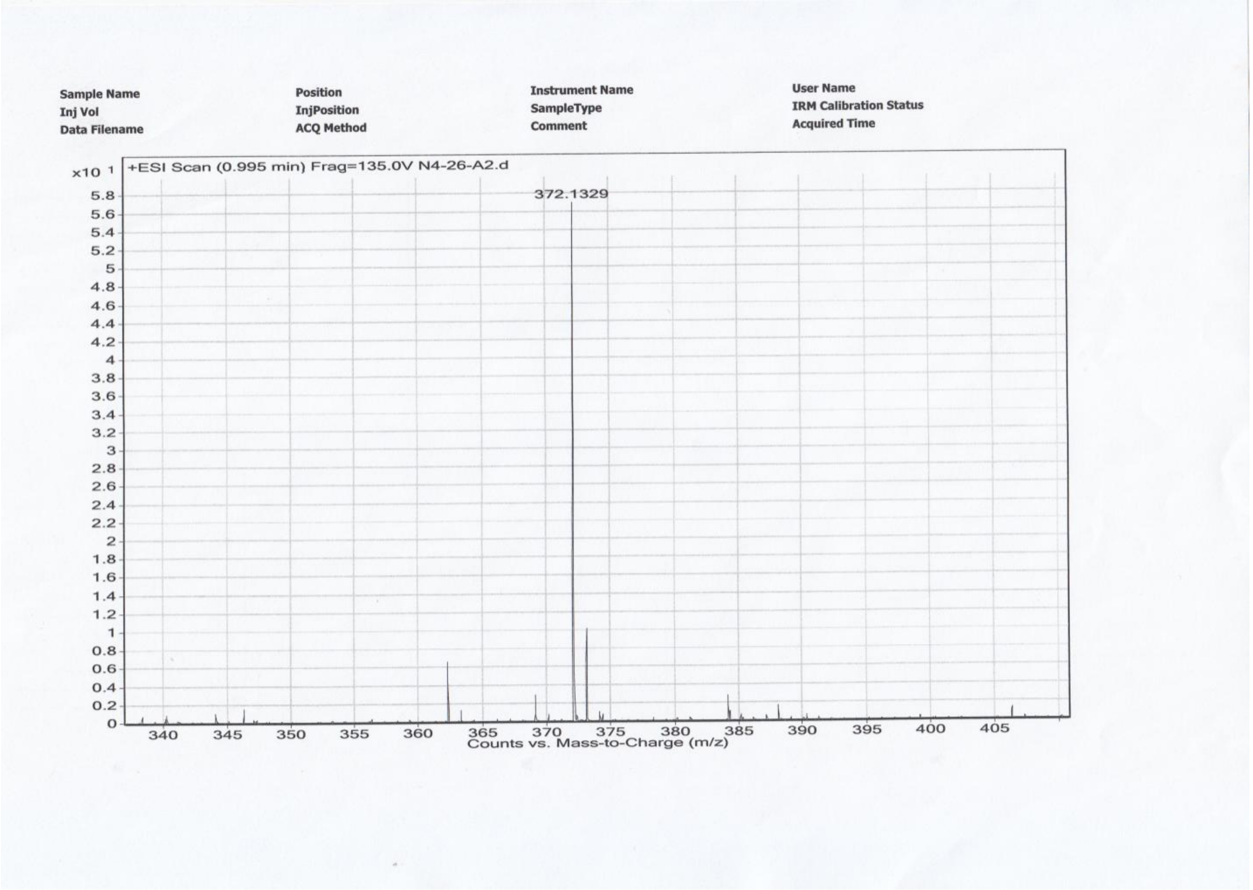

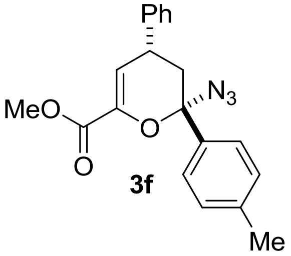

Chemical Formula: $C_{20}H_{19}N_3O_3$
Exact Mass: 349.1426
Molecular Weight: 349.3832
m/z: 349.1426 (100.0%), 350.1460 (21.6%), 351.1494 (2.2%), 350.1397 (1.1%)

HRMS exact mass calcd for $C_{20}H_{19}N_3NaO_3$ $[M + Na]^+$ **372.1324**, found **372.1329**.

Supplementary Figure 59. ^{1}H and ^{13}C NMR spectra for 3g

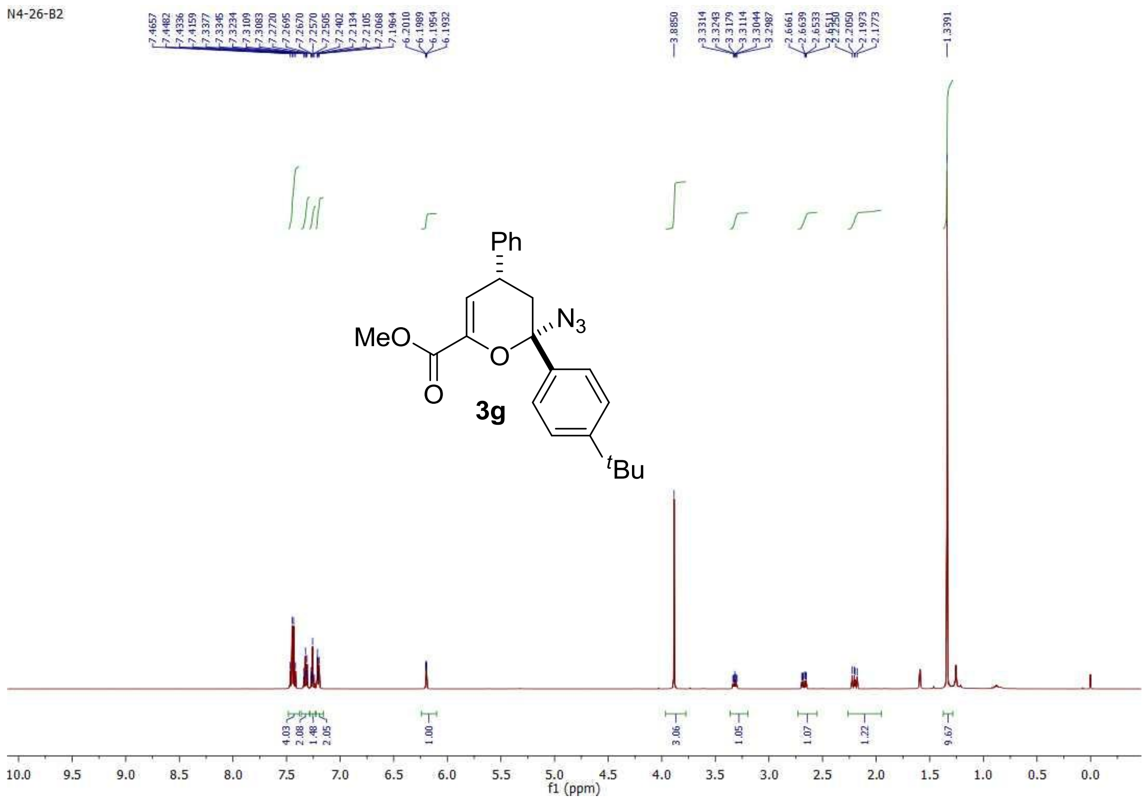

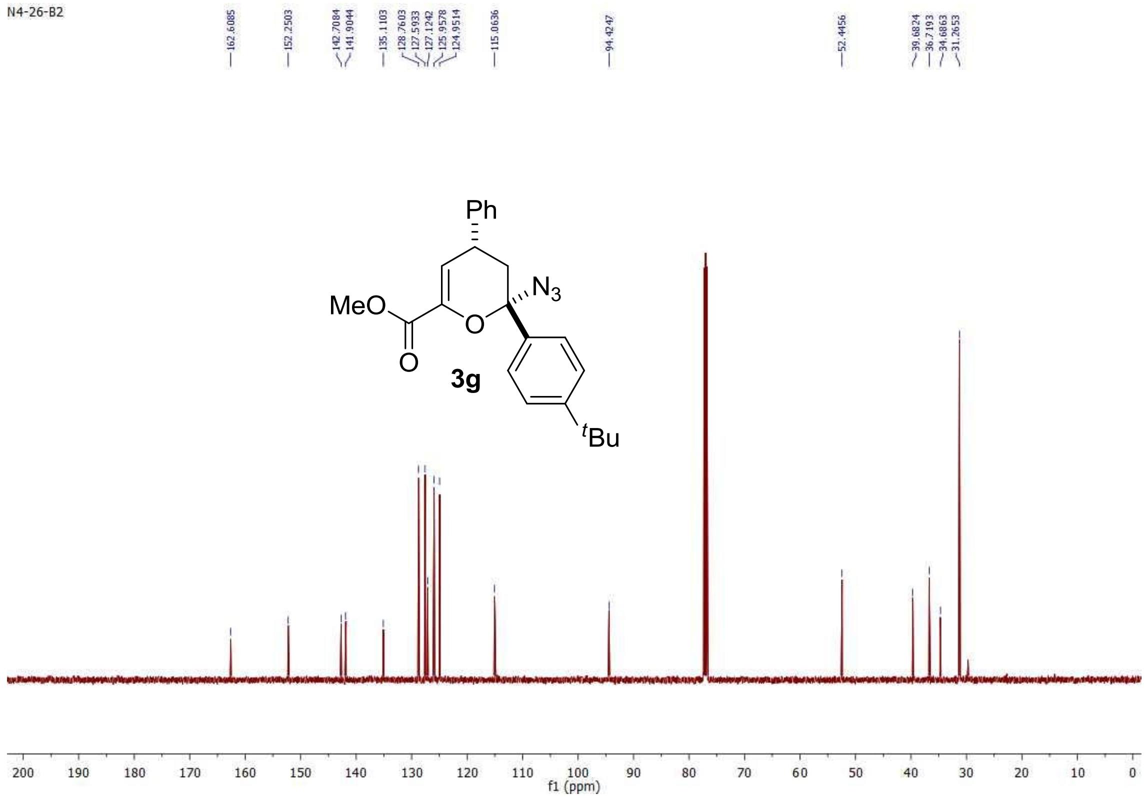

Supplementary Figure 60. HRMS spectra for 3g

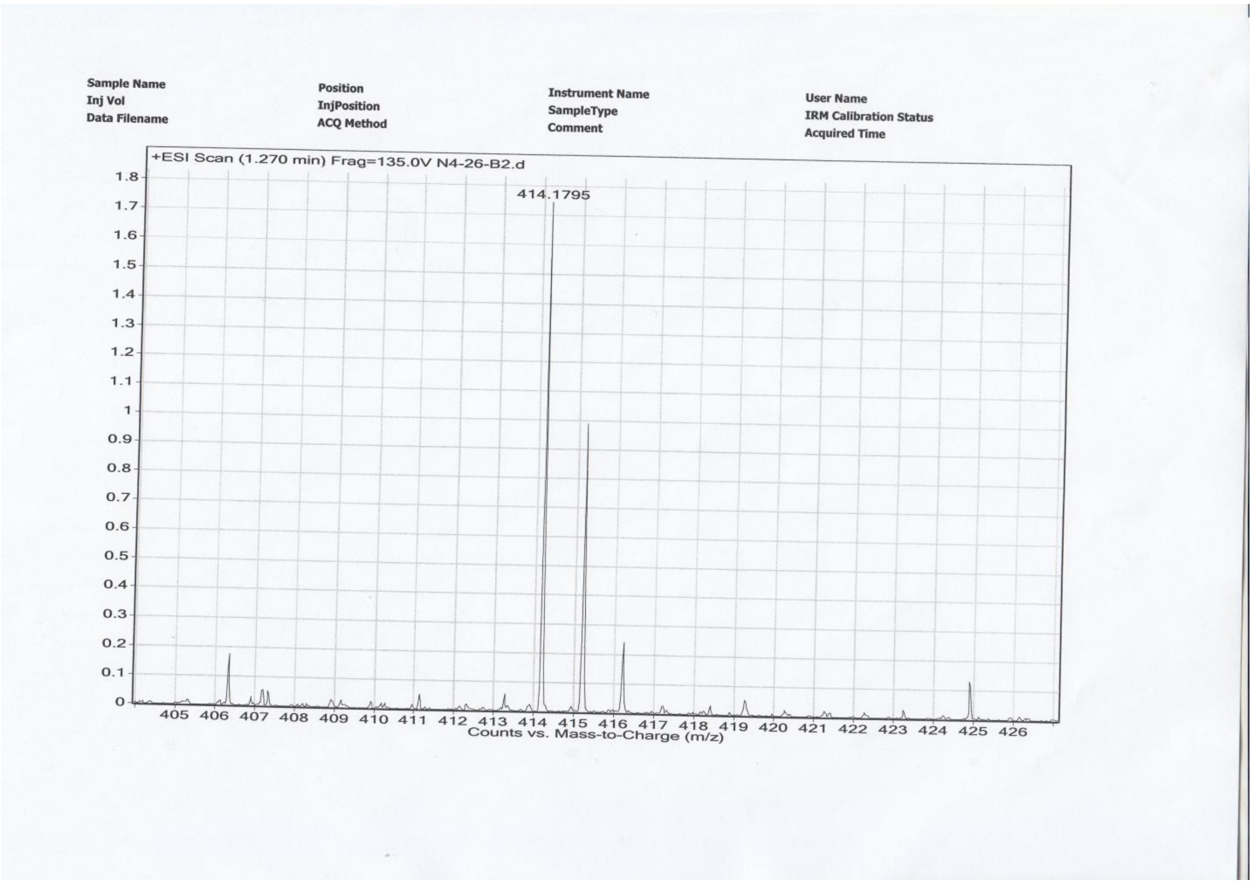

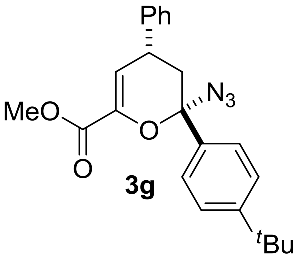

3g

Chemical Formula: $C_{23}H_{25}N_3O_3$
Exact Mass: 391.1896
Molecular Weight: 391.4629
m/z: 391.1896 (100.0%), 392.1929 (24.9%), 393.1963 (3.0%), 392.1866 (1.1%)

HRMS exact mass calcd for $C_{23}H_{25}N_3NaO_3$ $[M + Na]^+$ **414.1794**, found **414.1795**.

Supplementary Figure 61. ^{1}H and ^{13}C NMR spectra for 3h

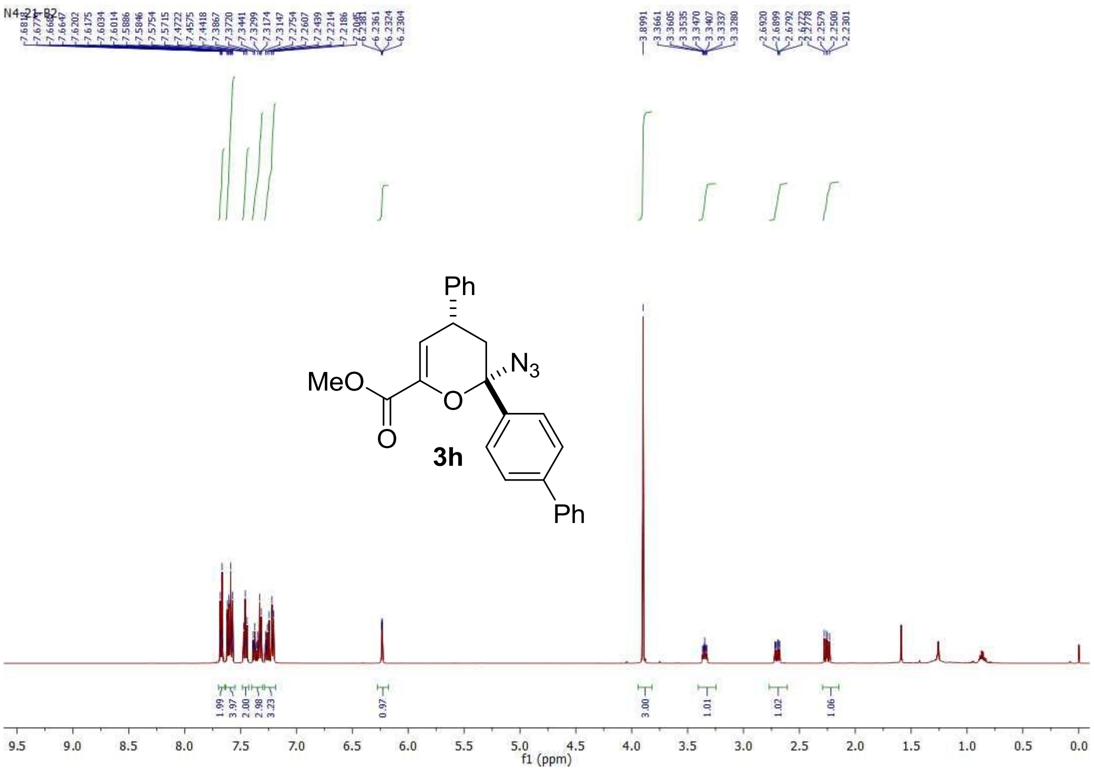

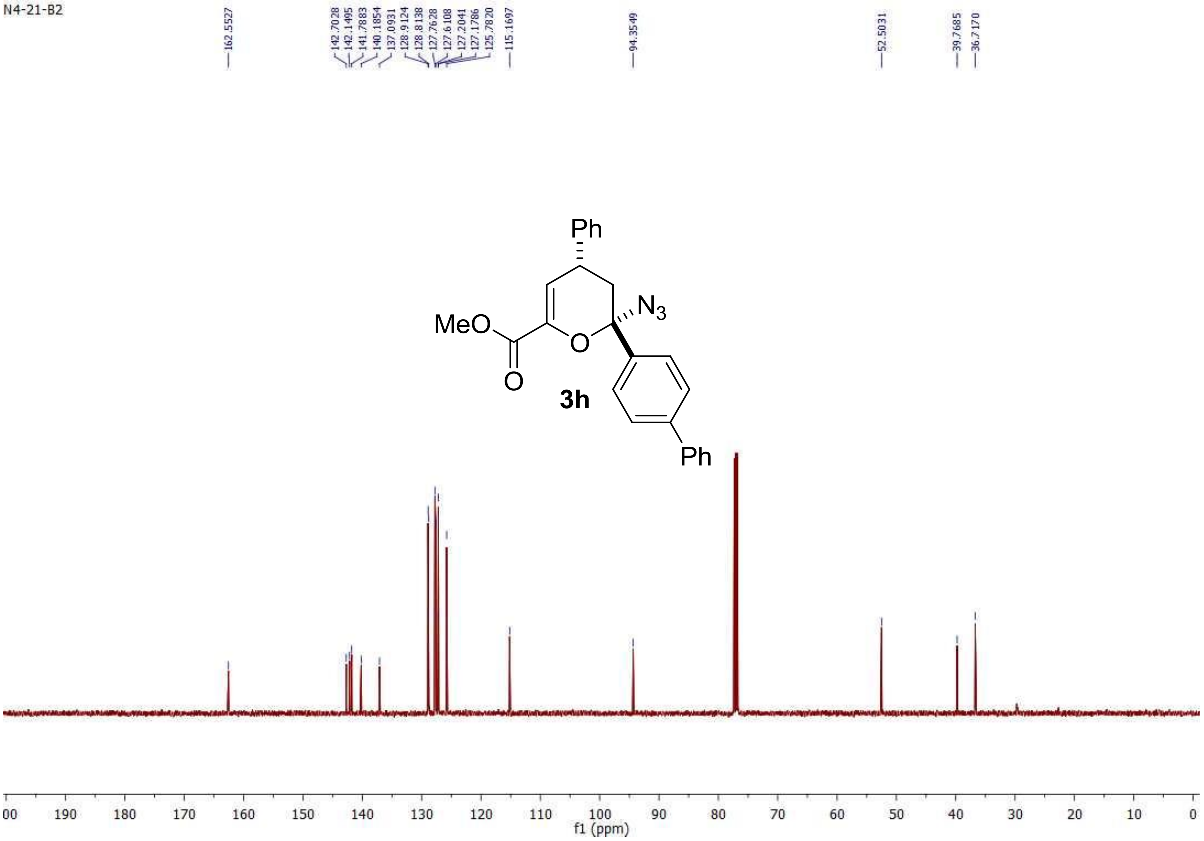

Supplementary Figure 62. HRMS spectra for 3h

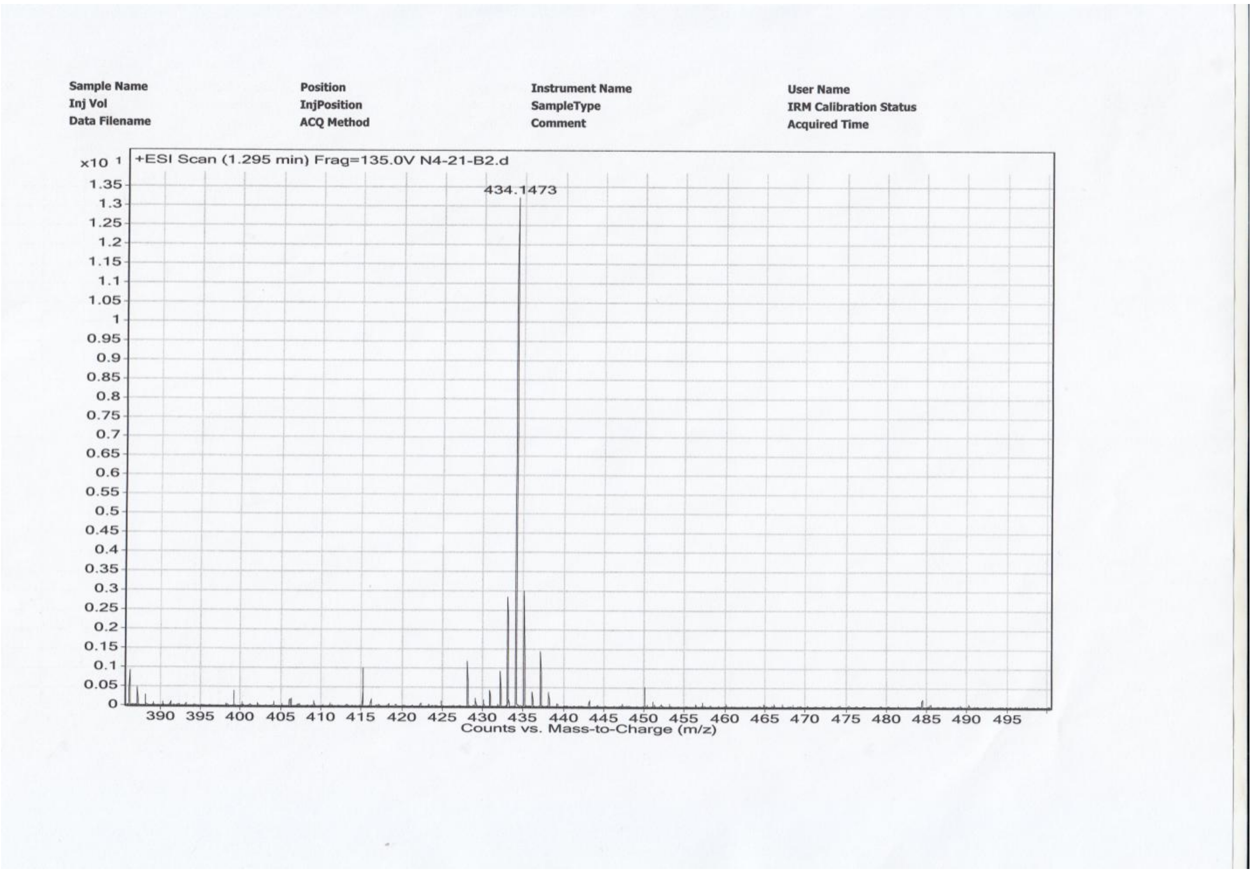

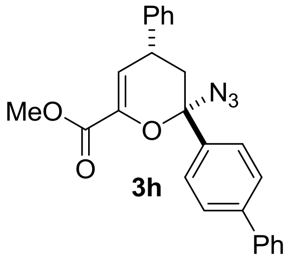

Chemical Formula: $C_{25}H_{21}N_3O_3$
Exact Mass: 411.1583
Molecular Weight: 411.4525
m/z: 411.1583 (100.0%), 412.1616 (27.0%), 413.1650 (3.5%), 412.1553 (1.1%)

HRMS exact mass calcd for $C_{25}H_{21}N_3NaO_3$ $[M + Na]^+$ **434.1481**, found **434.1473**.

Supplementary Figure 63. ^{1}H and ^{13}C NMR spectra for 3i

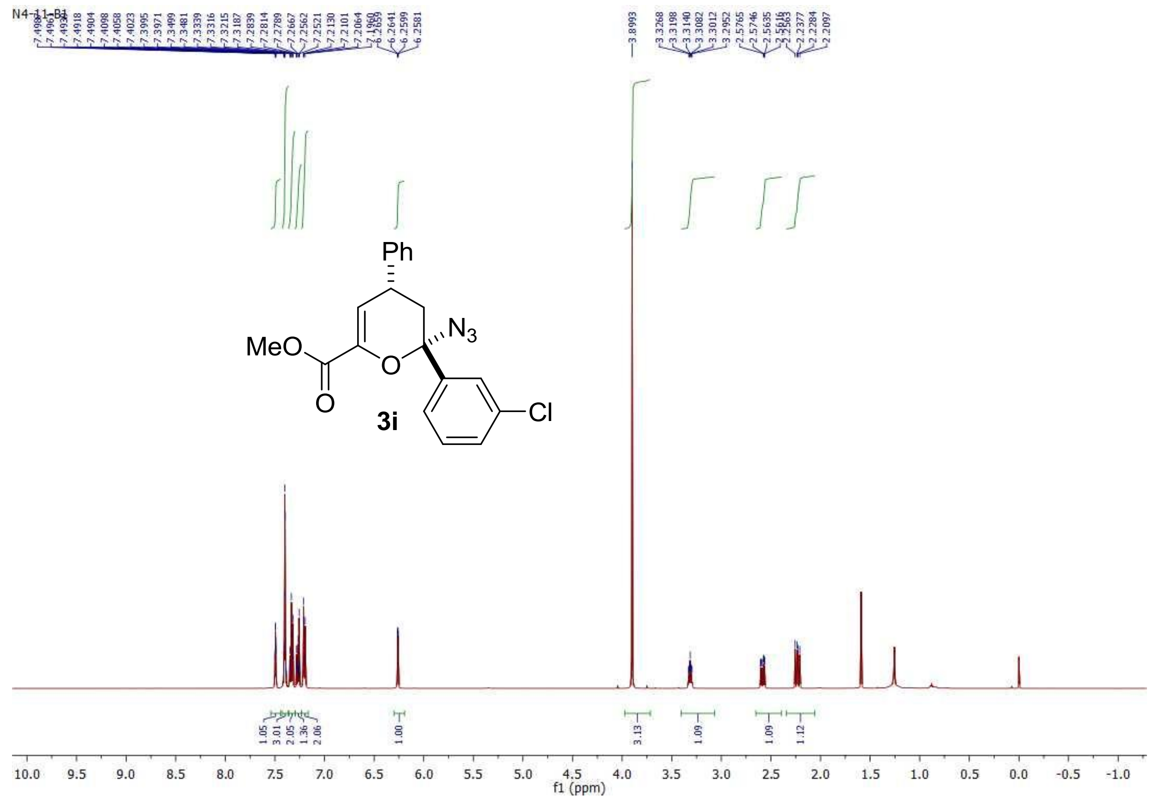

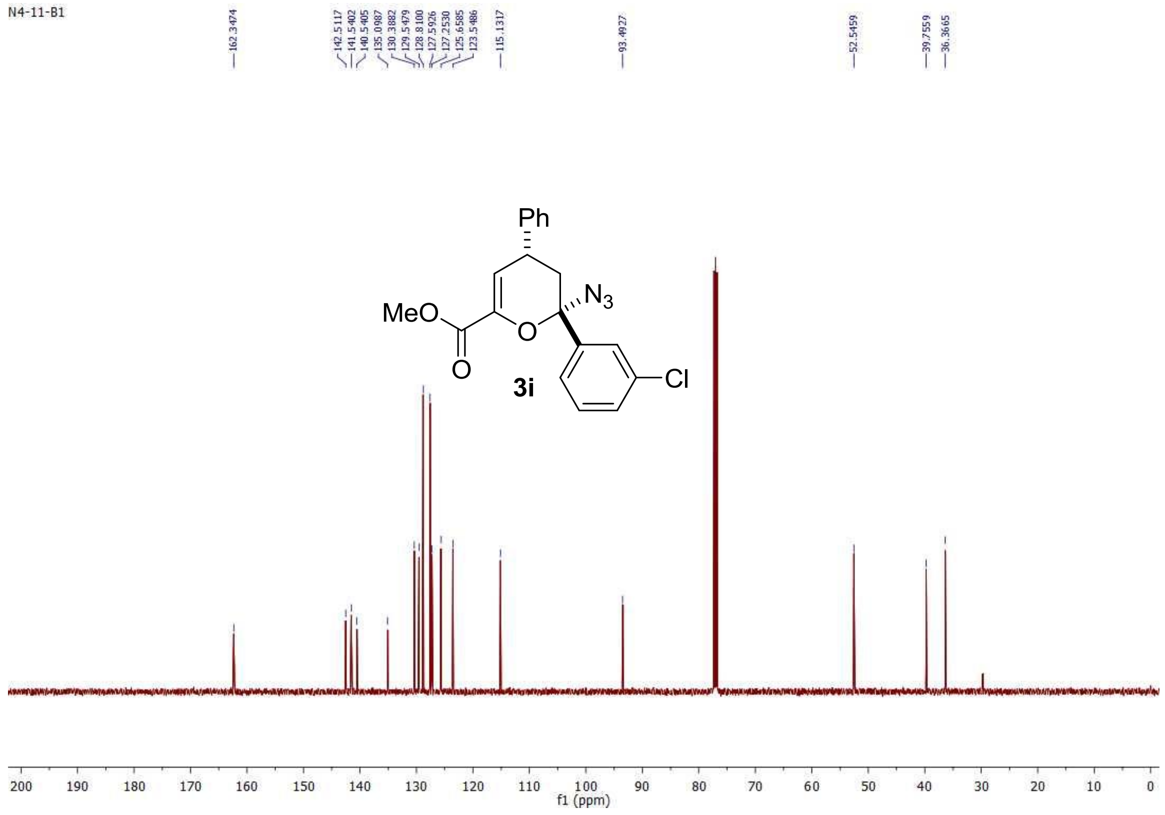

Supplementary Figure 64. HRMS spectra for 3i

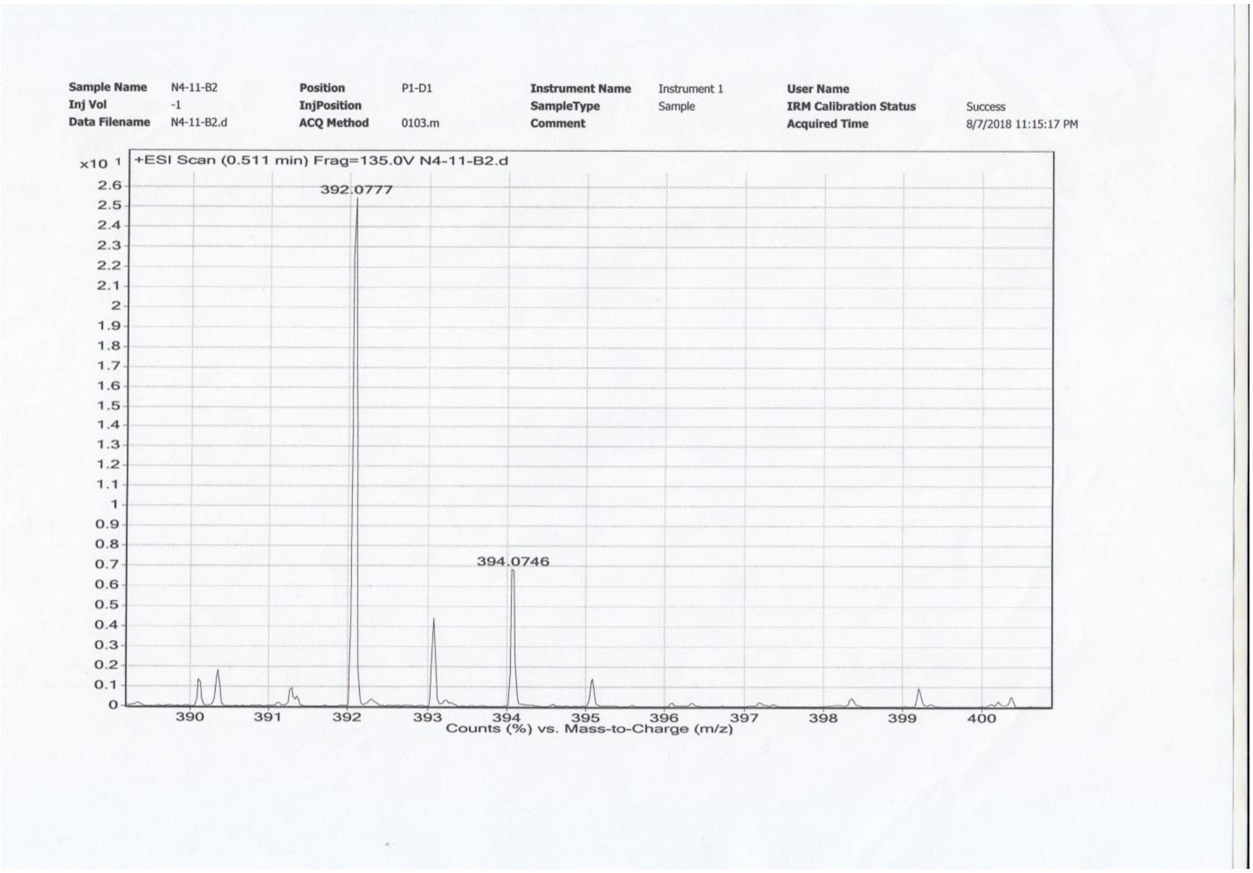

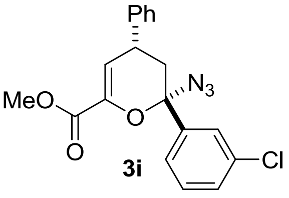

Chemical Formula: $C_{19}H_{16}ClN_3O_3$
Exact Mass: 369.0880
Molecular Weight: 369.8016
m/z: 369.0880 (100.0%), 371.0851 (32.0%), 370.0914 (20.5%), 372.0884 (6.6%), 371.0947 (2.0%), 370.0851 (1.1%)

HRMS exact mass calcd for $C_{19}H_{16}ClN_3NaO_3$ $[M + Na]^+$ **392.0778**, found **392.0777**.

Supplementary Figure 65. ^{1}H and ^{13}C NMR spectra for 3j

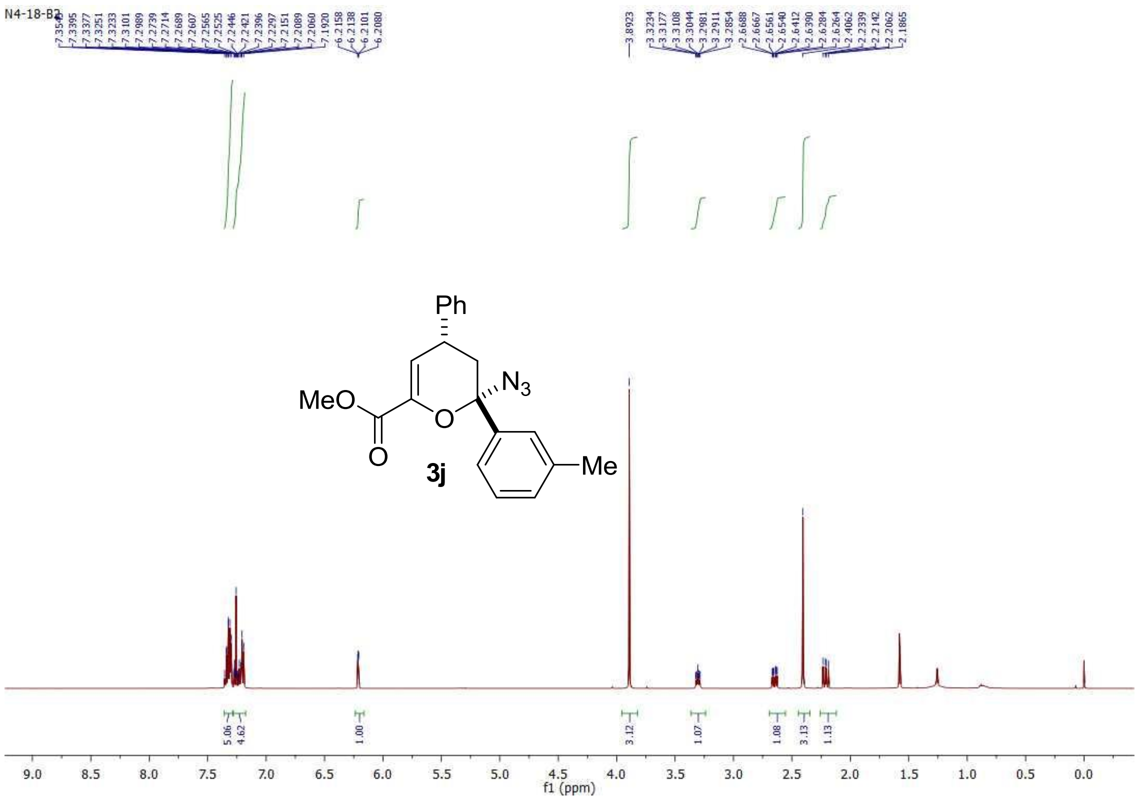

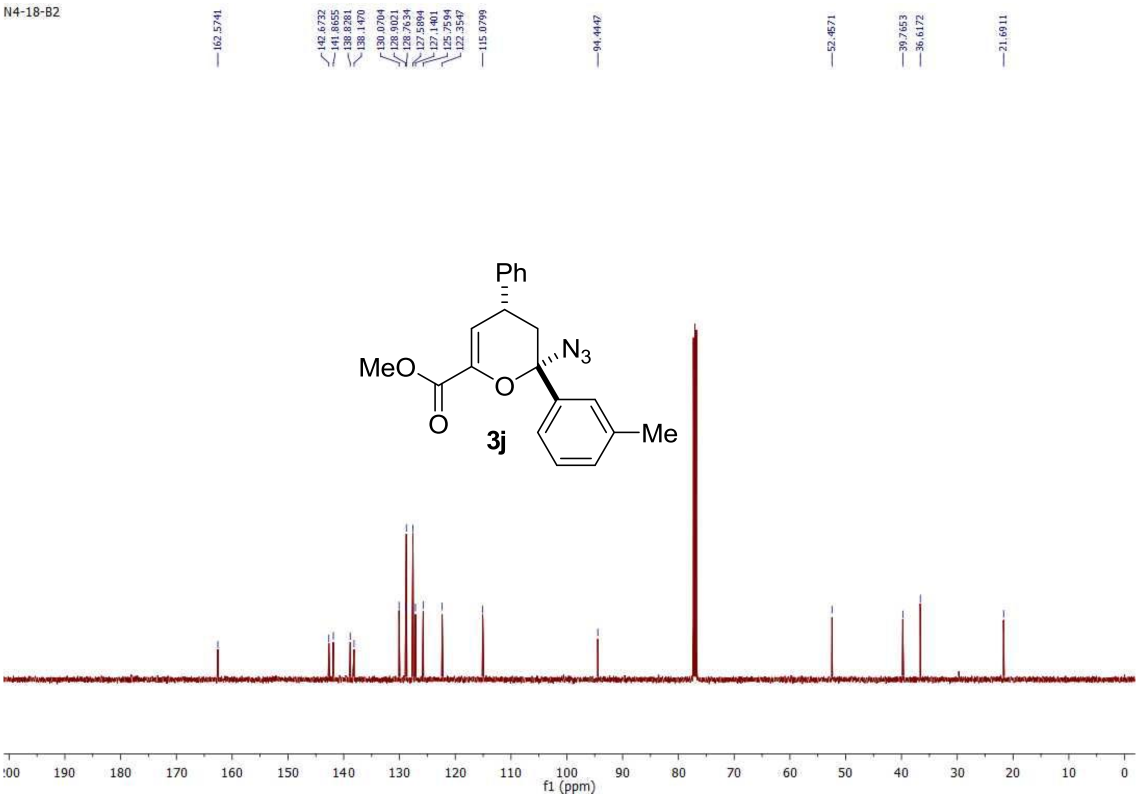

Supplementary Figure 66. HRMS spectra for 3j

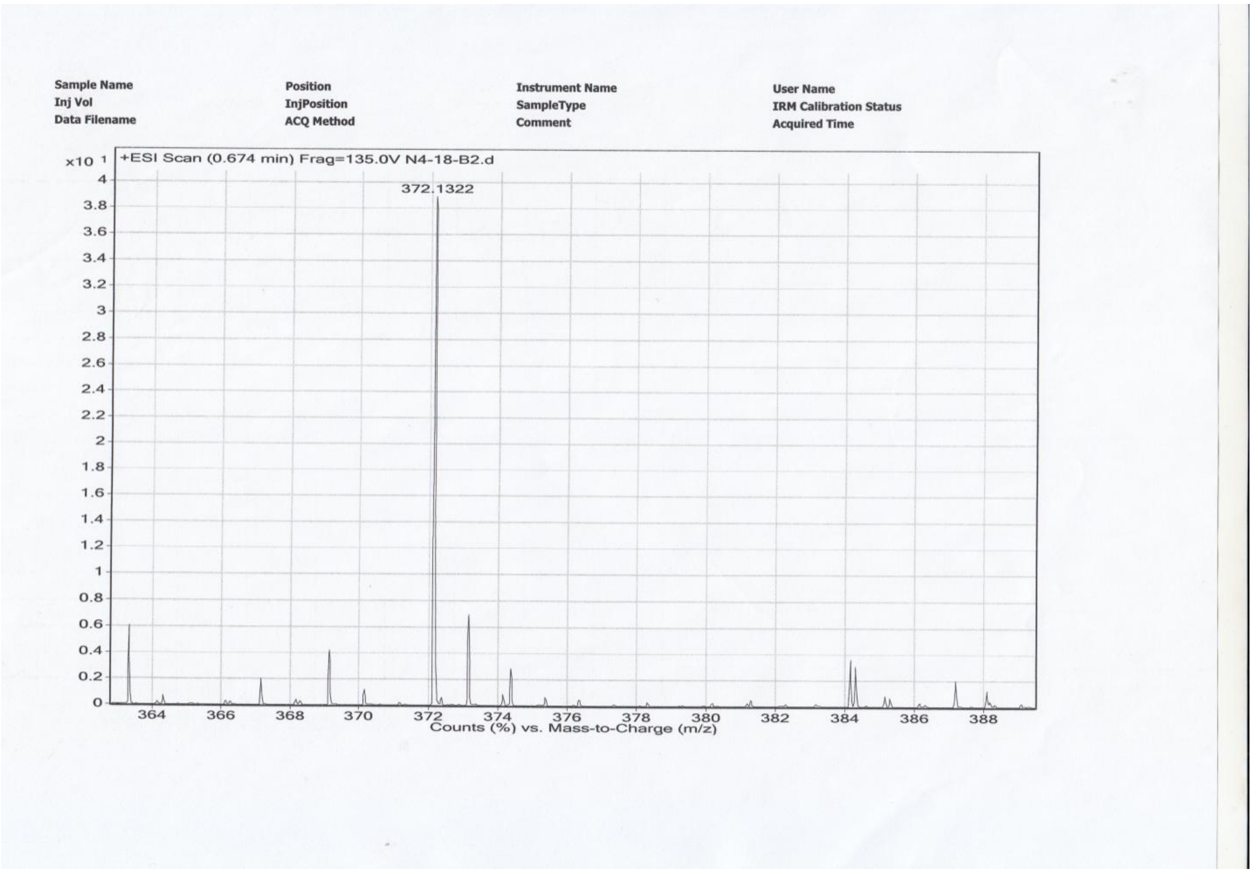

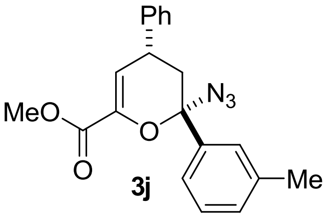

3j

Chemical Formula: $C_{20}H_{19}N_3O_3$
Exact Mass: 349.1426
Molecular Weight: 349.3832
m/z: 349.1426 (100.0%), 350.1460 (21.6%), 351.1494 (2.2%), 350.1397 (1.1%)

HRMS exact mass calcd for $C_{20}H_{19}N_3NaO_3$ $[M + Na]^+$ **372.1324**, found **372.1322**.

Supplementary Figure 67. ^{1}H and ^{13}C NMR spectra for 3k

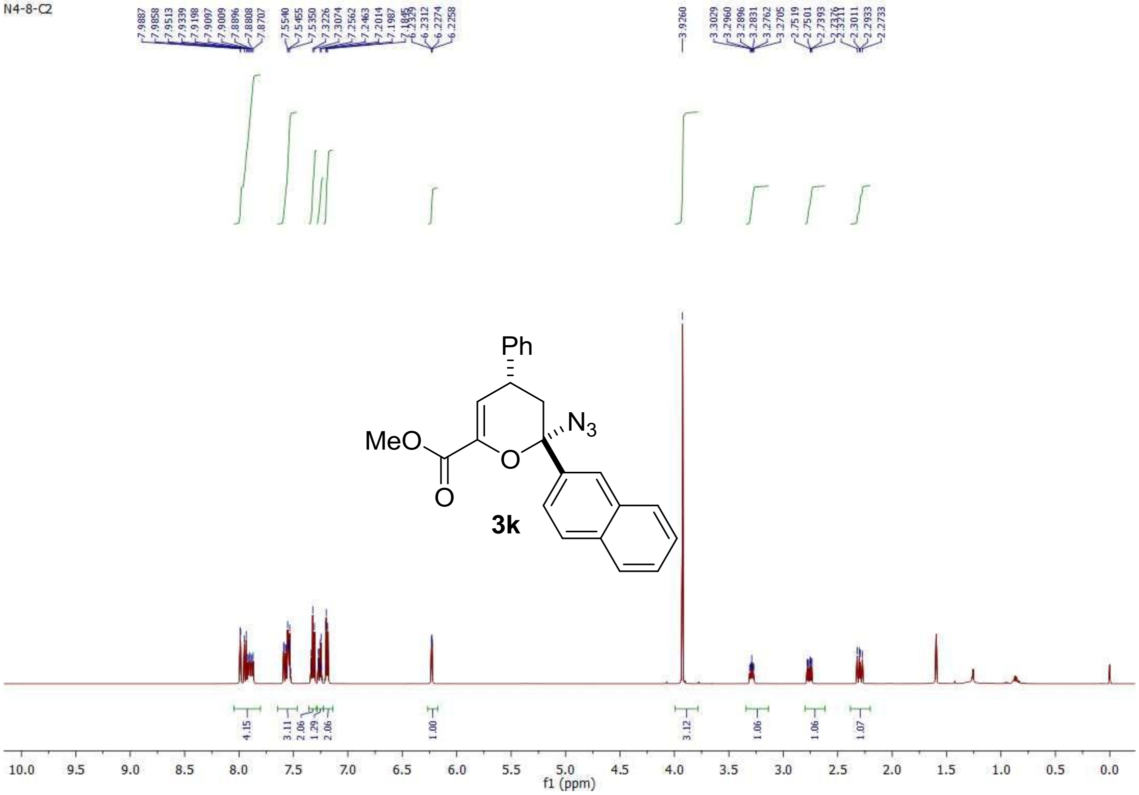

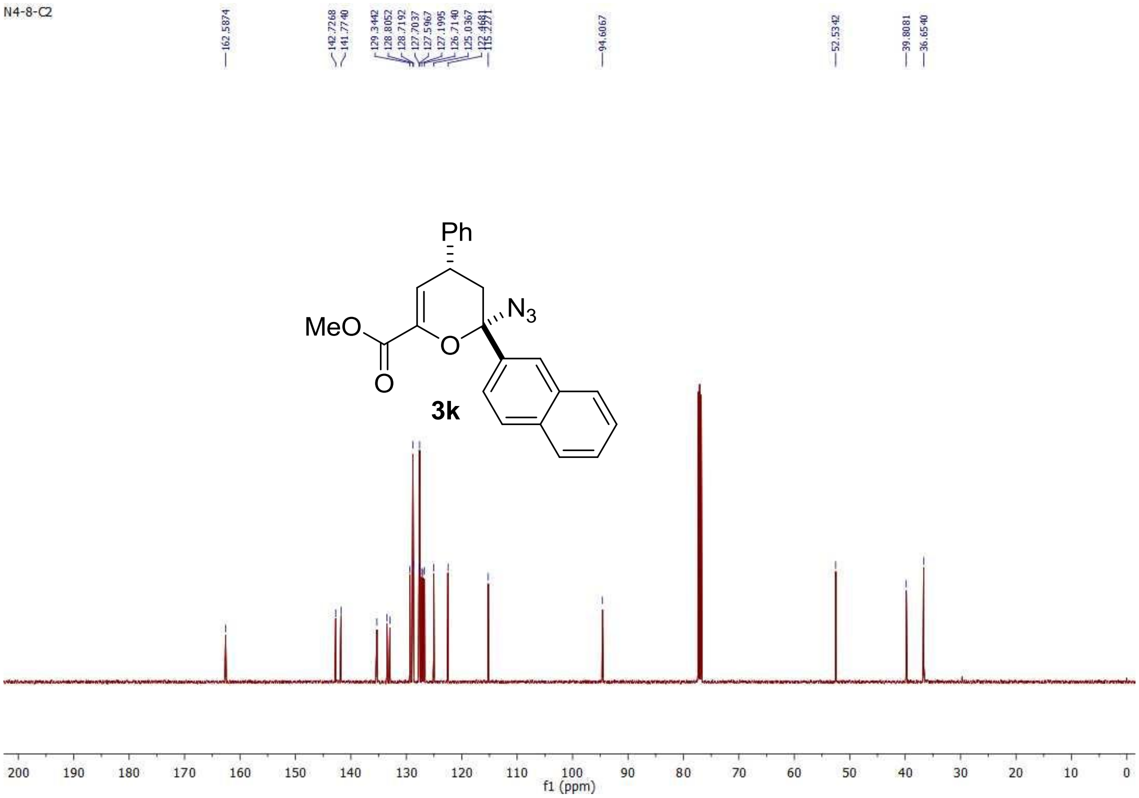

Supplementary Figure 68. HRMS spectra for 3k

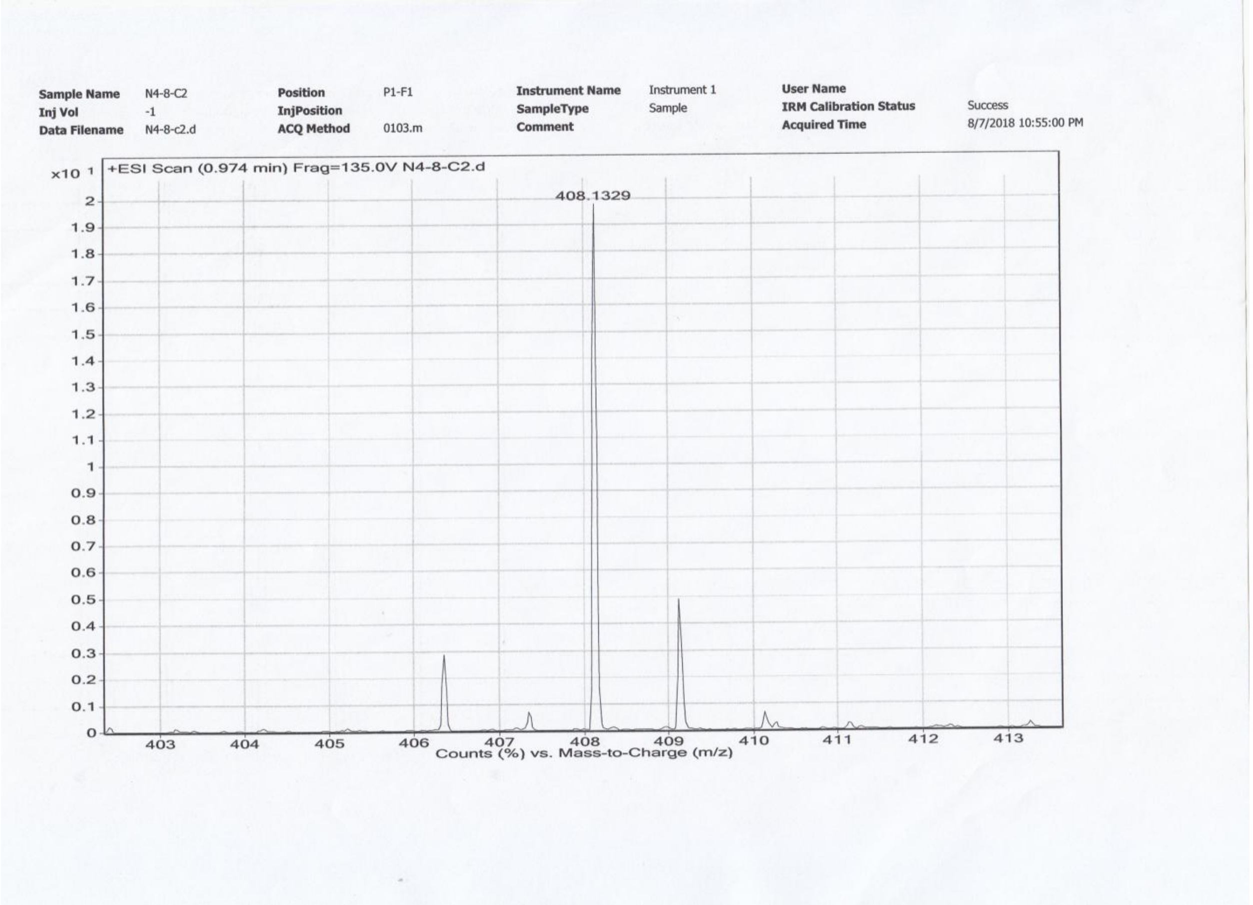

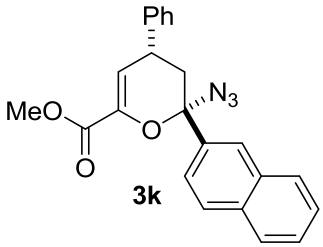

3k

Chemical Formula: $C_{23}H_{19}N_3O_3$
Exact Mass: 385.1426
Molecular Weight: 385.4153
m/z: 385.1426 (100.0%), 386.1460 (24.9%), 387.1494 (3.0%), 386.1397 (1.1%)

HRMS exact mass calcd for $C_{23}H_{19}N_3NaO_3$ $[M + Na]^+$ **408.1324**, found **408.1329**.

Supplementary Figure 69. ^{1}H and ^{13}C NMR spectra for 3l

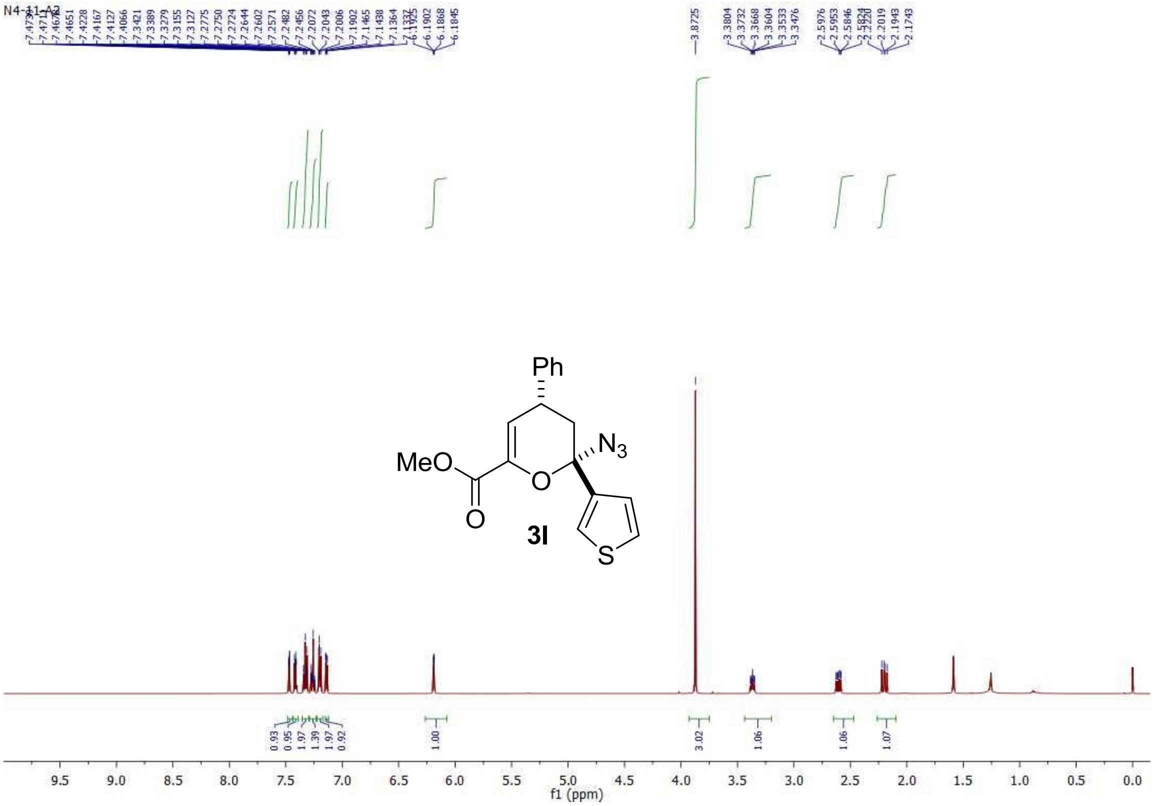

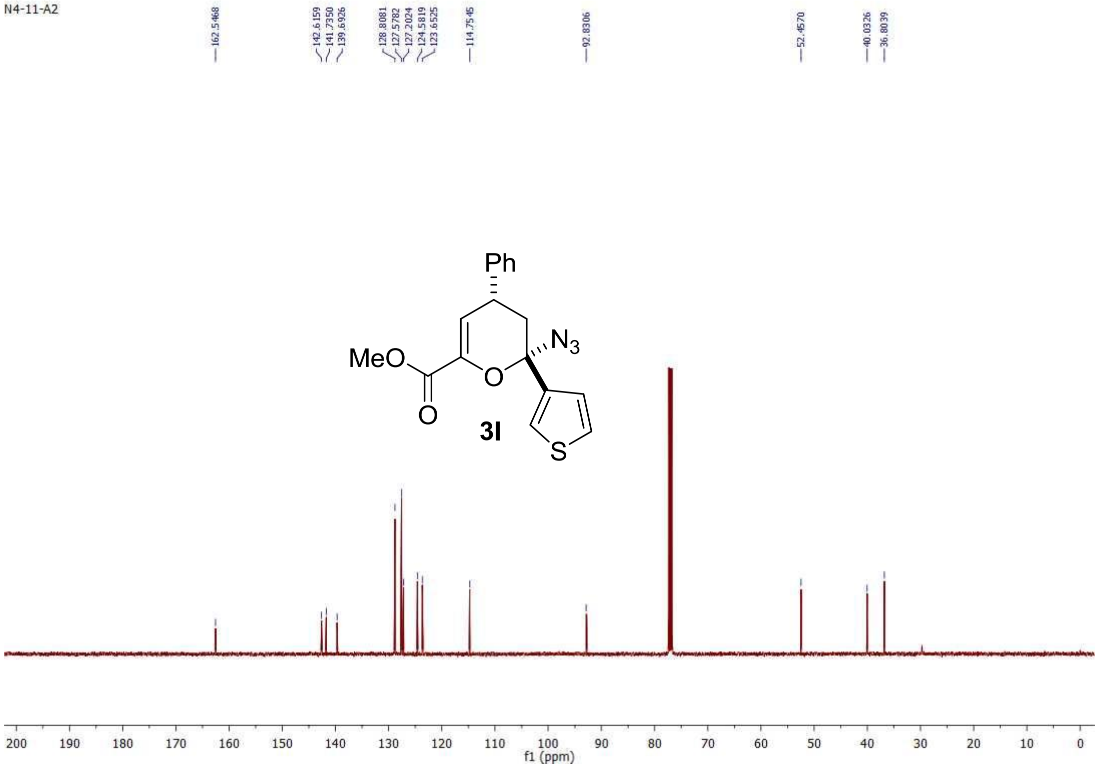

Supplementary Figure 70. HRMS spectra for 3l

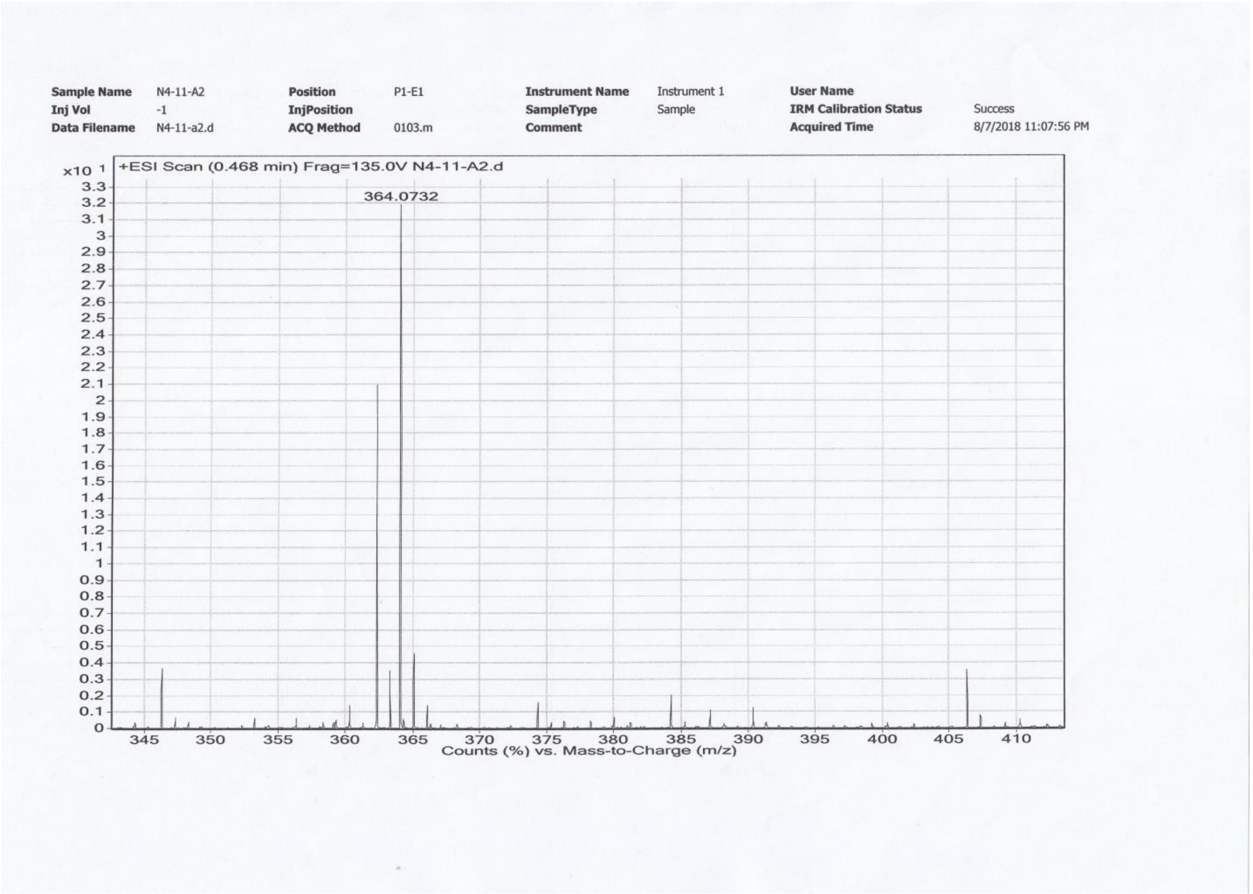

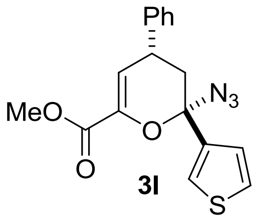

3l

Chemical Formula: $C_{17}H_{15}N_3O_3S$

Exact Mass: 341.0834

Molecular Weight: 341.3843

m/z: 341.0834 (100.0%), 342.0868 (18.4%), 343.0792 (4.5%), 343.0901 (1.6%), 342.0804 (1.1%)

HRMS exact mass calcd for $C_{17}H_{15}N_3NaO_3S$ $[M + Na]^+$ **364.0732**, found **364.0732**.

Supplementary Figure 71. ^{1}H and ^{13}C NMR spectra for 3m

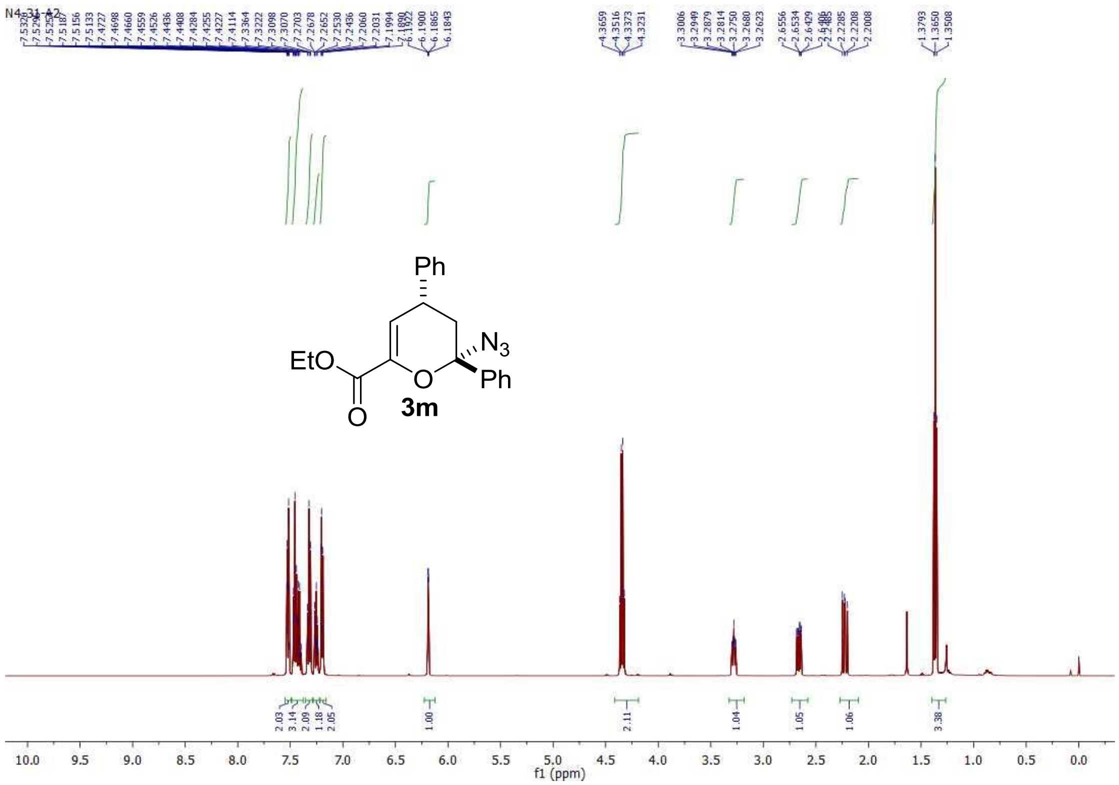

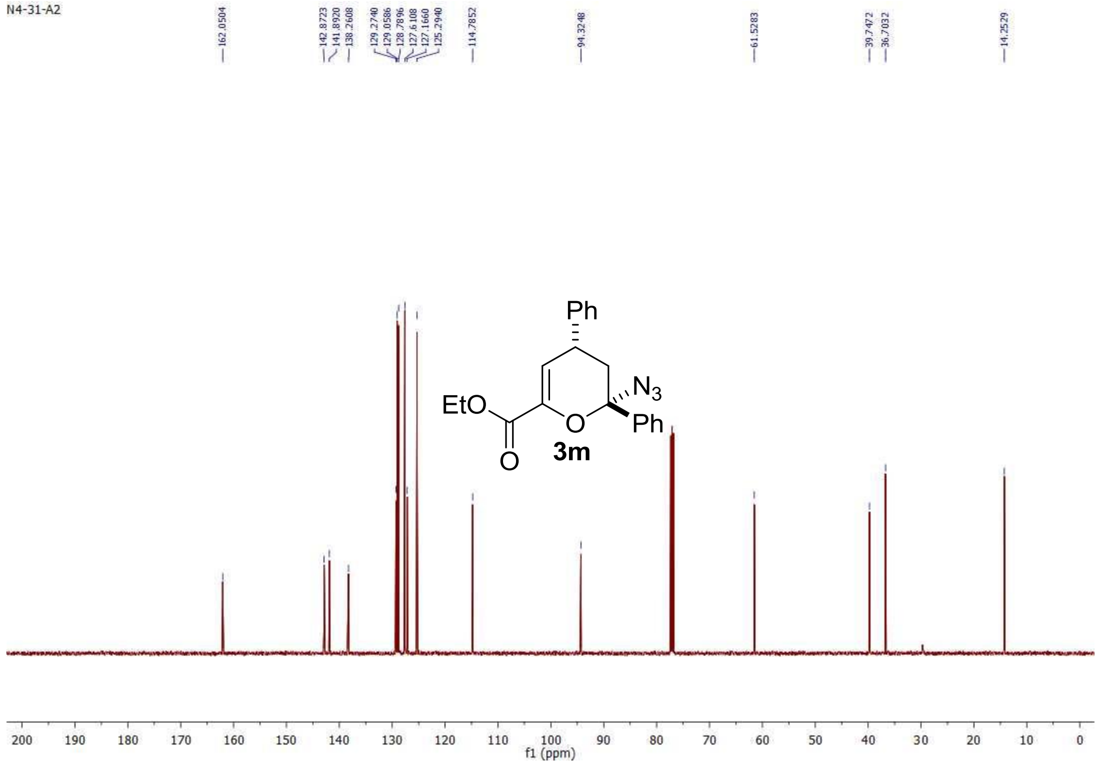

Supplementary Figure 72. HRMS spectra for 3m

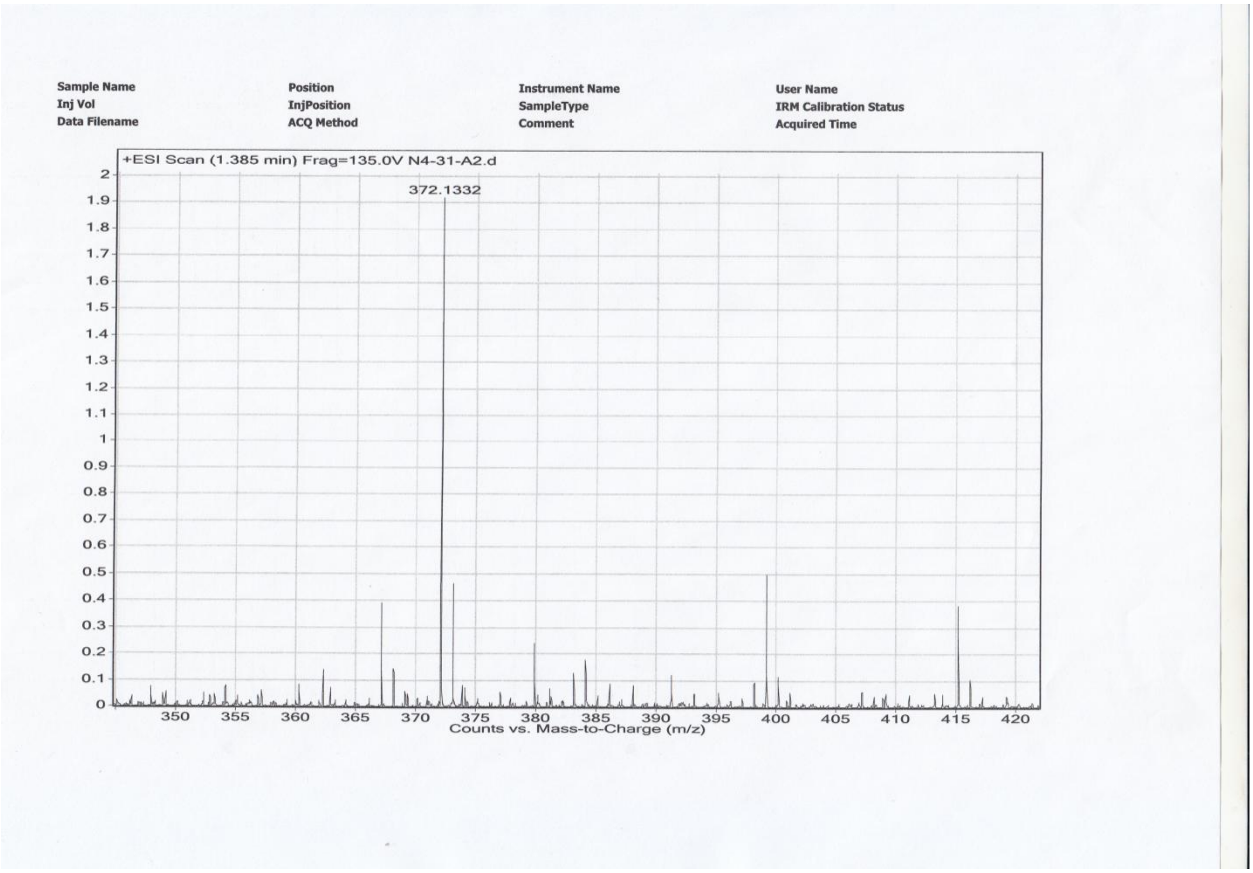

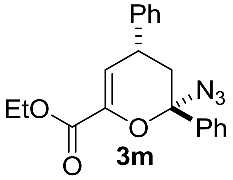

3m

Chemical Formula: $C_{20}H_{19}N_3O_3$
Exact Mass: 349.1426
Molecular Weight: 349.3832
m/z: 349.1426 (100.0%), 350.1460 (21.6%), 351.1494 (2.2%), 350.1397 (1.1%)

HRMS exact mass calcd for $C_{20}H_{19}N_3NaO_3$ $[M + Na]^+$ **372.1324**, found **372.1332**.

Supplementary Figure 73. ^{1}H and ^{13}C NMR spectra for 3n

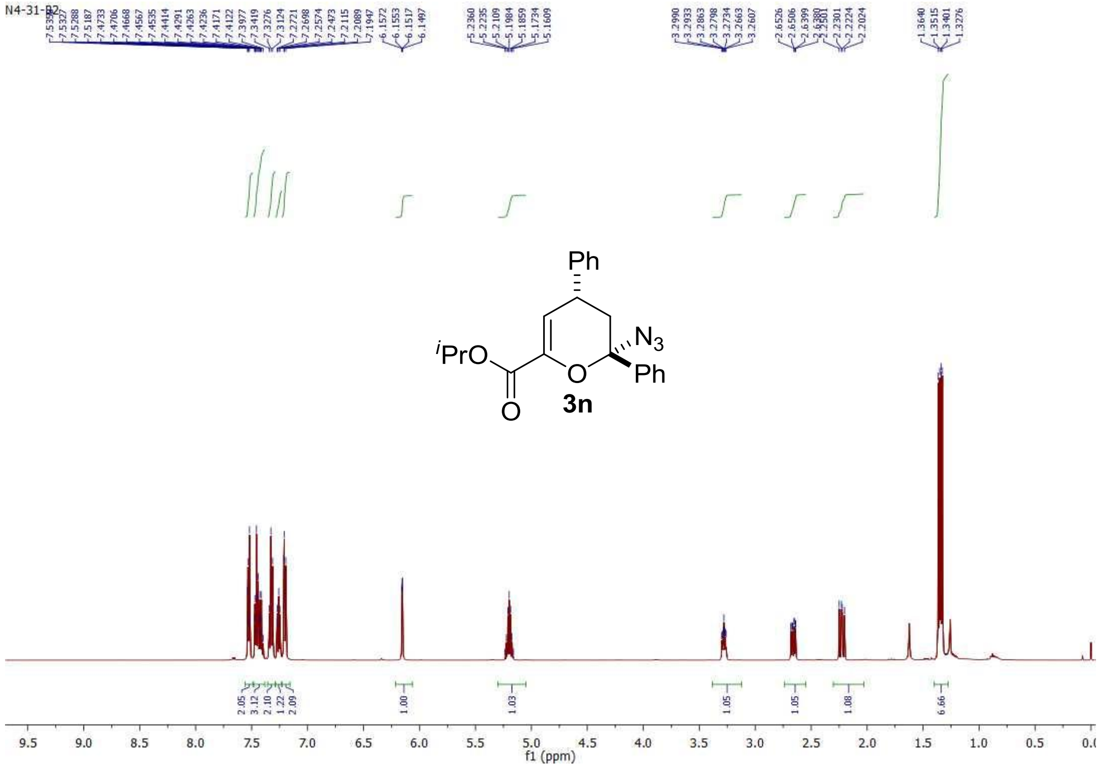

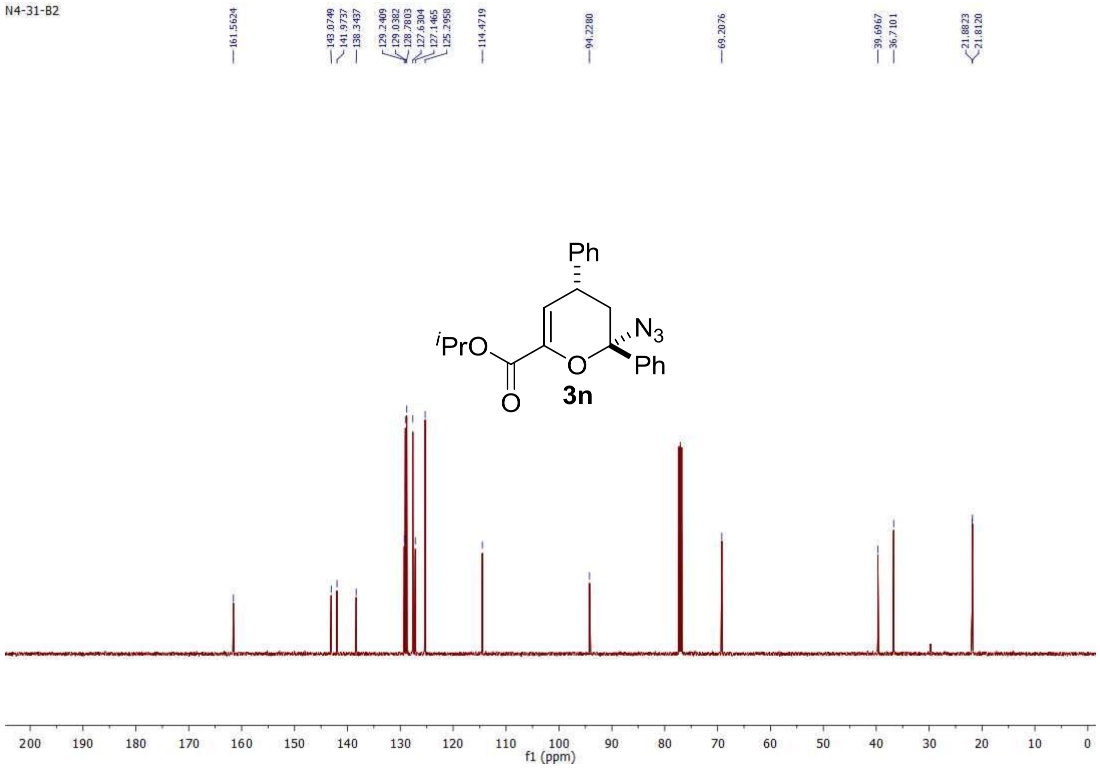

Supplementary Figure 74. HRMS spectra for 3n

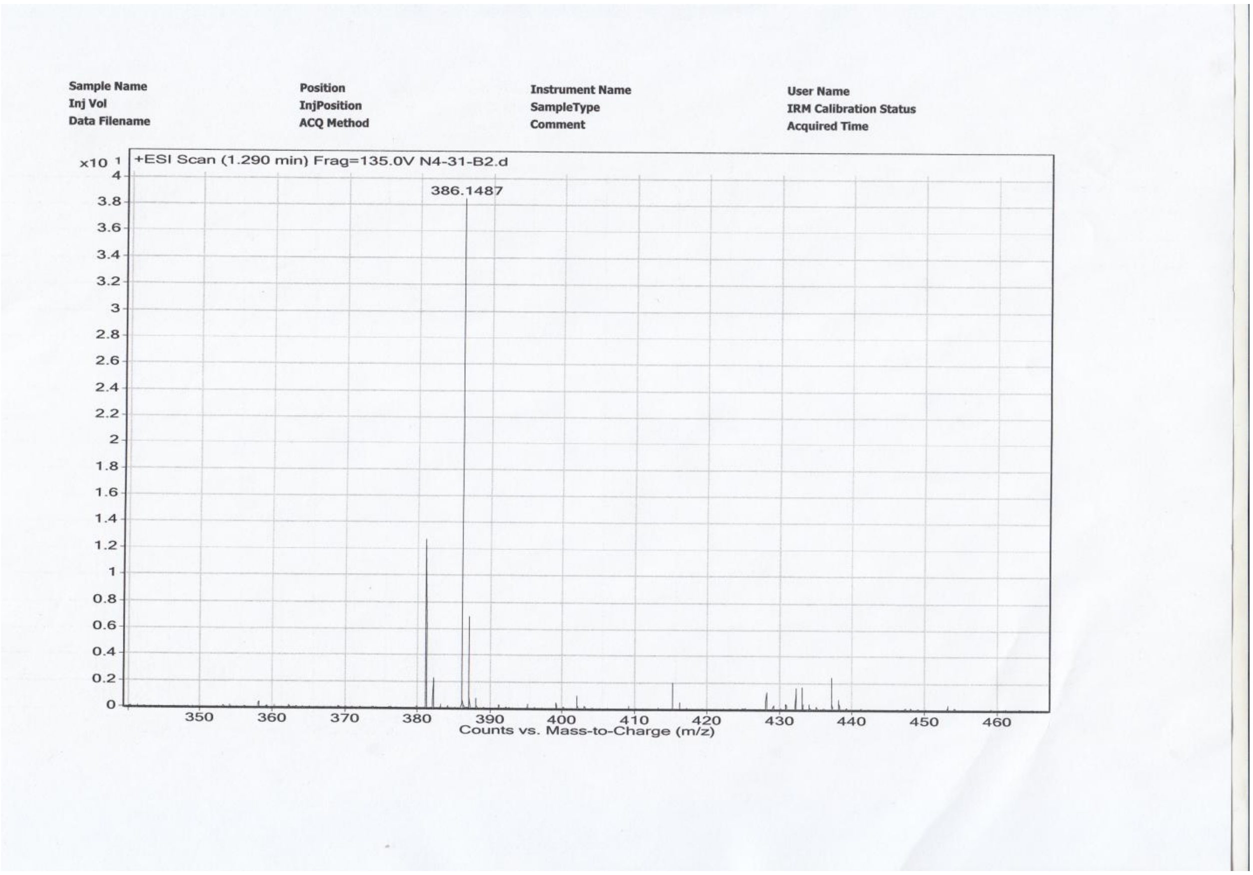

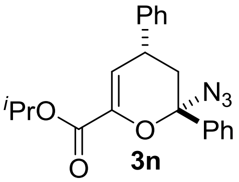

3n

Chemical Formula: $C_{21}H_{21}N_3O_3$
Exact Mass: 363.1583
Molecular Weight: 363.4097
m/z: 363.1583 (100.0%), 364.1616 (22.7%), 365.1650 (2.5%), 364.1553 (1.1%)

HRMS exact mass calcd for $C_{21}H_{21}N_3NaO_3$ $[M + Na]^+$ **386.1481**, found **386.1487**.

Supplementary Figure 75. ^{1}H and ^{13}C NMR spectra for 3o

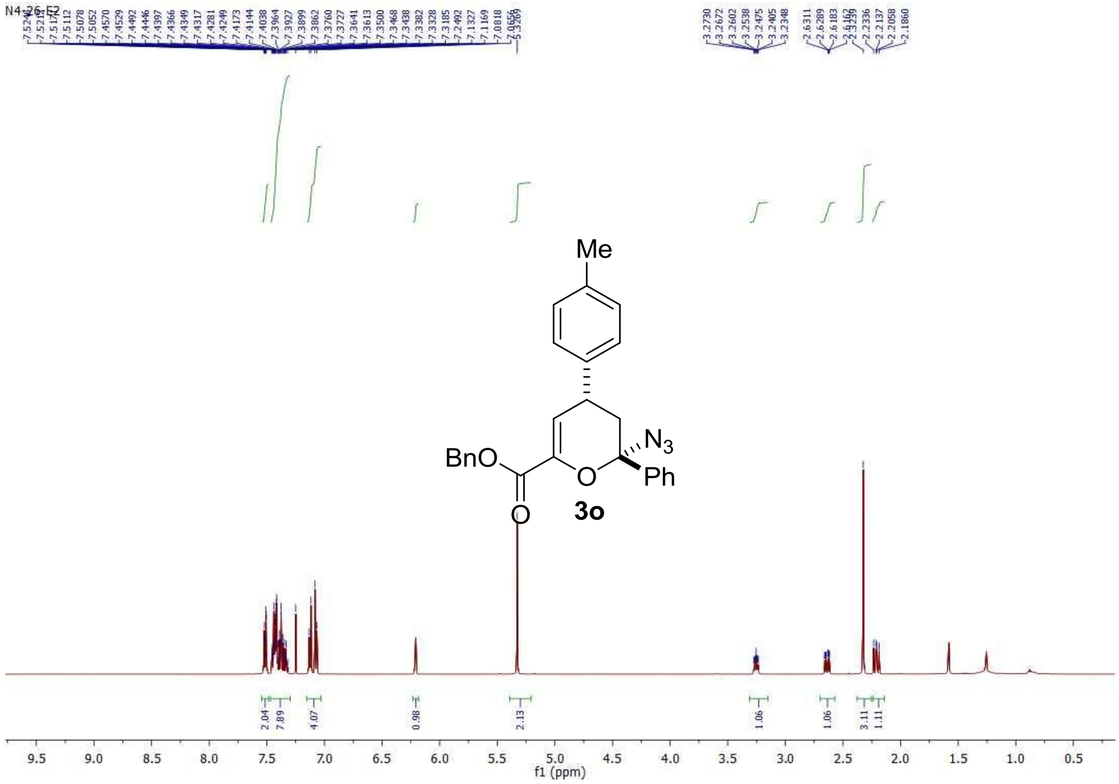

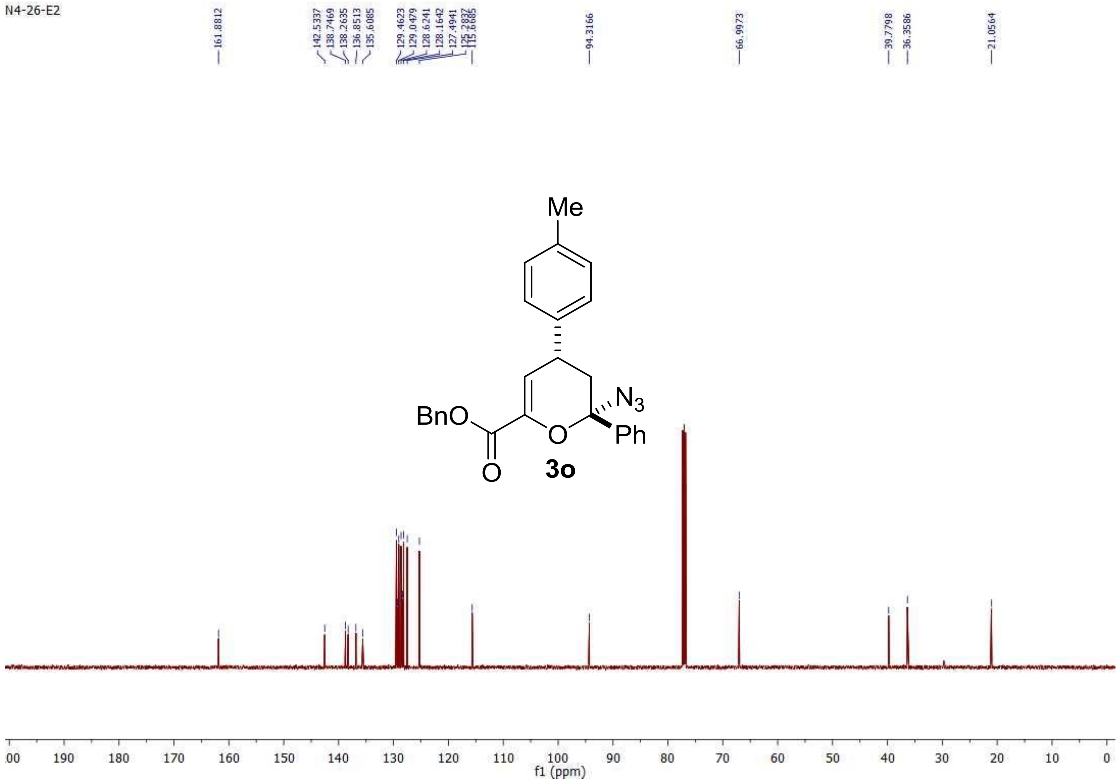

Supplementary Figure 76. HRMS spectra for 3o

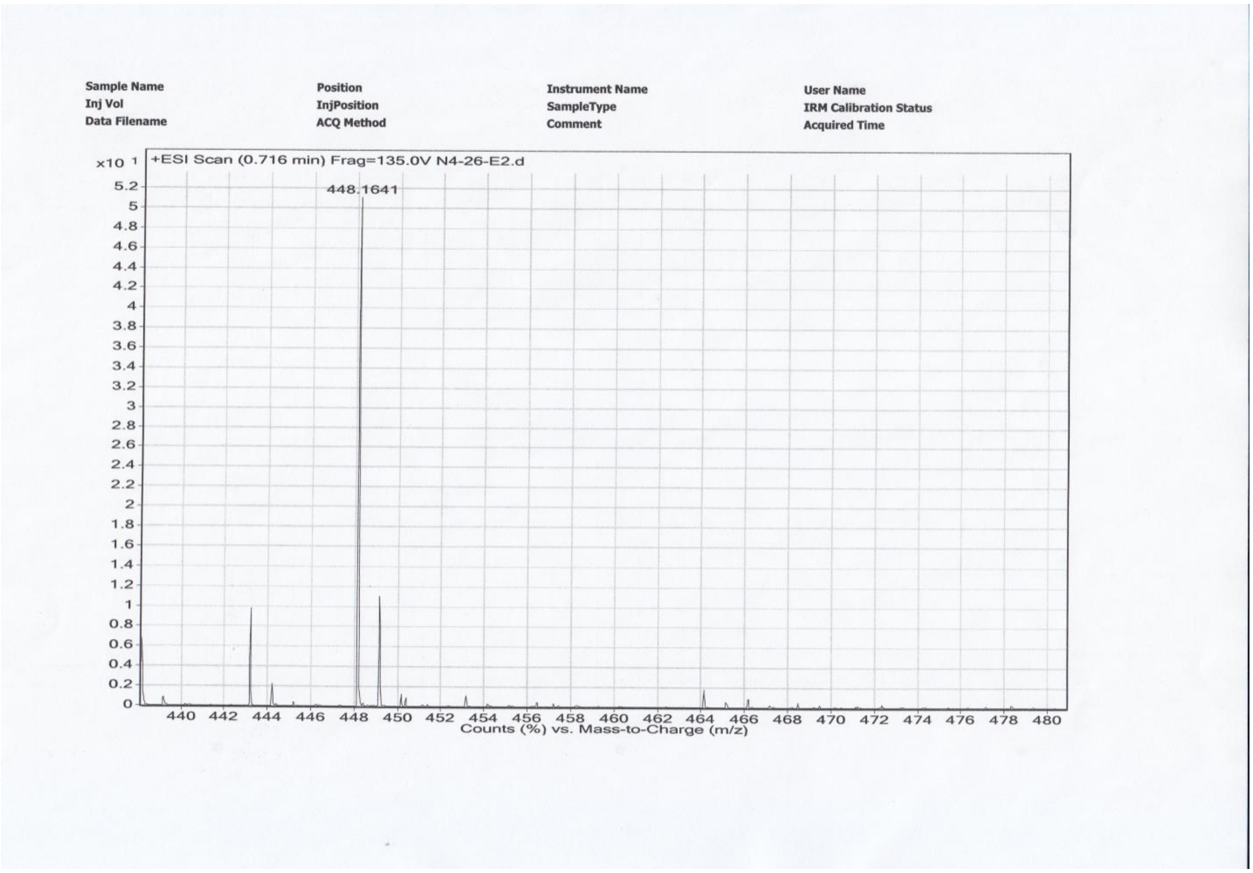

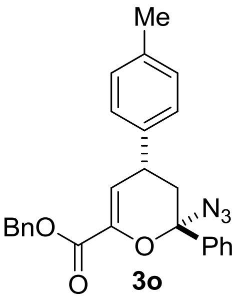

3o

Chemical Formula: $C_{26}H_{23}N_3O_3$
Exact Mass: 425.1739
Molecular Weight: 425.4791
m/z: 425.1739 (100.0%), 426.1773 (28.1%), 427.1807 (3.8%), 426.1710 (1.1%)

HRMS exact mass calcd for $C_{26}H_{23}N_3NaO_3$ $[M + Na]^+$ **448.1637**, found **448.1641**.

Supplementary Figure 77. ^{1}H and ^{13}C NMR spectra for 3p

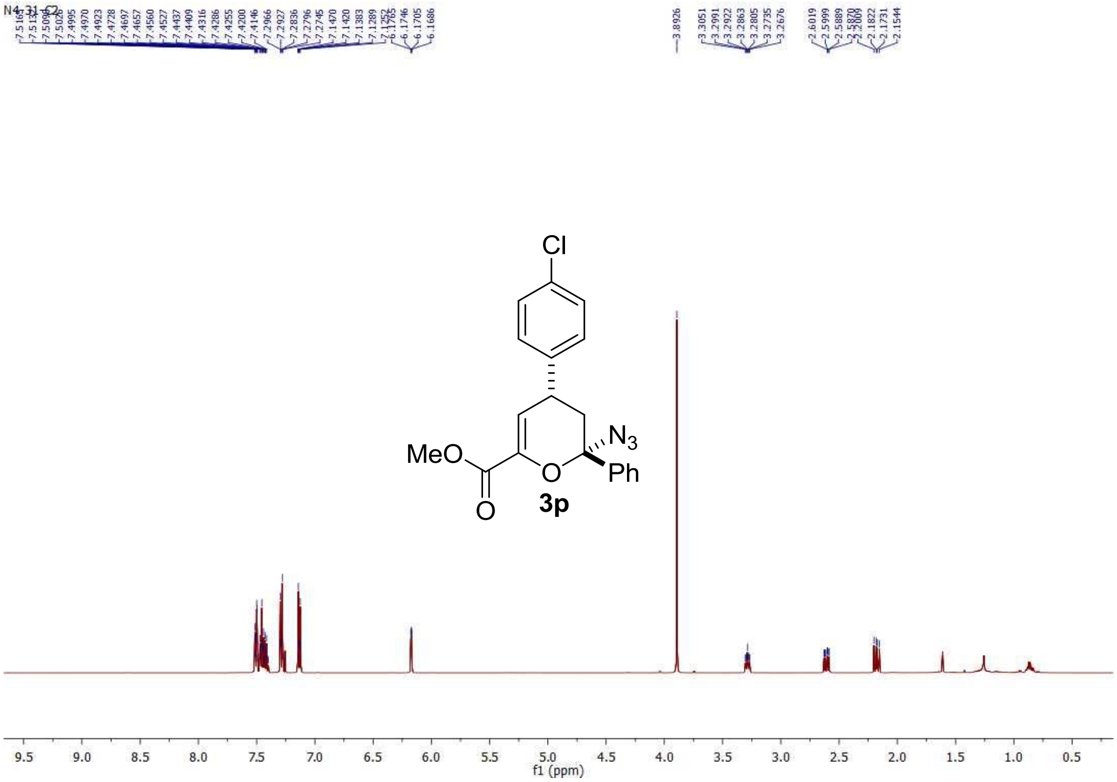

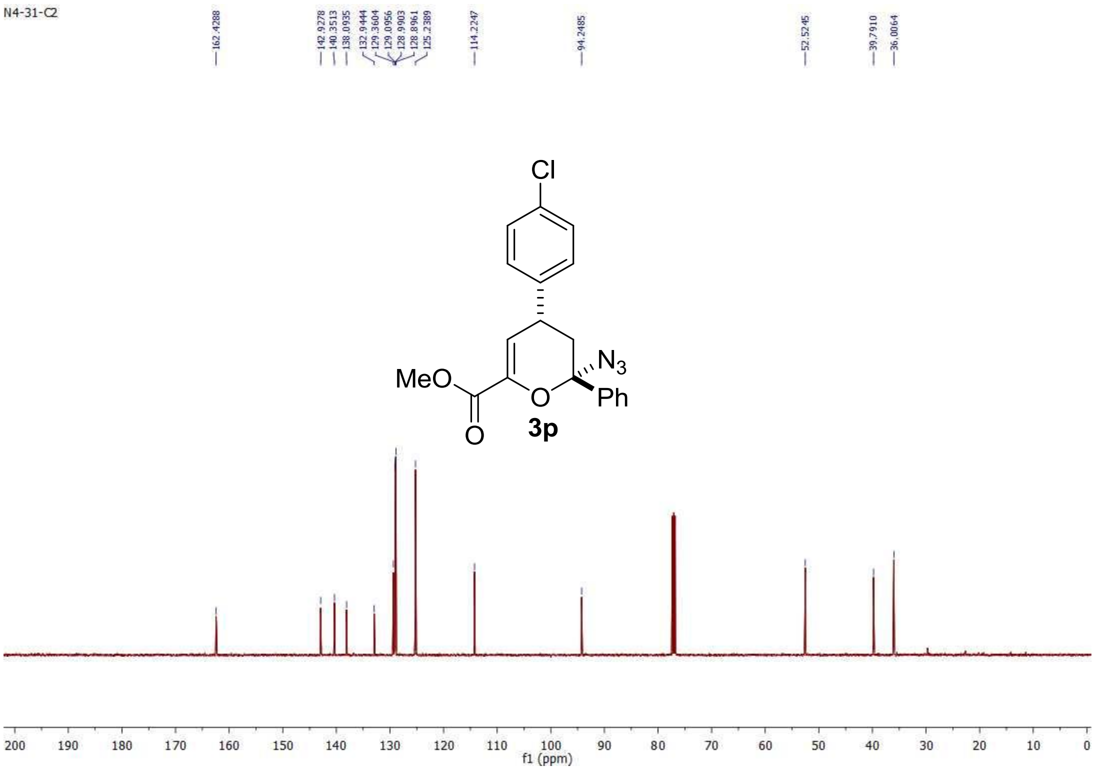

Supplementary Figure 78. HRMS spectra for 3p

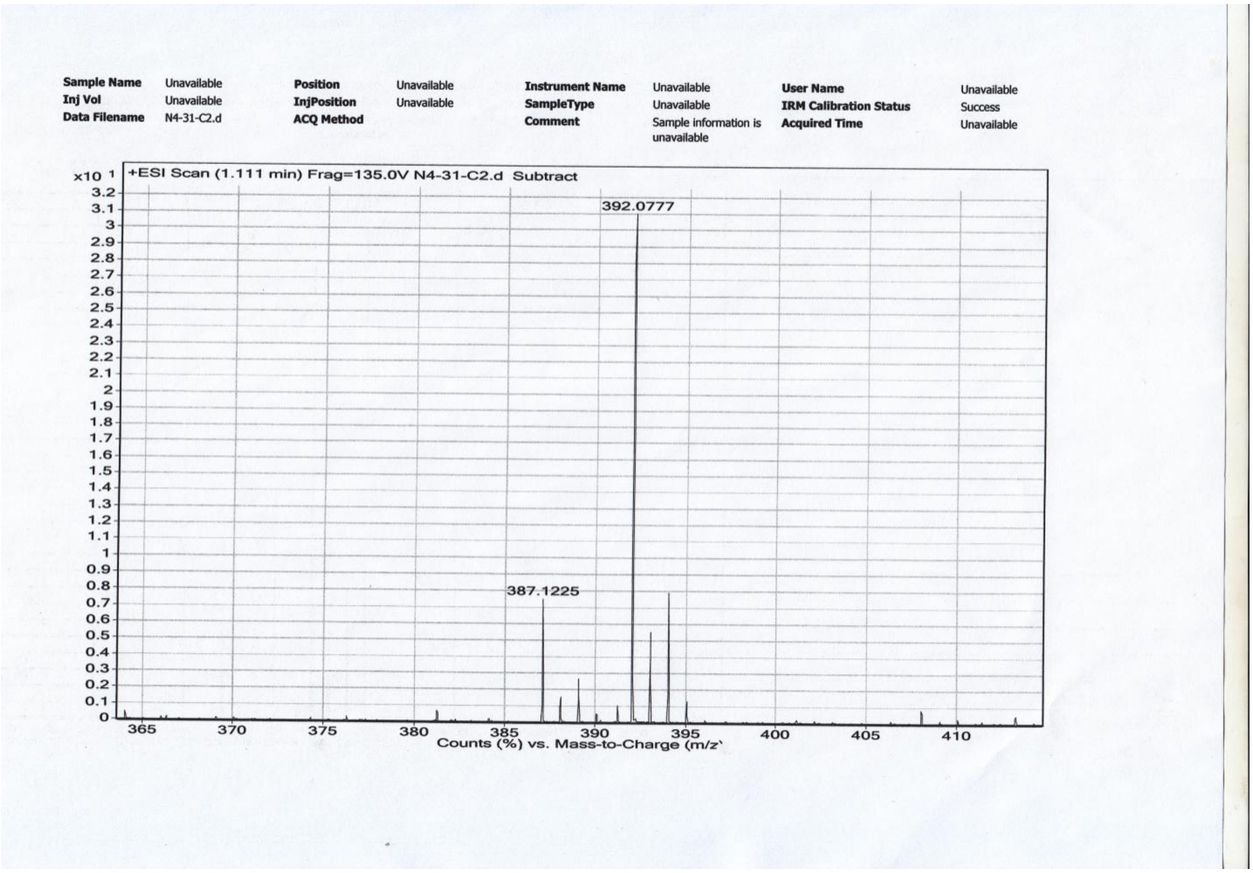

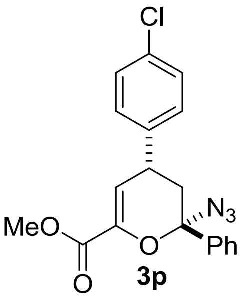

3p

Chemical Formula: $C_{19}H_{16}ClN_3O_3$
Exact Mass: 369.0880
Molecular Weight: 369.8016
m/z: 369.0880 (100.0%), 371.0851 (32.0%), 370.0914 (20.5%), 372.0884 (6.6%), 371.0947 (2.0%), 370.0851 (1.1%)

HRMS exact mass calcd for $C_{19}H_{16}ClN_3NaO_3$ $[M + Na]^+$ **392.0778**, found **392.0777**.

Supplementary Figure 79. ^{1}H and ^{13}C NMR spectra for 3q

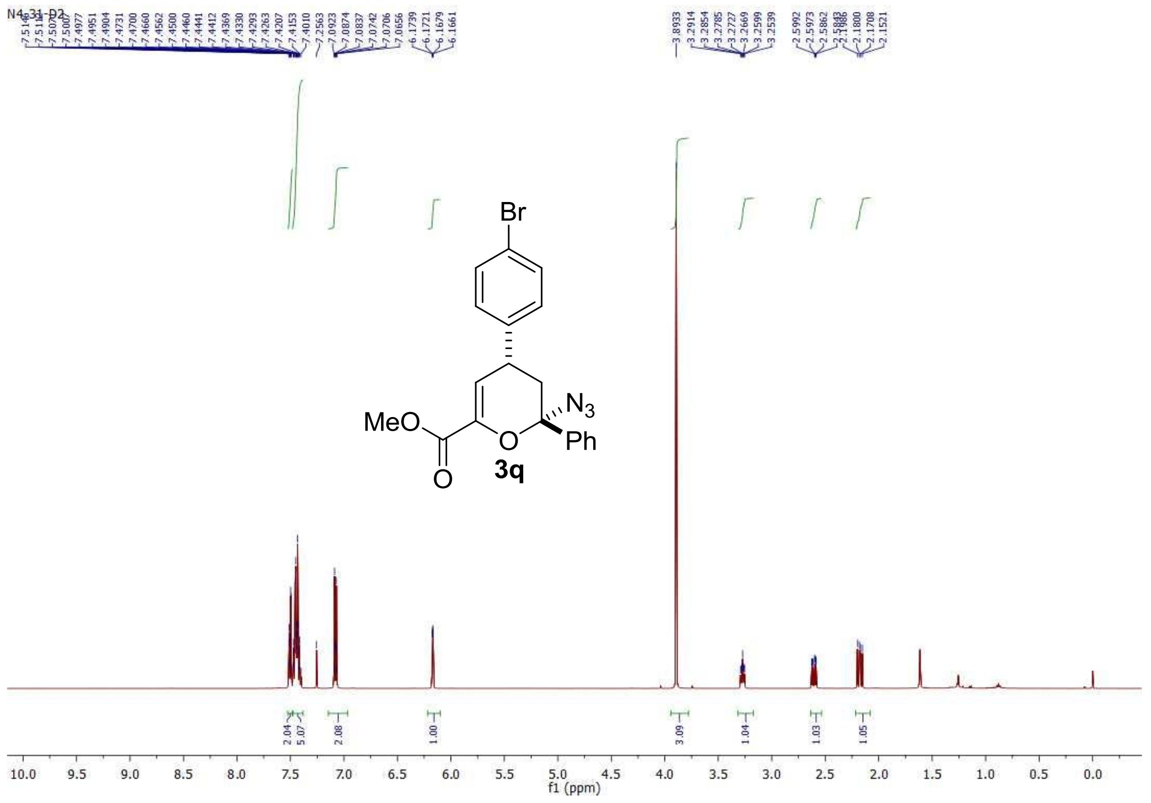

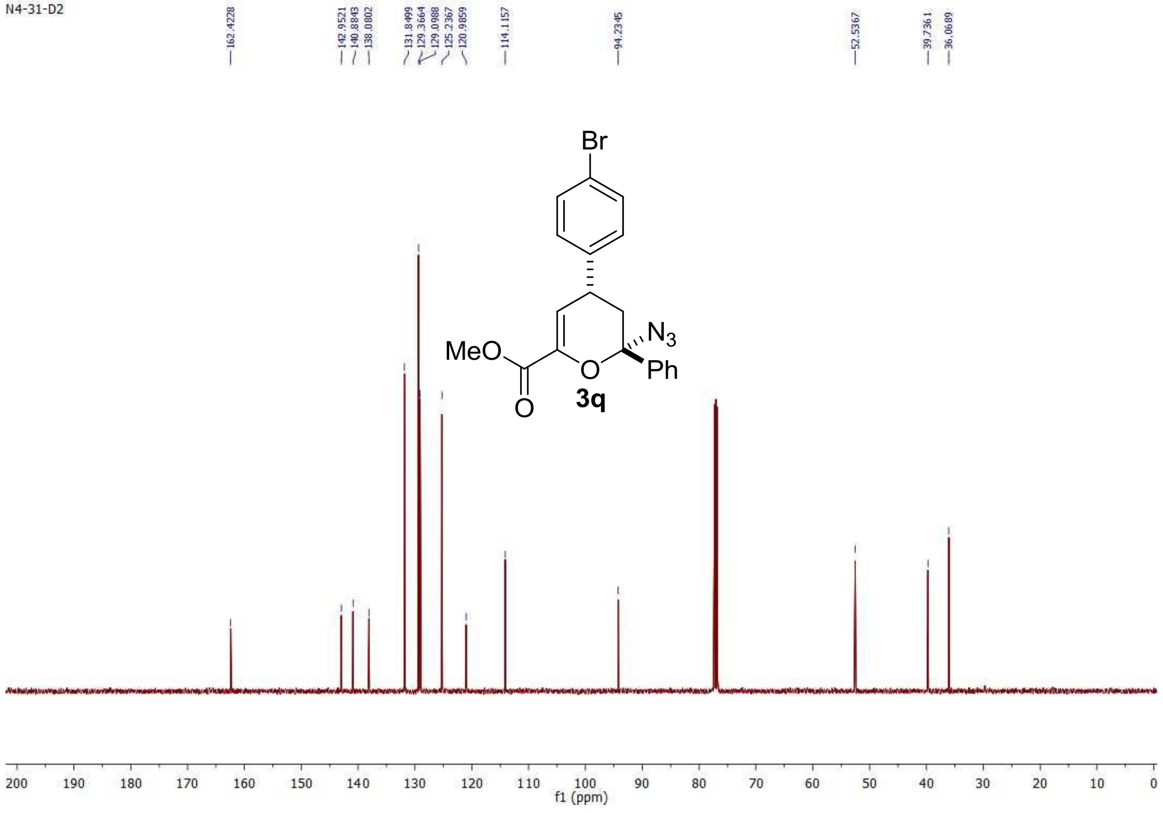

Supplementary Figure 80. HRMS spectra for 3q

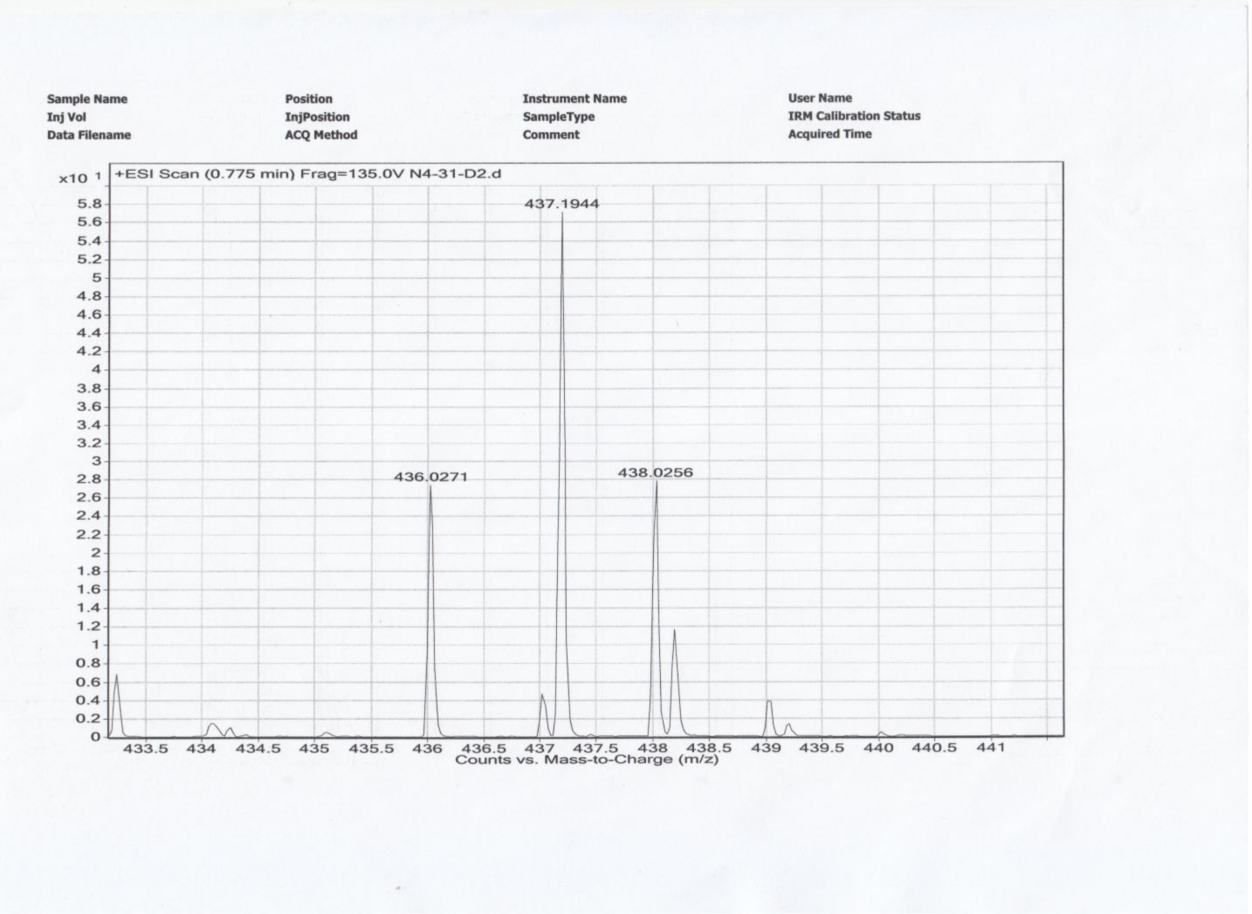

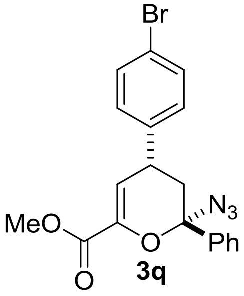

3q

Chemical Formula: $C_{19}H_{16}BrN_3O_3$
Exact Mass: 413.0375
Molecular Weight: 414.2526
m/z: 413.0375 (100.0%), 415.0355 (97.3%), 414.0409 (20.5%), 416.0388 (20.0%), 415.0442 (2.0%), 417.0422 (1.9%), 414.0345 (1.1%), 416.0325 (1.1%)

HRMS exact mass calcd for $C_{19}H_{16}BrN_3NaO_3$ $[M + Na]^+$ **436.0271**, found **436.0271**.

Supplementary Figure 81. ^{1}H and ^{13}C NMR spectra for 3r

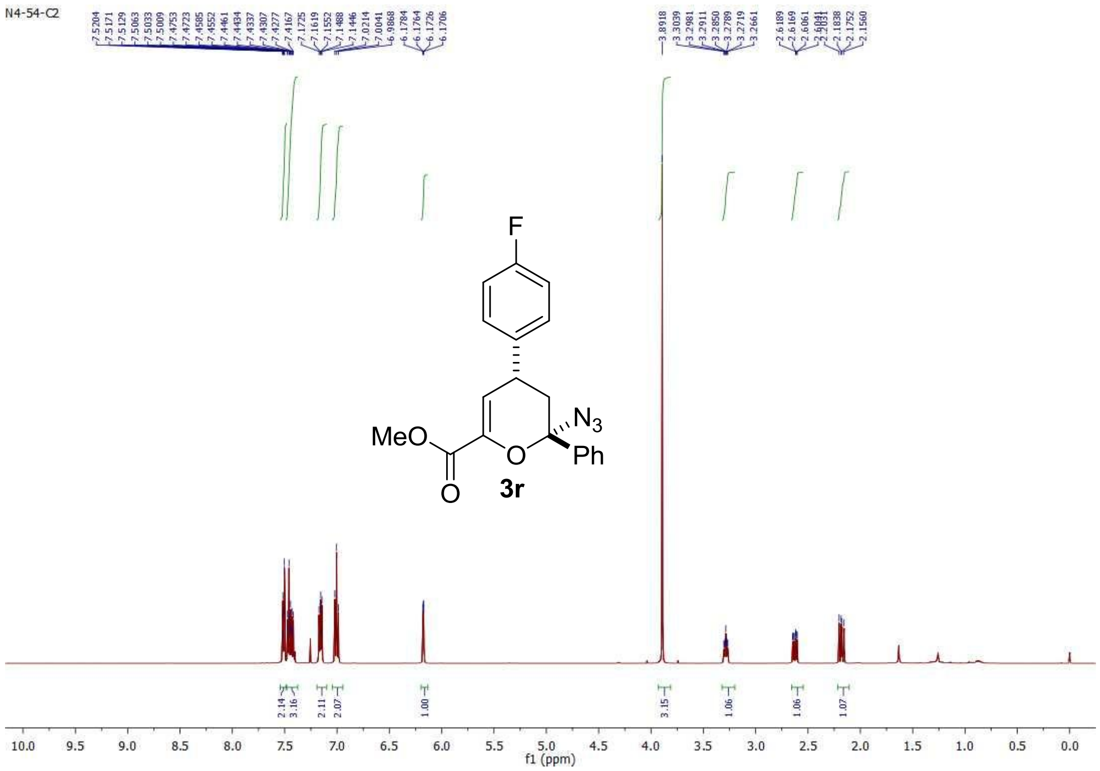

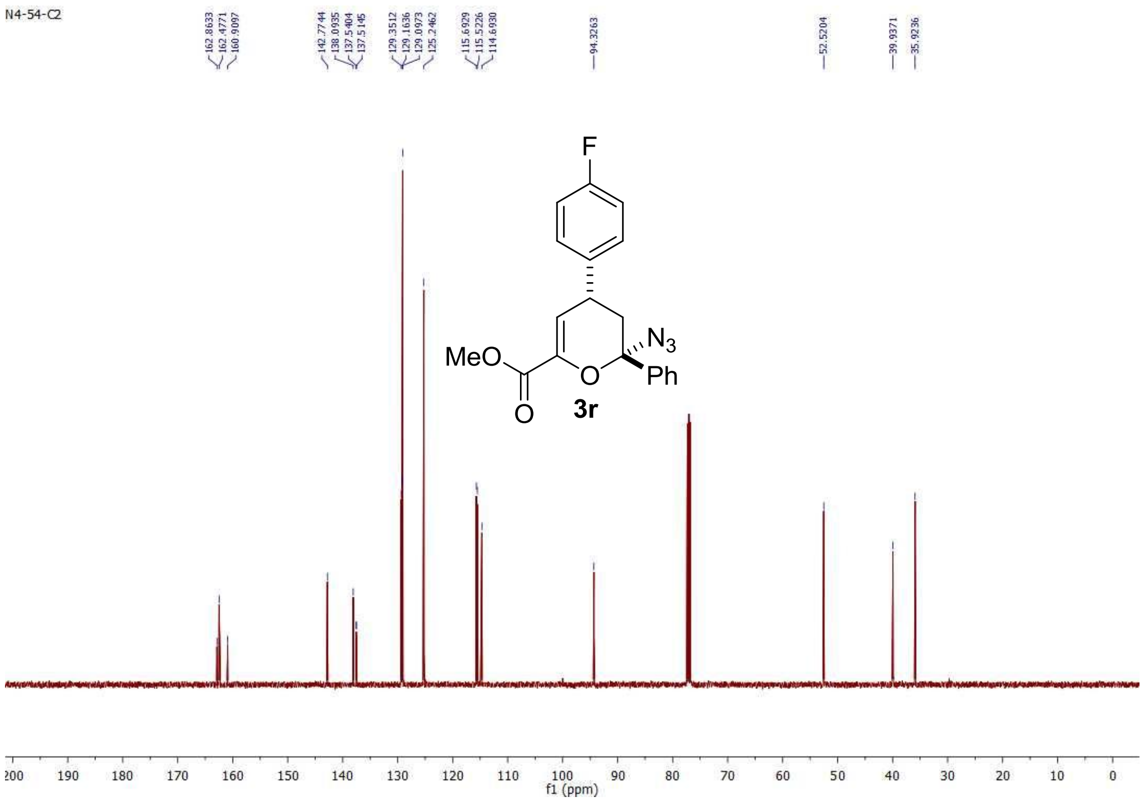

Supplementary Figure 82. HRMS spectra for 3r

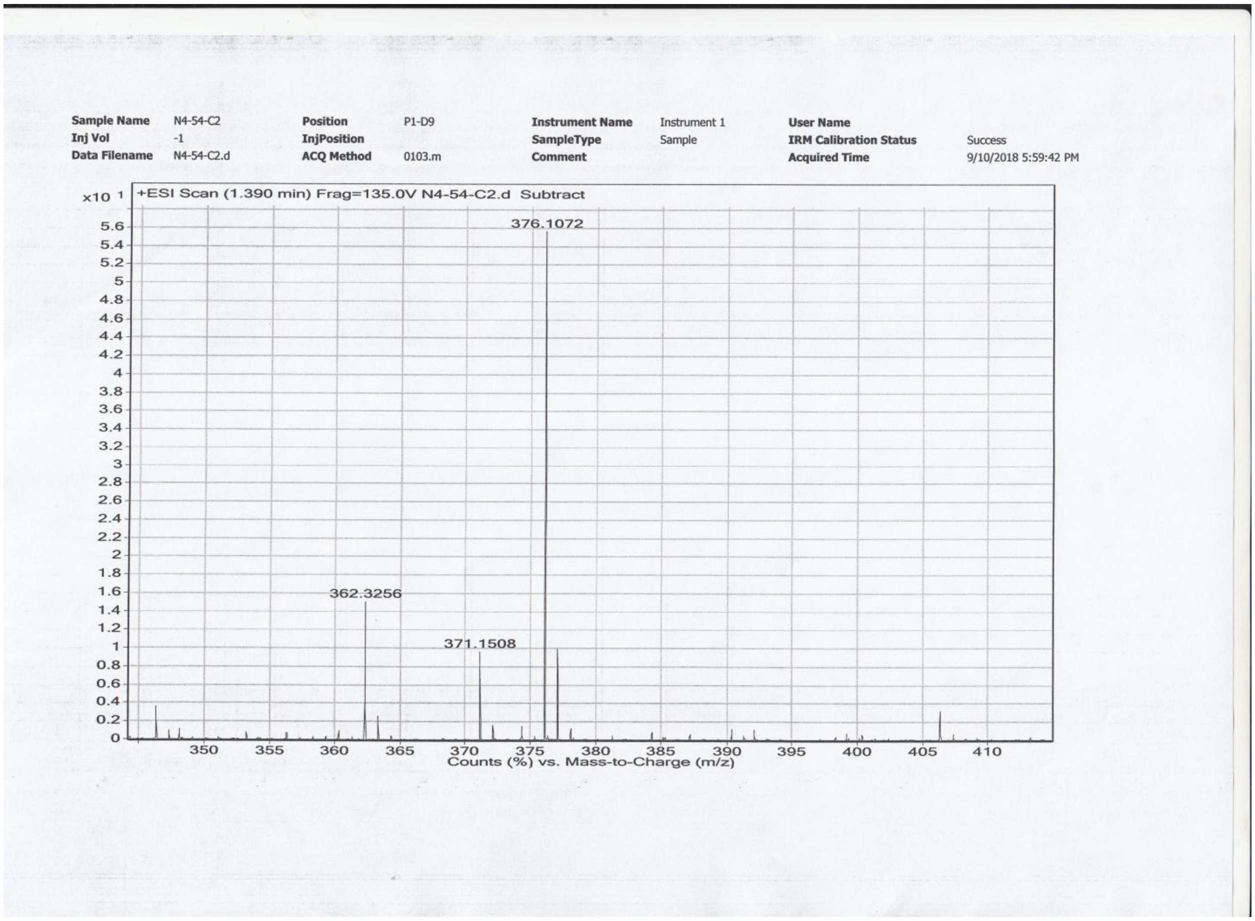

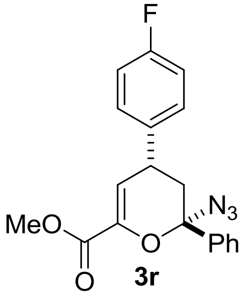

3r

Chemical Formula: $C_{19}H_{16}FN_3O_3$
Exact Mass: 353.1176
Molecular Weight: 353.3470
m/z: 353.1176 (100.0%), 354.1209 (20.5%), 355.1243 (2.0%), 354.1146 (1.1%)

HRMS exact mass calcd for $C_{19}H_{16}FrN_3NaO_3$ $[M + Na]^+$ **376.3368**, found **376.1072**.

Supplementary Figure 83. ^{1}H and ^{13}C NMR spectra for 3s

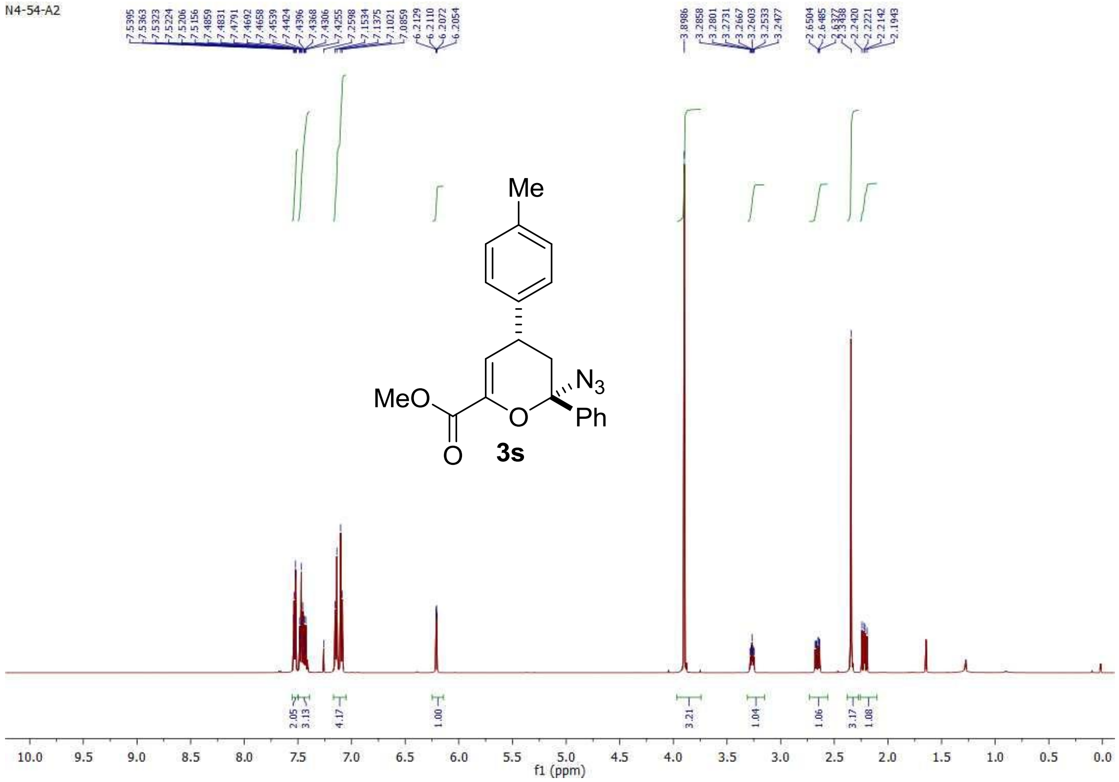

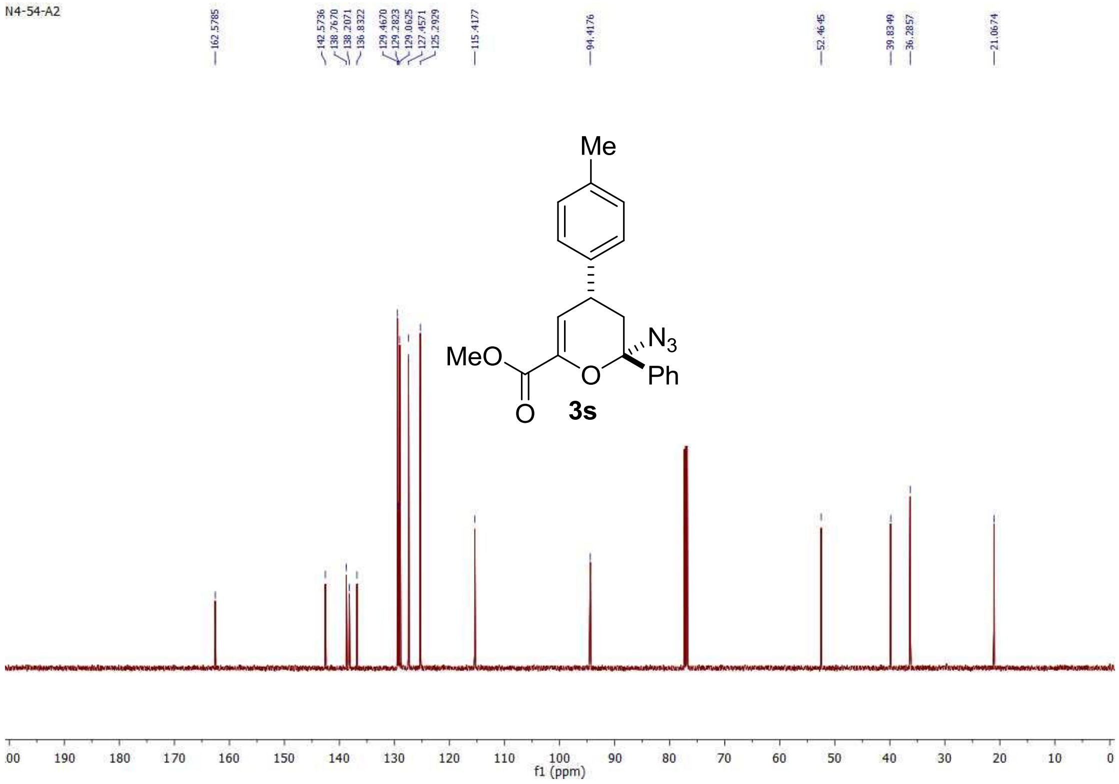

Supplementary Figure 84. HRMS spectra for 3s

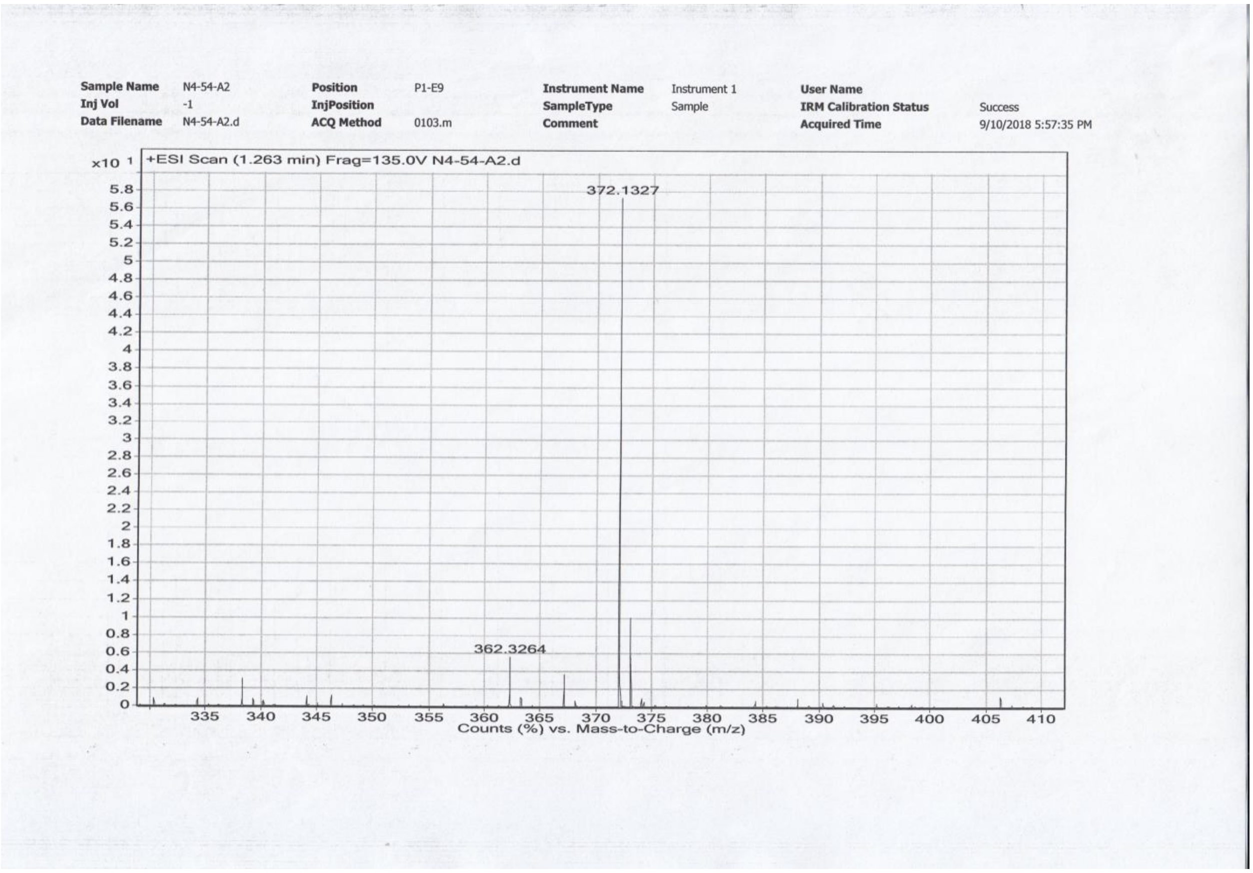

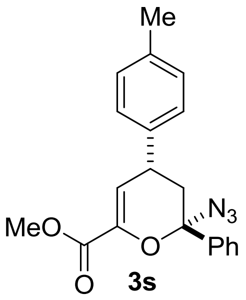

3s

Chemical Formula: $C_{20}H_{19}N_3O_3$
Exact Mass: 349.1426
Molecular Weight: 349.3832
m/z: 349.1426 (100.0%), 350.1460 (21.6%), 351.1494 (2.2%), 350.1397 (1.1%)

HRMS exact mass calcd for $C_{20}H_{19}N_3NaO_3$ $[M + Na]^+$ **372.1324**, found **372.1327**.

Supplementary Figure 85. ^{1}H and ^{13}C NMR spectra for 3t

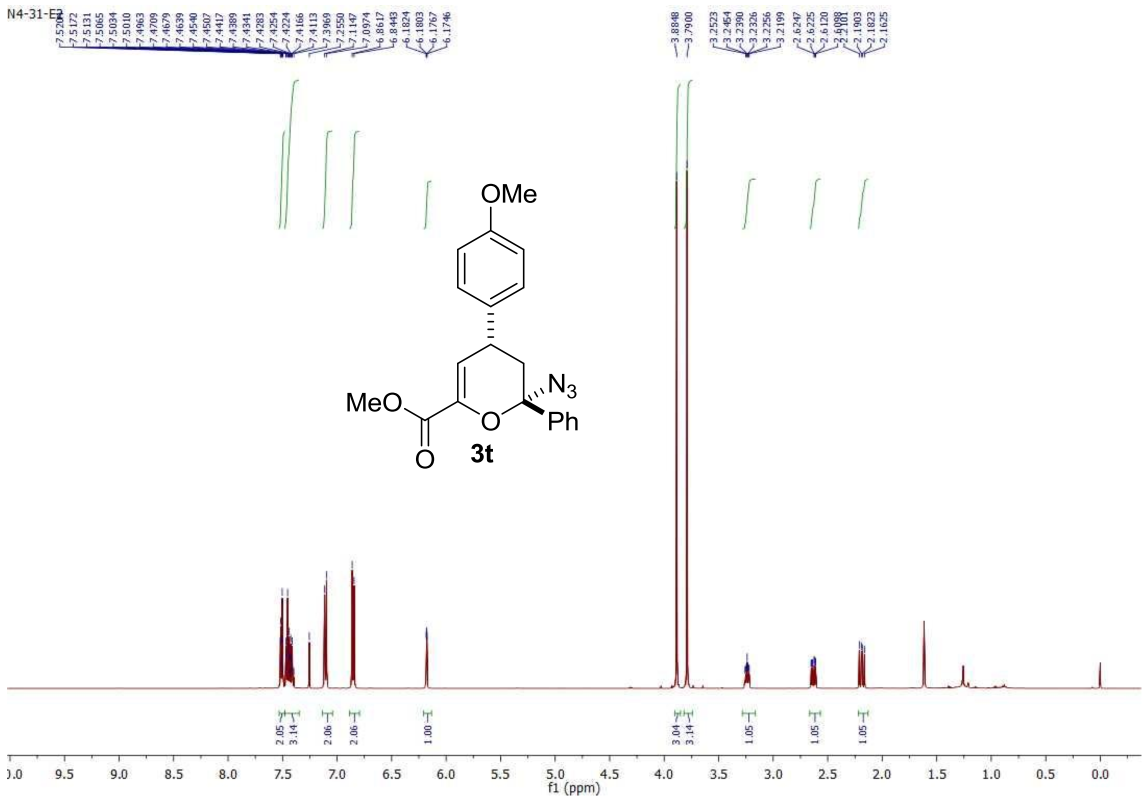

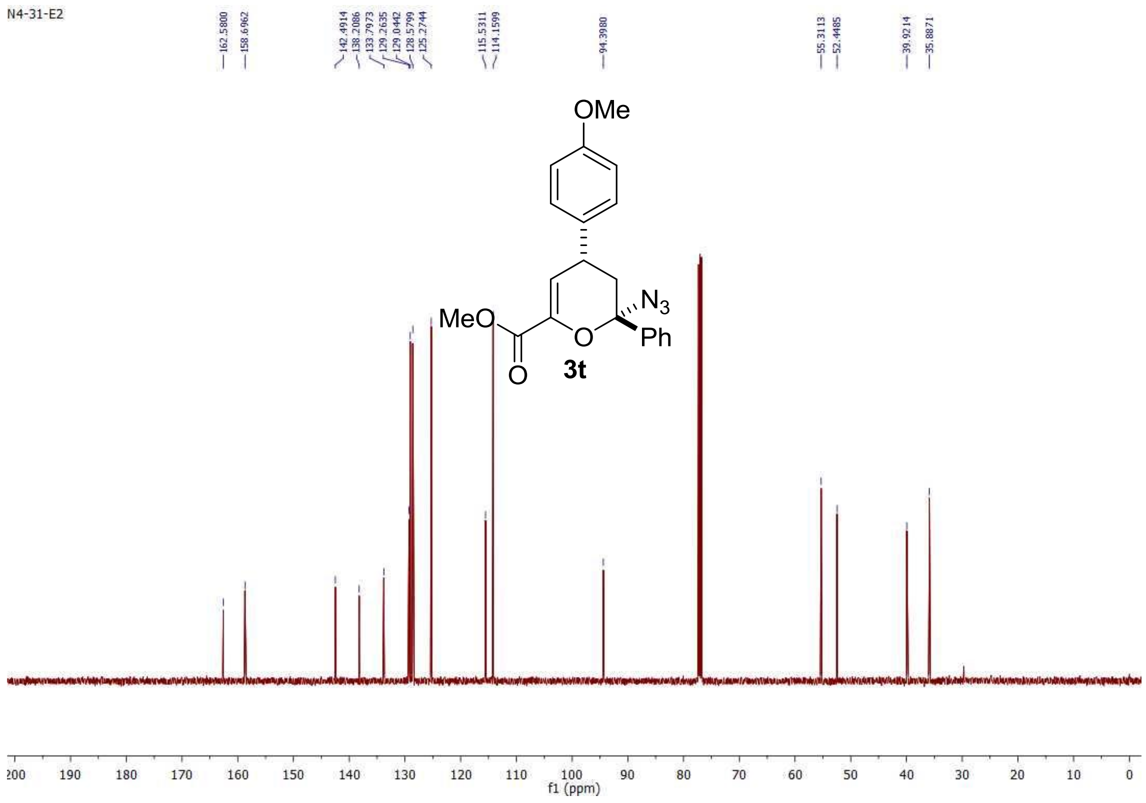

Supplementary Figure 86. HRMS spectra for 3t

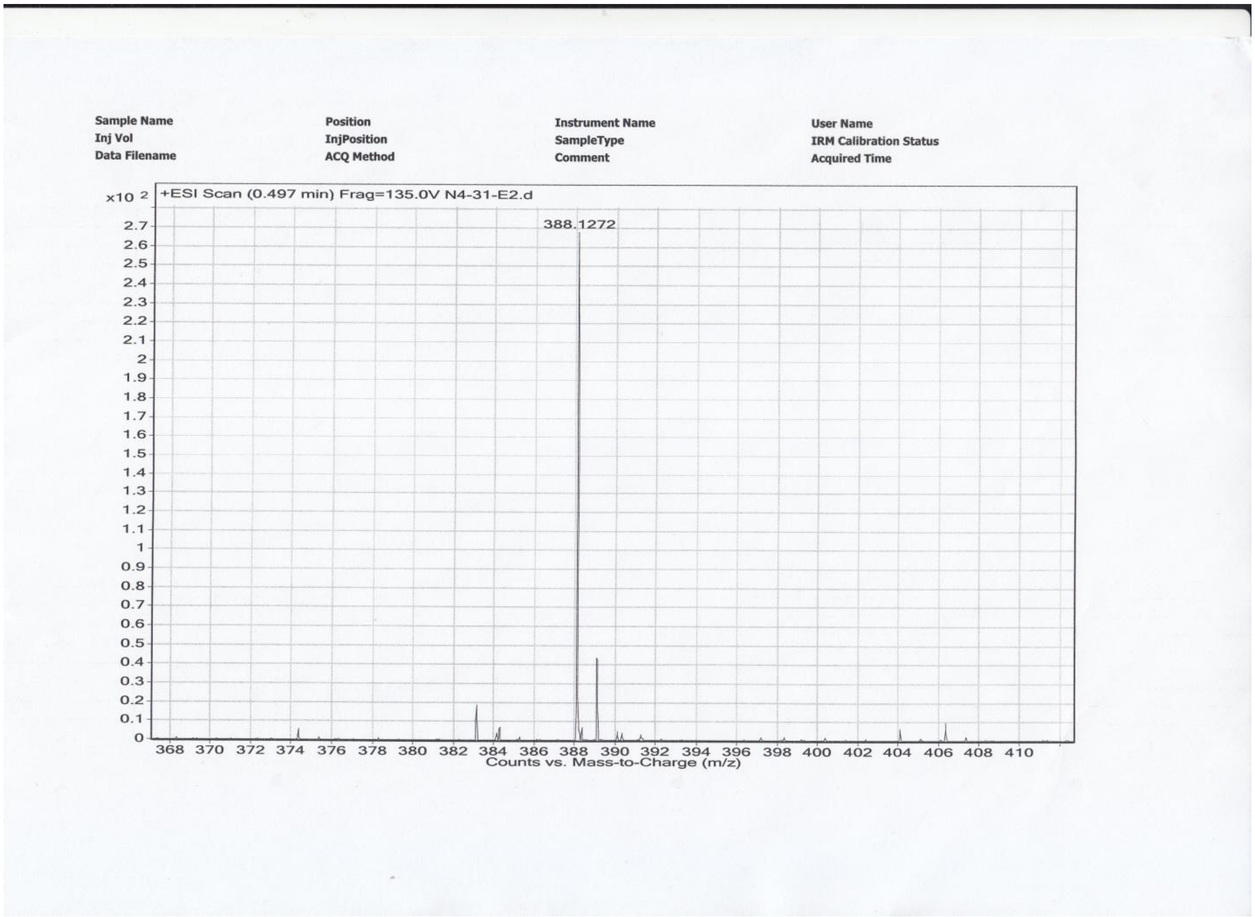

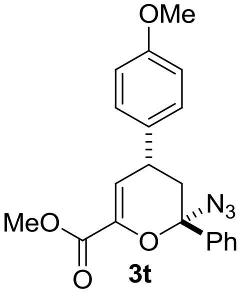

3t

Chemical Formula: $C_{20}H_{19}N_3O_4$
Exact Mass: 365.1376
Molecular Weight: 365.3826
m/z: 365.1376 (100.0%), 366.1409 (21.6%), 367.1443 (2.2%), 366.1346 (1.1%)

HRMS exact mass calcd for $C_{20}H_{19}N_3NaO_4$ $[M + Na]^+$ **388.1273**, found **388.1272**.

Supplementary Figure 87. ^{1}H and ^{13}C NMR spectra for 3u

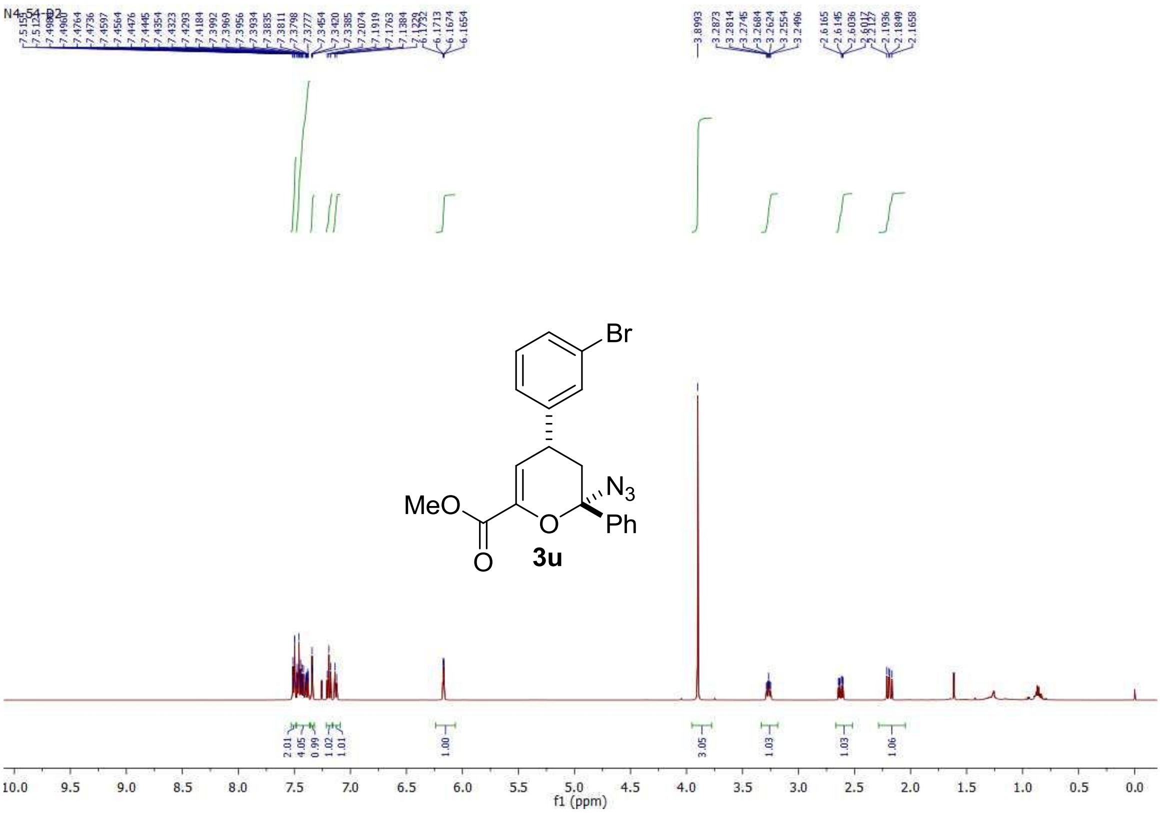

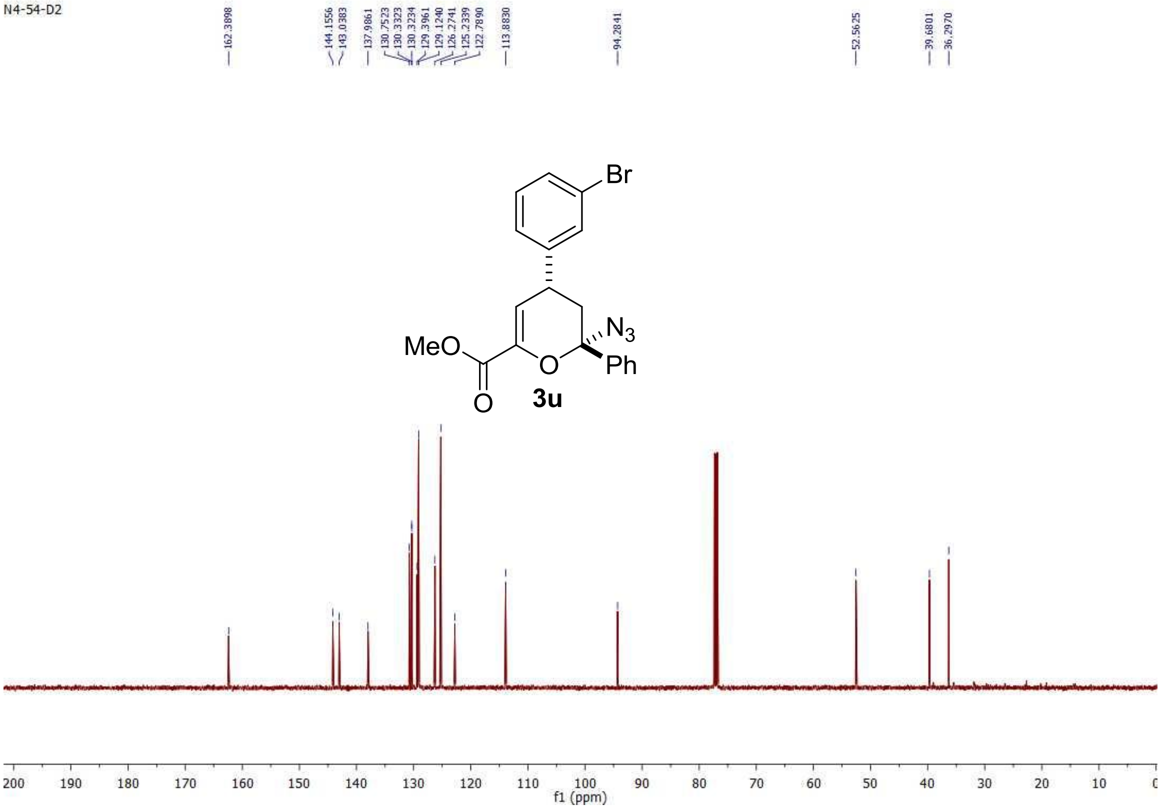

Supplementary Figure 88. HRMS spectra for 3u

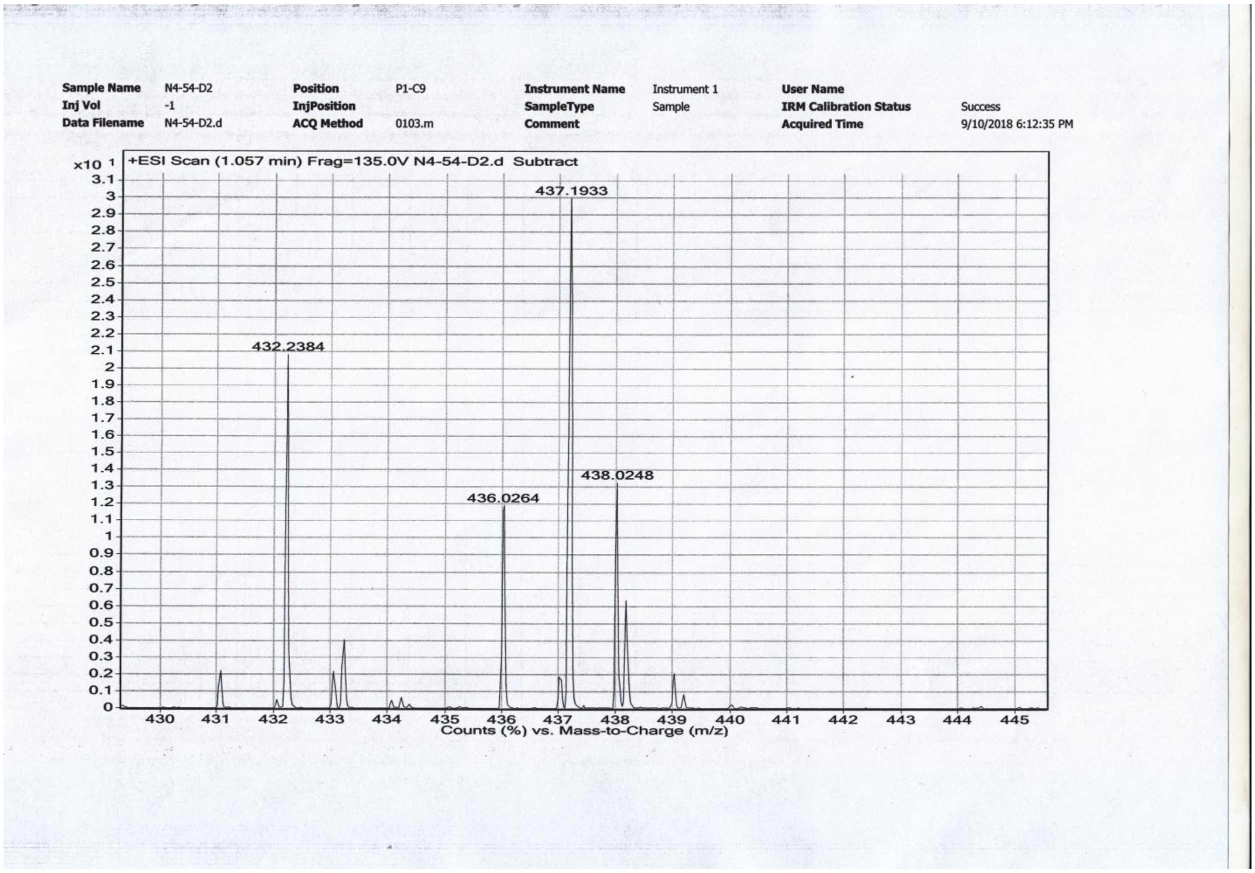

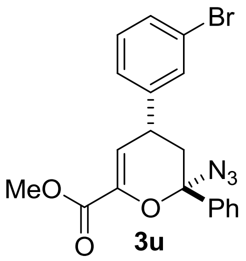

3u

Chemical Formula: $C_{19}H_{16}BrN_3O_3$

Exact Mass: 413.0375

Molecular Weight: 414.2526

m/z: 413.0375 (100.0%), 415.0355 (97.3%), 414.0409 (20.5%), 416.0388 (20.0%), 415.0442 (2.0%), 417.0422 (1.9%), 414.0345 (1.1%), 416.0325 (1.1%)

HRMS exact mass calcd for $C_{19}H_{16}BrN_3NaO_3$ $[M + Na]^+$ **436.0273**, found **436.0264**.

Supplementary Figure 89. ^{1}H and ^{13}C NMR spectra for 3v

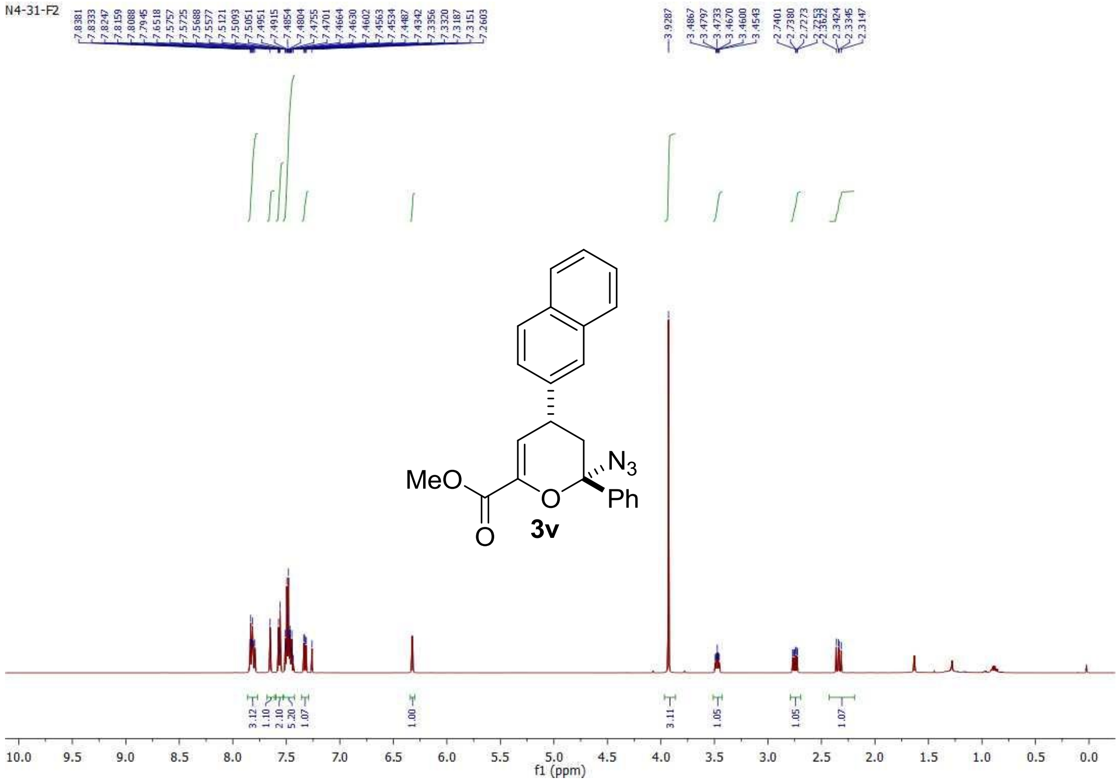

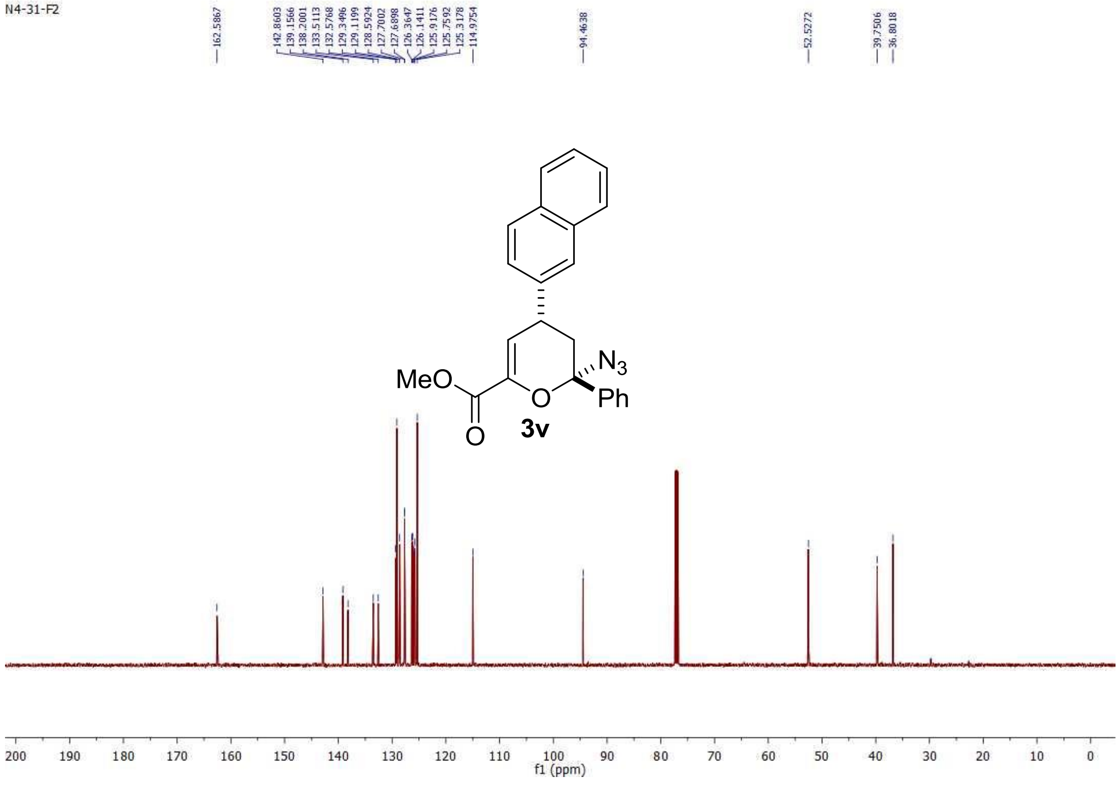

Supplementary Figure 90. HRMS spectra for 3v

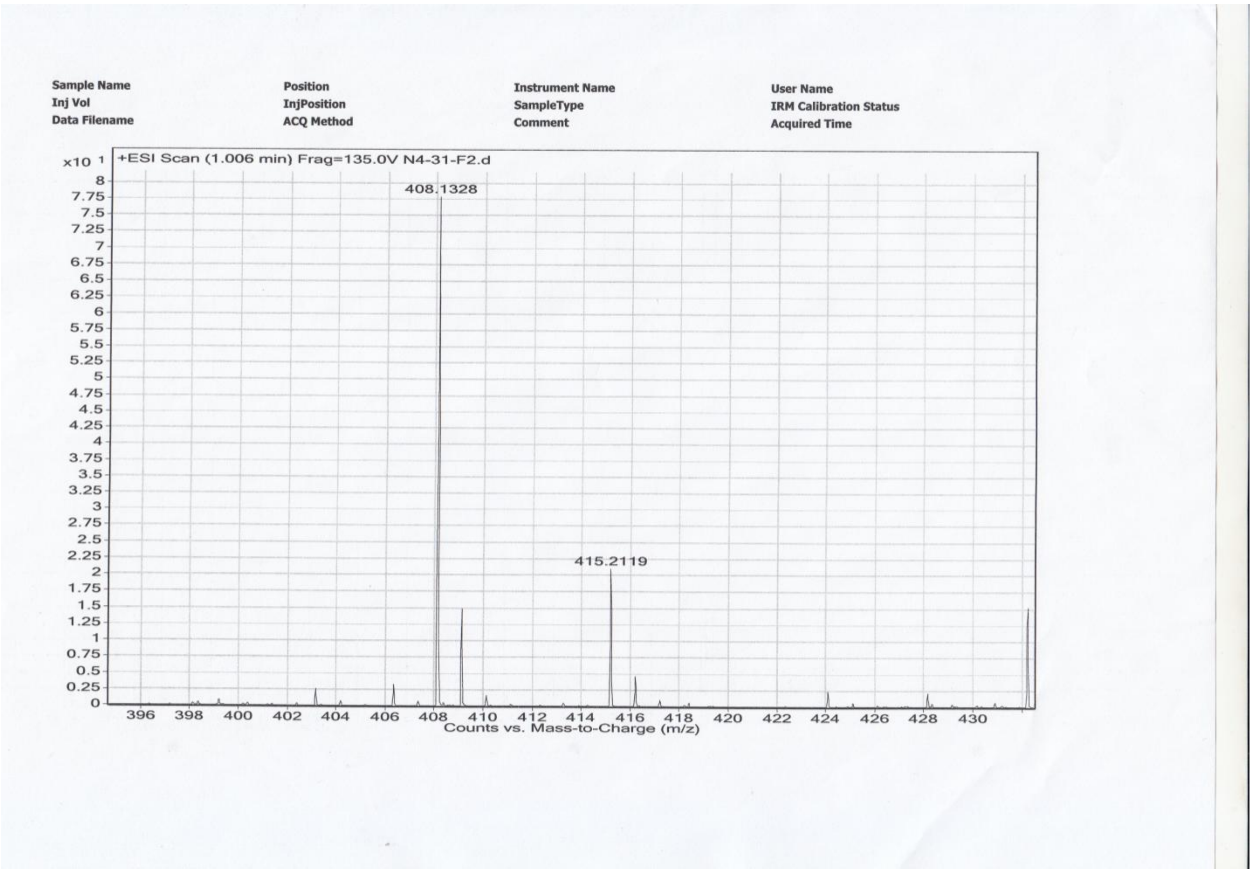

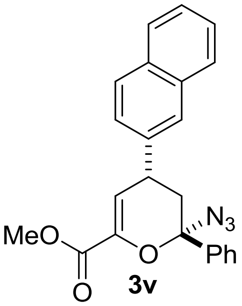

Chemical Formula: $C_{23}H_{19}N_3O_3$
Exact Mass: 385.1426
Molecular Weight: 385.4153
m/z: 385.1426 (100.0%), 386.1460 (24.9%), 387.1494 (3.0%), 386.1397 (1.1%)

HRMS exact mass calcd for $C_{23}H_{19}N_3NaO_3$ $[M + Na]^+$ **408.1324**, found **408.1328**.

Supplementary Figure 91. ^{1}H and ^{13}C NMR spectra for 3w

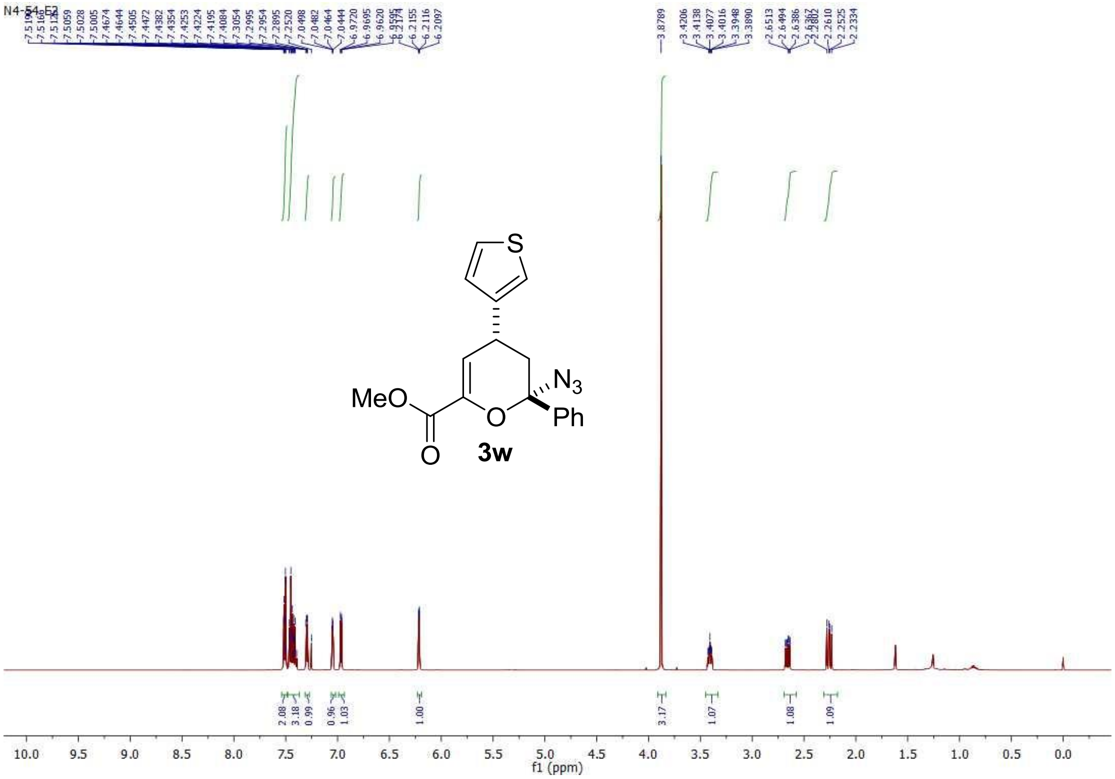

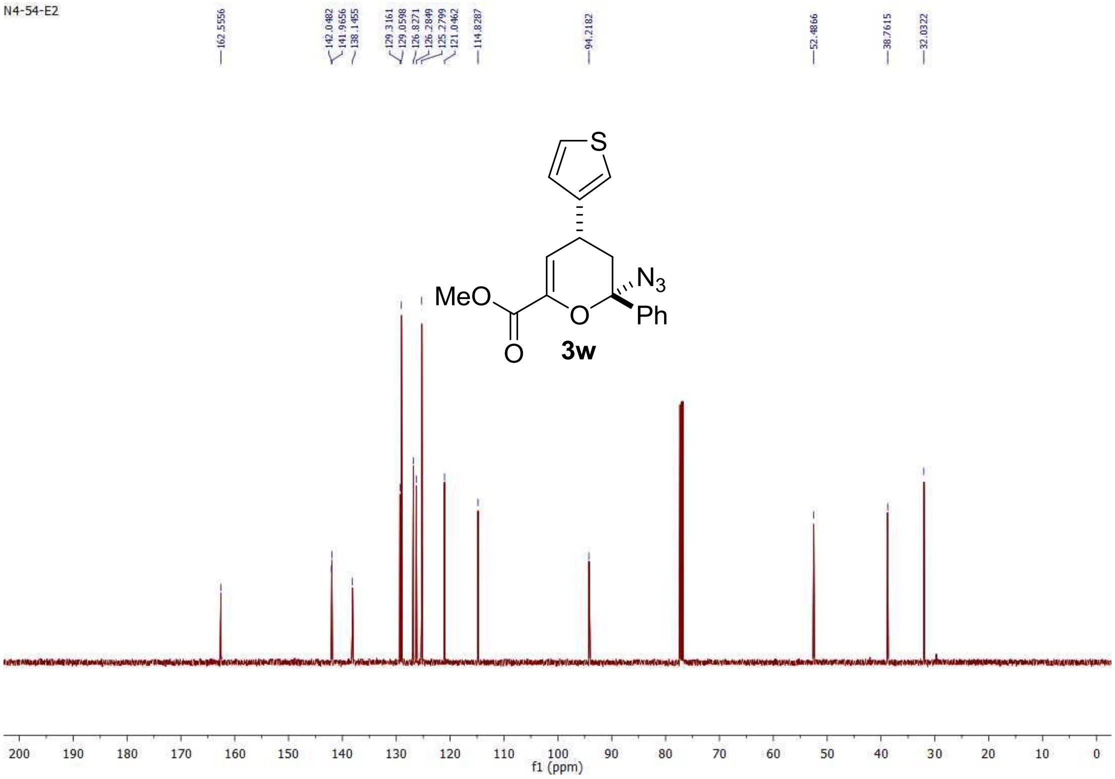

Supplementary Figure 92. HRMS spectra for 3w

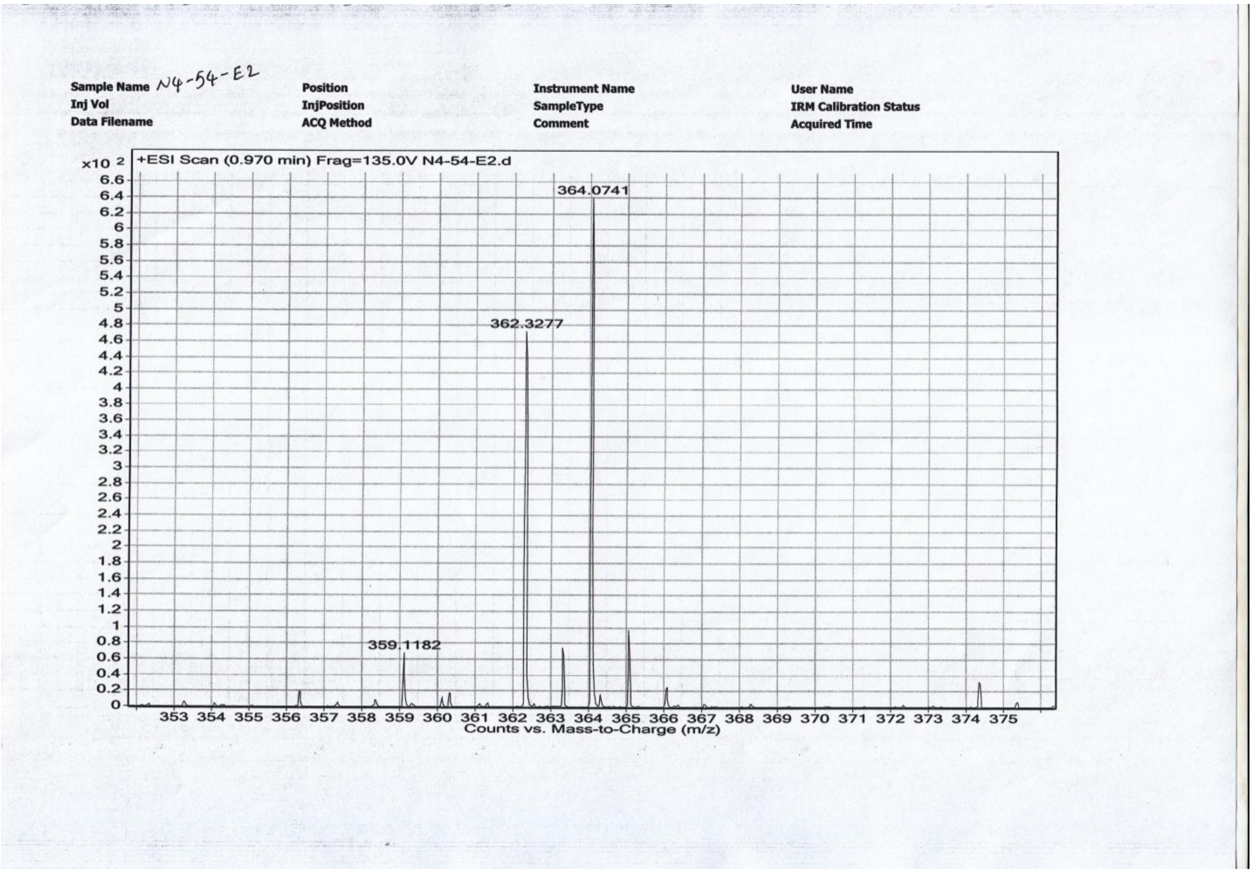

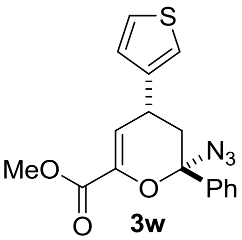

3w

Chemical Formula: $C_{17}H_{15}N_3O_3S$
Exact Mass: 341.0834
Molecular Weight: 341.3843
m/z: 341.0834 (100.0%), 342.0868 (18.4%), 343.0792 (4.5%), 343.0901 (1.6%), 342.0804 (1.1%)

HRMS exact mass calcd for $C_{17}H_{15}N_3NaO_3S$ $[M + Na]^+$ **364.0732**, found **364.0741**.

Supplementary Figure 93. ^{1}H and ^{13}C NMR spectra for 4a

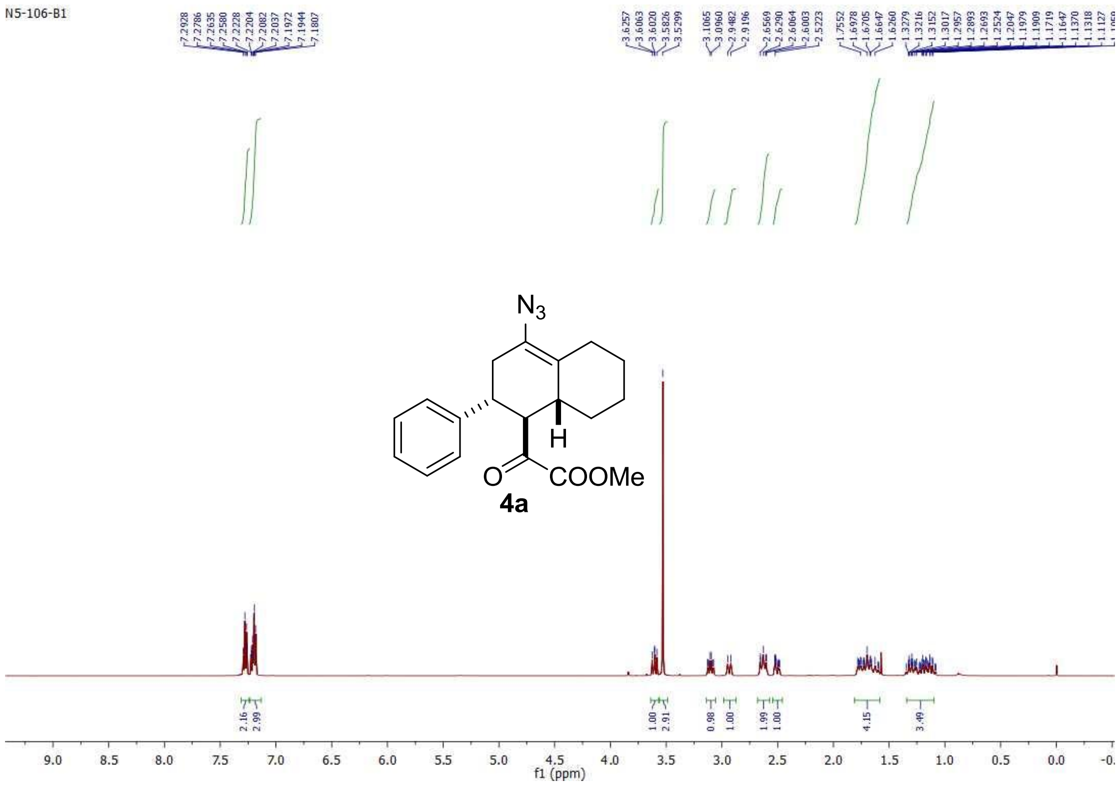

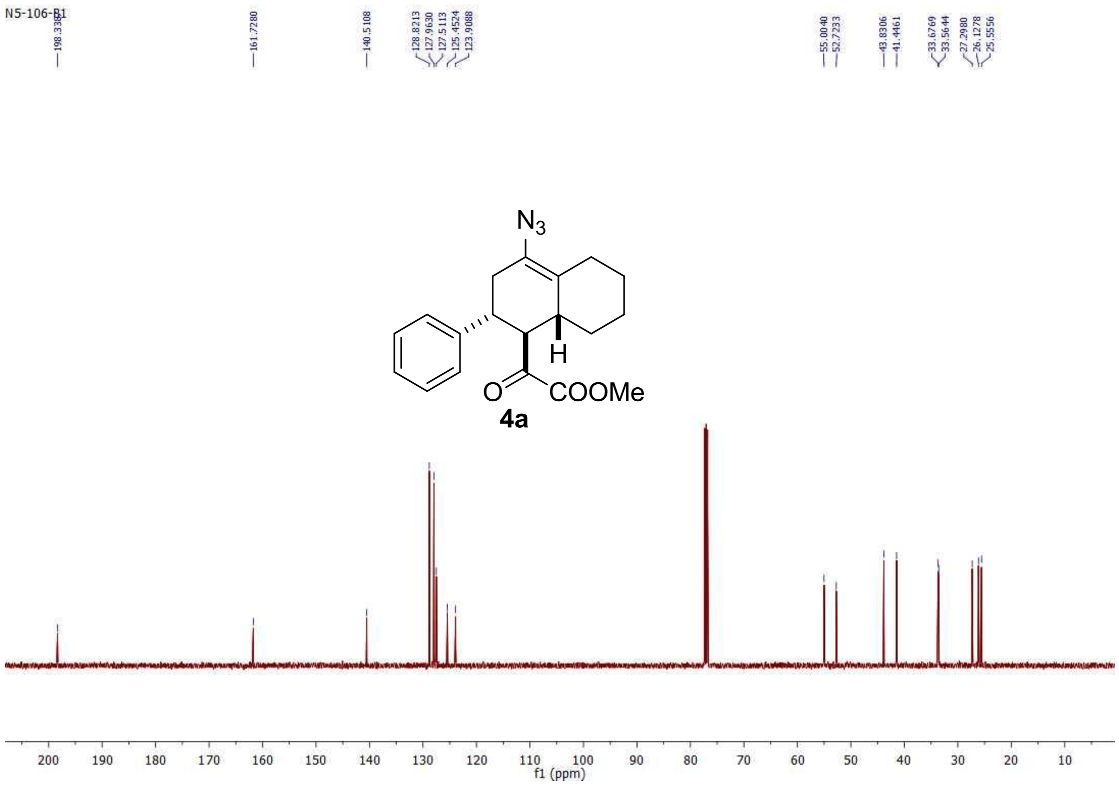

Supplementary Figure 94. HRMS spectra for 4a

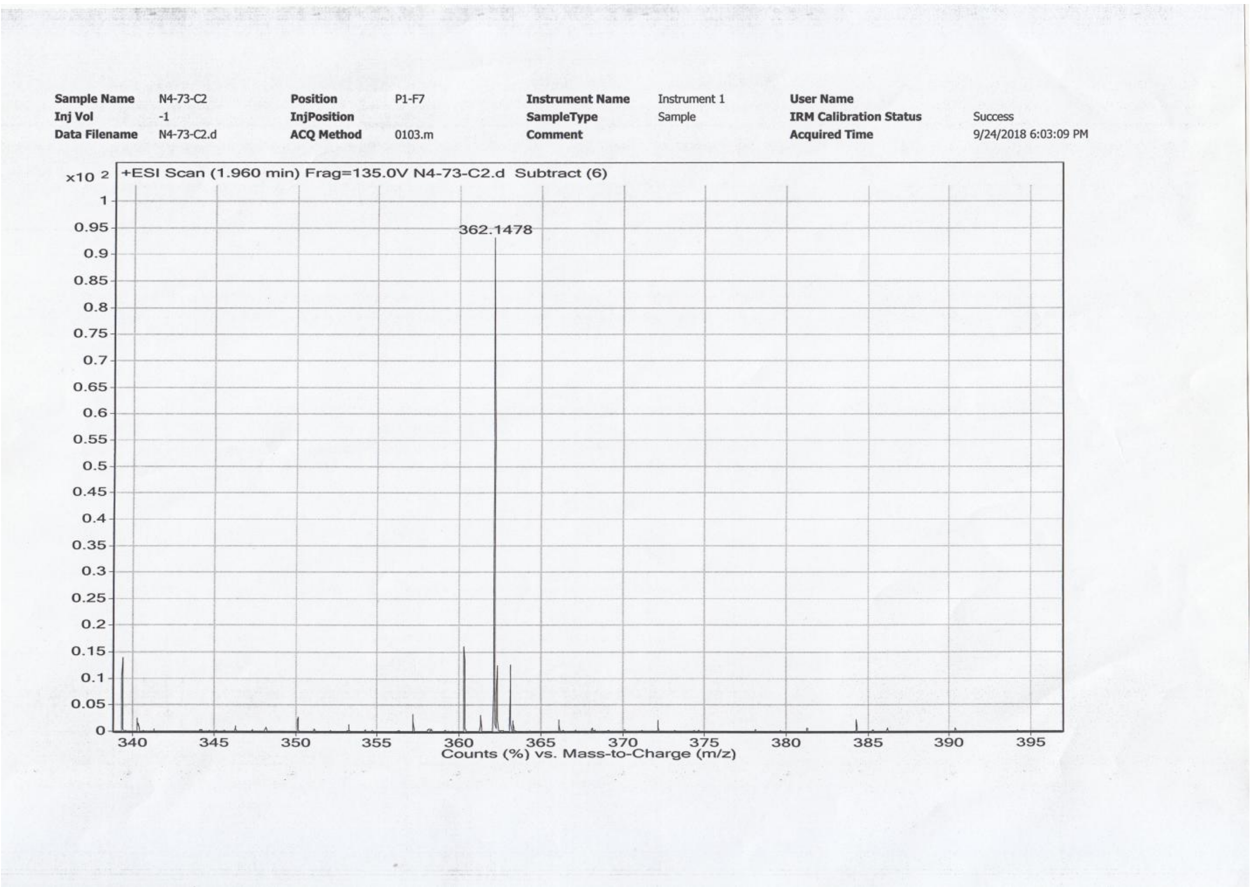

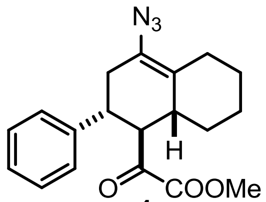

4a

Chemical Formula: $C_{19}H_{21}N_3O_3$
Exact Mass: 339.1583
Molecular Weight: 339.3883
m/z: 339.1583 (100.0%), 340.1616 (20.5%), 341.1650 (2.0%), 340.1553 (1.1%)

HRMS exact mass calcd for $C_{19}H_{21}N_3NaO_3$ $[M + Na]^+$ **362.1481**, found **362.1478**.

Supplementary Figure 95. ^{1}H and ^{13}C NMR spectra for 4b

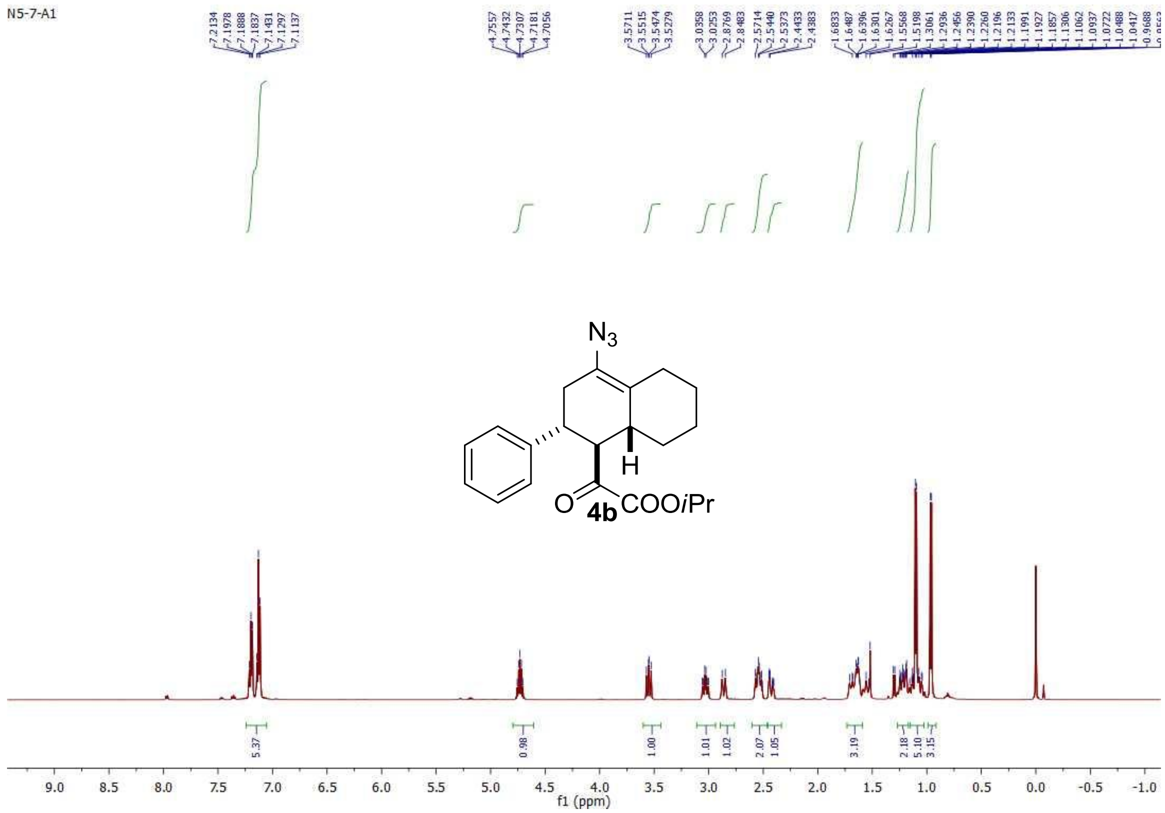

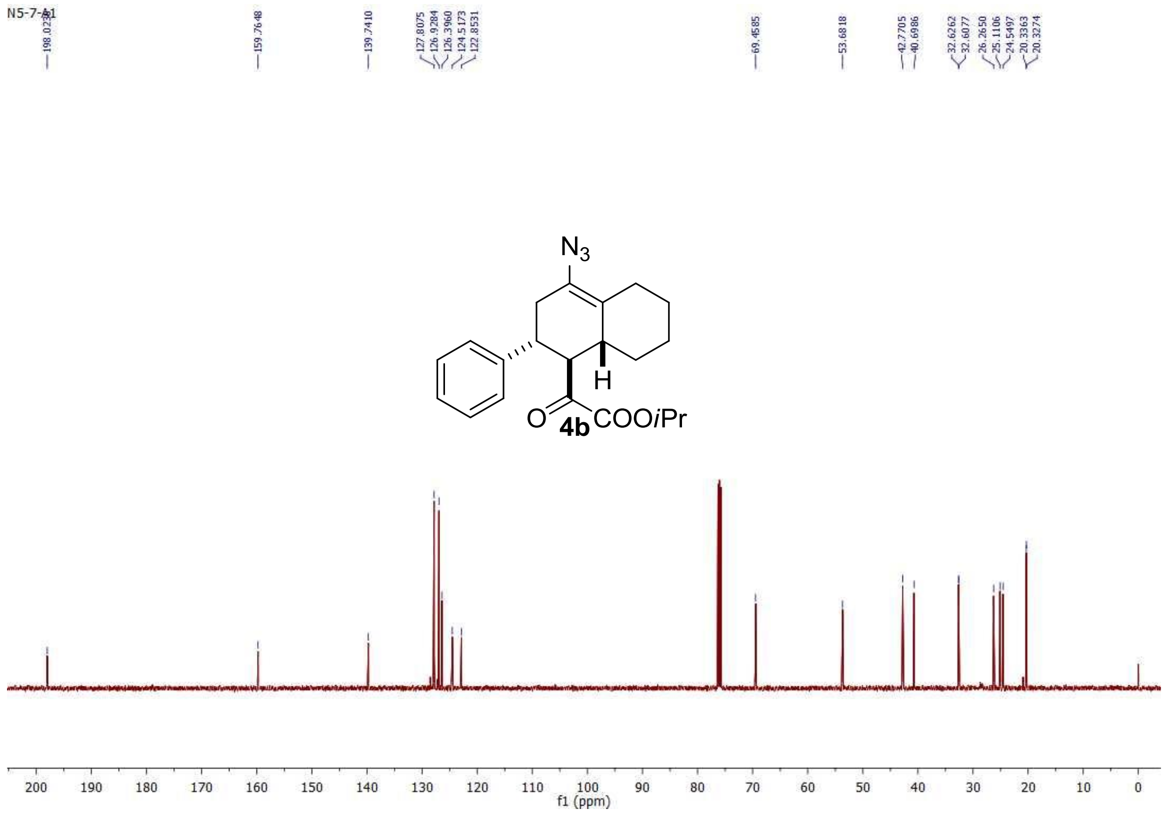

Supplementary Figure 96. HRMS spectra for 4b

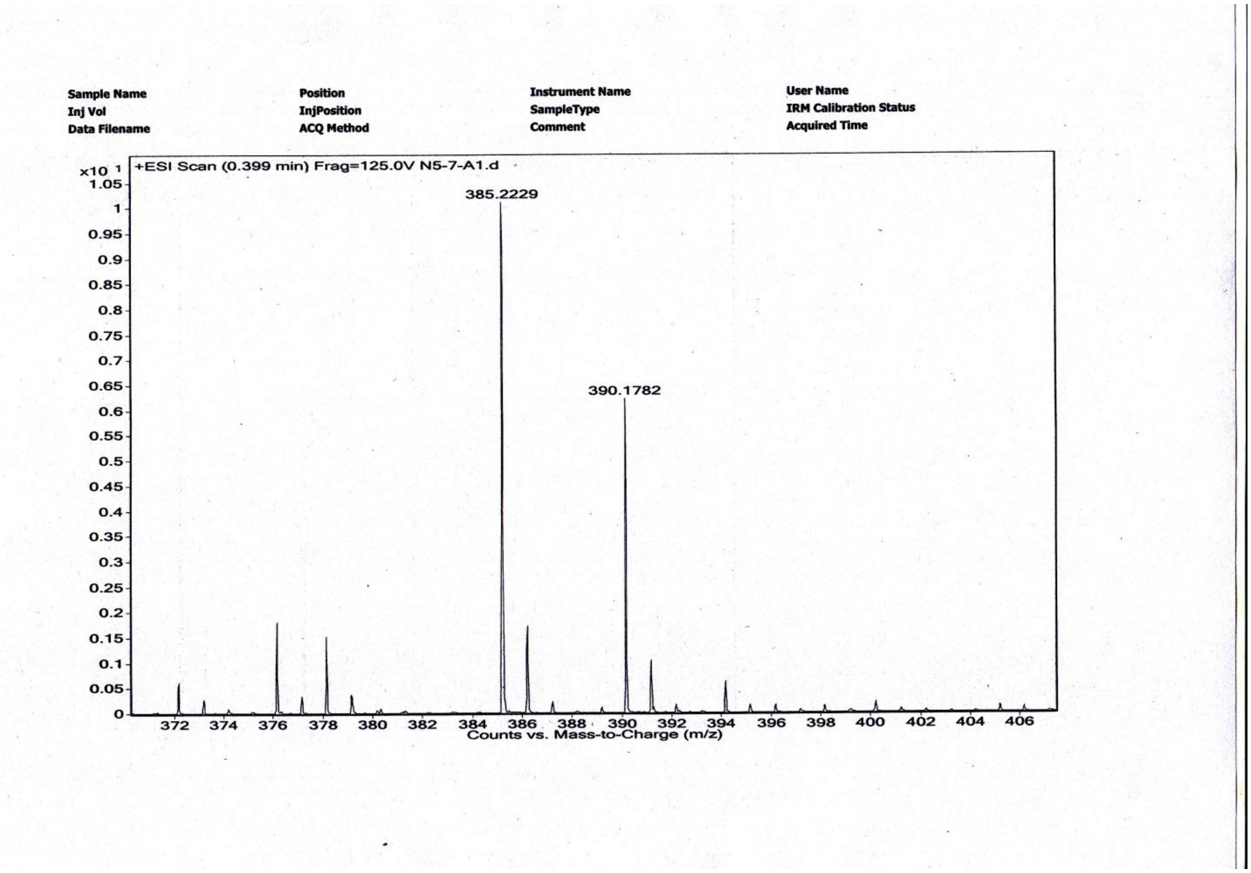

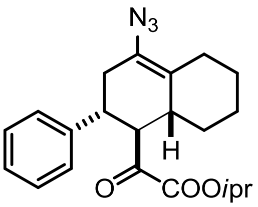

4b

Chemical Formula: $C_{17}H_{18}N_3O$

Exact Mass: 280.1450

Molecular Weight: 280.3443

m/z: 280.1450 (100.0%), 281.1483 (18.4%), 282.1517 (1.6%), 281.1420 (1.1%)

HRMS exact mass calcd for $C_{21}H_{25}N_3NaO_3$ $[M + Na]^+$ **390.1794**, found **390.1782**.

Supplementary Figure 97. ^{1}H and ^{13}C NMR spectra for 4c

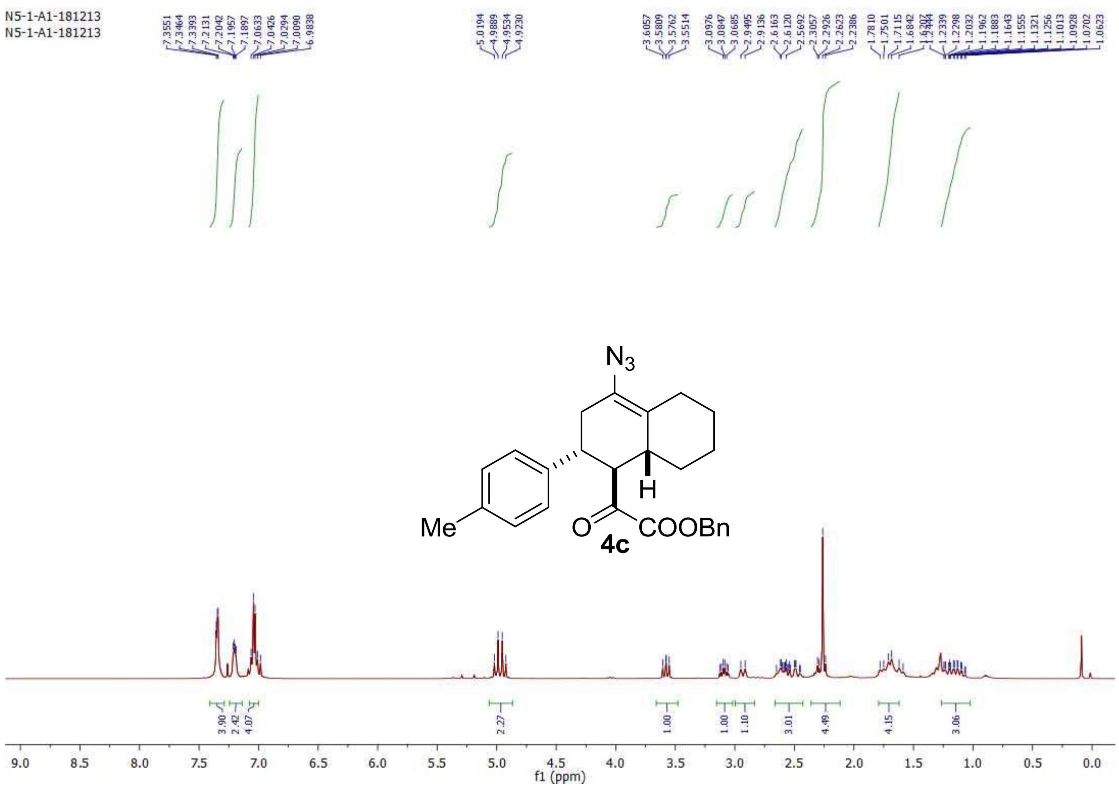

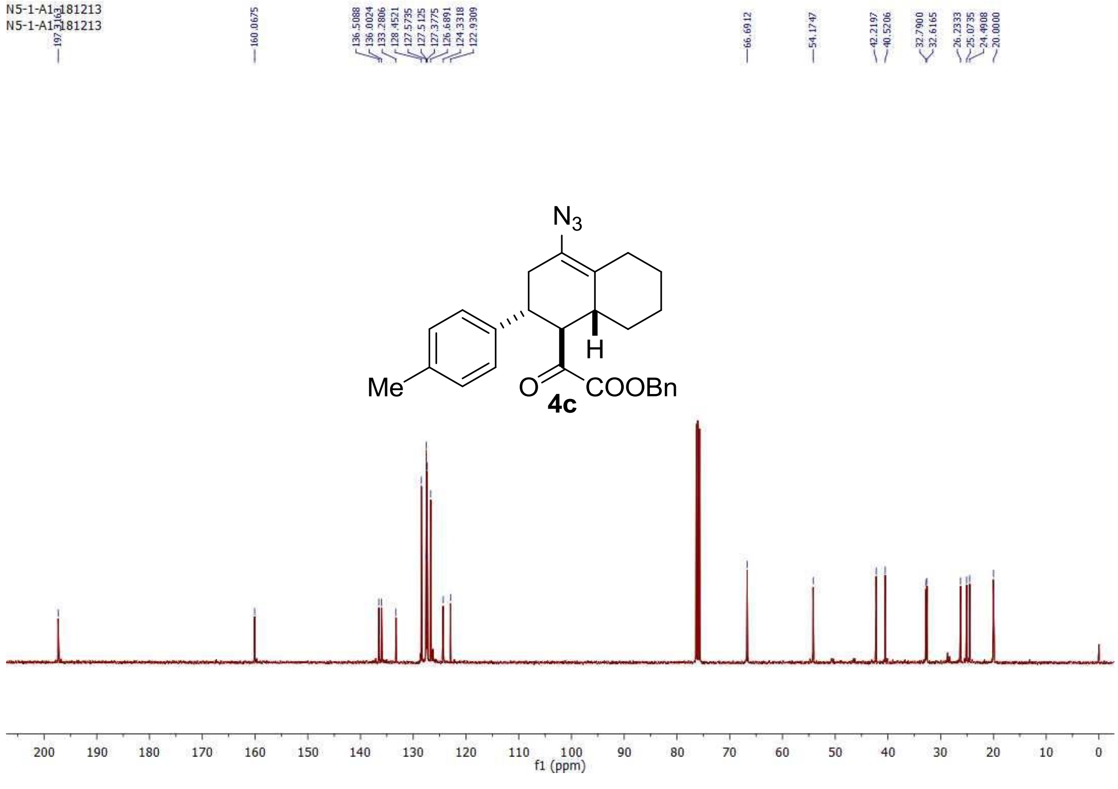

Supplementary Figure 98. HRMS spectra for 4c

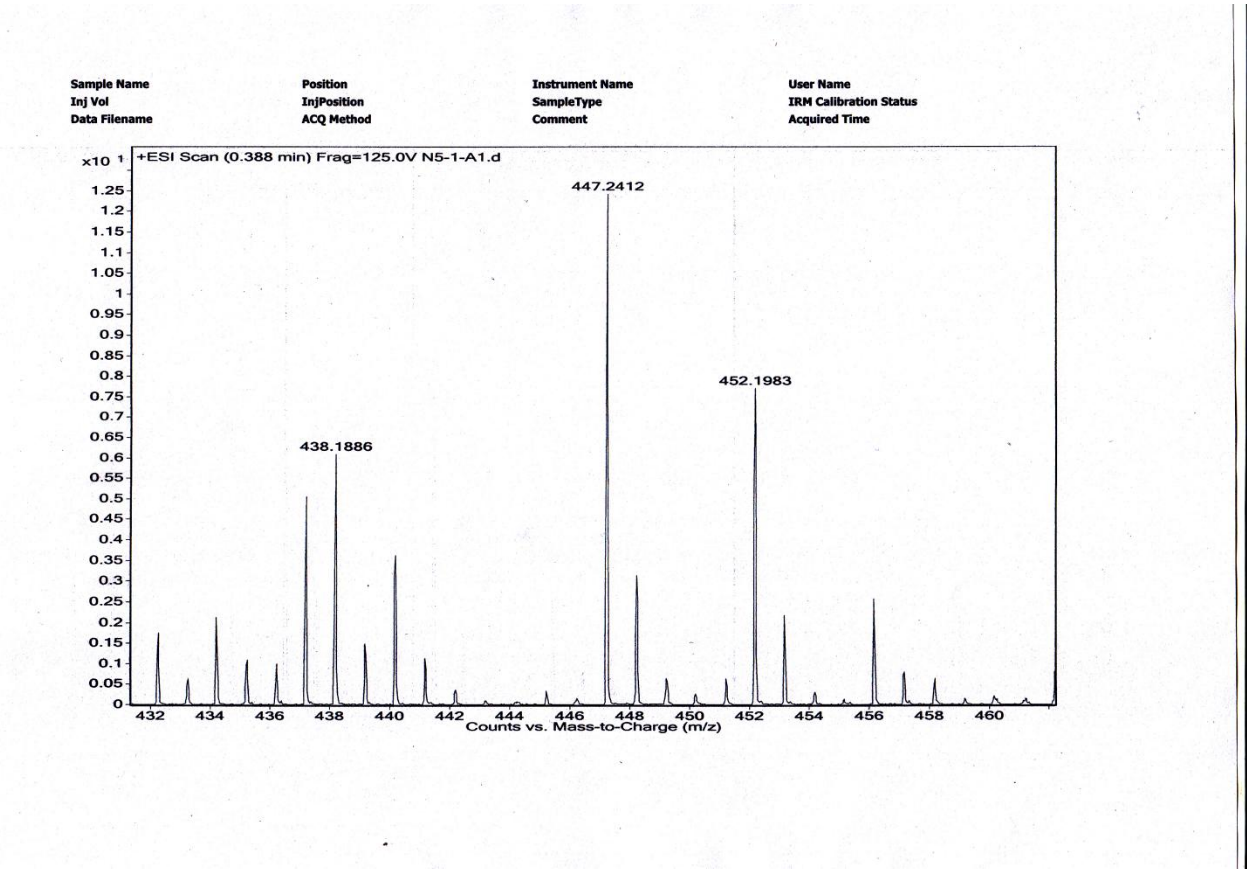

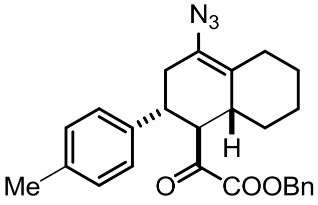

4c

Chemical Formula: $C_{26}H_{27}N_3O_3$
Exact Mass: 429.2052
Molecular Weight: 429.5109
m/z: 429.2052 (100.0%), 430.2086 (28.1%), 431.2120 (3.8%), 430.2023 (1.1%)

HRMS exact mass calcd for $C_{26}H_{27}N_3NaO_3$ $[M + Na]^+$ **452.1950**, found **452.1983**.

Supplementary Figure 99. ^{1}H and ^{13}C NMR spectra for 4d

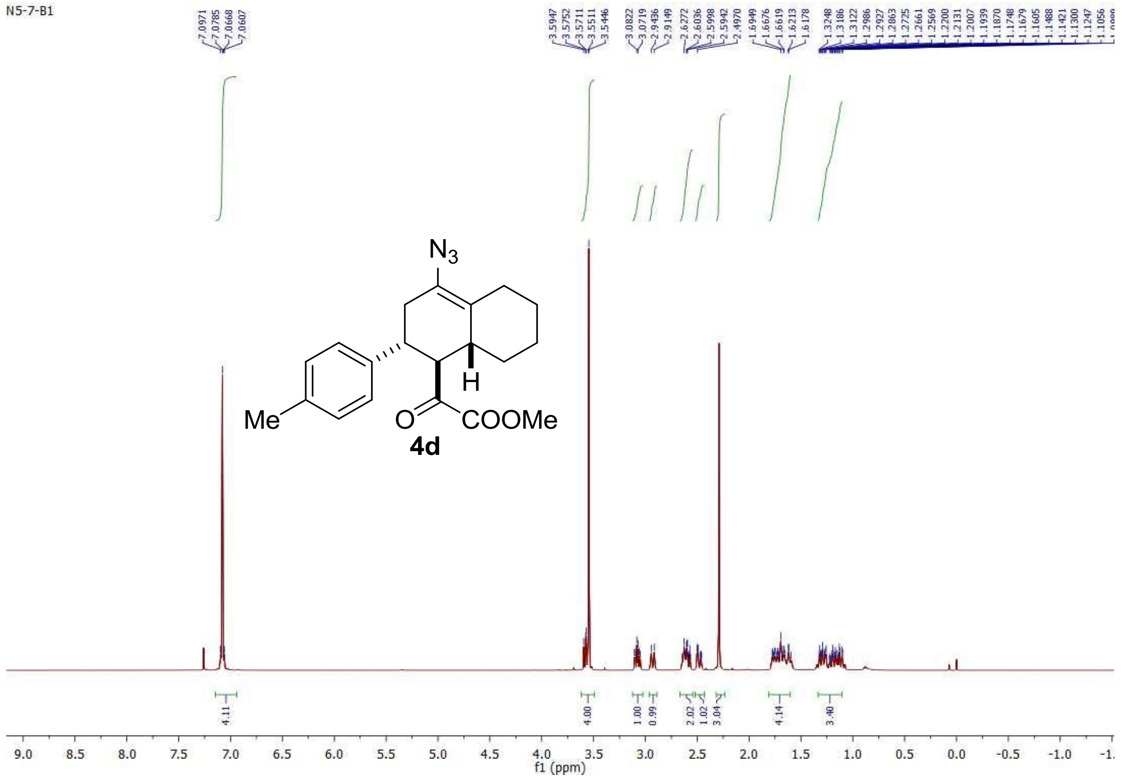

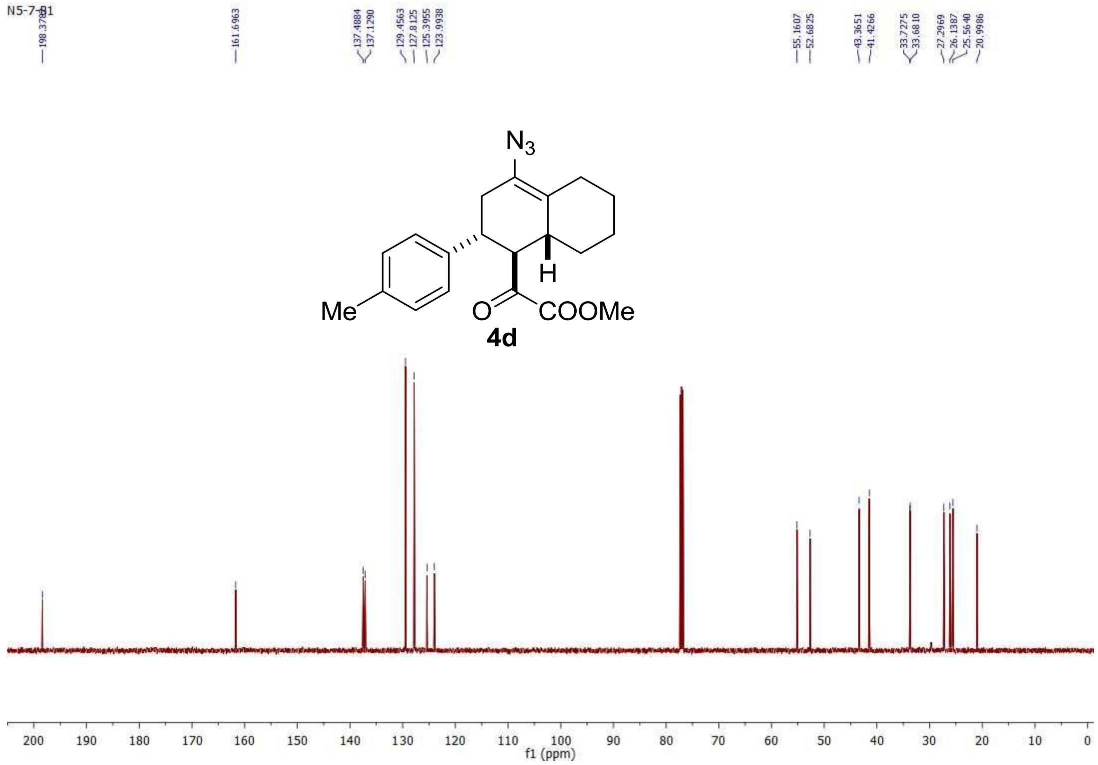

Supplementary Figure 100. HRMS spectra for 4d

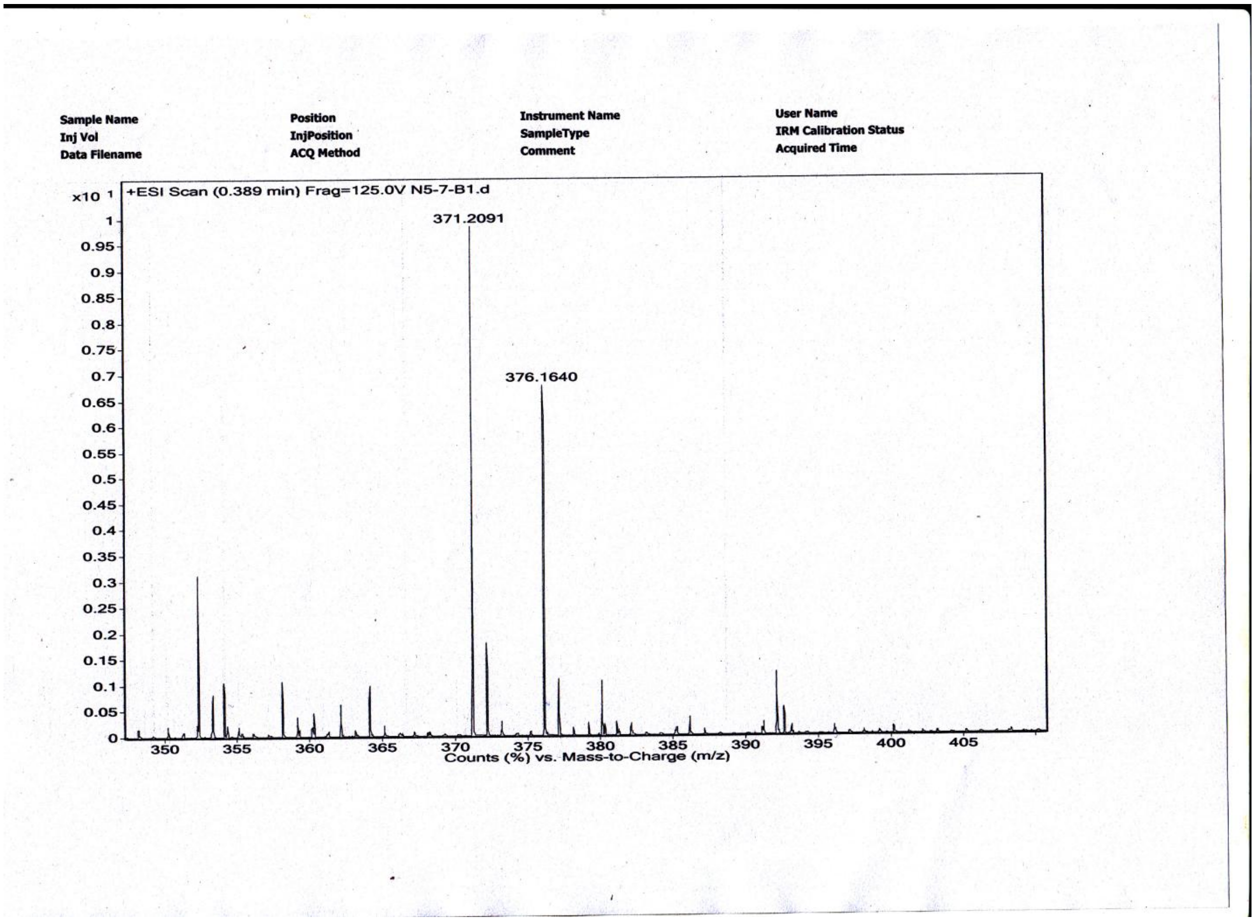

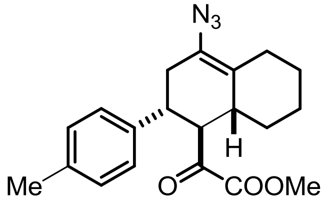

4d

Chemical Formula: $C_{20}H_{23}N_3O_3$
Exact Mass: 353.1739
Molecular Weight: 353.4149
m/z: 353.1739 (100.0%), 354.1773 (21.6%), 355.1807 (2.2%), 354.1710 (1.1%)

HRMS exact mass calcd for $C_{20}H_{23}N_3NaO_3$ $[M + Na]^+$ **376.1637**, found **376.1640**.

Supplementary Figure 101. ^{1}H and ^{13}C NMR spectra for 4e

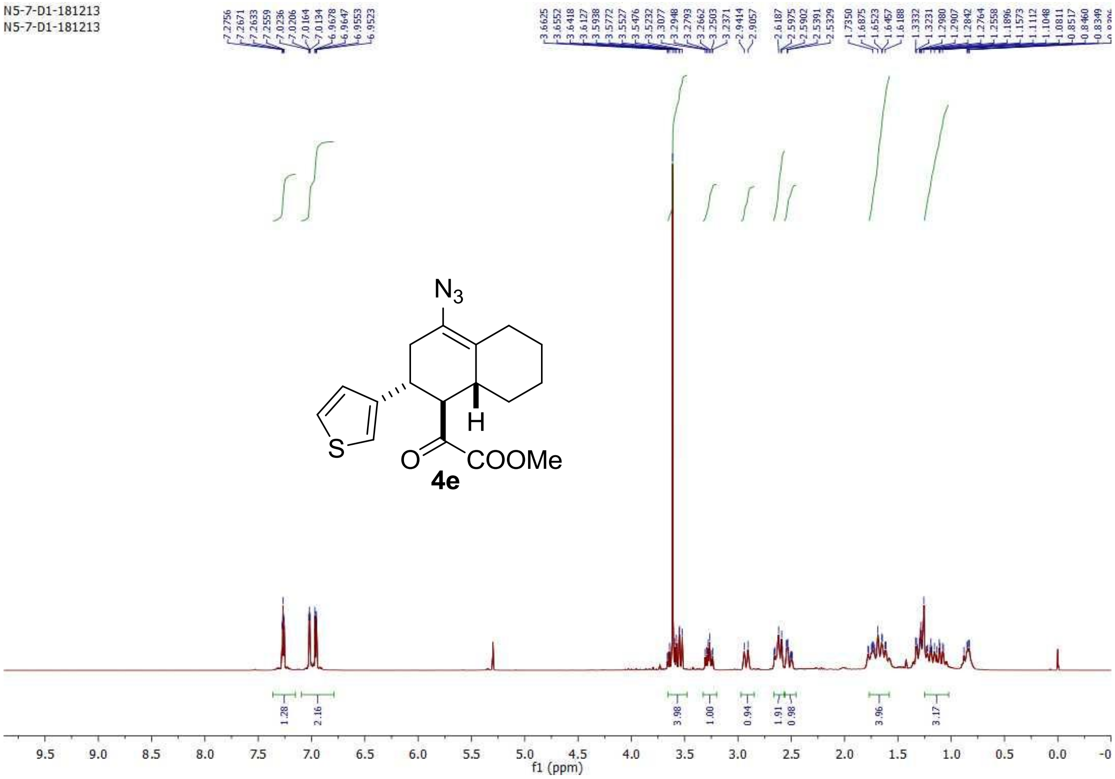

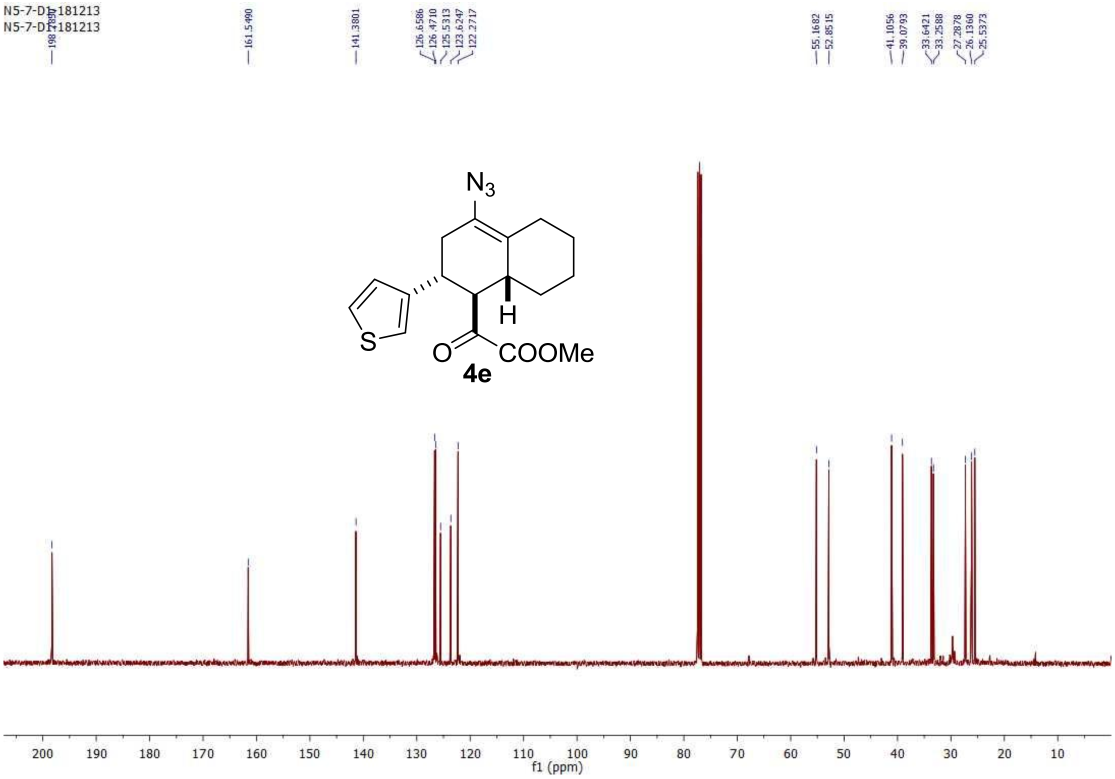

Supplementary Figure 102. HRMS spectra for 4e

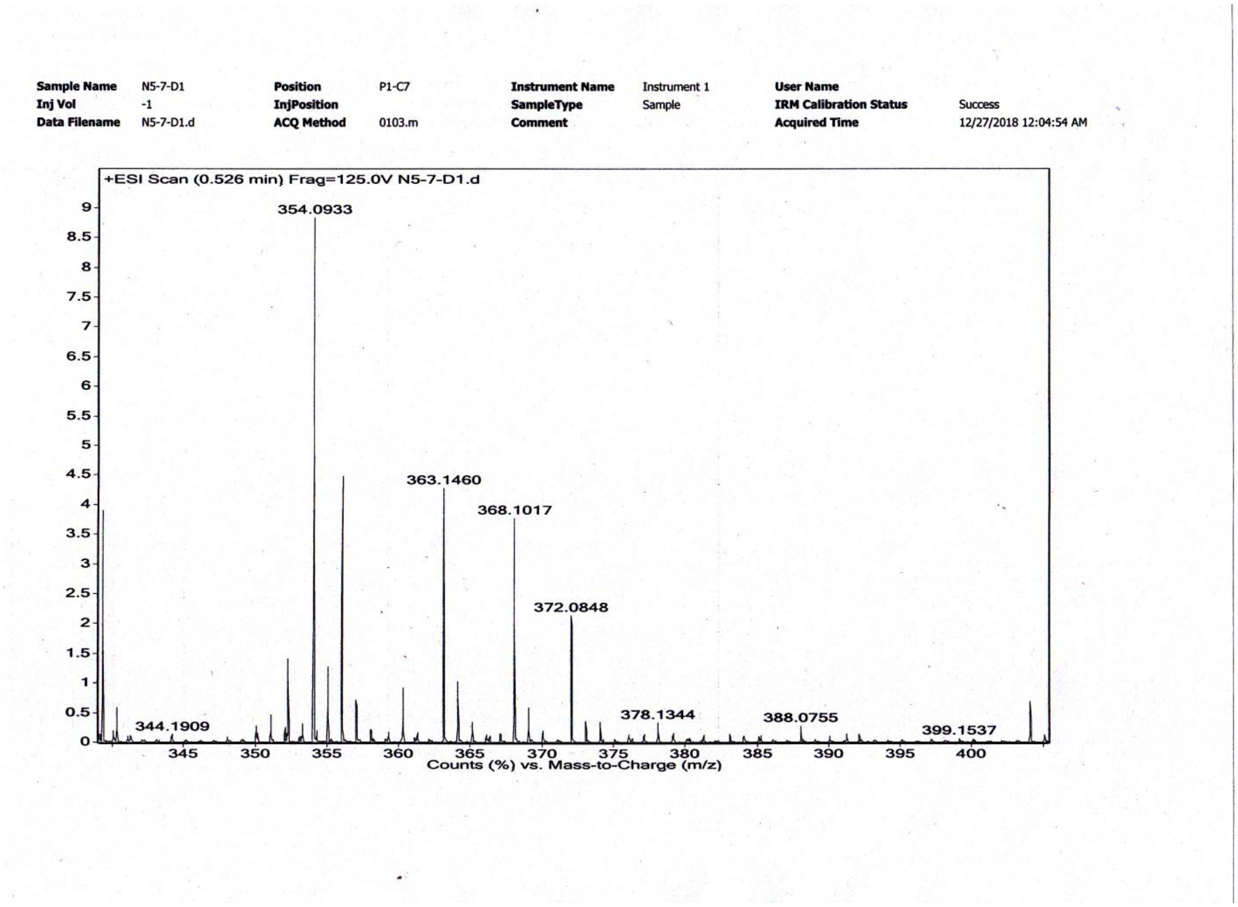

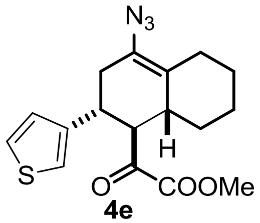

4e

Chemical Formula: $C_{17}H_{19}N_3O_3S$
Exact Mass: 345.1147
Molecular Weight: 345.4161
m/z: 345.1147 (100.0%), 346.1181 (18.4%), 347.1105 (4.5%), 347.1214 (1.6%), 346.1117 (1.1%)

HRMS exact mass calcd for $C_{17}H_{19}N_3NaO_3S$ $[M + Na]^+$ **368.1045**, found **368.1017**.

Supplementary Figure 103. ^{1}H and ^{13}C NMR spectra for 4f

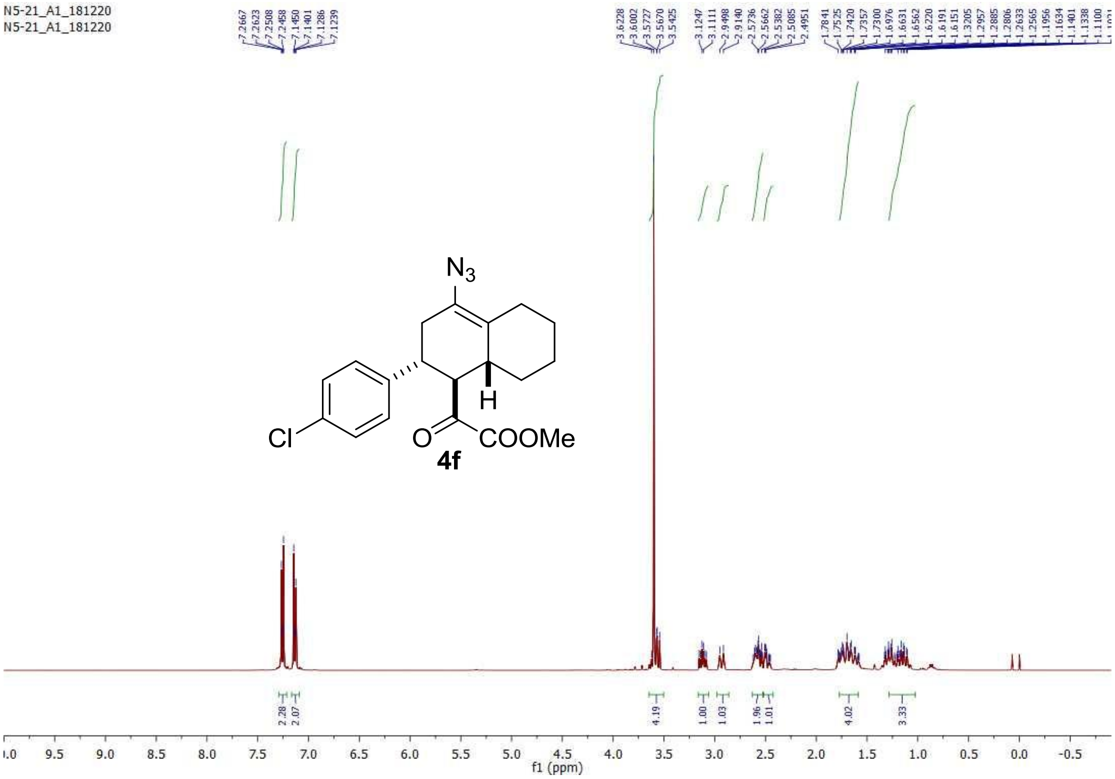

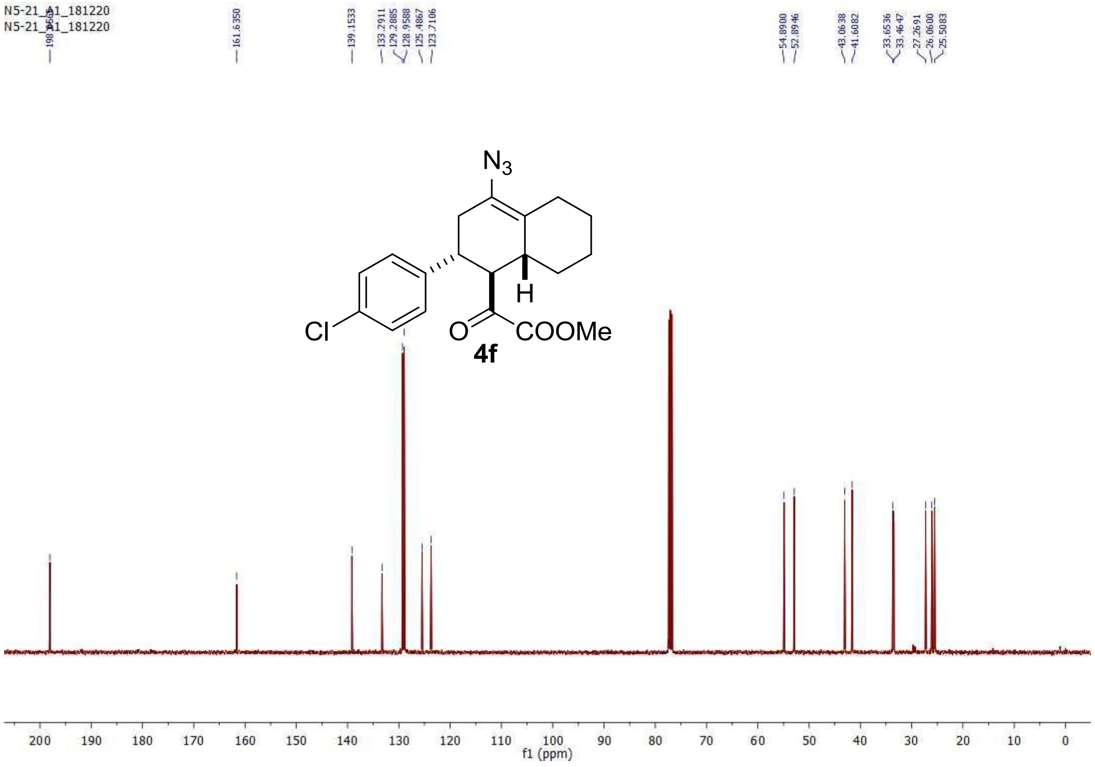

Supplementary Figure 104. HRMS spectra for 4f

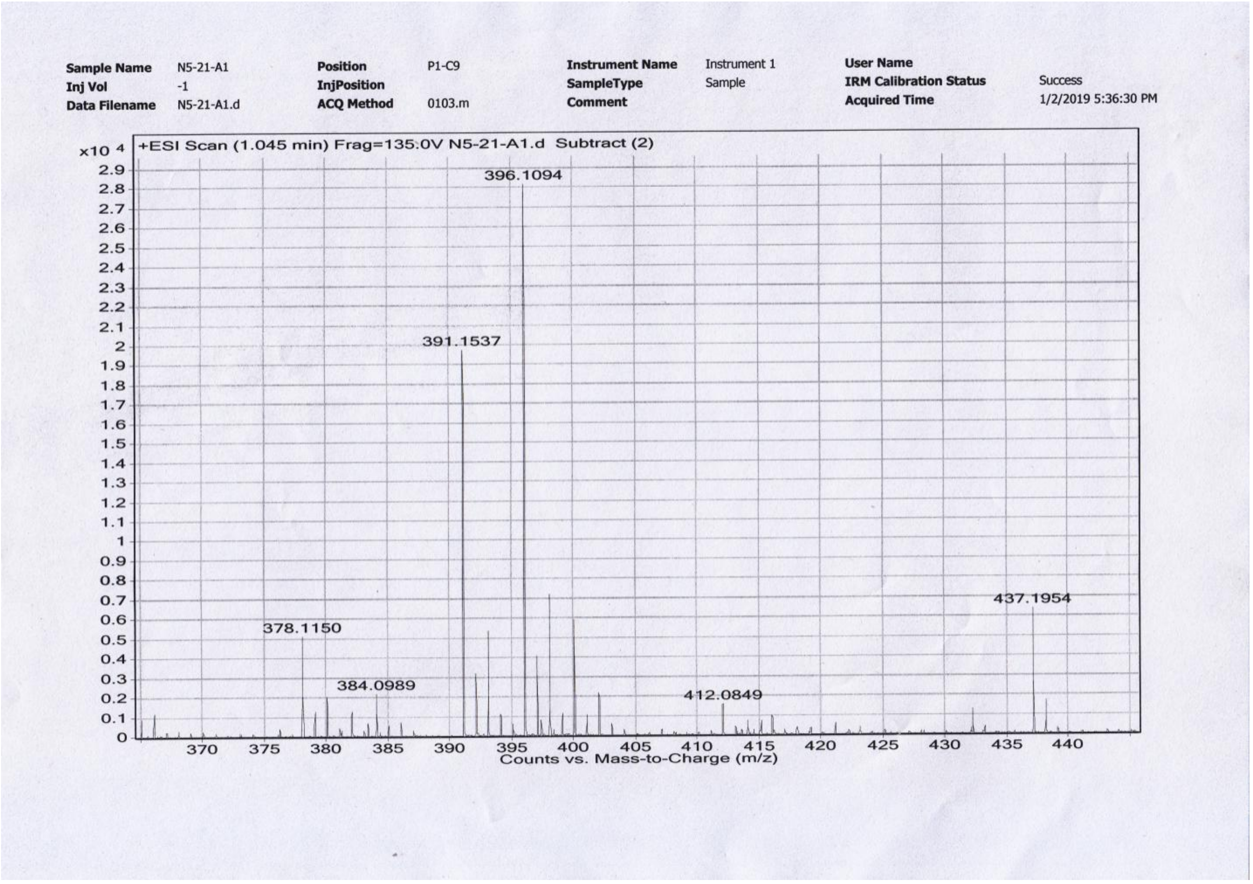

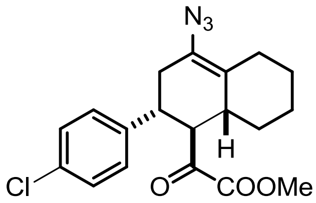

4f

Chemical Formula: $C_{19}H_{20}ClN_3O_3$
Exact Mass: 373.1193
Molecular Weight: 373.8334
m/z: 373.1193 (100.0%), 375.1164 (32.0%), 374.1227 (20.5%), 376.1197 (6.6%), 375.1260 (2.0%), 374.1164 (1.1%)

HRMS exact mass calcd for $C_{19}H_{20}ClN_3NaO_3$ $[M + Na]^+$ **396.1091**, found **396.1094**.

Supplementary Figure 105. ^{1}H and ^{13}C NMR spectra for 4g

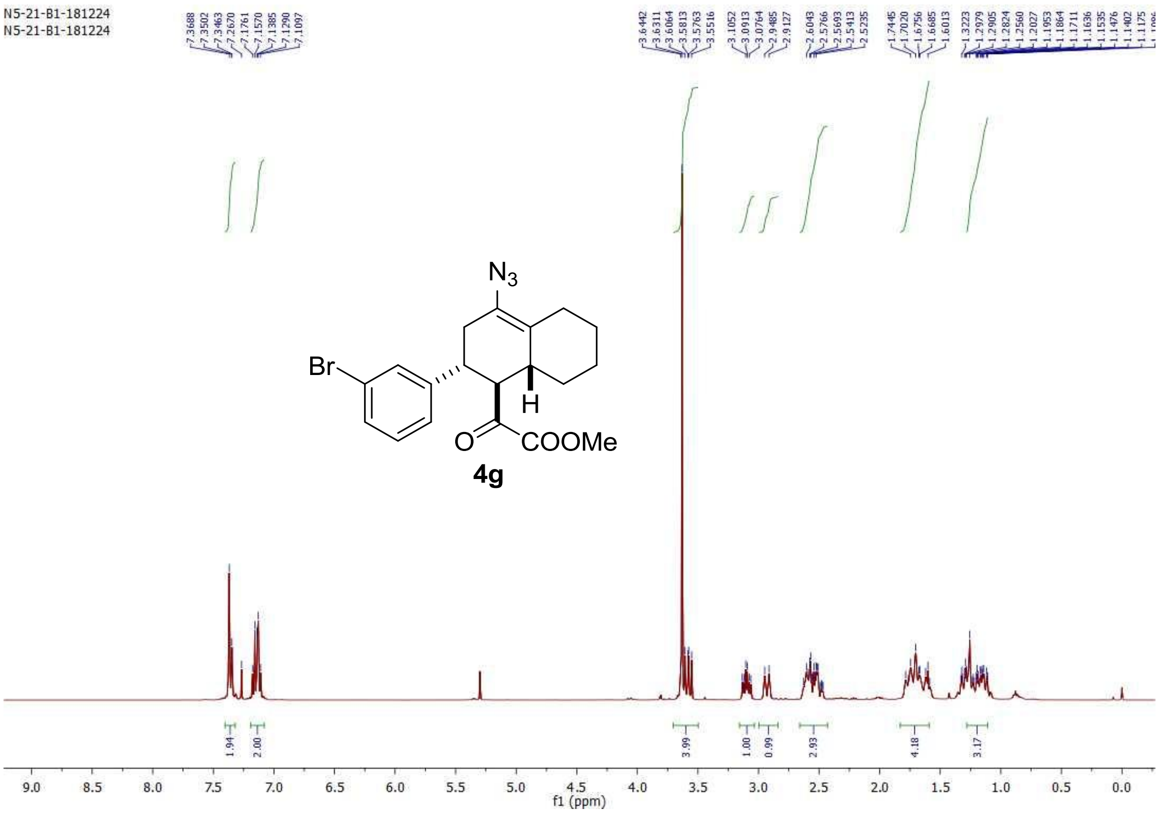

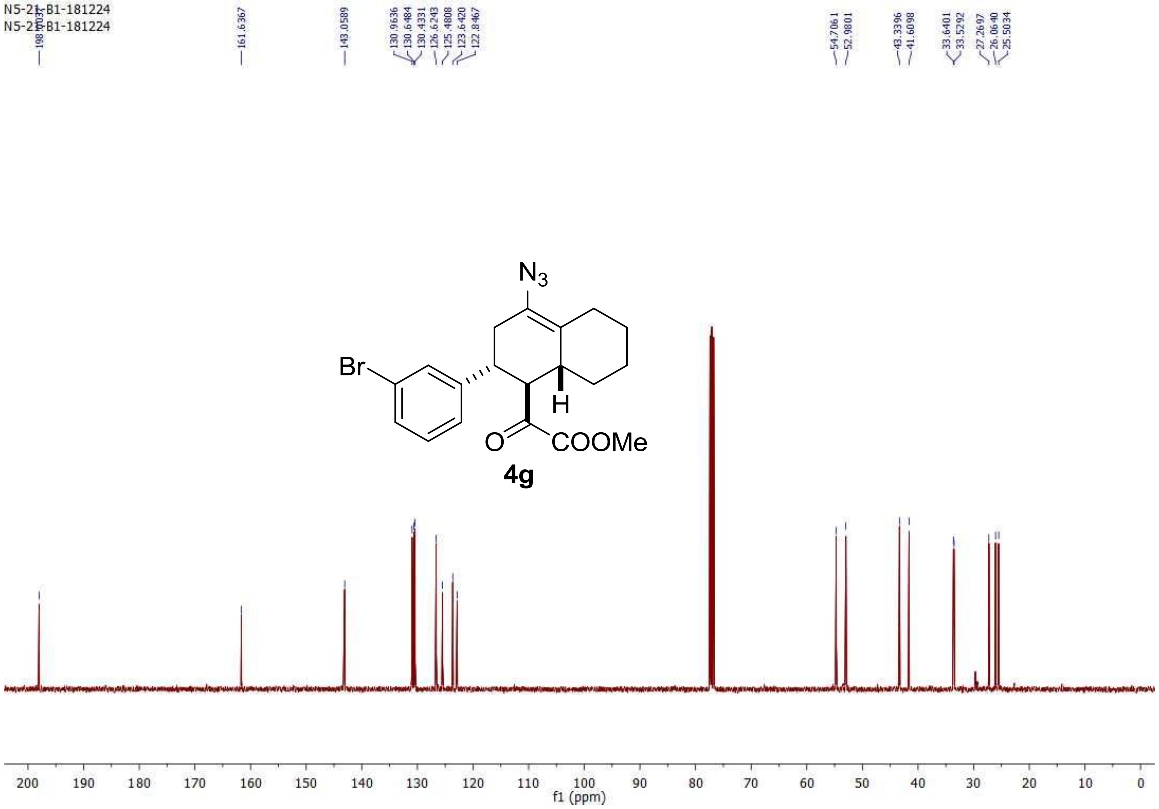

Supplementary Figure 106. HRMS spectra for 4g

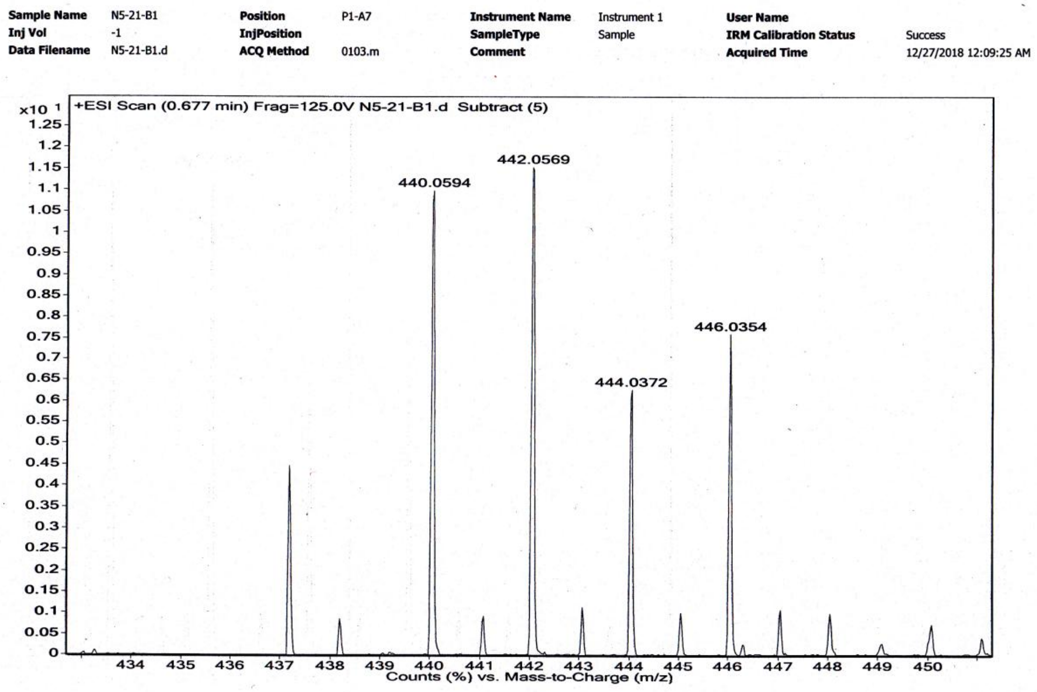

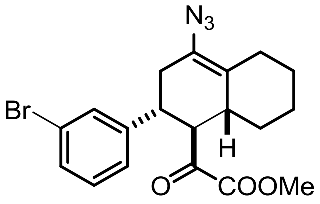

4g

Chemical Formula: $C_{19}H_{20}BrN_3O_3$
Exact Mass: 417.0688
Molecular Weight: 418.2844
m/z: 417.0688 (100.0%), 419.0668 (97.3%), 418.0722 (20.5%), 420.0701 (20.0%), 419.0755 (2.0%), 421.0735 (1.9%), 418.0658 (1.1%), 420.0638 (1.1%)

HRMS exact mass calcd for $C_{19}H_{20}BrN_3NaO_3$ $[M + Na]^+$ **440.0586**, found **440.0594**.

Supplementary Figure 107. ^{1}H and ^{13}C NMR spectra for 4h

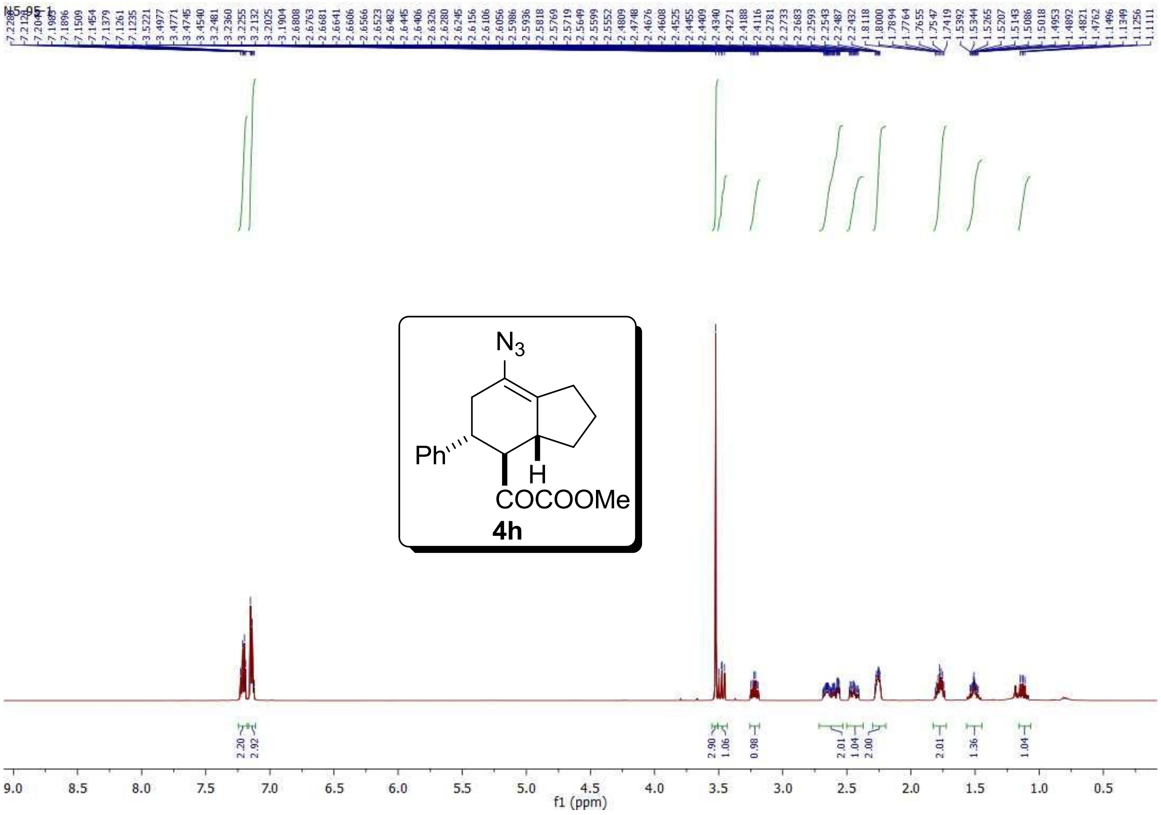

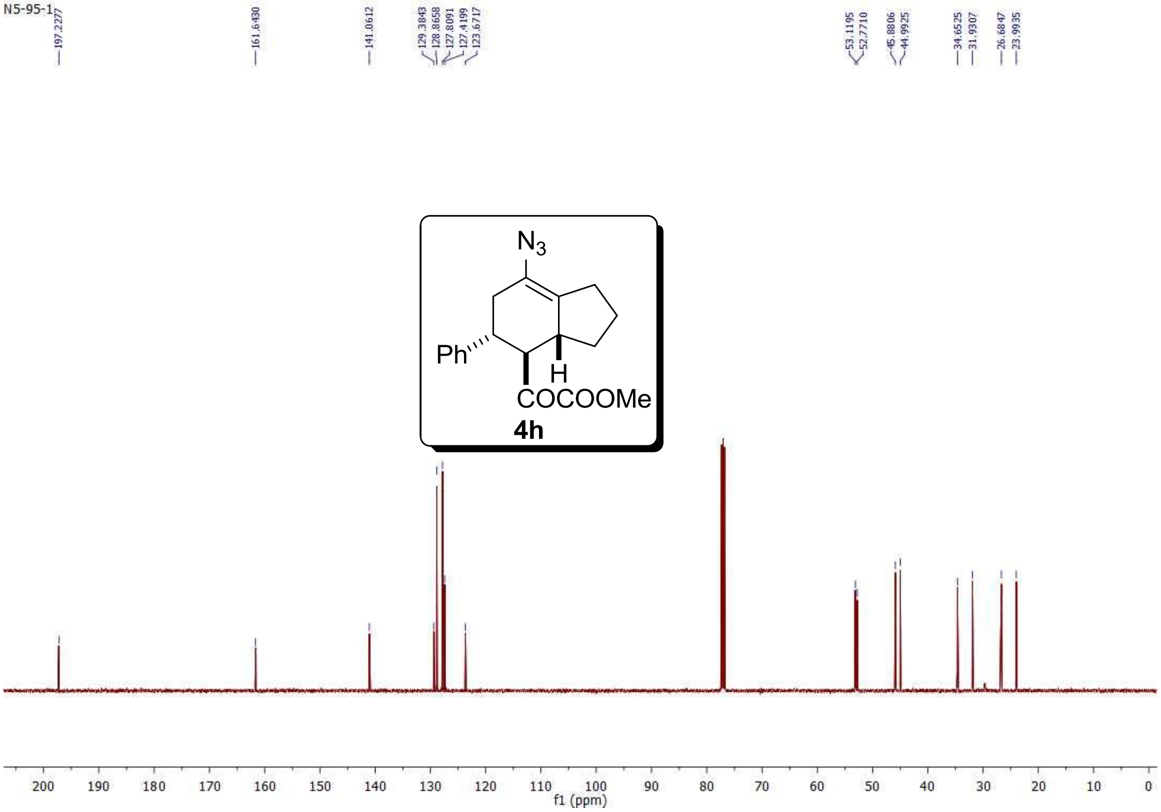

Supplementary Figure 108. HRMS spectra for 4h

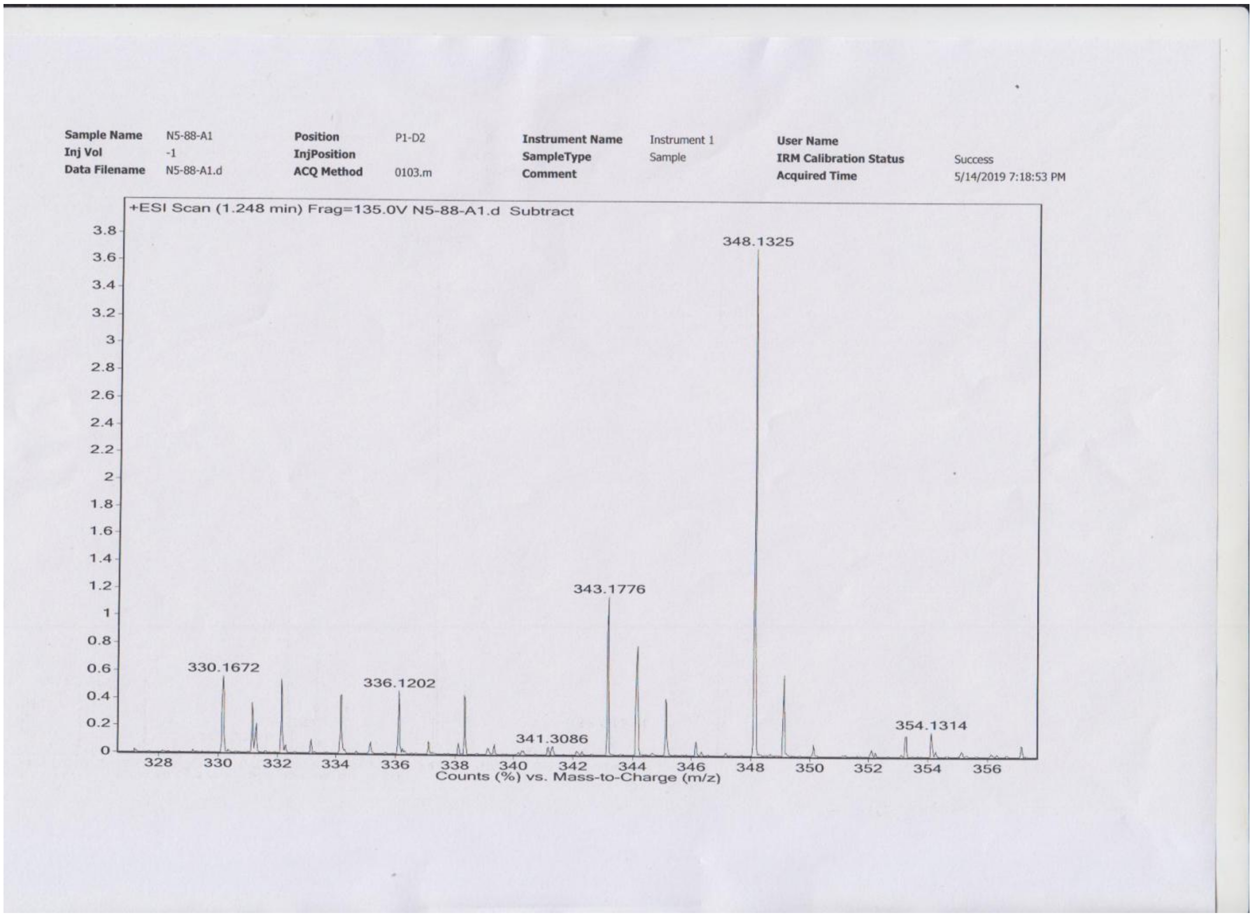

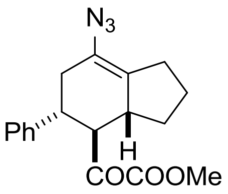

4h

Chemical Formula: $C_{18}H_{19}N_3O_3$

Exact Mass: 325.1426

Molecular Weight: 325.3618

m/z: 325.1426 (100.0%), 326.1460 (19.5%), 327.1494 (1.8%), 326.1397 (1.1%)

HRMS exact mass calcd for $C_{18}H_{19}N_3NaO_3$ $[M + Na]^+$ **348.1324**, found **348.1325**.

Supplementary Figure 109. ^{1}H and ^{13}C NMR spectra for 5

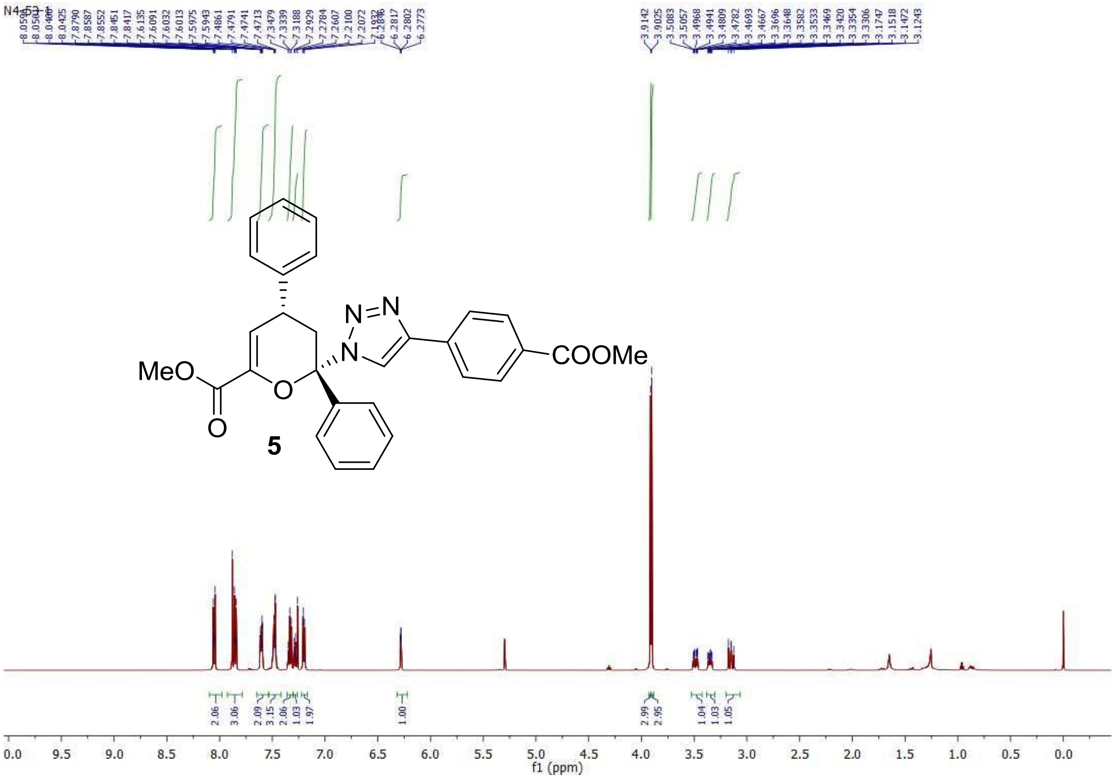

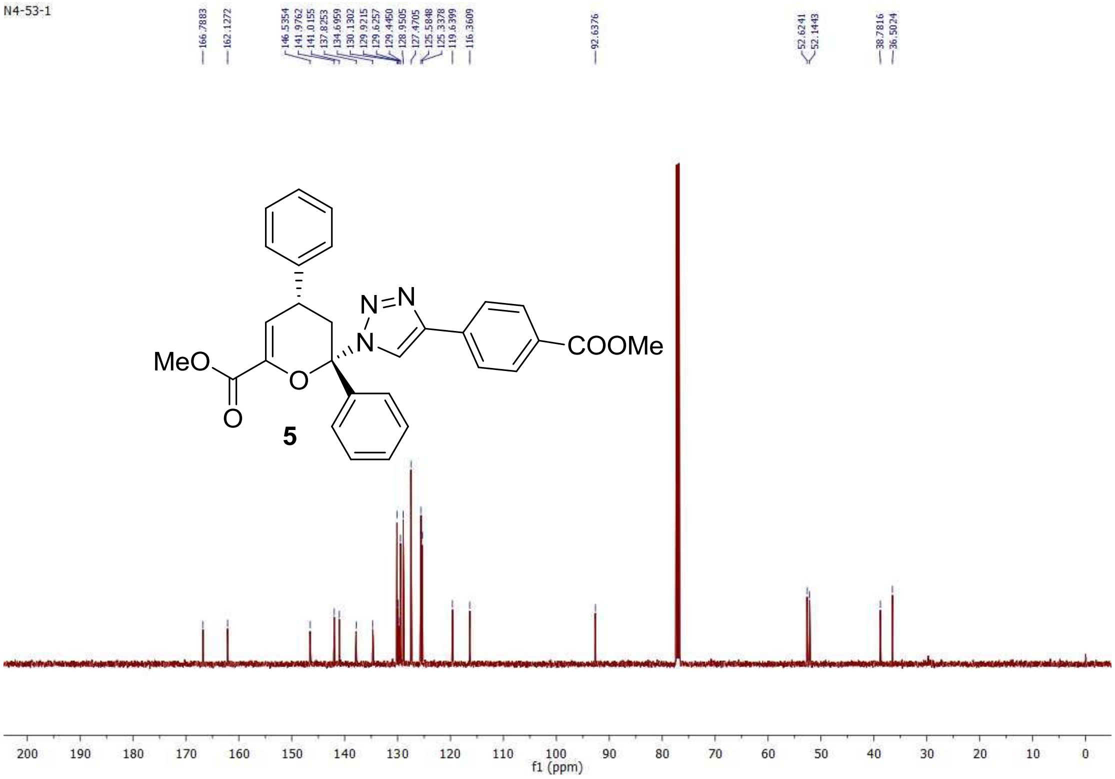

Supplementary Figure 110. HRMS spectra for 5

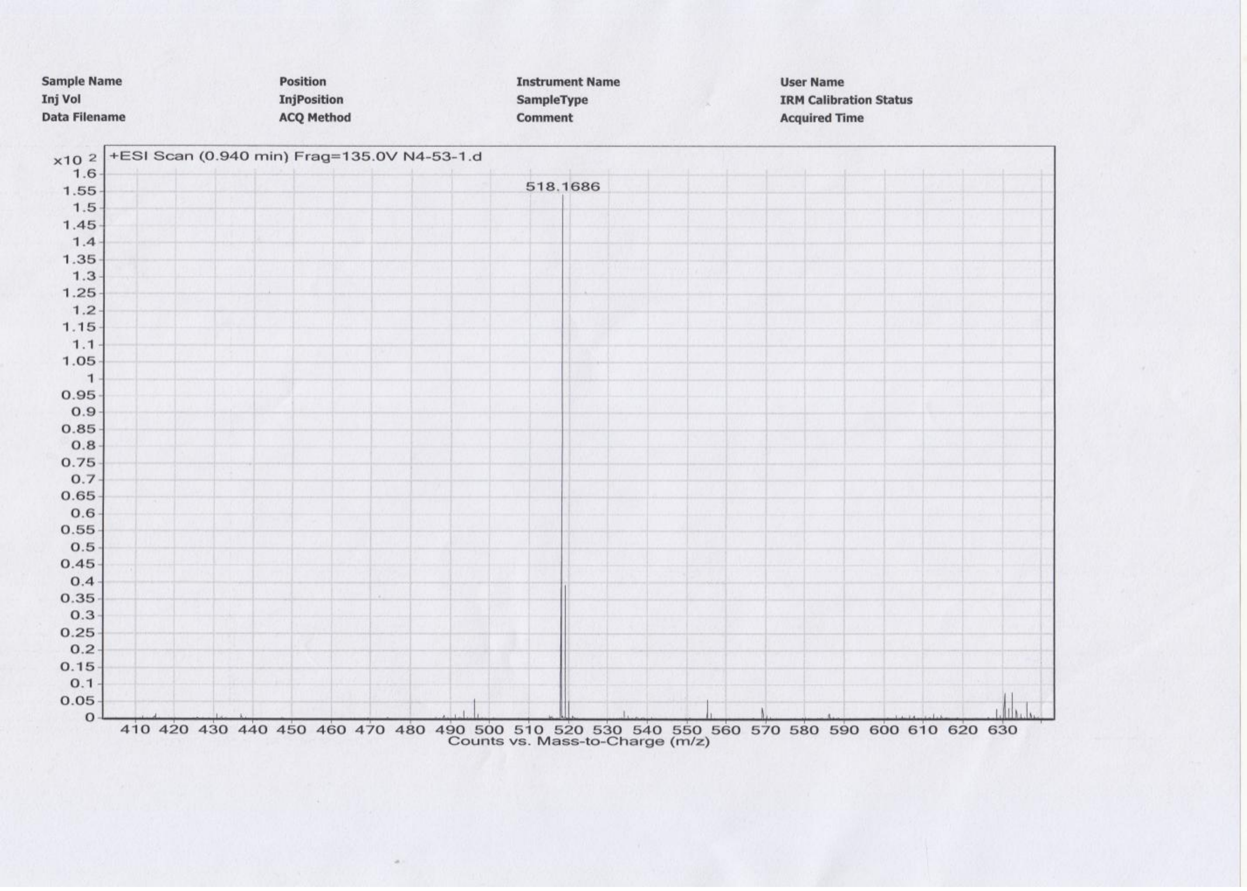

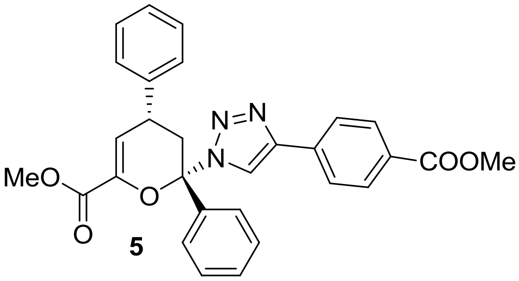

Chemical Formula: $C_{29}H_{25}N_3O_5$
Exact Mass: 495.1794
Molecular Weight: 495.5259
m/z: 495.1794 (100.0%), 496.1828 (31.4%), 497.1861 (4.7%), 496.1765 (1.1%), 497.1837 (1.0%)

HRMS exact mass calcd for $C_{29}H_{25}N_3NaO_5$ $[M + Na]^+$ **518.1692**, found **518.1686**.

Supplementary Figure 111. ^{1}H and ^{13}C NMR spectra for 6

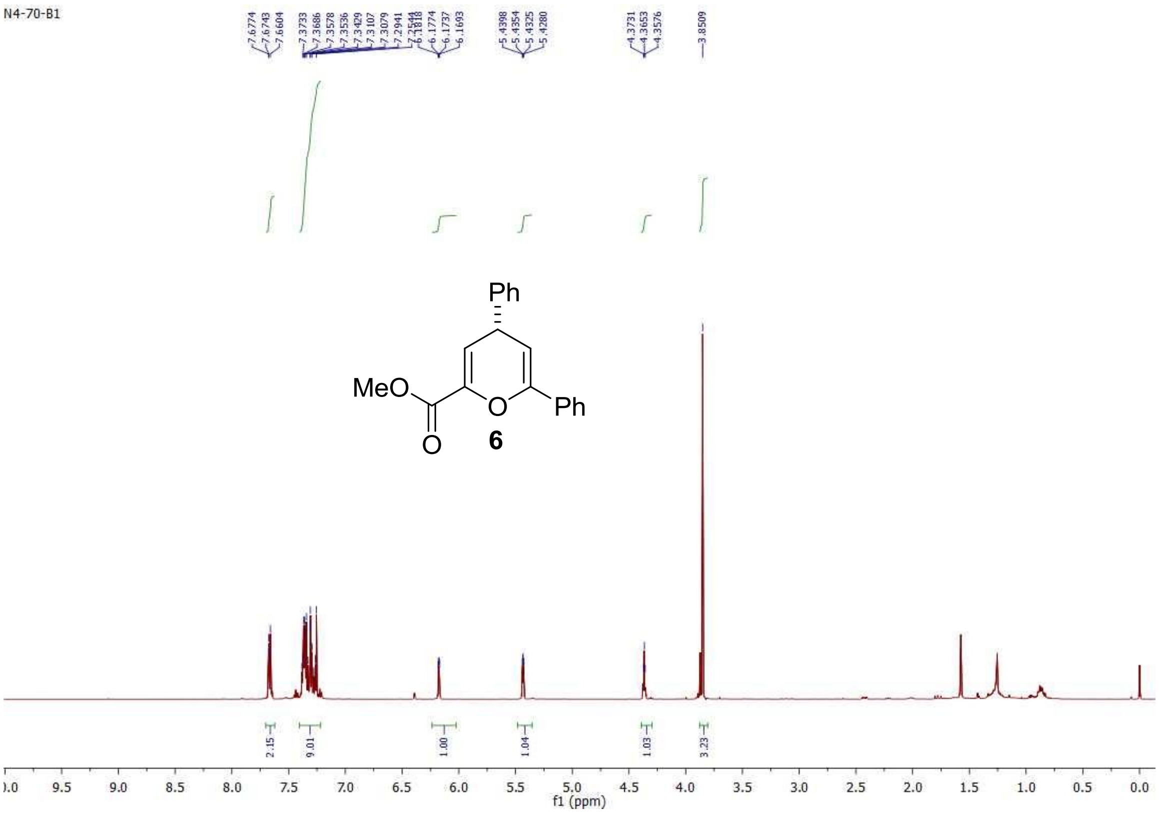

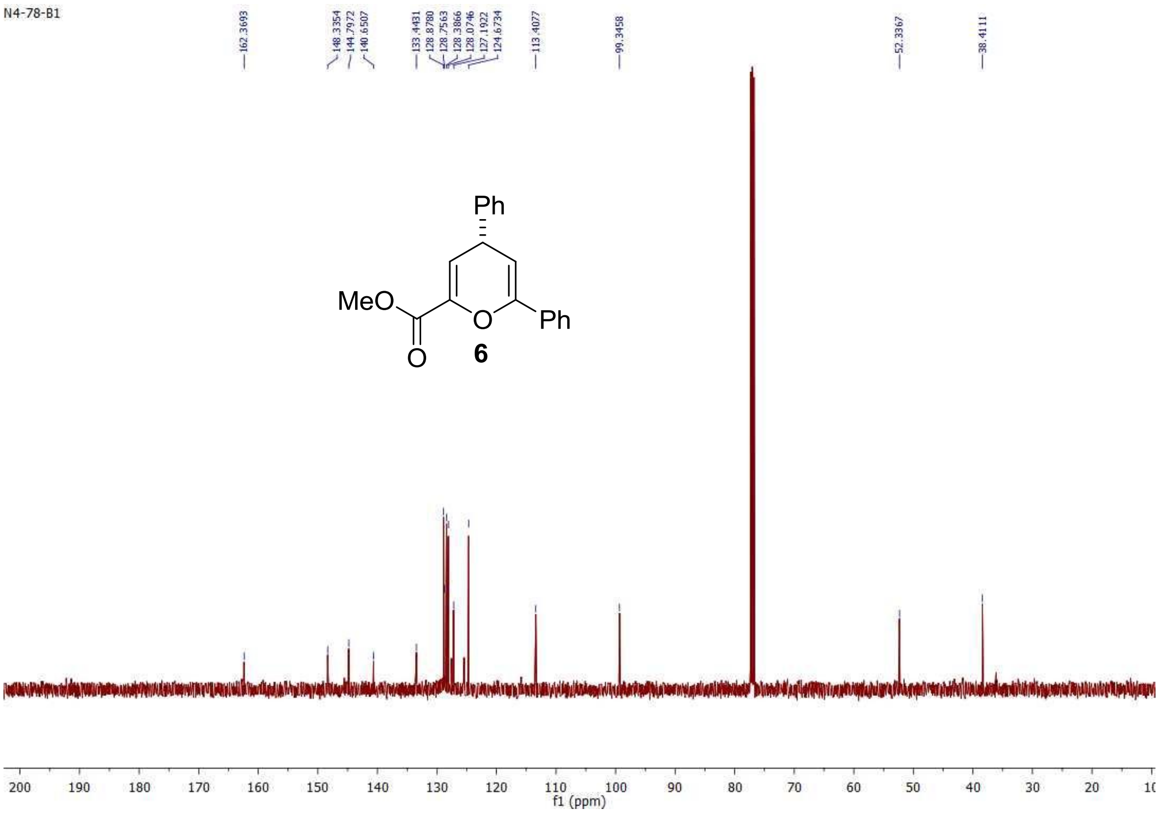

Supplementary Figure 112. HRMS spectra for 6

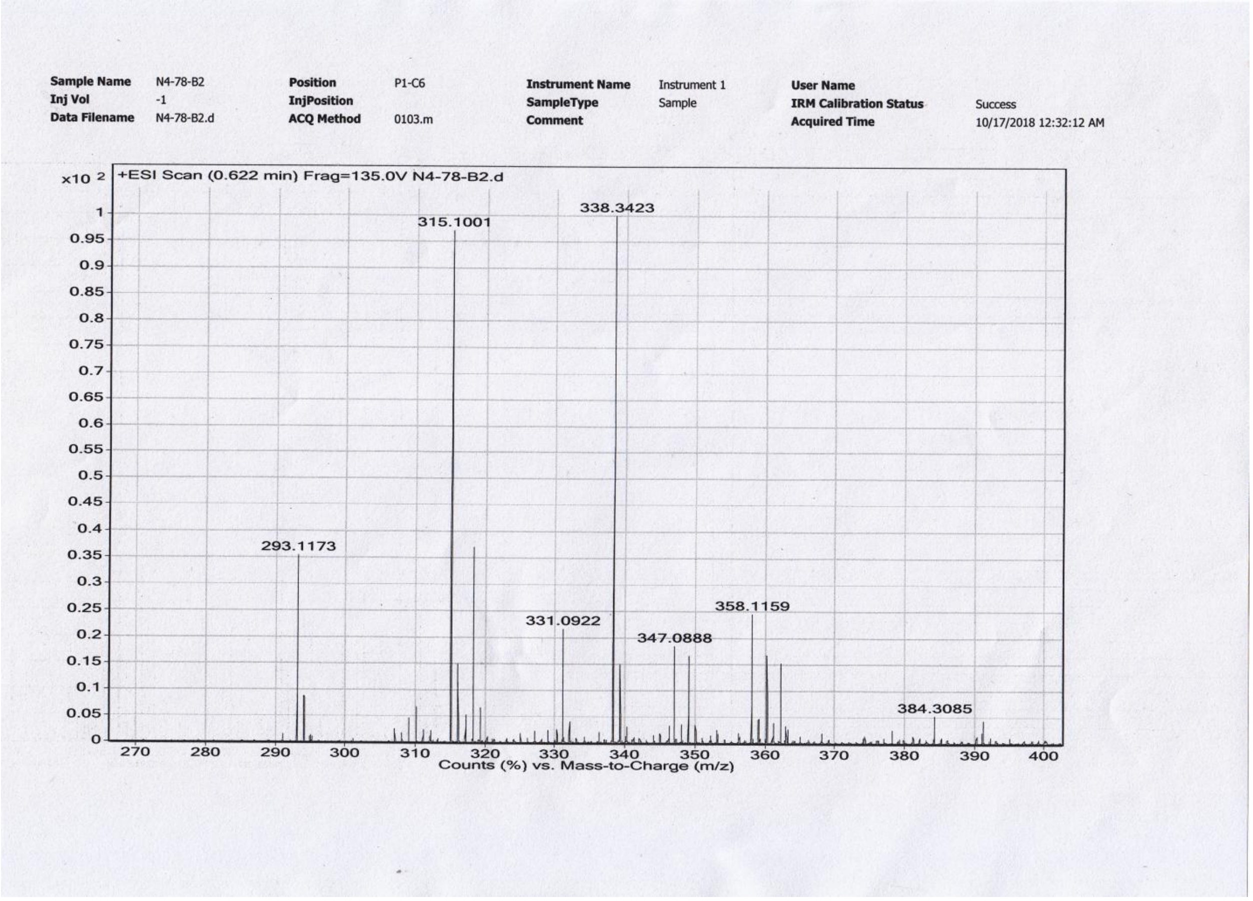

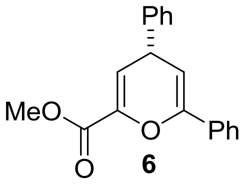

6

Chemical Formula: $C_{19}H_{16}O_3$
Exact Mass: 292.1099
Molecular Weight: 292.3285
m/z: 292.1099 (100.0%), 293.1133 (20.5%), 294.1167 (2.0%)

HRMS exact mass calcd for $C_{19}H_{16}NaO_3$ $[M + Na]^+$ **315.0997**, found **315.1001**.

Supplementary Figure 113. ^{1}H and ^{13}C NMR spectra for 7

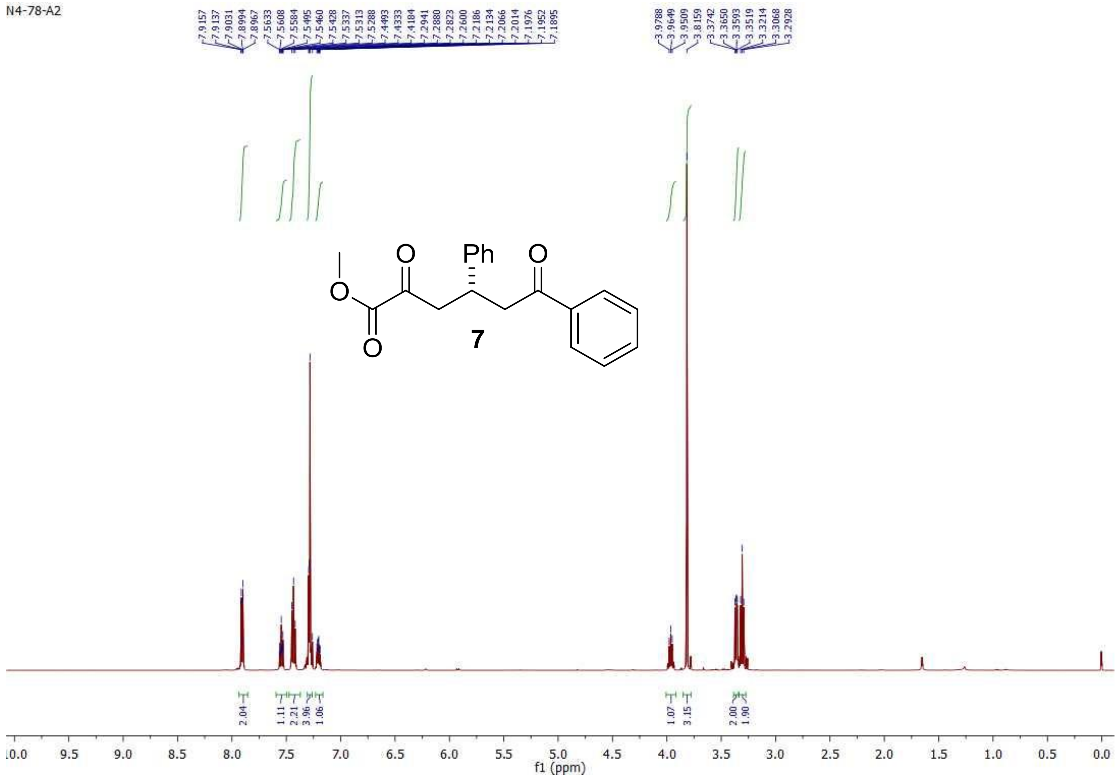

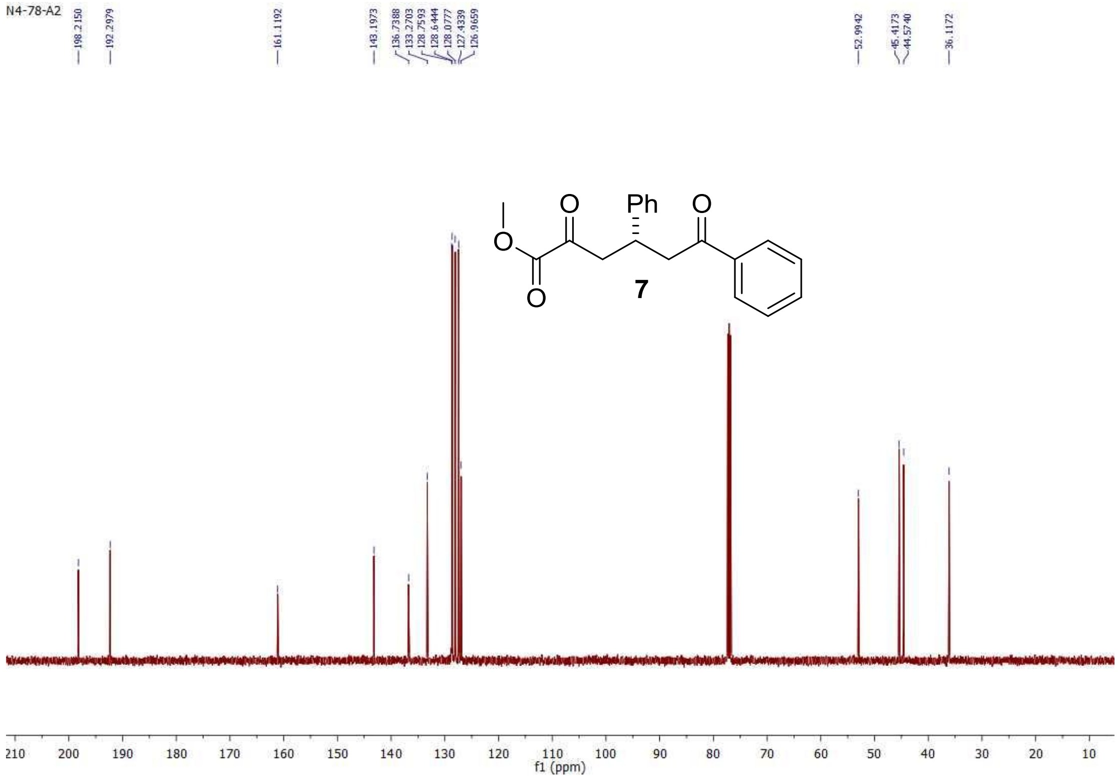

Supplementary Figure 114. HRMS spectra for 7

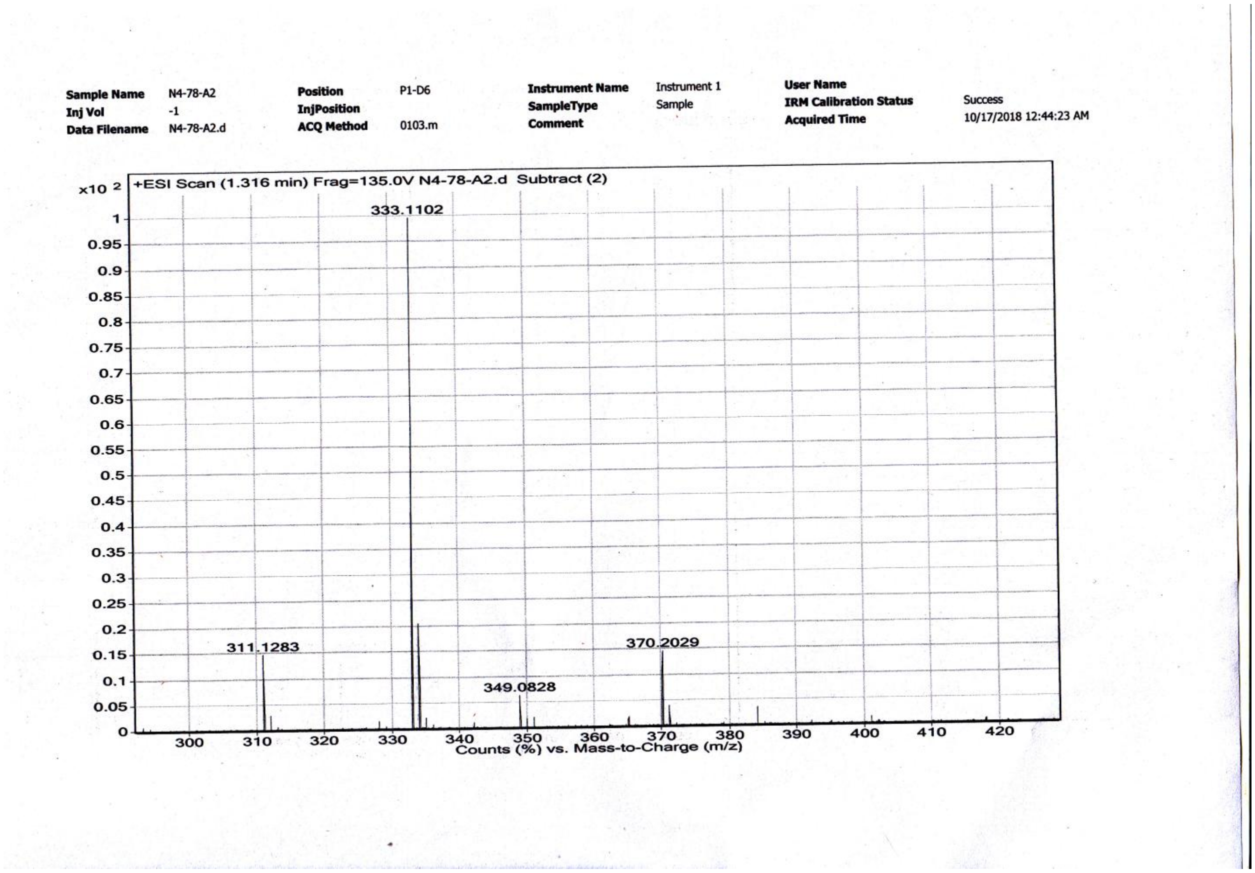

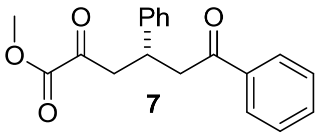

Chemical Formula: $C_{19}H_{18}O_4$
Exact Mass: 310.1205
Molecular Weight: 310.3438
m/z: 310.1205 (100.0%), 311.1239 (20.5%), 312.1272 (2.0%)

HRMS exact mass calcd for $C_{19}H_{18}NaO_4$ $[M + Na]^+$ **333.1103**, found **333.1102**.

Supplementary Figure 115. ^{1}H and ^{13}C NMR spectra for 8

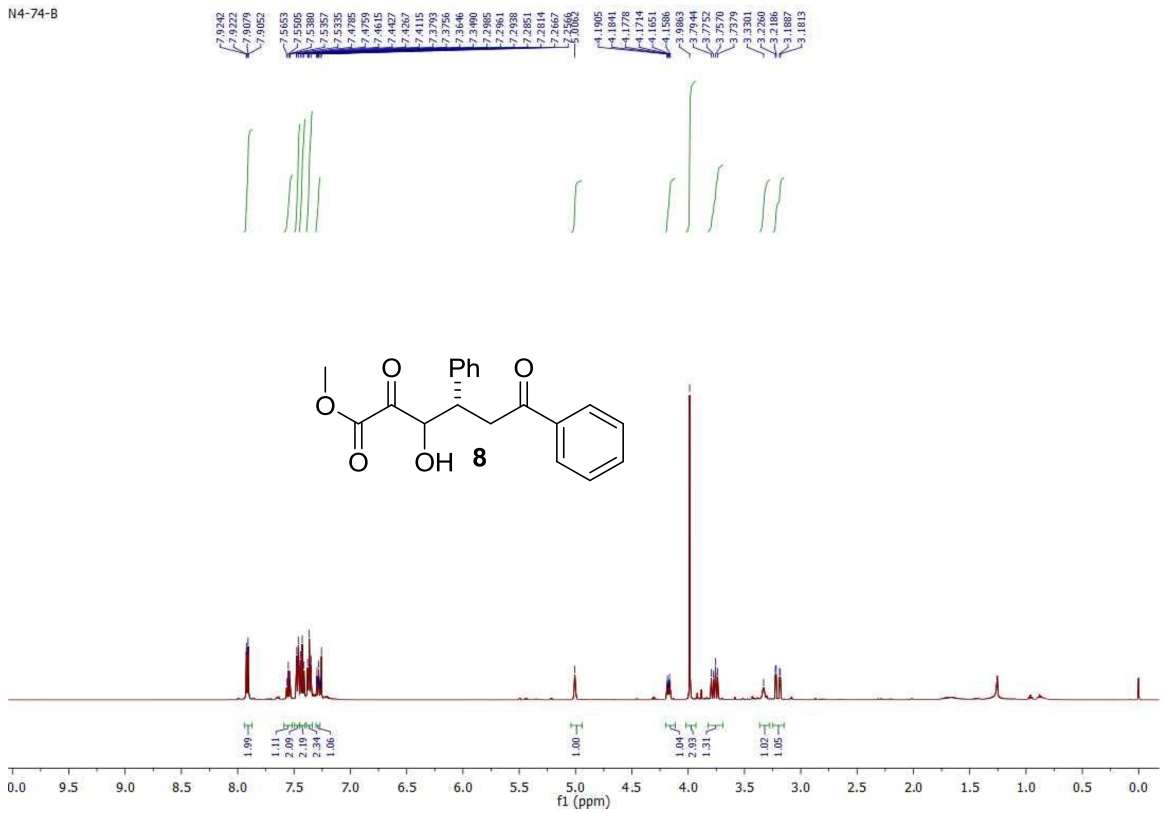

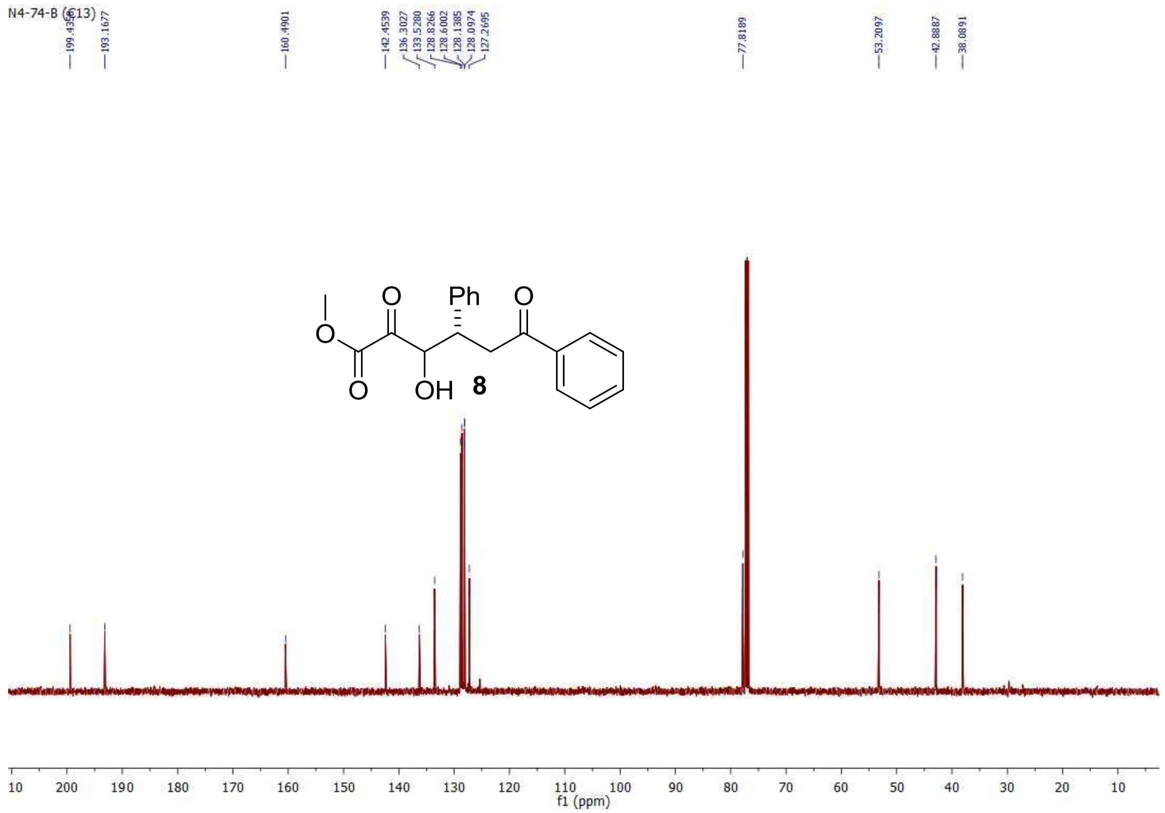

Supplementary Figure 116. HRMS spectra for 8

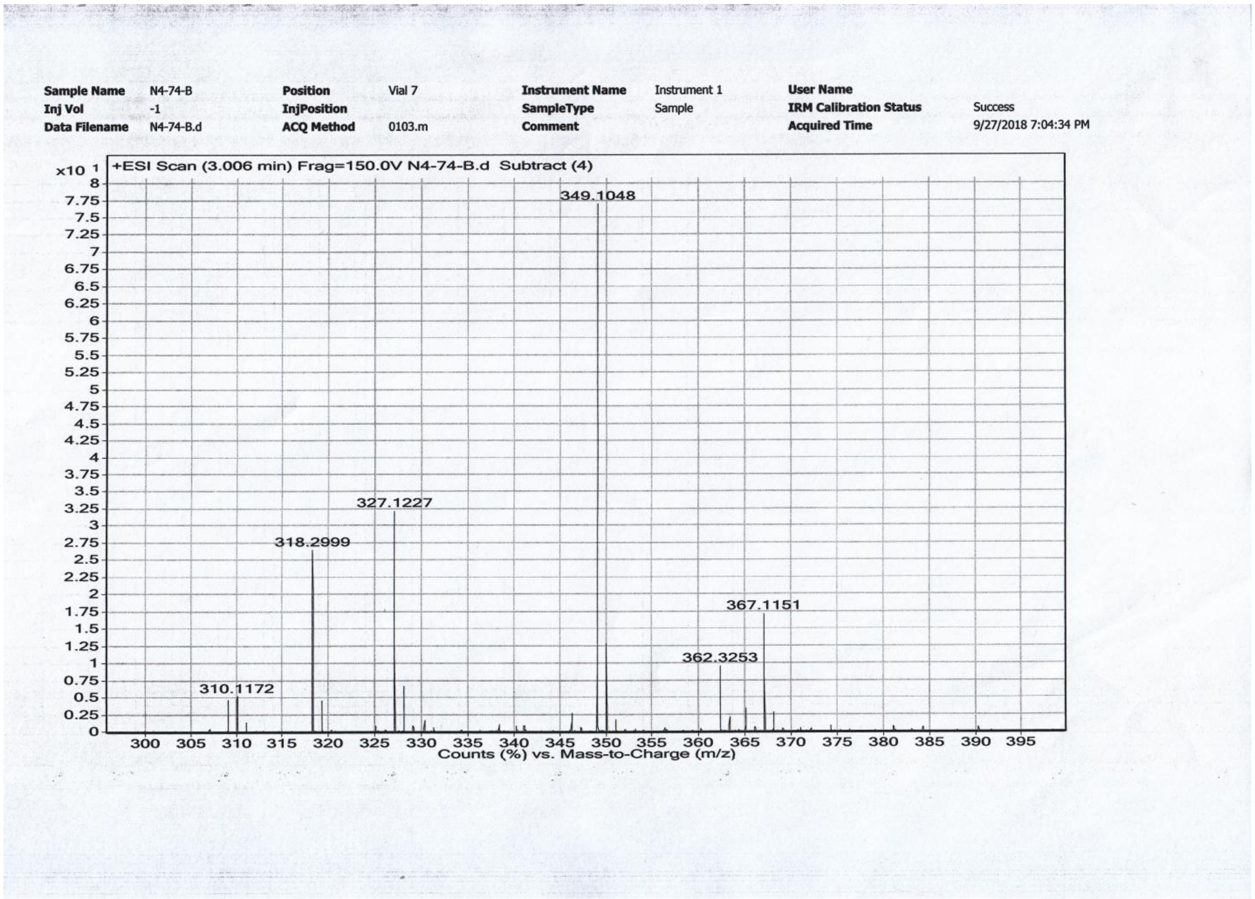

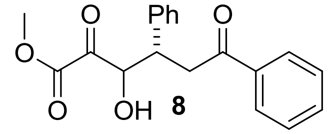

Chemical Formula: $C_{19}H_{18}O_5$
Exact Mass: 326.1154
Molecular Weight: 326.3432
m/z: 326.1154 (100.0%), 327.1188 (20.5%), 328.1221 (2.0%), 328.1197 (1.0%)

HRMS exact mass calcd for $C_{19}H_{18}NaO_5$ $[M + H]^+$ **349.1052**, found **349.1048**.

Supplementary Figure 117. ^{1}H and ^{13}C NMR spectra for 9

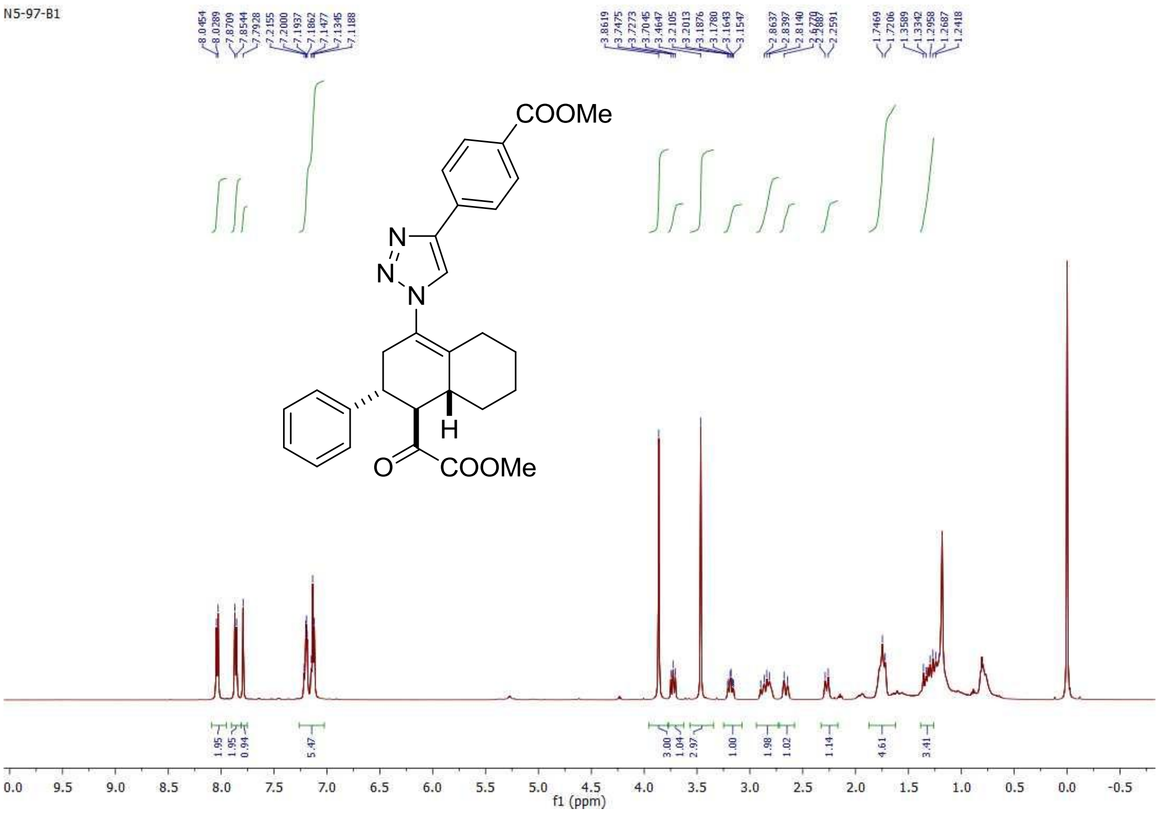

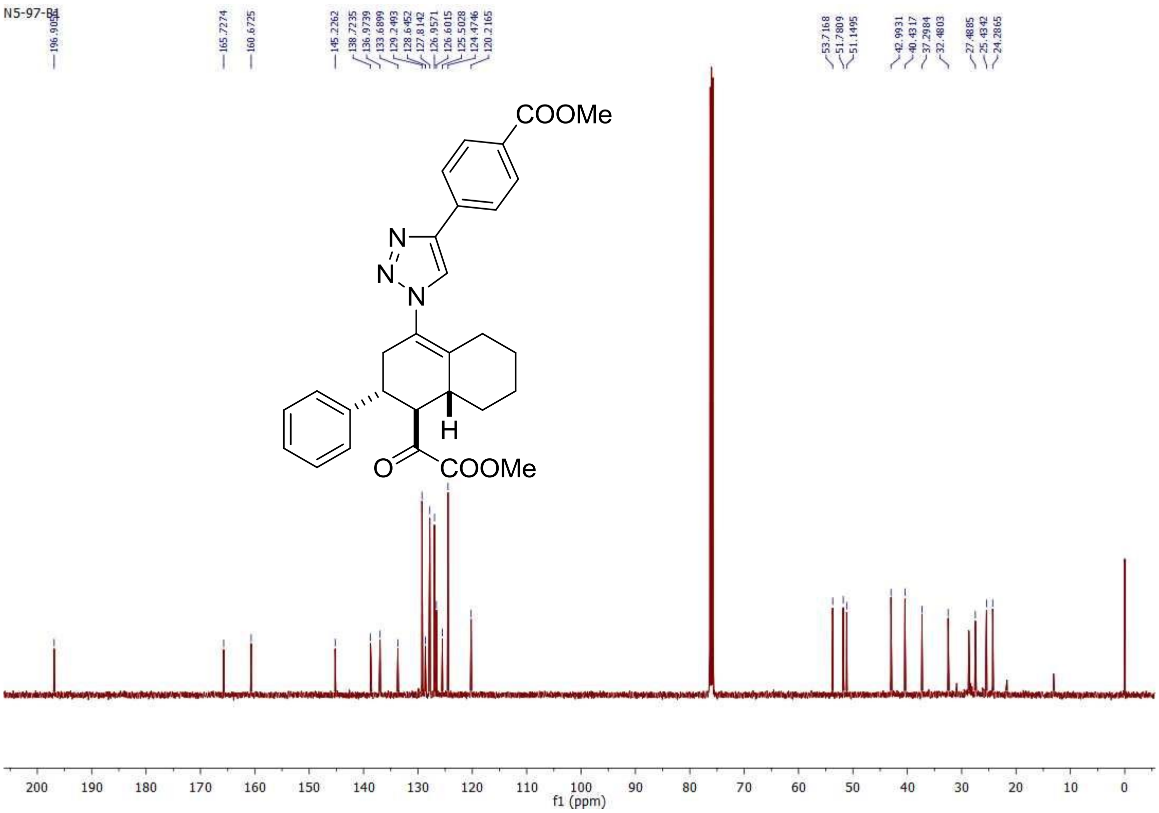

Supplementary Figure 118. HRMS spectra for 9

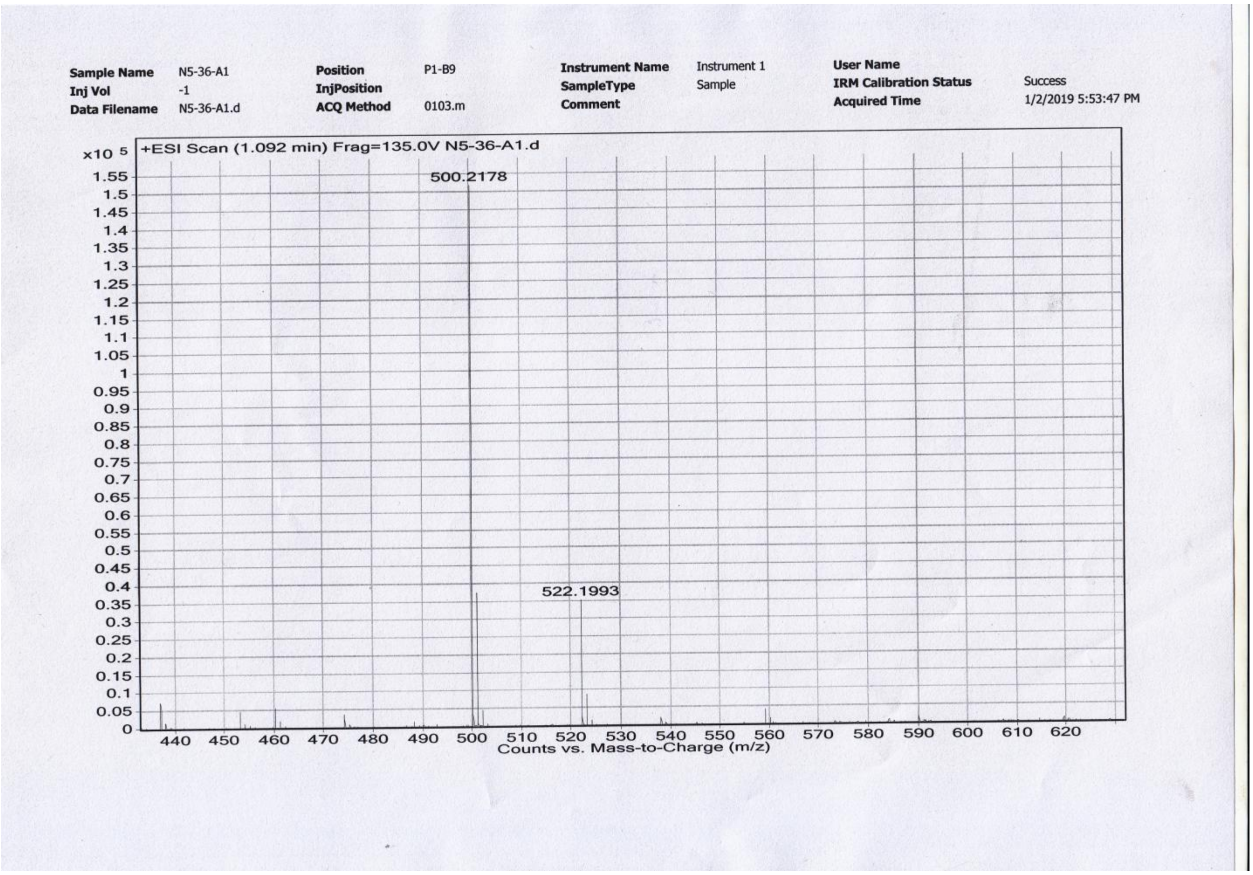

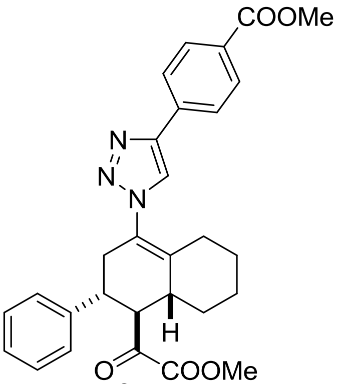

9

Chemical Formula: $C_{29}H_{29}N_3O_5$

Exact Mass: 499.2107

Molecular Weight: 499.5577

m/z: 499.2107 (100.0%), 500.2141 (31.4%), 501.2174 (4.7%), 500.2078 (1.1%), 501.2150 (1.0%)

HRMS exact mass calcd for $C_{29}H_{30}N_3O_5$ $[M + H]^+$ **500.2185**, found **500.2178**.

Supplementary Figure 119. ^{1}H and ^{13}C NMR spectra for 10

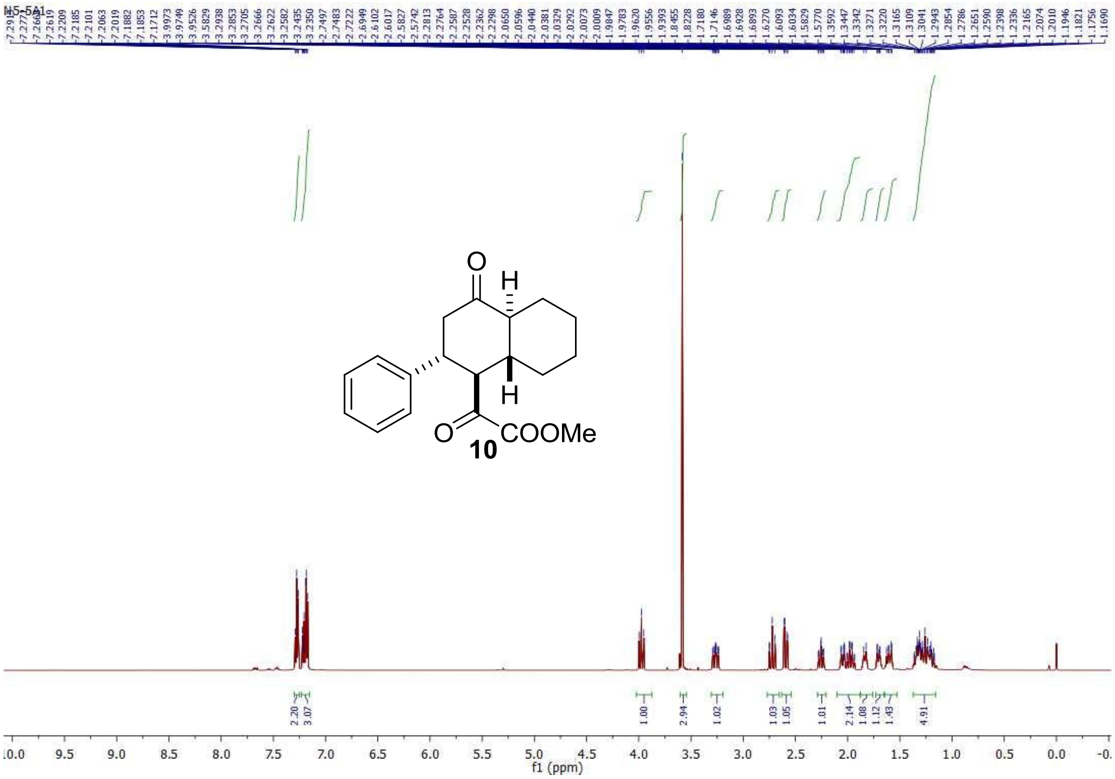

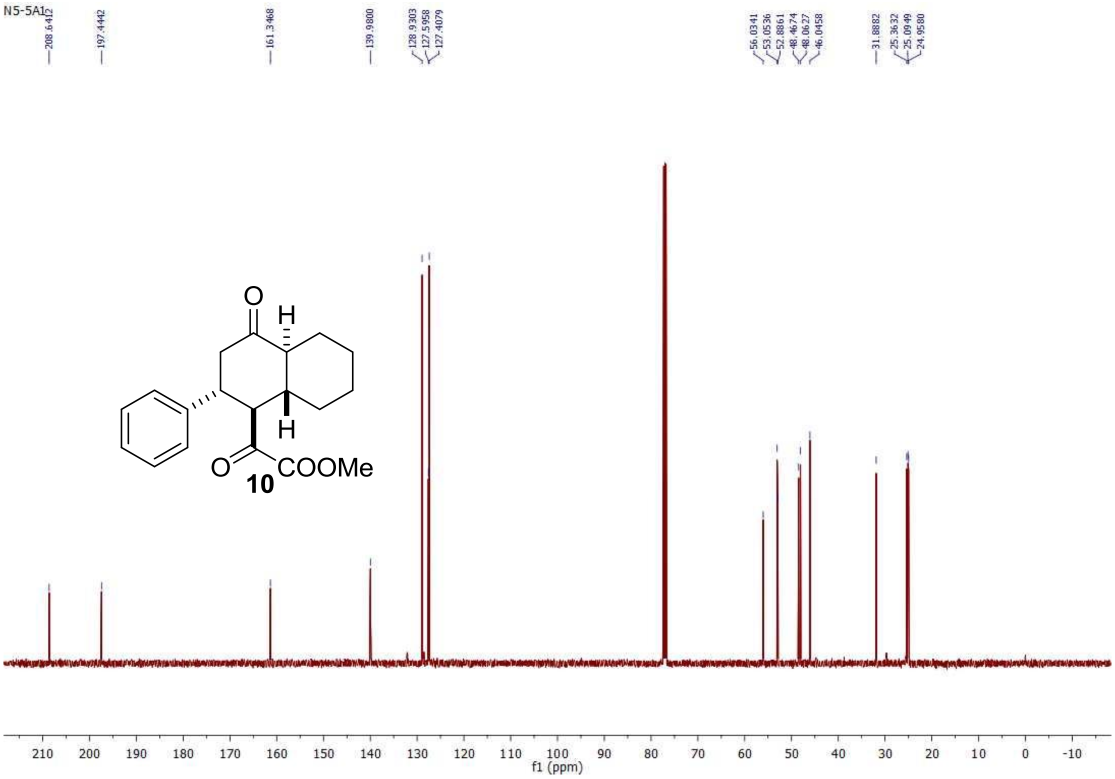

Supplementary Figure 120. HRMS spectra for 10

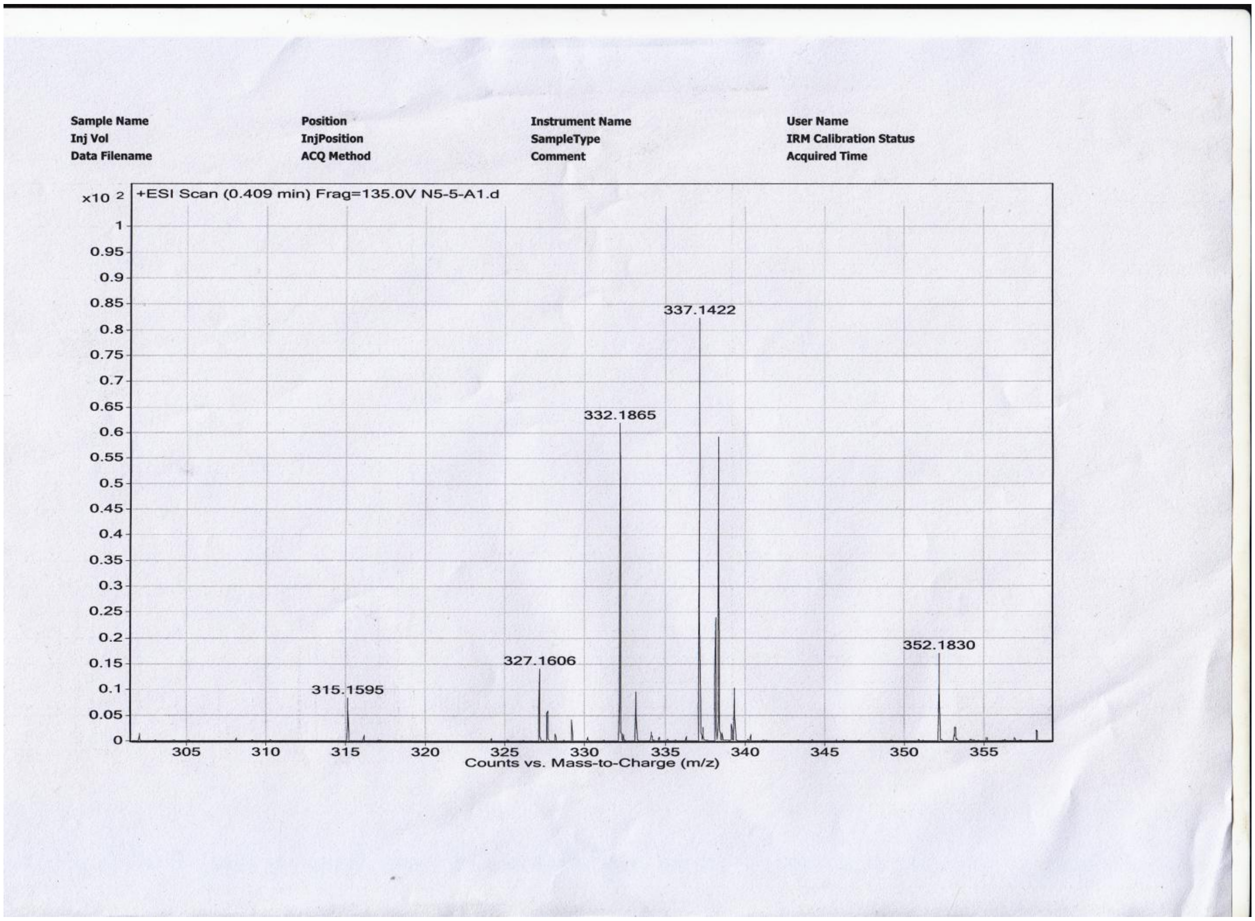

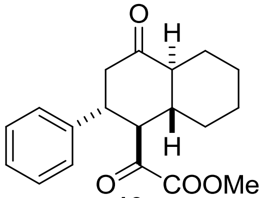

10

Chemical Formula: $C_{19}H_{22}O_4$
Exact Mass: 314.1518
Molecular Weight: 314.3756
m/z: 314.1518 (100.0%), 315.1552 (20.5%), 316.1585 (2.0%)

HRMS exact mass calcd for $C_{19}H_{22}NaO_4$ $[M + H]^+$ **337.1416**, found **337.1422**.

Supplementary Figure 121. ^{1}H and ^{13}C NMR spectra for L9

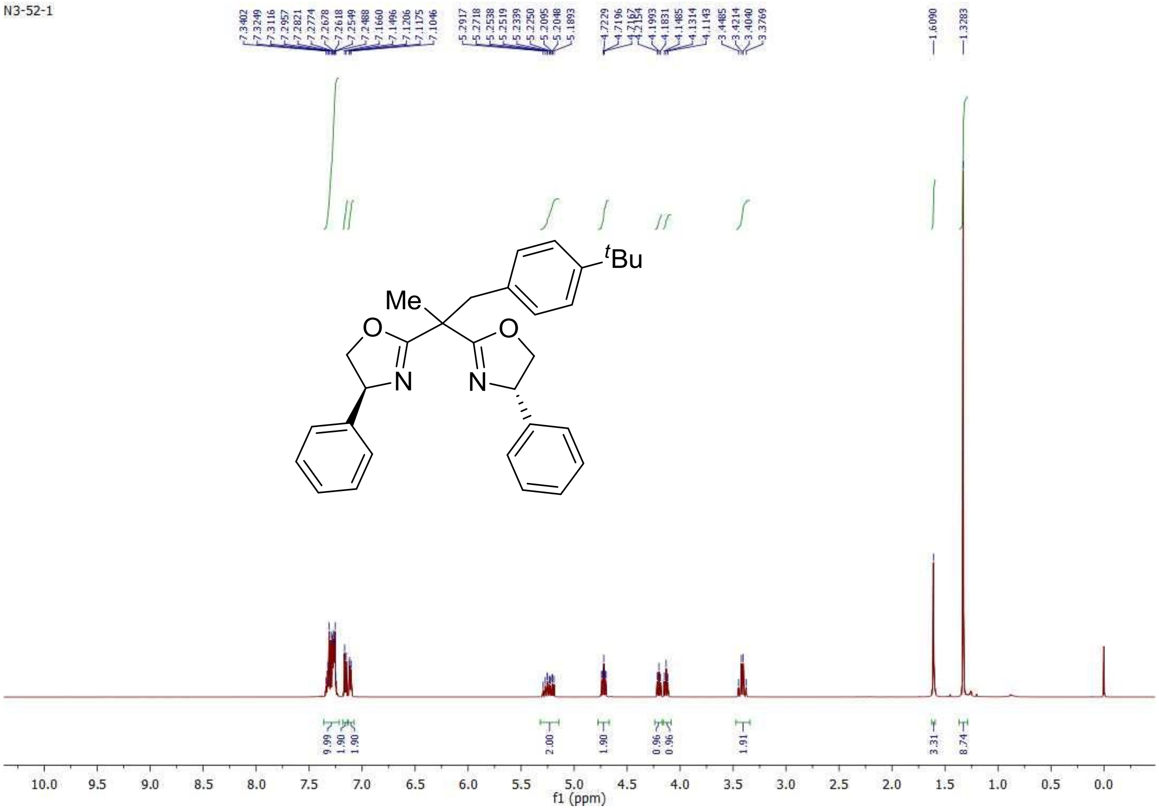

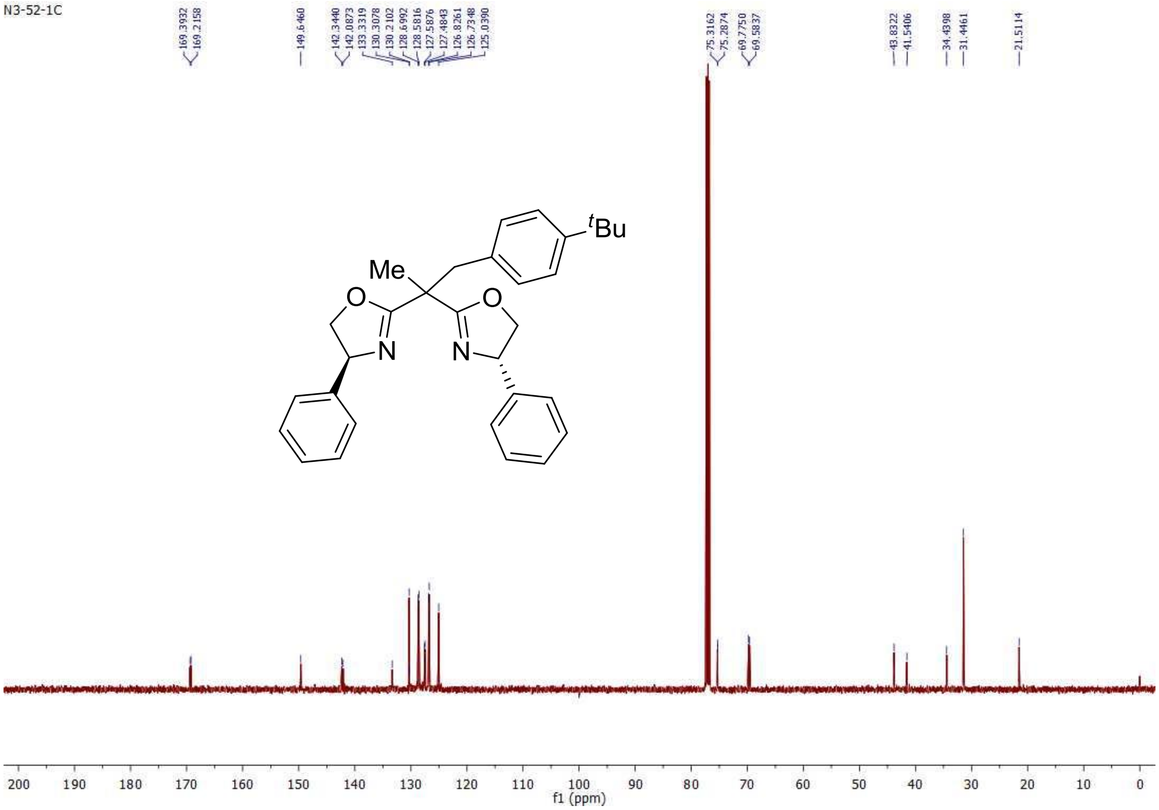

Supplementary Figure 122. HRMS spectra for L9

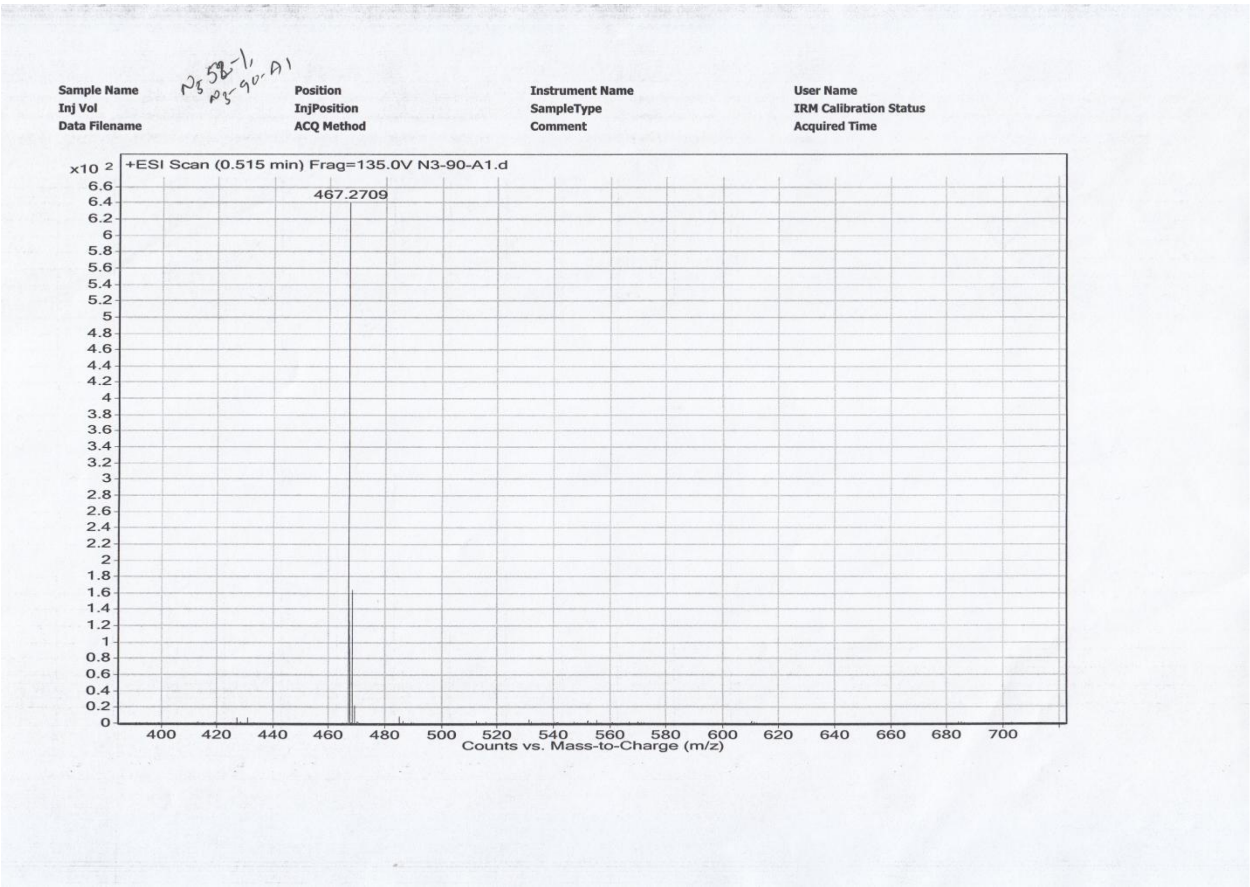

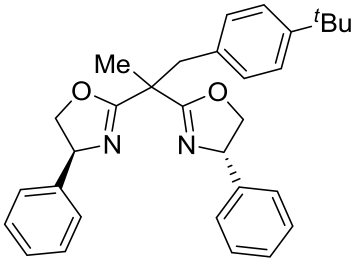

Chemical Formula: $C_{31}H_{34}N_2O_2$
Exact Mass: 466.2620
Molecular Weight: 466.6139
m/z: 466.2620 (100.0%), 467.2654 (33.5%), 468.2687 (5.4%)

HRMS exact mass calcd for $C_{31}H_{35}N_2O_2$ $[M + H]^+$ **467.2699**, found **467.2709**.

Supplementary Figure 123. ^{1}H and ^{13}C NMR spectra for L8

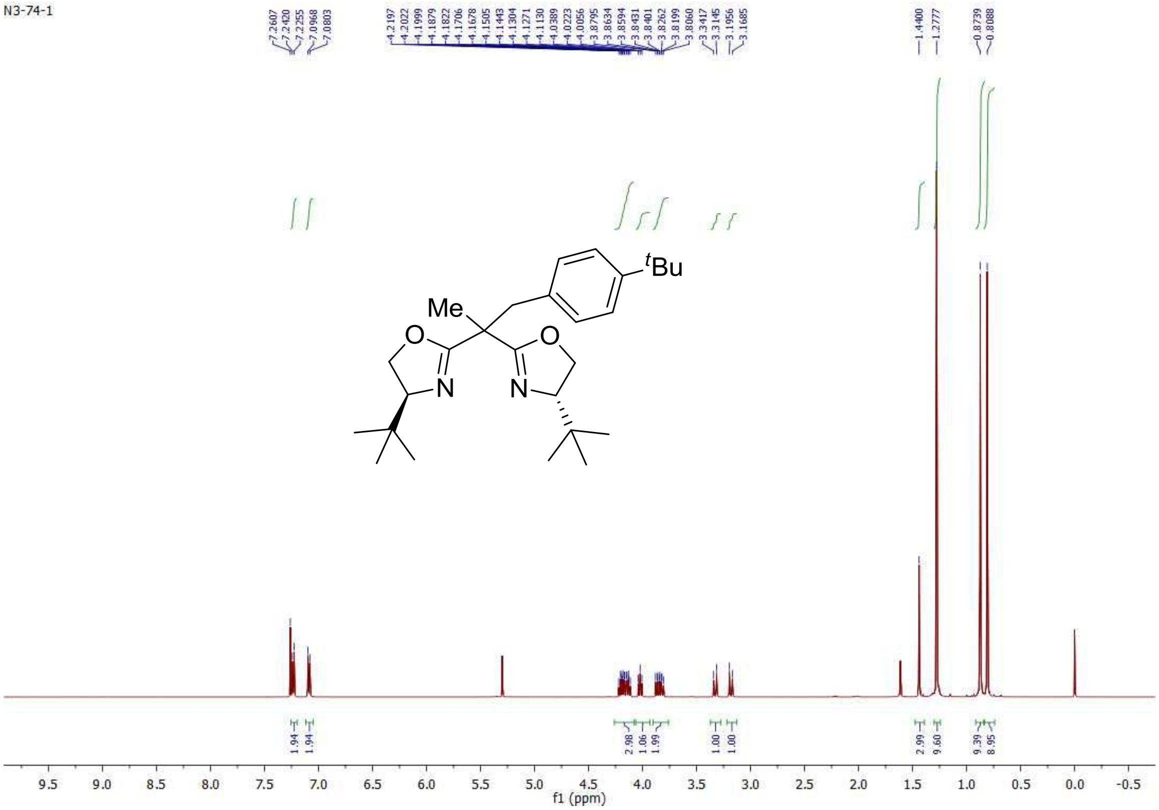

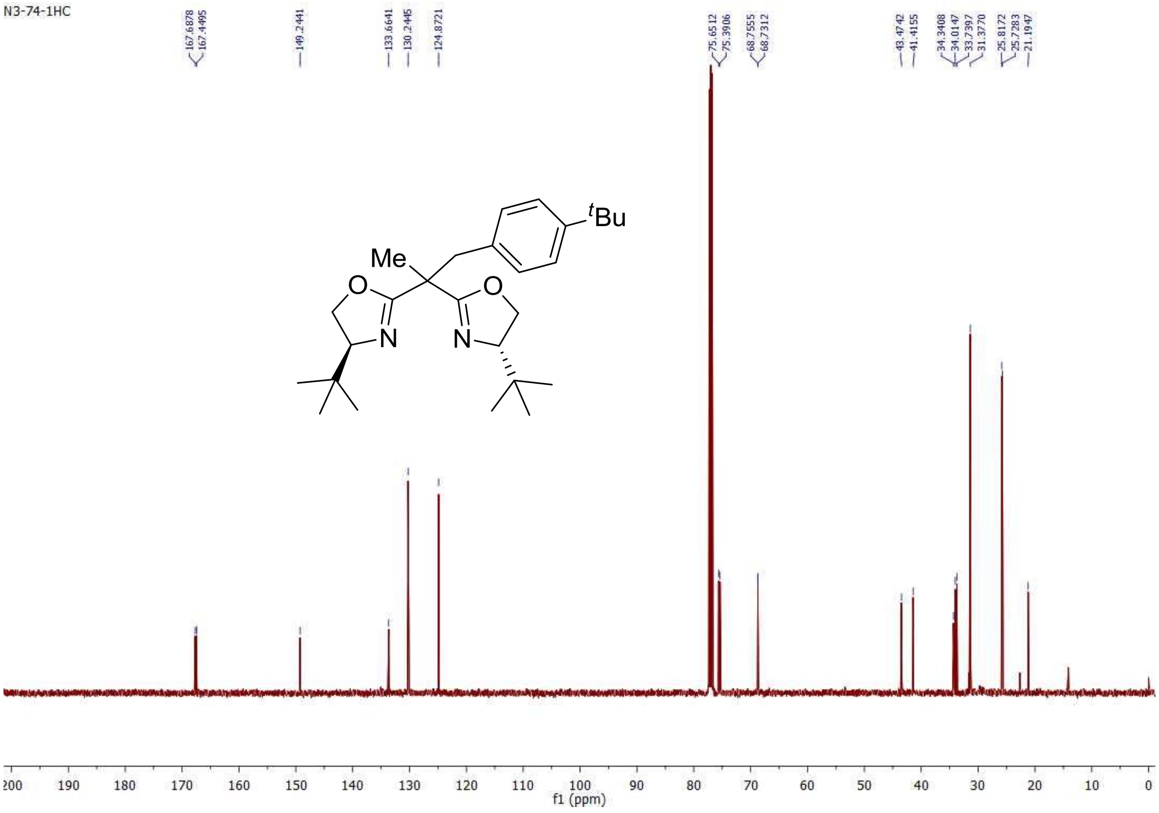

Supplementary Figure 124. HRMS spectra for L8

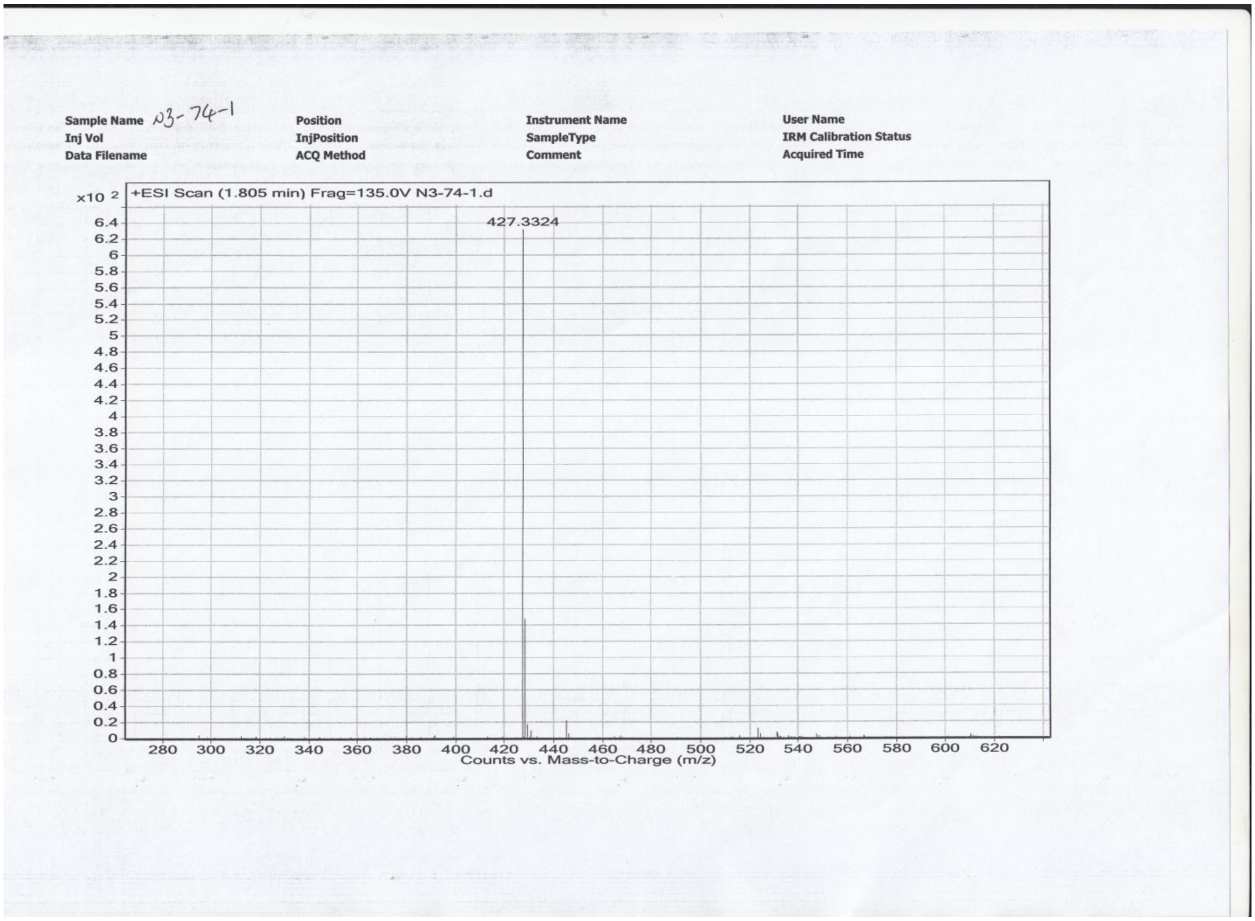

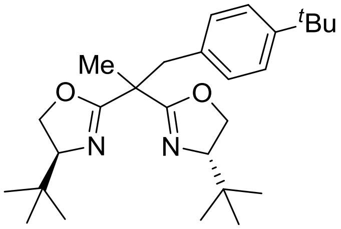

Chemical Formula: $C_{27}H_{42}N_2O_2$
Exact Mass: 426.3246
Molecular Weight: 426.6346
m/z: 426.3246 (100.0%), 427.3280 (29.2%), 428.3313 (4.1%)

HRMS exact mass calcd for $C_{27}H_{43}N_2O_2$ $[M + H]^+$ **427.3325**, found **427.3324**.

Supplementary References

1. Hua, Y. Z., Liu, M. M., Huang, P. J., Song, X., Wang, M. C., Wang, J. B. A new strategy for enantioselective construction of multisubstituted five-membered oxygen heterocycles via a domino Michael/Hemiketalization reaction. *Chem. Eur. J.*, **21**, 11994-11998 (2015).

2. a) Kanchupalli, V., Katukojvala, S. [1+1+3] Annulation of diazoenals and Vinyl Azides: Direct synthesis of functionalized 1-Pyrrolines through olefination. *Angew. Chem. Int. Ed*. **57**, 5531-5535 (2018).

3. Liu, Z., Liao, P., Bi, X. General silver catalyzed hydro azidation of terminal alkynes by combining TMS-N_3 and H_2O: Synthesis of vinyl azides. *Org. Lett*. **16**, 3668-3671 (2014).

4. Denmark, S. E., Stiff, C. M. Effect of ligand structure in the Bisoxazoline mediated asymmetric addition of methyllithium to imines. *J. Org. Chem*. **65**, 5875-5878 (2000).
